# Discovering genotype–phenotype relationships with machine learning and the Visual Physiology Opsin Database (*VPOD*)

**DOI:** 10.1093/gigascience/giae073

**Published:** 2024-10-26

**Authors:** Seth A Frazer, Mahdi Baghbanzadeh, Ali Rahnavard, Keith A Crandall, Todd H Oakley

**Affiliations:** Ecology, Evolution, and Marine Biology, University of California, Santa Barbara, California 93106, USA; Computational Biology Institute, Department of Biostatistics and Bioinformatics, Milken Institute School of Public Health, The George Washington University, Washington, DC 20052, USA; Computational Biology Institute, Department of Biostatistics and Bioinformatics, Milken Institute School of Public Health, The George Washington University, Washington, DC 20052, USA; Computational Biology Institute, Department of Biostatistics and Bioinformatics, Milken Institute School of Public Health, The George Washington University, Washington, DC 20052, USA; Department of Invertebrate Zoology, National Museum of Natural History, Smithsonian Institution, Washington, DC 20012, USA; Ecology, Evolution, and Marine Biology, University of California, Santa Barbara, California 93106, USA

**Keywords:** machine learning, regression, compiled database, genotype–phenotype relationships, predicting phenotypes, spectral sensitivity, color-vision, opsins, imputation

## Abstract

**Background:**

Predicting phenotypes from genetic variation is foundational for fields as diverse as bioengineering and global change biology, highlighting the importance of efficient methods to predict gene functions. Linking genetic changes to phenotypic changes has been a goal of decades of experimental work, especially for some model gene families, including light-sensitive opsin proteins. Opsins can be expressed *in vitro* to measure light absorption parameters, including λ_max_—the wavelength of maximum absorbance—which strongly affects organismal phenotypes like color vision. Despite extensive research on opsins, the data remain dispersed, uncompiled, and often challenging to access, thereby precluding systematic and comprehensive analyses of the intricate relationships between genotype and phenotype.

**Results:**

Here, we report a newly compiled database of all heterologously expressed opsin genes with λ_max_ phenotypes that we call the Visual Physiology Opsin Database (*VPOD*). *VPOD_1.0* contains 864 unique opsin genotypes and corresponding λ_max_ phenotypes collected across all animals from 73 separate publications. We use *VPOD* data and *deepBreaks* to show regression-based machine learning (ML) models often reliably predict λ_max_, account for nonadditive effects of mutations on function, and identify functionally critical amino acid sites.

**Conclusion:**

The ability to reliably predict functions from gene sequences alone using ML will allow robust exploration of molecular-evolutionary patterns governing phenotype, will inform functional and evolutionary connections to an organism’s ecological niche, and may be used more broadly for *de novo* protein design. Together, our database, phenotype predictions, and model comparisons lay the groundwork for future research applicable to families of genes with quantifiable and comparable phenotypes.

Key Points

- We introduce the Visual Physiology Opsin Database (*VPOD_1.0*), which includes 864 unique animal opsin genotypes and corresponding λ_max_ phenotypes from 73 separate publications.
- We demonstrate that regression-based machine learning models can reliably predict λ_max_ from gene sequence alone, predict nonadditive effects of mutations on function, and identify functionally critical amino acid sites.
- We provide an approach that lays the groundwork for future robust exploration of molecular-evolutionary patterns governing phenotype, with potential broader applications to any family of genes with quantifiable and comparable phenotypes.

## Introduction

Although critical to progress in drug and vaccine design [1–3], responses to climate change [4–8], and bioengineering [4, 9–11], accurately predicting gene function from sequences remains a significant challenge. While there are many ways to elucidate genotype–phenotype relationships experimentally, including deep mutational scanning, and *in vitro* heterologous expression with phenotyping, these techniques are often tedious and cost-prohibitive, especially when applied to broad comparative studies of gene families. In addition, accurately predicting the phenotype of a protein using computational methods alone is challenging because of data gaps and the sheer complexity of possible relationships between genes and phenotypes, including epistasis and the nonadditive effects of different mutations. Machine learning (ML) is gaining traction for its potential broad biological applications, accessibility, and faster speeds, especially in biological contexts where phenotype data are abundant and quantifiable. Here, classical regression and classification algorithms are sometimes used to train models for phenotype predictions using genotype–phenotype data [12, 13], while deep learning models can be used to integrate heterogeneous multilayered omics and environmental data for establishing higher-dimensional genotype–phenotype connections [14, 15] or *de novo* protein design [16]. In broader biological contexts, ML models often inform laboratory experiments to predict directional evolution of diseases and their variants [17–19] or to automate image sorting and animal identification from camera trap data [20–22]. In all cases, ML models are a worthwhile long-term investment for genotype–phenotype studies because models can iteratively improve as empirical data accumulate over time.

Such accumulation of important information is exemplified by decades of laboratory work that has led to significant progress in understanding the genetic basis of phenotypic changes for model gene families such as opsins. Opsins are a family of G-protein coupled receptors (GPCR) that bind to a retinal chromophore. The 2 units together, opsin and chromophore, form visual pigments that absorb photons [23]. Opsins have crucial roles in many organismal functions, including circadian rhythms, phototaxis, and image-forming color vision. A critical opsin phenotype is spectral sensitivity—the range of wavelengths to which a gene or organism is sensitive. The main parameter of opsin spectral sensitivity is λ_max_, the wavelength of light (in nm) with maximal absorbance [24]. Common methods of characterizing spectral sensitivities and λ_max_ include organ-level electroretinograms (ERGs) [25–27], cell-level microspectrophotometry (MSP) [28–32], purification of heterologously expressed opsins followed by spectrophotometry [33], and heterologous action spectroscopy using light response assays for opsins expressed in immortalized cell lines [34]. Different opsins are tuned by changes in amino acid sequences to respond to different wavelengths of light, and many previous studies have expressed experimentally mutated opsins and measured spectral sensitivities to establish genotype–phenotype connections [34–38]. Although other factors sometimes affect spectral responsiveness, including the type of chromophore to which an opsin is covalently bound (11-*cis* retinal or 11-*cis*-3,4-didehydro retinal) [39, 40], opsins provide a rare case where an intrinsic molecular function extends rather directly to organismal phenotypes, especially those involving color sensitivity. Despite opsins being a well-studied system with an extensive backlog of published literature, some previous authors expressed doubts that sequence data alone could provide reliable computational predictions of λ_max_ phenotypes [41–44]. At the same time, some λ_max_ predictions showed promise, although on the limited scale of vertebrate cone visual pigments via atomistic molecular simulations [45, 46]. Furthermore, only the nonanimal, microbial, or type 1 (T1) opsins have been systematically cataloged and used to examine genotype–phenotype predictive power of ML models [47, 48]. While some researchers have made significant efforts to compile peak sensitivity data for terrestrial animal photopigments [49] and taxon-specific light-sensitivity data for groups like frogs [50, 51] and ray-finned fishes [52, 53], these efforts currently lack direct links to genetic data that are essential for our current study. Consequently, the extensive data on genotype–phenotype associations of animal opsins remain disorganized, decentralized, often in noncomputer readable formats within older literature, and underanalyzed computationally.

Here, we report a genotype–phenotype database for animal opsins called the Visual Physiology Opsin Database (*VPOD*). We used standard literature searches to compile all heterologously expressed animal opsin genes with spectral sensitivity measurements. We used this newly compiled and harmonized database to evaluate ML methods for connecting genotypes and phenotypes. We created 11 subsets of the overall database to examine factors that impact the reliability and performance of ML models and briefly compared ML predictions to phylogenetic imputation [54, 55]. We also examined whether ML can predict intragenic epistasis, and we predicted amino acid sites particularly important for changing λ_max_. Using our database of 864 unique opsin sequences and corresponding λ_max_ values, we show ML models trained on opsin data accurately predict the λ_max_ of opsins from genetic data alone (highest *R*^2^ = 0.968 with a lowest mean absolute error [MAE] of 6.56 nm), especially when ample and diverse training data are available. ML also predicts some known effects of epistatic mutations on λ_max_. Finally, ML models identify several sites that cause shifts in λ_max_ (e.g., “spectral tuning sites”) and sites known to be structurally important, even in the absence of mutant data in training. When training data are sufficient, these results support the use of ML as a reliable and efficient predictor of λ_max_ for previously uncharacterized opsins, as a tool for identifying candidate spectral tuning sites and epistatic interactions, and as a more general method for linking gene sequences and phenotypes.

## Methods

### Compiling a genotype–phenotype database for animal opsins

**Figure 1:**
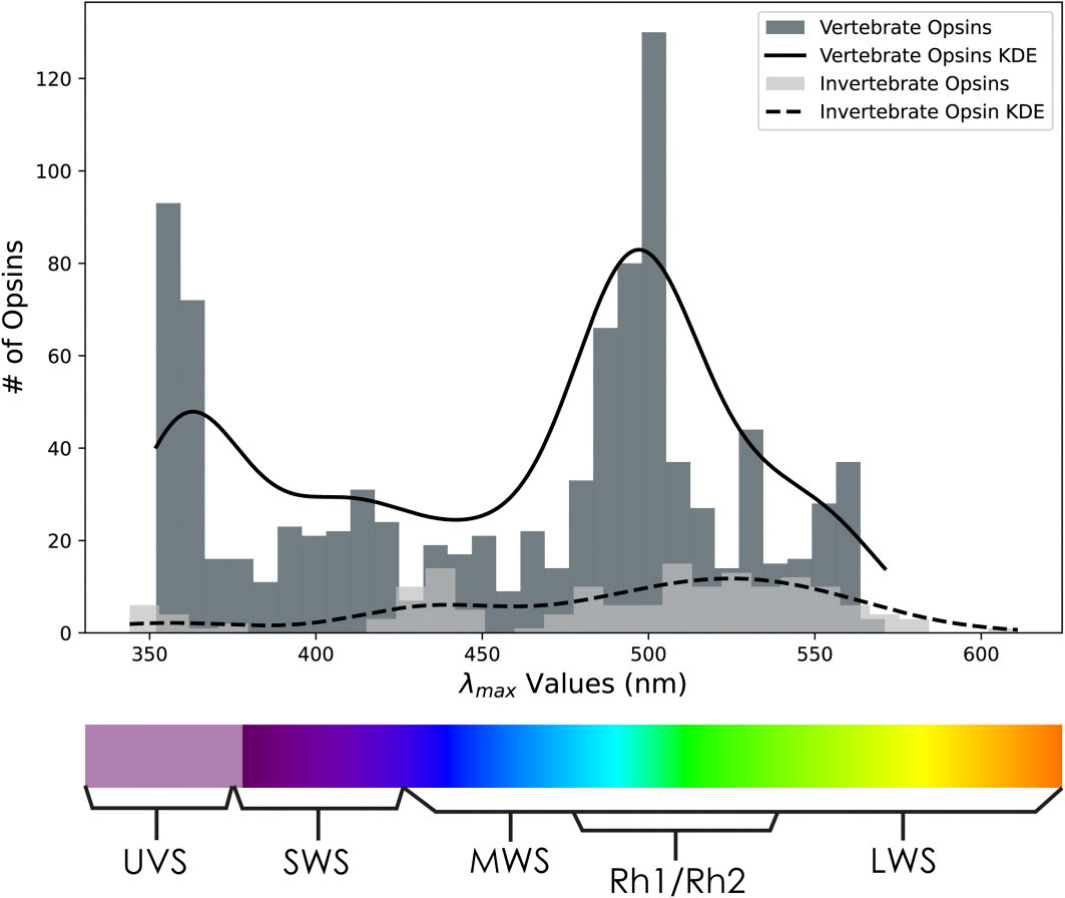
Histogram distributions of vertebrate and invertebrate opsins and absorbance data—λ_max_—from *VPOD_het_1.1* with a scaled kernel density estimate (KDE) curves overlaid to better visualize the general shape and characteristics of our λ_max_ distributions. Note an obvious data bias for vertebrate opsins, especially those with λ_max_ values between 350–375 nm and 480–510 nm, probably due to focal research on UVS and Rh1 opsins.

We collected λ_max_ data for opsins using typical literature review/search methods, with search engine, keywords, and date of access documented in the “*litsearch*” table of the *VPOD* database (RRID:SCR_025668). We cataloged all usable papers with λ_max_ data in the “*references*” table of *VPOD*, recording DOI and a key to link to the search that found the paper. We documented the details of heterologous expression experiments in the “*heterologous*” table, including species, GenBank accession number for the sequence, mutation(s) (if applicable) using a machine-readable notation, λ_max_, cell type for expression (e.g., HEK293, COS1, etc.), protein purification method, type of spectrum (e.g., dark or difference spectrum), and a key to link to the corresponding literature source. Note, we did not record the chromophore used to reconstitute the purified opsin protein because 11-*cis* retinal is the standard and all instances thus far recorded in the “*heterologous*” table are from experiments using 11-*cis* retinal (although future iterations of VPOD could record these details if data with alternative chromophores become available). We input opsin genetic data in an “*opsins*” table, recording opsin gene family names (e.g., long-wave sensitive = LWS, short-wave sensitive = SWS1, etc.). We also included specific “*gene names*” (where applicable), phylum, class, species information, accession number, DNA sequence, amino acid sequence, and the database from which sequences were retrieved (e.g., NCBI). We re-created all mutant and chimeric (e.g., 1 or more transmembrane domains of the mutant copied from a different sequence to replace the original) opsin sequences based on literature descriptions using a pair of Python scripts (*mutagenesis.py* and *chimeras.py*) available on our GitHub [56]. We added all heterologously expressed opsins from the literature to *VPOD*; we call this version of the database *VPOD_1.0*. We refer to heterologous data as *VPOD_het_1.0*, which will allow for future additions to the database to link specific opsin sequences to λ_max_ values established with methods other than heterologous expression, including microspectrophotometry or other methods. During the course of manuscript review, we found and entered 259 new heterologously expressed opsins into *VPOD*, an update we call *VPOD_1.1* (Fig. 1). We decided to keep results from *VPOD_1.0* in the main text because the new data points did not drastically alter any model performances. We also provide this table of performance metrics for *VPOD_1.1* (Supplementary Material 1 (S1)). Therefore, all tests and figures should still be assumed to use *VPOD_1.0* data unless stated otherwise.

### Training ML models with *deepBreaks*

We performed all data preprocessing, including data extraction, sequence alignments, and formatting, in the Jupyter notebooks “*opsin_model_wf.ipynb*,” available on GitHub. We used 2 multiple sequence alignment methods, MAFFT (RRID:SCR_011811) [57] and MUSCLE (RRID:SCR_011812) [58], and a version of both alignments with a Gblocks (RRID:SCR_015945) [59] refinement (for a total of 4 alignments), all set to their default parameters to begin to test the sensitivity of model performance to different alignments. We then trained various ML models employing a custom version of *deepBreaks* [60], an ML tool designed for exploring genotype–phenotype associations. *deepBreaks* takes aligned genotype data (DNA, RNA, amino acid) and some measure(s) of corresponding continuous or categorical phenotype data as input to train ML models. *deepBreaks* uses one-hot encoding to convert amino acid sequences into numerical values. One consequence of this encoding is any amino acids at a given position in the alignment, which are not present at that position in any training data, will be treated equivalently as unseen. For example, cases of only A and V at a highly conserved site in the training set that are presented with a sequence with T at that site will be considered as no A and no V. The models cannot distinguish the input whether it is T or other unseen amino acids at that site. The results produced by *deepBreaks* encompass a compilation of 12 regression ML models [60], showcasing 10 metrics of cross-validation performance (ranked by *R*^2^) and a feature importance report derived from the top-performing models that ranks amino acid positions by their relative importance to each model (from 0.0–1.0, with 1.0 being a site with the highest relative importance) for the phenotype in question (λ_max_). The metrics used to determine these relative importance scores of each position vary based on the structure and output of the algorithms used for model training. For example, xgboost [61] and LightGBM [62, 63] use the number of times a feature appears in a tree as a proxy for importance [60], while AdaBoost [64] and random forest [65, 66], use Gini importance, which quantifies a feature’s contribution to improving prediction accuracy [60, 67, 68]. For a more detailed explanation on how position importance scores are calculated for different models, refer to the “*Interpretation*” heading under the methods section of the *deepBreaks* publication [60]. In addition to *R*^2^, *deepBreaks* reports the MAE, mean absolute percent error (MAPE), mean square error (MSE), and root mean square error (RMSE) for each of the 12 ML models. We evaluated the performance of algorithms based on their relative ranks to look for patterns in which algorithms performed better for different data subsets and approaches. *deepBreaks* also produces a set of distribution box plots (default is 100) to visualize phenotypes (λ_max_) associated with a particular amino acid identity at a site of interest, ordered alphabetically.

### Understanding model performance using different subsets of the database

**Figure 2:**
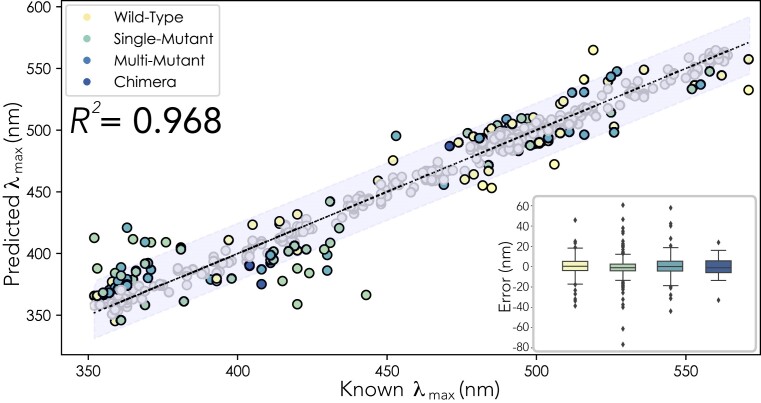
ML model predictions on whole vertebrate opsin dataset, *n* = 721, *R*^2^ = 0.968, MAE = 6.68 nm, MAPE = 1.52. Sequences were iteratively and randomly selected to be withheld from the training dataset (*n* = 50) to act as unseen test data. This was repeated until all sequences had been sampled once. Predictions in which the absolute difference between the “known” and “predicted” λ_max_ are <10 nm are represented by gray dots. All predictions in which the absolute difference between the “known” and “predicted” λ_max_ are >10 nm are represented by colored dots. Yellow dots represent WT predictions, mutants with only a single mutation are green, mutants with greater than 1 mutation are light blue, and chimeric opsins are dark blue. The light gray bar surrounding the trend line represents a 95% confidence interval. Inset: Boxplot distribution of prediction error for different opsin data types from the top-performing vertebrate opsin ML model to better visualize our sources of error. Note, the median for each boxplot hovers around 0 nm. Single mutations have the largest spread of error, but this is most likely due to the high abundance of that data type over all others.

We created 11 data subsets with varying levels of taxonomic and gene family inclusivity (Table 1) to test which factors most impact the reliability/performance of ML methods. We used naming conventions that include versioning to improve reproducibility and reliability of individual datasets and models. For example, 1 subset combines ultraviolet and SWS opsins, which we named *VPOD_uss_het_1.0*. Our convention is to name the subset (in this case USS = “ultraviolet and short-wave sensitive” opsins), name the source of phenotype data (heterologous = het), and record the version number of the dataset (1.0). We also created subsets for medium- and long-wave sensitive opsins (*VPOD_mls_het_1.0*) and all rod (Rh1) and rod-like (Rh2) opsins (*VPOD_rod_het_1.0*). Other subsets use species taxonomy, one for vertebrates (*VPOD_vert_het_1.0*) and another for invertebrates (*VPOD_inv_het_1.0*). For taxonomic subsets, we considered all sequences from phylum Chordata as “vertebrates” and the rest as “invertebrates.” Another subset excludes all mutant opsin sequences, called “wild-types” (*VPOD_wt_het_1.0*). A final named subset is the whole dataset (*VPOD_wds_het_1.0*) (Fig. 2).

**Table 1:** Performance metrics across opsin subsets and top-performing models

Name	Data subset version	# sequences	Top ML algorithm	*R* ^2^ ^a^	MAE (nm)^b^	MAPE (%)^b^	MSE^a^	RMSE^a^
Whole dataset	*VPOD_wds_het_1.0*	864	LGBM	0.947	7.47	1.71	207	13.8
All wild types	*VPOD_wt_het_1.0*	318	Bayesian Ridge	0.902	10	2.18	297	16.5
All mutants	*VPOD_mut_het_1.0*	546	LGBM	0.951	7.89	1.86	194	13.4
Vertebrates	*VPOD_vert_het_1.0*	721	LGBM	0.968	6.56	1.49	111	10.3
WT vertebrates	*VPOD_wt_vert_het_1.0*	274	GBR	0.961	5.46	1.18	82.1	8.36
Invertebrates	*VPOD_inv_het_1.0*	143	LGBM	0.814	14.7	3.22	614	23.1
Rods	*VPOD_rod_het_1.0*	352	Bayesian Ridge	0.834	3.51	0.71	27.7	5.04
WT Rods	*VPOD_wt_rod_het_1.0*	157	GBR	0.783	3.57	0.72	31.9	5.11
MWS/LWS	*VPOD_mls_het_1.0*	91	XGB	0.677	8.77	1.82	317	15
UVS/SWS	*VPOD_uss_het_1.0*	280	GBR	0.821	8.02	2.06	200	13.6
WT UVS/SWS	*VPOD_wt_uss_het_1.0*	66	Adaboost	0.865	7.79	1.87	152	10.6
T1 opsins	*Karyasuyama_T1_ops*	884	Random Forest	0.804	9.41	1.76	186	13.5

a *R* ^2^, mean square error (MSE), and root mean square error (RMSE) are often interpreted as direct measures of comparing/analyzing model performance and used as training loss terms of the objective function—which measures how well the model fits the training data. One has to often balance between this and the regularization term, which controls the complexity of the model. Thus, a high performance is both simple and predictive, a trade-off referred to as the “*bias-variance*” trade-off.

b Mean absolute error (MAE) and mean absolute percent error (MAPE) are in relation to the absolute error λ_max_ predictions and interpreted in the same units of “nm.”

**Figure 3:**
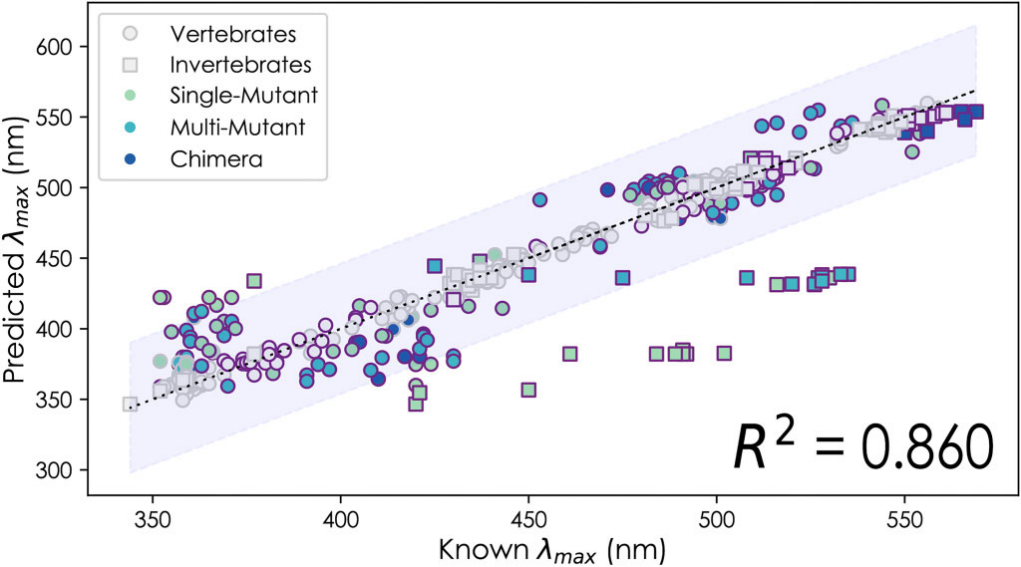
Scatterplot of wild-type model’s λ_max_ predictions for 546 mutant opsins, with an *R*^2^ of 0.860, MAE of 12.36 nm, and MAPE of 2.91%. Mutant predictions in which the absolute difference between the “known” and “predicted” λ_max_ are <10 nm are represented by gray dots. All predictions in which the absolute difference between the “known” and “predicted” λ_max_ are >10 nm are represented by colored symbols, further separated by invertebrate (squares) and vertebrate (circles) opsins. Mutants with only a single mutation are green, mutants with greater than 1 mutation are light blue, and chimeric opsins are dark blue. Mutations that caused a shift of >10 nm from the WT are outlined in purple. The light gray bar surrounding the trend line represents a 95% confidence interval.

Using various subsets of data, we performed a number of experiments to better understand the performance of ML models in predicting λ_max_. First, to better understand how training data relate to model performance, *R*^2^, and training data size, we gradually increased the size of training datasets by starting from zero and incrementally adding between 15 and 50 randomly selected sequences at a time for the whole dataset (WDS), vertebrate, wild-type (WT), and rod subsets separately, repeating the process 3 times per subset (Supplementary Material 2 (S2)). We then analyzed the fit between the size of training datasets (x-axis) and model performance (y-axis), comparing 6 nonlinear models with Akaike information criterion (AIC) to find the model that best explains the observed variation (Supplementary Material 3 (S3). Second, to understand if ML could predict known phenotypic changes due to experimental mutations, we queried the top-performing WT model (which lacks data from artificially mutated sequences) using all experimentally mutated opsins to predict their known phenotypes. We plotted these results using *matplotlib* [69] to visualize characteristics of poorly predicted outliers (e.g., taxonomic bias or sensitivity to mutations, which caused large shifts in λ_max_ from the WT) (Fig. 3). To test further whether including these mutant data significantly improves predictions of λ_max_, we used the *VPOD_het_1.1* dataset (Supplementary Material 1 (S1)) and a *Wilcoxon signed-rank test* [70, 71] to compare distributions of squared error for predictions by the WDS model (contains mutant data) and WT model (no mutant data) on all mutant data (*n* = 761) and separately comparing only mutants causing the largest phenotypic changes in λ_max_ (>10 nm from the wild-type; *n* = 346). To accomplish this for the WDS models, we iteratively removed 25 mutant opsins at a time from training data, used the same training algorithm (gradient boosted regressor [GBR]), and predicted λ_max_ values of withheld opsins following the completion of model training (withheld opsins are not used as test data during the actual model training), until all mutant opsins were sampled once (this notebook is available on GitHub as “*vpod_wf_iterate_subsample.ipynb*.” Third, we examined the ability of our models to predict λ_max_ of 30 invertebrate opsins not in *VPOD_1.0* because they are only known from physiological studies (Supplementary Material 4 (S4), Supplementary Material 5 (S5)). Here, we collected data both characterized by single-cell microspectrophotometry (MSP) or electroretinogram methods and with expression localized to cell type by *in situ* hybridization (ISH), to link λ_max_ to a specific opsin (the sequences and metadata can be found in “*msp_erg_raw.txt*” and “*msp_erg_meta.tsv*,” while the resulting predictions can be found under the “*msp_tests*” folder on our GitHub repository). Finally, we directly compared predictive capabilities of models trained on different data subsets by randomly selecting and removing the same 25 wild-type ultraviolet or short-wave sensitive opsins from the training data of the WDS, vertebrate, WT, and ultraviolet sensitive (UVS)/SWS models before training and querying the model with those same sequences following training (Supplementary Material 4 (S4), Supplementary Material 6 (S6)).

### Comparing machine learning and phylogenetic imputation

We compared performance of ML models to phylogenetic imputation, which estimates phenotypes using phylogenetic information [54, 55]. Phylogenetic imputation uses maximum likelihood (we will not abbreviate maximum likelihood as ML to avoid confusion with machine learning), usually assuming Brownian motion to predict missing phenotypes using a phylogenetic tree, such that more closely related species or sequences have more similar phenotypes. For the phylogeny, we constructed opsin gene trees in phyML [72], assuming the “WAG” substitution model [73] and a proportion of 0.029 invariable sites, with Gamma as a rate across sites model, and 4 substitution rate classes. We randomly removed 50 opsin sequences and their corresponding λ_max_ values from each of the ML training datasets (with the exception of the smaller medium wavelength-sensitive (MWS)/LWS and invertebrate datasets, where we only removed 15), then estimated the removed λ_max_ values using phylogenetic imputation. We used the phylogenetic imputation submodule of the *phytools* R package [74] for imputation. We compared imputed and actual λ_max_ using regression. Imputation seemed sensitive to input alignment, perhaps caused by very short or zero length branch lengths in the phylogeny, as we could only complete imputation with *phytools* after removing uninformative and heavily gapped regions with Gblocks. To allow direct comparisons of regressions between imputation and ML, we re-created ML training–data alignments using MAFFT, MUSCLE, and Gblocks in the same way as for imputation and predicted λ_max_ for the same sets of sequences as imputation (Supplementary Material 7 (S7)).

### Testing ability of ML to account for intragenic epistasis

Functional predictions are often misled by epistasis [41], so we tested the ability of our WDS models to predict the effects of epistatic mutations by haphazardly selecting 3 double mutants with previously demonstrated epistatic effects from training data in which double mutants, each single mutant, and wild-type sequence are all characterized by heterologous expression. The 3 epistatic double mutants are all derived from bovine rhodopsins: D83N_A292S, F261Y_A269T, and A164S_A269T. We removed the double mutants from the training dataset but retained single mutants to test whether the model treats the mutations as additive or epistatic. We hypothesized that the many instances of multimutant sequences with epistatic effects in the training set would allow the model to account for both the magnitude and direction of intragenic epistasis. We then ran a separate test where we removed the same double mutants plus their corresponding single mutants to observe whether the WDS model still predicts epistatic effects from wild-type data alone. We subsequently repeated this same process for the WT and vertebrate models (Supplementary Material 8 (S8)).

We ran an additional experiment to test the general ability to to predict epistatic interactions between mutations for all available data. Here, we identified all multimutants that have phenotype data for each individual component mutation. Next we selected those multimutants with nonadditive (epistatic) interactions between mutations (which we define as >1 nm difference between the actual multimutant phenotype and the sum of changes in phenotype due to the individual mutations). These 111 “epistatic mutants” were then all removed from WDS (*VPOD_wds_het_1.1*) to create a new training dataset called “WDS-minusepi” that lacks evidence of intragenic epistasis. For this test, we hypothesized that if the ML approach can account for epistasis, the RMSE of predictions of the 111 epistatic mutants would be significantly lower for the model trained with WDS-minusepi than the model trained with no mutants at all (WT). We tested for statistically significant differences in the distributions of square error for predictions made by WDS-minusepi versus WT and WDS-minusepi versus the epistasis-free additive mutation values (EAMVs, which represent the expected λ_max_ for mutants if the effects of their singular mutational components were treated as additive). We also predicted a statistically significant difference between predictions made by WT and EAMV only if WT contains enough natural variation (not based on mutants) to observe patterns of intragenic epistasis. These statistical tests assumed a Bonferroni correction for multiple tests.

### Identifying known spectral tuning sites

In addition to predicting λ_max_, we wanted to identify amino acid sites with strong effects on the phenotype, called spectral tuning sites for opsins. To do so, *deepBreaks* produces an “importance report” of the relative importance of amino acid positions within the sequence relative to the phenotype. This report is generated for each of the top 3 performing models, with the addition of a column that calculates the “mean relative importance” value of each individual position. We automated the translation of these feature representations of aligned amino acid positions compared to bovine rhodopsin for the sake of interpretability. We also included the amino acid residue identity at each corresponding position and whether it is in one of the opsin transmembrane domains (TMDs). We used this to provide us with a standardized context for analysis of the most significant positions highlighted by the models, which we could use to compare to published mutants and known spectral tuning sites. We analyzed the importance report for each model to see what positions it highlighted as most important, with an extra emphasis placed on the output for the WT models since it was the least likely to be biased by the presence of already known mutant data (Supplementary Material 9 (S9)), as previous researchers often chose suspected tuning sites for mutagenesis experiments.

## Results

### Data description: A genotype–phenotype database for animal opsins

*VPOD* is a new database, available on GitHub and in *GigaDB* [75] that currently includes all heterologously expressed animal opsins. We refer to a subset of the database with only heterologous data as *VPOD_het_1.0*, although for version 1.0, this is synonymous with the entire database. *VPOD_het_1.0* relies on 73 publications, mainly primary sources, with dates ranging from the 1980s to 2023. The database contains opsin sequences and phenotype data from 166 unique species (counting 35 reconstructed ancestors), including fishes, amphibians, reptiles, mammals, crustaceans, and bivalves. Altogether, *VPOD_het_1.0* contains 864 unique opsin sequences and corresponding λ_max_ values. This includes 318 unique WT opsins and 546 unique experimentally mutated opsins (447 from vertebrates and 99 from invertebrates) from 82 species (73 vertebrate and 9 invertebrate species). Of the mutants, 73 are “chimeric,” meaning 1 or more transmembrane domains of the mutant are copied from a different opsin to replace the original. Phylogenetically, *VPOD_het_1.0* is mainly vertebrate opsins (*n* = 721), with only 143 unique invertebrate opsins (Supplementary Material 10 (S10)). The vertebrate opsins consist of 113 UVS opsins, 167 SWS opsins, 8 MWS opsins, 83 LWS opsins, 237 rhodopsin (Rh1) opsins, and 113 rhodopsin-like (Rh2) opsins (Supplementary Material 10 (S10)). Phenotypically, *VPOD_het_1.0* spans a range of λ_max_ values from 350 to 611 nm. The highest concentration of phenotype values are between 350–375 nm and 475–525 nm (Fig. 1) due to the literature bias favoring characterization of UVS/SWS opsins and rhodopsins (Rh1).

### The data used for model training strongly impact accuracy

Several models trained with different subsets of data predicted λ_max_ with high accuracy (Table 1). The top-performing models from these subsets consistently used the same 5 algorithms, including the gradient boosting regressor (GBR) [68, 76], Bayesian ridge (BR) [77, 78], light gradient boosting machine (LGBM) [79], random forest (RF) [66], and extreme gradient boosted machine (XGB) [61]. For example, *VPOD_vert_het_1.0*—trained with all vertebrate wild-type, mutant, and chimeric opsins—had the highest 10-fold cross-validation (CV) *R*^2^ (0.968) and lowest MAE (6.56 nm) of any models we compared (Fig. 2). Similarly, *VPOD_wds_het_1.0*, trained with the whole dataset, had very high *R*^2^ (0.947) and low MAE (7.47 nm). The 2 data subsets also shared the same 5 top-performing models (GBR, BR, LGBM, RF, and XGB). In addition, *VPOD_wt_het_1.0*—trained without mutants and only wild-type data—had a similarly high *R*^2^ (0.902) and a low MAE (10.3 nm) when predicting unseen wild-type data. Overall, this “wild-type-only” model also fared well, even when predicting mutant data not included in the model (Fig. 3). While these performance metrics are impressive, it is important to remember that phylogenetic relatedness between sequences of a dataset could inflate values, like *R*^2^, when using random sampling for cross-validation because opsins that are more similar to those in the training data will be easier to predict, and phylogenetically clustered sequences will also be more likely to be resampled. Roberts et al. [80] provide a discussion of alternative cross-validation strategies such as “block cross-validation” for nonindependent data types, including phylogenetically related data, which can help mitigate this issue. Despite overall high *R*^2^, we noticed multiple instances where mutations that cause large shifts in λ_max_ (>10 nm) were not well predicted by the wild-type-only model, as indicated by large residual values for the predictions of these mutant sequences (Fig. 3). We found including mutant data significantly improves predictions of λ_max_ when comparing predictions of models trained with (WDS) and without (WT) mutant data and rejecting the null hypotheses of no underlying differences between the distribution of squared error for predictions of all mutants (*P* = 9.96e-22, WDS RMSE = 12.6 nm, WT RMSE = 17.6 nm) (Supplementary Material 11 (S11)) and when predicting phenotypes of mutants with large shifts in λ_max_ (*P* = 2.29e-25, WDS RMSE = 17.0 nm, WT RMSE = 24.2 nm) (Supplementary Material 11 (S11)).

In addition to including mutant data, data availability more generally improves predictive power, with performance thresholds and plateaus depending on the genetic diversity of the training data. Overall accuracy in predicting λ_max_ for our models trained on more genotypically and phenotypically complete subsets of data (WDS, vertebrate, WT) improves as a function of the number of sequences in a dataset and shows an initial plateau (*R*^2^ = ∼0.80–0.90) of diminishing returns around 120 to 200 sequences that continues to taper off above 200 sequences (Supplementary Material 2 (S2), Supplementary Material 3 (S3). Consistent with a rough performance threshold, we found models from data subsets with fewer than ∼200 training sequences to far less accurately predict λ_max_. For example, *VPOD_mls_het_1.0*—trained only on the 91 MWS/LWS opsins of vertebrates—and *VPOD_inv_het_1.0*—trained only on 144 invertebrate opsins—showed among the lowest *R*^2^ (0.677 and 0.814, respectively; Table 1). For all data subsets, we found the relationship between number of sequences in a dataset and model performance best fits a reciprocal model, which is suitable when the dependent variable plateaus as the independent variable grows larger. We found the coefficients of the reciprocal equations to be different between data subsets and to increase in negative magnitude with a decrease in taxonomic/genetic diversity (the rod model holding the largest negative value of −44). These equations do not account directly for taxonomic, genetic, or phenotypic diversity, as the raw number of genes is the value of the x-axis. Therefore, one should be cautious about applying them to predict model performance based on training data size alone.

The complicated relationship between size of training dataset and predictive power is further illustrated by models from some larger data subsets that resulted in rather poor predictions. One large dataset (884 sequences), the previously published Karyasuyama type 1 opsin dataset (*Karyasuyama_T1_ops* [47]), showed only moderate *R*^2^ (0.804) and MAE (9.41), similar to models from the much smaller invertebrate data (Table 1). One explanation for lower predictive power could be that the very old age of T1 opsins led to a higher complexity and diversity of genotype–phenotype associations, which are not yet completely sampled enough to allow good predictions. In addition, models based on rod, UVS/SWS, and MWS/LWS subsets tend to show lower *R*^2^ than might be at first expected (Supplementary Material 2 (S2), Supplementary Material 3 (S3), especially since these 3 datasets together comprise the training data for the vertebrate model (our highest performing model, *R*^2^ = 0.968). For example, the rod model, with 352 sequences, should have resulted in a model with an *R*^2^ around 0.900 to 0.960 based on the trend lines for the WDS and vertebrate datasets (Supplementary Material 2 (S2), Supplementary Material 3 (S3) but resulted in an *R*^2^ = 0.831. A possible explanation for this lower *R*^2^ value for rod models is the small degree of variability in λ_max._ When variation is low, even very small differences from model predictions could lead to larger differences in *R*^2^. Therefore, when a data subset such as rod opsins contains limited variability in the response variable (λ_max_), additional metrics that are less sensitive to variance will be important, such as MAE or RMSE, which report the absolute magnitude of errors rather than the proportion of explained variance. To illustrate further, most models tested on their ability to predict the λ_max_ for a set of 25 subsampled WT-SWS opsins from *VPOD* performed relatively poorly based on *R*^2^ alone (Supplementary Material 4 (S4)), with the vertebrate model (*R*^2^ = 0.914, MAE = 7.89) demonstrating a relatively greater predictive power than all other models (Supplementary Material 4 (S4), Supplementary Material 6 (S6)). However, between the vertebrate and lowest performing model (SWS model; *R*^2^ = 0.778, MAE = 11.6 nm), there is only a 3.71-nm increase in MAE, a much less dramatic perceived shift in performance than might be interpreted from *R*^2^ alone.

When predicting λ_max_ of 30 unseen wild-type invertebrate opsins from a separately curated MSP dataset, almost every model performed rather poorly, with exception of the WT model (*n* = 30, *R*^2^ = 0.887, MAE = 17.5) (Supplementary Material 4 (S4), Supplementary Material 5 (S5)). The best-performing model produced by the sparsely populated “*Invertebrate*” dataset could only predict unseen invertebrate opsins with an *R*^2^ of 0.837 and MAE of 26.3 nm (Supplementary Material 4 (S4), Supplementary Material 6 (S6)). Until the models are trained with more invertebrate (r-opsin) data, we would not put high confidence in the estimates of λ_max_. Furthermore, these separately curated invertebrate opsins are independent of the phylogenetic relatedness of the data used in model training and therefore provide a less inflated estimate of the ability to predict λ_max_ compared to random resampling of training data. Because of the sparsity of invertebrate data in the training set, this result further highlights that opsins more distantly related to those in the database will be more difficult to predict.

### ML predictions of λ_max_ are comparable to phylogenetic imputation

Both ML and phylogenetic imputation were often accurate predictors of λ_max_ (Supplementary Material 7 (S7)). When using the same test data, ML models usually outperformed phylogenetic imputation, however slightly (Supplementary Material 7 (S7)), albeit using far less computational time: ML used on the order of minutes to calculate models, and imputation used on the order of hours to generate opsin phylogenies. The MWS/LWS dataset was the only instance where phylogenetic imputation (*R*^2^ = 0.784) largely outperformed ML (*R*^2^ = 0.512). We found our implementation protocol for phylogenetic imputation required removing aligned sites with extensive gaps (for which we used Gblocks); we speculate this lessened the impacts of very short branch lengths on model fitting during imputation. To allow direct comparisons between approaches, we also used the same trimmed alignments for training ML models. Interestingly, there was a slight but noticeable decrease in ML performance following Gblocks trimming for the invertebrate, MWS/LWS, and UVS/SWS datasets (Supplementary Material 7 (S7)). The *R*^2^ of the MWS/LWS model dropped from 0.677 to 0.645, while the invertebrate model dropped from 0.814 to 0.797 (Supplementary Material 7 (S7)). ML performance remained relatively consistent after tripping for the WT, vertebrate, WDS, SWS/UVS, and rod models, with only a slight reduction in *R*^2^ (<0.01) and slight increase in MAE (±1 nm) for the WT model. We speculate the observed differences in ML performance following Gblocks processing is due to the reduced number of features in the datasets from removing aligned sites.

### ML often predicts the effects of epistatic mutations

The WDS successfully predicted 3 out of 3 individual instances of epistasis (Supplementary Material 8 (S8)) using sequences that were removed from the training data before using the model to predict known epistatic phenotypes. For double mutant D83N_A292S, the model predicted 485.2 nm, which was 0.2 nm off the known λ_max_ of 485 nm. If the WDS model believed the sites were additive, the resulting λ_max_ based on adding shifts of single mutants would have been much lower, at 477.5 nm. Second, for mutant F261Y_A269, the model predicted 520.0 nm, for which the known λ_max_ was 520 nm. An additive prediction would have been higher, 524 nm. Third, for mutant A164S_A269T, the model predicted a λ_max_ of 515.5 nm, where the known λ_max_ was 514 nm. This is a special case in which the double mutant experiences a form of epistasis where the effect of mutation A269T (λ_max_ = 514) masks the shift otherwise caused by mutation A164S (λ_max_ = 502 nm). Thus, the model correctly predicted an instance of epistasis in which one mutation masks the effect of another.

We also queried the WT model with these same 3 double mutants to test the importance of mutant sequences in informing the model on epistatic interactions. However, without any mutant data at all, the WT model did not display the same abilities to predict epistasis in any instance. For the double mutant D83N_A292S, the model predicted that neither the individual mutations nor the double mutant would have a significant effect on λ_max_, and all were predicted to be 499.9 nm. For double mutants F261Y_A269 and A164S_A269T, the WT model successfully predicted all individual mutations would cause a red shift (although F261Y and A269 were >3 nm off their known λ_max_) but incorrectly treated the mutational effects as additive for the double mutant (Supplementary Material 8 (S8)).

Our broader experiment to test the predictability of epistatic effects using the WDS-minusepi model (which excluded from training all 111 opsins with known nonadditive mutational effects, which we call epistatic opsins) correctly predicted epistasis for 105 of 111 of the epistatic opsins with higher *R*^2^ (0.969) and much lower RMSE (12.4 nm) than predictions by the WT model (*R*^2^ = 0.894, RMSE = 22.3 nm), which contains no experimentally mutated opsins, and the EAMV (*R*^2^ = 0.878, RMSE = 29.8 nm), which ignores epistatic effects, respectively (Supplementary Material 12 (S12)). Our test of the null hypotheses of no underlying differences between the distribution of squared error for predictions of the 111 epistatic mutants were rejected after Bonferroni correction by the WDS-minusepi model versus WT model (*P* = 1.24e-06) and WDS-minusepi model versus EAMV (*P* = 2.56e-09) but not rejected for the WT model versus EAMV (*P* = 0.086) (Supplementary Material 12 (S12)). Together, the large differences in RMSE and the results of the statistical tests strongly support the idea that the inclusion of even single mutants significantly reduces the error of ML models when predicting epistatic interactions between mutations and that this error is also less than the error we would observe if our models simply treated mutations as additive. Nevertheless, the insignificant difference between WT predictions and EAMV indicates there is not enough information about epistatic interactions in wild-type (nonmutant) data alone to accurately predict intragenic epistasis.

### ML predicts tuning sites from wild-type sequences alone

**Figure 4:**
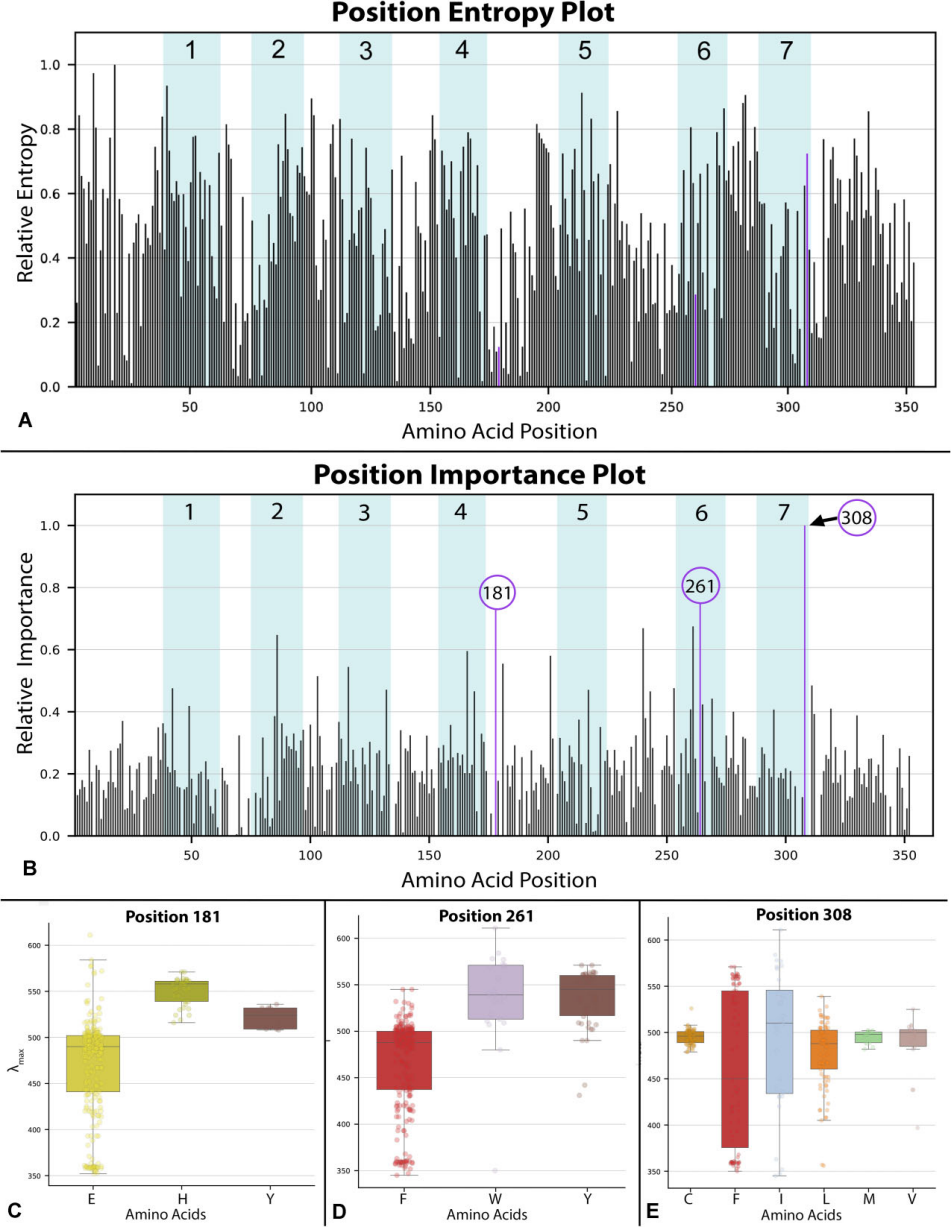
(A, B) Blue bars indicate the 7 transmembrane domain regions of the bovine rhodopsin and are labeled accordingly. Purple bars indicate the top 3 most important positions to predictions of λ_max_ by the “BayesianRidge” ML regression model trained on the WT opsin dataset. (A) Bar graph of relative entropy scores by position calculated via Shannon entropy [71, 88, 89] using the multisequence alignment for the WT data subset. (B) Bar graph of relative importance by position generated via “BayesianRidge” ML regression model trained on the WT opsin dataset. We interpret positions with higher relative importance as having a larger effect or weight on λ_max_ prediction. Positions 181 [35, 83], 261 [87, 91], and 308 [81] are highlighted in purple because they are among the highest scoring sites and have all been previously characterized as functionally important to opsin phenotype and function. Based on an *R*^2^ of 0.001, there is no linear relationship between relative entropy by position and the relative importance of scores by position. (C–E) These distribution box plots provide a visualization for which amino acid (aa) residues at a particular site are associated with different ranges of lambda max at a site of interest, ordered alphabetically, not by frequency (left to right). For a more detailed explanation on how position importance scores are calculated for different models, refer to the “Interpretation” heading under the methods section of the *deepBreaks* publication [60].

The full WT model and its few variants (SWS and rod WT models) predict several previously characterized “spectral tuning sites”—functionally demonstrated to change λ_max_—even with no information on mutants used in the training data (Fig. 4, Supplementary Material 9 (S9)). For the primary WT model alone, we found 15 of the top 25 amino acid sites, ranked by relative importance to the model (all ≥0.40), were spectral tuning sites previously characterized by mutagenesis and heterologous expression (Supplementary Material 9 (S9)). For example, the especially well-characterized position 308 (p308), known for its role in tuning LWS opsins and considered 1 of the 5 key sites in characterizing LWS opsins under the “five-site rule” [81], had the highest relative importance value of 1.0 when using the full WT model, indicating the amino acid identity at p308 is especially important for predicting λ_max_. In another example, the full WT model highlighted p181, a phylogenetically conserved counterion in the retinal-opsin Schiff base interaction for all nonvertebrate opsins [82, 83]. Additionally, the transition from E to H at p181 (E181H) is a characteristic of the red-shifted vertebrate LWS opsins [35, 83], easily visualized in Fig. 4C. When predicting λ_max_ of bovine rhodopsin with mutation E181H, the WT model predicted a red shift compared to wild type, as observed with the natural evolution of the LWS opsin lineage. The WT SWS/UVS model similarly highlighted p113, a site functionally characterized as the counterion in the retinal-opsin Schiff base interaction for all vertebrate opsins [35, 83] and as a known spectral tuning site in SWS/UVS opsins [84]. Moreover, even the WT rod model, trained on a mere 157 sequences, identified p292 (Supplementary Material 9 (S9)), another well-characterized and conserved spectral tuning site for vertebrate rhodopsins [85–87], as the site with highest relative importance to its predictions of rhodopsin λ_max_. These spectral tuning sites are not simply conserved sites, as there is little to no correlation between amino acid sites important to model predictions (importance scores) and their relative *Shannon entropy* [88, 89] scores (*R*^2^ = 0.001). This is somewhat expected as *deepBreaks* drops all conserved (“zero-entropy”) sites during preprocessing, because a site with no variation provides no important information about the effects of variation on the resulting phenotype. In addition, we predict any correlation between site conservation and model importance would be for sites that are moderately conserved and in close proximity to opsin–chromophore binding site (position 296) or binding pocket [41, 42, 90].

## Discussion

To better understand methods to connect genes and their functions, we initiate *VPOD*, a database of opsin genes and corresponding spectral sensitivity phenotypes. Here, we used *VPOD_1.0* to examine the ability of ML models to predict functions of opsin genes, predict intragenic epistasis, and identify amino acid sites critical for functional changes. In all cases, ML shows promise, especially when given enough training data.

### The important relationship between data availability and predictive power

The predictive power of λ_max_ is often high when using ML for opsins, and it improves with a greater amount and variety of data, albeit with diminishing returns. In particular, the number of opsin genes, their genetic diversity, and the relationship between genetic and phenotypic differences are all critical in determining predictive power. Particularly illustrative of these ideas are our analyses with and without experimentally mutated opsins. Even though we might conceive of all wild-type data as natural mutants chosen by evolution, experimentally induced mutations are particularly important by often changing just 1 amino acid that drastically changes phenotype. As such, we found that including mutant data usually improved predictive power, and conversely, predicting some phenotypes from laboratory mutagenesis was sometimes difficult without including other mutant data in model training (Supplementary Material 11 (S11)). However, relying on published mutant data alone is not optimal because it is derived from a nonrandom subset of species because people continue to work in established systems. Nevertheless, the genotype–phenotype landscape may be sampled well enough using high numbers of only wild-type genes, as evidenced by the small difference in performance when adding mutant data to the wild-type subset of well-sampled vertebrate opsins (Table 1). In contrast, adding mutant data to the sparsely sampled invertebrate opsins made a big difference. For invertebrate opsins, using only wild-type data (ignoring all mutants) led to some very inaccurate predictions, especially of large phenotypic shifts caused by experimental mutagenesis (Fig. 3), indicating the genotype–phenotype space is still undersampled for invertebrates. This is expected since ML learns from patterns in the underlying dataset, making predictions of distantly related opsins from those in *VPOD* more unreliable. We acknowledge this as a significant drawback for the ML approach, especially in systems or taxonomic groups lacking sufficient or reliable data. Thus, given this currently limited dataset, we do not put high confidence in the λ_max_ estimates of either wild-type or mutant invertebrate (rhabdomeric) opsins. Therefore, targeting invertebrate opsins should be a high priority for new additions to *VPOD*.

A large diversity of training data is also critical for reliably predicting intragenic epistasis—the nonadditive effects on a phenotype of interactions between 2 or more mutations within a gene—which is common [10, 41, 43, 44, 92, 93] and an obstacle to connecting genotypes and phenotypes [41, 94–96]. Our most complete datasets (whole dataset and vertebrate dataset) identified known cases of intragenic epistasis, but our models trained without experimental mutagenesis data did not. Moreover, ML demonstrates some capacity to predict the epistatic interactions between mutations, even when only provided with the single mutation components—as is evidenced by our WDS-minusepi dataset test (Supplementary Material 12 (S12)). Similarly to the overall predictive power of λ_max_ above, predicting epistasis probably requires sufficient variation at interacting sites, which seems especially enhanced by experimentally mutated genes.

Variation in the availability of genotype–phenotype data for training impacts not only the predictive power of phenotype but also the converse: the ability to predict amino acid sites that change λ_max_. Several models, including those trained with the WDS, vertebrate, and WT data, were able to successfully predict previously characterized spectral tuning sites. This is less surprising for models trained with WDS and vertebrate datasets due to the prevalence of data, even including mutants in the training data from experiments that specifically targeted sites thought by researchers to be functionally informative. Yet even without any targeted mutational data, 3 model variants using only wild-type data predicted experimentally well-characterized spectral tuning/functional sites, including sites important to the stability of the opsin–chromophore interaction (P181 and P113). This demonstrates the strong potential for ML models to identify amino acid sites that govern phenotype, leading to predictions of candidate spectral tuning sites, which can be confirmed with mutagenesis experiments [38, 86] if not done so already.

### ML algorithm type contributes to the predictive power of ML models

While probably not as important as the training data used, the ML algorithm itself also impacts predictive power. All 5 of the best-performing ML algorithms (GBR, BR, LGBM, RF, and XGB) are variants of the decision tree model architecture (Supplementary Material 13 (S13)), and 3 of 5, including GBR, LGBM, and XGB, are “gradient boosted” decision tree–based ML algorithms. The gradient boosted algorithms all share the same general principles of gradient boosting [76, 97], including the use of ensembles of “weak learners,” usually decision trees, which work sequentially and “gradient descent” when minimizing a loss function, to improve ML model performance. While LGBM generally performed best for predicting phenotype, it was not as effective in predicting the epistatic effects of mutations, where GBR and XGB showed the highest performance. This suggests that while LGBM excels in general phenotype prediction, the details of GBR and XGB may be better suited for epistasis prediction. The difference likely arises from the unique aspects of each algorithm’s model training and settings of hyperparameters. XGB and LGBM differ from GBR by the addition of a regularization term to the objective function and in the process of ensemble tree construction during model training: GBR and XGB use level-based tree fitting while LGBM uses leaf-based tree fitting. One consequence of leaf-based tree construction is that due to its faster convergence/training time, it can create complex trees that are more prone to overfitting, thereby “learning” patterns that may not exist as it constructs trees on a “best-first basis” with a fixed number of n-terminal nodes [63, 79]. This creates a model that often performs well on training data but may overgeneralize, missing finer grained collinearities and interdependencies, which would be important for predicting epistasis. As such, our models might be improved by fine-tuning hyperparameters (e.g., learning rate, max-depth, and number of estimators), and the choice of which model to use will depend on the end goals of the analysis.

### The assumptions of our method and limitations of ML extrapolation

Understanding the limitations and assumptions inherent in predictive modeling is vital for accurately interpreting animal color sensitivity from opsin sequences, especially considering the impact of various factors on sensitivity beyond the opsin itself across multiple levels of biological organization. At the cell level, we assume that λ_max_ measured in cell culture (e.g., HEK293, COS cells) is the same as in living photoreceptor cells. We also assume the photopigment uses 11-*cis*-retinal, as all heterologously expressed opsins in *VPOD* were reconstituted using this chromophore. However, this assumption is violated in some organisms because they use 13-*cis*-retinal as the *in vivo* chromophore [23, 98, 99], which is associated with a red shift in λ_max_ [35, 98]. At the organ level, filters such as oil droplets in bird eyes [100–103], pigments in butterfly eyes [104], or a combination of transmissive filter and narrow band reflector in mantis shrimp larval eyes [105] each may selectively influence light reaching photoreceptor cells and therefore animal color sensitivity. Finally, organismal responses to light involve neural processes, so even if an organism possesses the physiological ability to detect certain wavelengths, it still may not have a use for that ability. Similar considerations for all these assumptions will apply when using ML to infer other functions from other genes. In fact, many genes are more susceptible than opsins (but see [106] showing the pressure of ocean depth may slightly affect λ_max_ phenotypes) to changes in pH, temperature, and other environmental factors [107], such that databases compiling these gene functions should also record these parameters for use in training ML models.

Perhaps the most important caveat of using ML models to accurately predict phenotype or functional sites is that we assume there is a genotype–phenotype association that we can fit to a function and that our models were trained using ample data to capture these associations. Based on the nonlinear fit between size of training dataset and model performance, we estimate that including about 200 sequences (and corresponding λ_max_) from a taxonomically and phenotypically diverse range still provides improvements to model performance. Above 200 sequences, there is still improvement, but at a diminishing rate consistent with a reciprocal model (Supplementary Material 2 (S2), Supplementary Material 3 (S3). That said, we encourage caution when extrapolating these results to predict model performance on training data size alone as the equations we used do not account directly for taxonomic, genetic, or phenotypic diversity. When using ML for predicting functionally important sites, the addition of experimental mutants to training data that cause large phenotypic changes could heavily bias which sites are selected as “most important” and potentially mask the importance of other sites. Here again, providing a diverse set of genotype–phenotype data should allow for the discovery of new functional sites, even when including known mutants in the training data with large phenotypic effects. Additionally, providing a large number of mutations from a limited breadth of taxa can bias model predictions as not all mutations will have the same effect on different sequences, especially if they are genetically distant. This makes it all the more important to consider the level of genetic diversity used to train a model when extrapolating to find potentially important functional sites (i.e., if identifying tuning sites for rhodopsins, then using a dataset of only rhodopsins would likely be the best approach, but if data are sparse or if looking for sites that may largely impact spectral tuning across opsin subfamilies, a genetically and phenotypically broad dataset may be better).

## Conclusion

Using opsin sequence data with *deepBreaks*, we were able to train regression-based ML models to reliably predict λ_max_, often accounting for nonadditive effects of mutations on function (intragenic-epistasis) and identifying amino acid sites critical for function. We expect future work will improve these already promising results even further through at least 2 general directions. First, adding more data to *VPOD* will improve results, especially adding invertebrate (rhabdomeric opsins) data, as technical knowledge improves for expressing these genes [34]. In addition, phenotypic data—besides the *in vitro* heterologous expression targeted here—is expansive, including λ_max_ measurements from microspectrophotometry and electroretinograms, but will take considerable effort to link these phenotypes to specific opsin genes. Second, our models can be improved to take advantage of more information. One important addition should be inclusion of physicochemical properties of the amino acids [108], as implemented with success on a small scale of only 26 amino acid positions of microbial opsins to predict red-shifted phenotypes for optogenetics [109]. Additionally, information on protein structure could be particularly important, such as the distance of an amino acid from the binding pocket of the chromophore [40]. While there are only a few solved crystal structures for opsins [110, 111] to provide such data, indirect techniques like homology modeling [112] or neural network–based structural prediction [113] might be usable. Other information about opsins could also be predictive, such as which G-protein the opsin signals to, allowing prediction of which amino acids dictate G-protein specificity. Opsin kinetics [e.g., 114], or even the habitat depth at which the animal lives in the ocean, which not only influences light environment but also alters which amino acids are used in opsins [115], could improve predictive power of the ML models. Finally, we once again caution against treating predictions of λ_max_ uncritically, because the quantity and quality of genotype–phenotype data used to train a model—including the taxonomic, genetic, and phenotypic diversity—is integral to the reliability of a model’s predictions. Thus, ML models like those used here can be considered tools to make predictions based on summaries of existing knowledge, thereby complementing traditional experimental methods.

### Potential implications

Given the high performance demonstrated in this article, current models are already robust enough to allow several applications. First, predicting λ_max_ will often be useful, especially for vertebrate opsins. For example, ML could provide an estimate of λ_max_ in a hogfish, whose skin expresses an opsin with unknown absorption and where λ_max_ has implications for a conceptual model of chromatophore expansion [116]. Second, estimates of λ_max_ from opsin sequences formed part of an argument that changes in gene expression, not sequence, adapted Amazon fishes to local light environments [117]. On broader taxonomic scales, predictions of λ_max_ from opsin sequences could expand studies of adaptation, molecular evolution, and constraint in comparison to light environments [118]. Another application could be protein design for optogenetics—the use of genetic light sensors to induce and study expression or response pathways [119–121]—including those associated with embryogenesis [122,123], stress and depression [124–126], or neuronal diseases [127, 128]. Finally, our models could be used to simulate molecular evolution under a realistic genotype–phenotype landscape. One shortcoming presently for such simulations is that our models are not trained with nonfunctional opsins, so even nonfunctional genes would be predicted to have functional λ_max_ values. A solution could be to add large-scale mutagenesis data to the training set, such as that from deep mutational scanning [129], although the authors indicated the method is only in a proof-of-concept stage, such that the results are too noisy to be useful for model training. As the *VPOD* database expands, there will be many applications for ML, and similar techniques can also be applied to other gene families such as luciferases [16,130,131].

## Availability of Supporting Source Code and Requirements

**Project name**: The Visual Physiology Opsin Database (VPOD).

**Project homepage:** https://github.com/VisualPhysiologyDB/visual-physiology-opsin-db [56].

**License:** GNU General Public License (GPL)—Version 3, 29 June 2007.

**RRID:** SCR_025668.

**Operating system(s)**: Windows, MacOS, and Linux.

**Programming language:** Python, R.

**Other requirements**: Conda 4.9.2, deepBreaks 1.1.2, GBlocks 0.91b, MAFFT 7.520-1, MUSCLE 3.8.31, mySQL workbench 8.0.36, Python 3.9, RStudio 2023.06.2+562.

**Docker image of the latest version of the deepBreaks:** [132].

The Docker image provided above includes a summary of required package libraries and instructions on how to use it. Along with our existing online materials with tools used, *deepBreaks*, we also have a Jupyter notebook, instructions for Conda installation, and Code Ocean (RRID:SCR_015532) capsule [133], for deepBreaks.

These resources should help practitioners using the main ML program we used, deepBreaks, described elsewhere, use the VPOD database for Opsin applications.

## Supplementary Material

giae073_GIGA-D-24-00053_Original_Submission

giae073_GIGA-D-24-00053_Revision_1

giae073_GIGA-D-24-00053_Revision_2

giae073_GIGA-D-24-00053_Revision_3

giae073_Response_to_Reviewer_Comments_Original_Submission

giae073_Response_to_Reviewer_Comments_Revision_1

giae073_Response_to_Reviewer_Comments_Revision_2

giae073_Reviewer_1_Report_Original_Submission Robert Lucas -- 3/28/2024 Reviewed

giae073_Reviewer_2_Report_Original_Submission Nikolai Hecker -- 4/4/2024 Reviewed

giae073_Reviewer_2_Report_Revision_1 Nikolai Hecker -- 7/9/2024 Reviewed

giae073_Reviewer_2_Report_Revision_2 Nikolai Hecker -- 7/9/2024 Reviewed

giae073_Reviewer_3_Report_Original_Submission Fabio Cortesi -- 4/14/2024 Reviewed

giae073_Supplemental_File

## Data Availability

The dataset(s) supporting the results and all other code used in this article are available in the “*Visual Physiology Opsin Database*” GitHub repository archived in Zenodo [134] and [135]. A DOME-ML (Data, Optimisation, Model, and Evaluation in Machine Learning) annotation [136] supporting the current study is available for scruitiny [137].

## Additional Files

**Supplementary Material 1 (S1)**. Performance metrics across opsin subsets and top performing models for *VPOD_1.1*.

**Supplementary Material 2 (S2)**. Tracking model performance vs. number of sequences in training data.

**Supplementary Material 3 (S3)**. Three functions fitted to visualize the relationship between training data size (number of genotypes and corresponding phenotypes) vs. model performance (*R*^2^) based on results from the vertebrate subset of data. The Akaike information criterion (AIC) is a measure used for model selection when comparing different statistical models, accounting for both the goodness of fit of the model and the simplicity of the model (the number of parameters used). The goal is to find a balance between a model’s ability to explain the data and its complexity, preventing overfitting.

**Supplementary Material 4 (S4)**. Comparing ML predictions on invertebrate and vertebrate UVS/SWS opsin MSP data.

**Supplementary Material 5 (S5)**. Graph of WT model predictions for 30 unseen invertebrate opsins, *R*^2^ = 0.887, MAE = 17.5 nm, MAPE = 4.05. All the “known” λ_max_ values are from physiological measures, including MSP or ERG measurements (instead of purified heterologously expressed opsins), and are linked to a particular opsin sequence by *in situ* hybridization. The light gray bar surrounding the trend line represents a 95% confidence interval.

**Supplementary Material 6 (S6)**. Graph of vertebrate model predictions for unseen WT-UVS/SWS data, *n* = 25, *R*^2^ = 0.914, MAE = 7.89 nm, MAPE = 1.90. All sequences were randomly selected from the UVS/SWS model under the condition that they were WT opsins. The light gray bar surrounding the trend line represents a 95% confidence interval.

**Supplementary Material 7 (S7)**. Comparing performances of ML predictions and phylogenetic imputation on a subsample of opsin data.

**Supplementary Material 8 (S8)**. Results for epistasis test on the WDS, vertebrate, WT, and rod models.

**Supplementary Material 9 (S9)**. Functionally characterized spectral tuning sites predicted by the WT models.

**Supplementary Material S10 (S10)**. Phylogenetic gene tree of all wild-type opsins (*n* = 362), including ancestral constructs (branch lengths = 0), constructed from the VPOD_wt_het_1.1 dataset. In this tree, we have annotated the major opsin groups (c-opsins, r-opsins, and some tetraopsins), then further annotated the c-opsin families (LWS, SWS1, SWS2, Rh1, and Rh2). We have also assigned taxonomic annotations by class, which are color-coded and provided by the key.

**Supplementary Material S11 (S11)**. Including data from experimentally mutated opsin sequences reduces errors in predicting λ_max_. (A) Distributions of errors from predicting λ_max_ of experimentally mutated opsin sequences. Blue are prediction errors when using the WT model, which lacks experimentally mutated sequences (root mean square error [RMSE] = 17.6 nm). Orange are prediction errors when using the WDS model, which includes experimentally mutated sequences (RMSE = 12.6 nm). (B) Data from experimental mutants significantly improves predictions of λ_max_ when using a model trained with experimental mutants (WDS) compared to a model without (WT) experimental mutant data, rejecting the null hypotheses of no difference between prediction errors based on different models. At top is the distribution of differences between predictions with and without experimental mutants in the training data for large effect mutations (>10 nm). At bottom is the same for all mutations. We plot differences of absolute error instead of squared error in B for easier visualization, although *P* values were calculated using distributions of squared errors. Additionally, plotting raw differences allows seeing most values are below zero, meaning predictions with WDS (which has experimental mutants) have less error than those without experimental data from mutants (WT).

**Supplementary Material 12 (S12)**. Including data from experimentally mutated opsin sequences reduces errors in predicting epistatic effects. (A) We analyzed opsins with multiple mutations whose known effect on λ_max_ phenotype were nonadditive (epistasis). In purple, we plot the difference (absolute error in nm) between known λ_max_ phenotypes with epistasis, compared to λ_max_ phenotypes ignoring epistasis by assuming individual mutations are not additive, which we call epistasis-free additive mutation values (EAMVs). Here root mean square error (RMSE) = 29.8 nm. In blue, we plot errors when predicting epistatic phenotypes using a model trained without opsins containing experimentally generated mutations (WT), which lead to RMSE = 12.4 nm. In orange, we plot errors when predicting epistatic phenotypes using a model trained with opsins containing experimentally generated mutations but excluding those whose mutational effects are nonadditive (WDS-minusepi), which lead to RMSE = 12.4. (B) Our tests of the null hypotheses of no underlying differences between the distribution of squared error for predictions of λ_max_ for the 111 “epistatic opsins” were rejected with Wilcoxon signed-rank tests after Bonferroni correction by the WDS-minusepi model versus WT model (*P* = 1.24e-06); WDS-minusepi model versus EAV (*P* = 2.56e-09), but not rejected for the WT model versus EAV (*P* = 0.086). The large differences in RMSE and the results of the statistical comparisons strongly support the idea that the inclusion of even single mutants greatly reduces the error of ML models when predicting epistatic interactions between mutations and that this error is significantly less than the error we would observe if our models simply treated mutations as additive. Conversely, the insignificant difference between WT predictions and EAMV indicates there is not enough information about epistatic interactions in wild-type (which excludes artificially mutated opsins) data alone to accurately predict intragenic epistasis. As with S11, we plot differences of absolute error instead of squared error in B for easier visualization but use squared error for statistical comparison.

**Supplementary Material 13 (S13)**. Ranked ML algorithm performances.

## Abbreviations

Adaboost: adaptive boosting; AIC: Akaike information criterion; COS1: monkey kidney cell line; CV: cross-validation; EAMV: epistasis-free additive mutation value; ERG: electroretinogram; GBR: gradient boosting regressor; GPCR: G-protein coupled receptor; HEK293: human embryonic kidney cell line; ISH: *in situ* hybridization; KDE: kernel density estimate; LGBM: light gradient boosting machine; LWS: long-wave sensitive; MAE: mean absolute error; MAPE: mean absolute percentage error; ML: machine learning; MSE: mean squared error; MSP: microspectrophotometry; MWS: medium wavelength-sensitive; NCBI: National Center for Biotechnology Information; RMSE: root mean square error; SWS: short-wave sensitive; T1: type 1 {microbial opsins}; TMD: transmembrane domain; USS: ultraviolet and short-wave sensitive; UVS: ultraviolet sensitive; VPOD: Visual Physiology Opsin Database; WAG: Whelan and Goldman substitution model; WDS: whole dataset; WT: wild-type; XGB: extreme gradient boosting; λ_max_: lambda max/wavelength of light with maximal absorbance.
