# Supplementary material for: Discovering genotype–phenotype relationships with machine learning and the Visual Physiology Opsin Database (VPOD)
Source: Gigascience. 2024 Oct 26;13:giae073. doi: 10.1093/gigascience/giae073 (PMC11512451; doi:10.1093/gigascience/giae073)
Supplement: giae073_GIGA-D-24-00053_Original_Submission [file giae073_giga-d-24-00053_original_submission.pdf]

GigaScience

Discovering genotype-phenotype relationships with machine learning and the Visual Physiology Opsin Database (VPOD)

--Manuscript Draft--

**Manuscript Number:**	GIGA-D-24-00053
**Full Title:**	Discovering genotype-phenotype relationships with machine learning and the Visual Physiology Opsin Database (VPOD)
**Article Type:**	Research
**Funding Information:**	Directorate for Biological Sciences (DEB-2153773) — Prof Todd Oakley<br>Directorate for Biological Sciences (IOS-1754770) — Prof Todd Oakley<br>Directorate for Biological Sciences (DEB-2109688) — Dr. Keith A. Crandall

Abstract:

Background

Predicting phenotypes from genetic variation is foundational for fields as diverse as bioengineering and global change biology, highlighting the importance of efficient methods to predict gene functions. Linking genetic changes to phenotypic changes has been a goal of decades of experimental work, especially for some model gene families including light-sensitive opsin proteins. Opsins can be expressed in vitro to measure light absorption parameters, including λmax - the wavelength of maximum absorbance - which strongly affects organismal phenotypes like color vision. Despite extensive research on opsins, the data remain dispersed, uncompiled, and often challenging to access, thereby precluding systematic and comprehensive analyses of the intricate relationships between genotype and phenotype.

Results

Here, we report a newly compiled database of all heterologously expressed opsin genes with λmax phenotypes called the Visual Physiology Opsin Database (VPOD). VPOD_1.0 contains 864 unique opsin genotypes and corresponding λmax phenotypes collected across all animals from 73 separate publications. We use VPOD data and deepBreaks to show regression-based machine learning (ML) models often reliably predict λmax, account for non-additive effects of mutations on function, and identify functionally critical amino acid sites.

Conclusion

The ability to reliably predict functions from gene sequences alone using ML will allow robust exploration of molecular-evolutionary patterns governing phenotype, will inform functional and evolutionary connections to an organism’s ecological niche, and may be used more broadly for de-novo protein design. Together, our database, phenotype predictions, and model comparisons lay the groundwork for future research applicable to families of genes with quantifiable and comparable phenotypes.

**Corresponding Author:**	Todd Oakley, PhD<br>UCSB: University of California Santa Barbara<br>Santa Barbara, CA UNITED STATES
**Corresponding Author Secondary Information:**	
**Corresponding Author's Institution:**	UCSB: University of California Santa Barbara
**Corresponding Author's Secondary Institution:**	
**First Author:**	Todd Oakley, PhD
**First Author Secondary Information:**	

Powered by Editorial Manager® and ProduXion Manager® from Aries Systems Corporation

**Order of Authors:**	Todd Oakley, PhD
	Seth A. Frazer, BS
	Mahdi Baghbanzadeh, BS
	Ali Rahnavard, PhD
	Keith A. Crandall, PhD
**Order of Authors Secondary Information:**	
**Additional Information:**	
**Question**	**Response**
Are you submitting this manuscript to a special series or article collection?	No
**Experimental design and statistics**<br><br>Full details of the experimental design and statistical methods used should be given in the Methods section, as detailed in our Minimum Standards Reporting Checklist. Information essential to interpreting the data presented should be made available in the figure legends.<br><br>Have you included all the information requested in your manuscript?	Yes
**Resources**<br><br>A description of all resources used, including antibodies, cell lines, animals and software tools, with enough information to allow them to be uniquely identified, should be included in the Methods section. Authors are strongly encouraged to cite Research Resource Identifiers (RRIDs) for antibodies, model organisms and tools, where possible.<br><br>Have you included the information requested as detailed in our Minimum Standards Reporting Checklist?	Yes
**Availability of data and materials**<br><br>All datasets and code on which the conclusions of the paper rely must be	Yes

Powered by Editorial Manager® and ProduXion Manager® from Aries Systems Corporation

either included in your submission or deposited in publicly available repositories (where available and ethically appropriate), referencing such data using a unique identifier in the references and in the “Availability of Data and Materials” section of your manuscript.<br><br>Have you have met the above requirement as detailed in our Minimum Standards Reporting Checklist?	

Manuscript adding OA statements with supplemental
Click here to access/download;Manuscript;gigasci_ms_frazer_oakley_2024

Discovering genotype-phenotype relationships with machine learning and the Visual Physiology Opsin Database (VPOD)

Seth A. Frazer[1], Mahdi Baghbanzadeh[2], Ali Rahnavard[2], Keith A. Crandall[2,3], Todd H. Oakley[1,*]

Affiliations:
[1]Ecology, Evolution, and Marine Biology, University of California, Santa Barbara, California 93106

[2]Computational Biology Institute, Department of Biostatistics and Bioinformatics, Milken Institute School of Public Health, The George Washington University, Washington, DC 20052
[3]Department of Invertebrate Zoology, National Museum of Natural History, Smithsonian Institution, Washington, DC 20012

ORCID and Email:
Seth A. Frazer
sethfrazer@ucsb.edu
https://orcid.org/0000-0002-3800-212X

Mahdi Baghbanzadeh
mbagh@gwu.edu
https://orcid.org/0000-0002-1878-2691

Keith A. Crandall
kcrandall@gwu.edu
https://orcid.org/0000-0002-0836-3389

Ali Rahnavard
rahnavard@gwu.edu
https://orcid.org/0000-0002-9710-0248

Todd H. Oakley
oakley@ucsb.edu
https://orcid.org/0000-0002-4478-915X
*Correspondence to oakley@ucsb.edu

Abstract

Background: Predicting phenotypes from genetic variation is foundational for fields as diverse as bioengineering and global change biology, highlighting the importance of efficient methods to predict gene functions. Linking genetic changes to phenotypic changes has been a goal of decades of experimental work, especially for some model gene families including light-sensitive opsin proteins. Opsins can be expressed in vitro to measure light absorption parameters, including λ_{max} - the wavelength of maximum absorbance - which strongly affects organismal phenotypes like color vision. Despite extensive research on opsins, the data remain dispersed, uncompiled, and often challenging to access, thereby precluding systematic and comprehensive analyses of the intricate relationships between genotype and phenotype.

Results: Here, we report a newly compiled database of all heterologously expressed opsin genes with λ_{max} phenotypes called the Visual Physiology Opsin Database (*VPOD*). *VPOD_1.0* contains 864 unique opsin genotypes and corresponding λ_{max} phenotypes collected across all animals from 73 separate publications. We use *VPOD* data and *deepBreaks* to show regression-based machine learning (ML) models often reliably predict λ_{max}, account for non-additive effects of mutations on function, and identify functionally critical amino acid sites.

Conclusion: The ability to reliably predict functions from gene sequences alone using ML will allow robust exploration of molecular-evolutionary patterns governing phenotype, will inform functional and evolutionary connections to an organism's ecological niche, and may be used more broadly for *de-novo* protein design. Together, our database, phenotype predictions, and model comparisons lay the groundwork for future research applicable to families of genes with quantifiable and comparable phenotypes.

Key words: Machine learning; Regression, Compiled database; Genotype-phenotype relationships; Predicting phenotypes; Spectral sensitivity; Color-vision; Opsins; Imputation

Key Points

- We introduce the Visual Physiology Opsin Database (VPOD_1.0), which includes 864 unique animal opsin genotypes and corresponding λ_{max} phenotypes from 73 separate publications.

- We demonstrate that regression-based ML models can reliably predict λmax from gene sequence alone, predict non-additive effects of mutations on function, and identify functionally critical amino acid sites.

- We provide an approach that lays the groundwork for future robust exploration of molecular-evolutionary patterns governing phenotype, with potential broader applications to any family of genes with quantifiable and comparable phenotypes.

Introduction

Although critical to progress in drug and vaccine design [1–3], responses to climate change [4–8], and bioengineering [4,9–11], accurately predicting gene function from sequences remains a significant challenge. While there are many ways to elucidate genotype-phenotype relationships experimentally, including deep mutational scanning, and in-vitro heterologous expression with phenotyping, these techniques are often tedious and cost-prohibitive, especially when applied to broad comparative studies of gene families. In addition, accurately predicting the phenotype of a protein using computational methods alone is challenging because of data gaps and the sheer complexity of possible relationships between genes and phenotypes, including epistasis and the non-additive effects of different mutations. Machine learning (ML) is gaining traction for its potential broad biological applications, accessibility, and faster speeds, especially in biological contexts where phenotype data are abundant and quantifiable. Here, classical regression and classification algorithms are sometimes used to train models for phenotype predictions using genotype-phenotype data [12,13], while deep learning models can be used to integrate heterogeneous multi-layered omics and environmental data for establishing higher dimensional genotype-phenotype connections [14,15] or *de-novo* protein design [16]. In broader biological contexts, ML models often inform laboratory experiments to predict directional evolution of diseases and their variants [17–19] or to automate image sorting and animal identification from camera trap data [20–22]. In all cases, ML models can be iteratively improved as data gradually accumulates, making them a worthwhile long-term investment.

Decades of laboratory work have led to significant progress in understanding the genetic basis of phenotypic changes for model gene families such as opsins. Opsins are a family of G-protein Coupled Receptors (GPCR) which bind to a chromophore and absorb photons. Opsins have crucial roles in many organismal functions, including circadian rhythms, phototaxis, and image-forming color vision. A critical opsin phenotype is spectral sensitivity - the range of wavelengths to which a gene or organism is sensitive. The main parameter of opsin spectral sensitivity is λ_{max}, the wavelength of light (in nm) with maximal absorbance [23]. Common methods of characterizing spectral sensitivities and λ_{max} include organ-level electroretinograms (ERG) [24–26], cell-level microspectrophotometry (MSP) [27–31], and purification of heterologously expressed opsins followed by spectrophotometry. Different opsins are tuned by changes in amino acid sequences to respond to different wavelengths of light, and many previous studies have expressed experimentally mutated opsins and measured spectral sensitivities to establish genotype-phenotype connections [32–36]. Although other factors sometimes affect spectral responsiveness, opsins provide a rare case where an intrinsic molecular function extends rather directly to organismal phenotypes, especially those involving color sensitivity. Despite opsins being a well-studied system with an extensive backlog of published literature, previous authors expressed doubts that sequence data alone can provide reliable computational predictions of λ_{max} phenotypes [37–40]. Furthermore, only the non-animal, microbial, or Type-1 (T1) opsins [41,42] have been cataloged and used to examine genotype-phenotype predictive power of ML models. The extensive data on animal opsin genotype-phenotype associations remains

disorganized, decentralized, often in non-computer readable formats in older literature, and under-analyzed computationally.

Here, we report a genotype-phenotype database for animal opsins called the Visual Physiology Opsin Database (*VPOD*). We used standard literature searches to compile all heterologously expressed animal opsin genes with spectral sensitivity measurements. We used this newly compiled and harmonized database to evaluate ML methods for connecting genotypes and phenotypes. We created eleven subsets of the overall database to examine factors that impact the reliability and performance of ML models and compared ML predictions to phylogenetic imputation [43,44]. We also examined whether ML can predict intragenic epistasis, and we predicted amino acid sites particularly important for changing λ_{max}. Using our database of 864 unique opsin sequences and corresponding λ_{max} values, we show ML models trained on opsin data accurately predict the λ_{max} of opsins from genetic data alone [highest R^2 =0.968 with a lowest mean absolute error (MAE) of 6.56 nm], especially when ample and diverse training data are available. ML also predicts some known effects of epistatic mutations on λ_{max}. Finally, ML models identify several sites that cause shifts in λ_{max} (e.g., 'spectral tuning sites') and sites known to be structurally important, even in the absence of mutant data in training. These results support the use of ML as a reliable and efficient predictor of λ_{max} for previously uncharacterized opsins, as a tool for identifying candidate spectral tuning sites and epistatic interactions, and as a more general method for linking gene sequences and phenotypes.

Methods

Compiling a genotype-phenotype database for animal opsins

We collected λ_{max} data for opsins using typical literature review/search methods, documented in the '*litsearch'* table of the *VPOD* database to record search engine, keywords, and date of access. We cataloged all usable papers with λ_{max} data in the '*references'* table of *VPOD*, recording DOI and a key to link to the search that found the paper. We documented the details of heterologous expression experiments in the '*heterologous'* table, including species, GenBank accession number for the sequence, mutation(s) (if applicable) using a machine-readable notation, λ_{max}, cell type for expression (e.g., HEK293, COS1, etc.), protein purification method, type of spectrum (e.g., dark or difference spectrum), and a key to link to the corresponding literature source. We input opsin genetic data in an *'opsins'* table, recording opsin gene family names (e.g., long-wave sensitive=LWS, short-wave sensitive=SWS1, etc.). We also included specific '*gene names'* (where applicable), phylum, class, species information, accession number, DNA sequence, amino acid sequence, and the database from which sequences were retrieved (e.g., NCBI). We recreated all mutant and chimeric (e.g. one or more transmembrane domains of the mutant copied from a different sequence to replace the original) opsin sequences based on literature descriptions using a pair of Python scripts (*mutagenesis.py* and *chimeras.py*) available on our GitHub (https://github.com/VisualPhysiologyDB/visual-physiology-opsin-db). We added all heterologously expressed opsins from the literature to the current version of *VPOD*, which we call *VPOD_1.0*. We refer to heterologous data as a data subset named *VPOD_het_1.0*,

which will allow for future additions to the database to link specific opsin sequences to λ_{max} values established with microspectrophotometry or other methods.

Training ML models with *deepBreaks*

We performed all data pre-processing, including data extraction, sequence alignments, and formatting, in the Jupyter notebooks '*opsin_model_wf.ipynb*', available on GitHub. We used two multiple sequence alignment methods, MAFFT [45] and MUSCLE [46], and a version of both alignments with a Gblocks [47] refinement (for a total of four alignments), all set to their default parameters to begin to test the sensitivity of model performance to different alignments. We then trained various ML models employing a custom version of *deepBreaks* [48], an ML tool designed for exploring genotype-phenotype associations. *deepBreaks* takes aligned genotype data (DNA, RNA, Amino Acid) and some measure(s) of corresponding continuous or categorical phenotype data as input to train ML models. deepBreaks uses one-hot encoding to convert amino acid sequences into numerical values. One consequence of this encoding is any amino acids at a given position in the alignment, which are not present at that position in any training data, will be treated equivalently as unseen. For example, cases of only A and V at a highly conserved site in the training set that are presented with a sequence with T at that site will be considered as no A and no V. The models cannot distinguish the input whether it's T or other unseen amino acids at that site. The results produced by *deepBreaks* encompass a compilation of 12 regression ML models [48], showcasing ten metrics of cross-validation performance (ranked by R^2) and a report derived from the top-performing models, which ranks amino acid positions by their relative importance to the model (from 0.0-1.0, with 1.0 being a site with the highest relative importance) for the phenotype in question (λ_{max}). We evaluated the performance of algorithms based on their relative ranks to look for patterns in which algorithms performed better for different data subsets and approaches. *deepBreaks* also produces a set of distribution box plots (default is 100) to visualize phenotypes (λ_{max}) associated with a particular amino acid identity at a site of interest, ordered alphabetically.

Understanding model performance using different subsets of the database

We created eleven data subsets with varying levels of taxonomic and gene family inclusivity (Table 1) to test which factors most impact the reliability/performance of ML methods. We used naming conventions that include versioning to improve reproducibility and reliability of individual datasets and models. For example, one subset combines ultraviolet and SWS opsins, which we named *VPOD_uss_het_1.0.* Our convention is to name the subset (in this case USS = 'Ultraviolet and Short-wave Sensitive' opsins); name the source of phenotype data (heterologous = het), and record the version number of the dataset (1.0). We also created subsets for medium- and long-wave sensitive opsins (*VPOD_mls_het_1.0)* and all rod (Rh1) and rod-like (Rh2) opsins (*VPOD_rod_het_1.0*). Other subsets use species taxonomy, one for vertebrates (*VPOD_vert_het_1.0)* and another for invertebrates (*VPOD_inv_het_1.0)*. For taxonomic subsets, we considered all sequences from phylum Chordata as 'vertebrates' and the rest as

'invertebrates'. Another subset excludes all mutant opsin sequences, called 'wild-types' (*VPOD_wt_het_1.0)*. A final named subset is the whole data set (*VPOD_wds_het_1.0)*.

Using various subsets of data, we performed a number of experiments to better understand the performance of ML models in predicting λ_{max}. First, to better understand how training data relate to model performance, R^2 , and training data size, we gradually increased the size of training datasets, using the WDS, Vertebrate, WT, and Rod subsets separately, by adding between 15-50 randomly selected sequences at a time, repeating the process three times per data split (Table S1). We then analyzed the fit between the size of training data sets (x-axis) and model performance (y-axis), comparing six non-linear models with AIC to find the model that best explains the observed variation (Figure S2). Second, to understand if ML could predict known phenotypic changes due to experimental mutations, we queried the top performing WT model (which lacks data from artificially mutated sequences) using all experimentally mutated opsins to predict their phenotypes. We plotted these results using *matplotlib [49]* to visualize characteristics of poorly predicted outliers (e.g., taxonomic bias or sensitivity to mutations which caused large shifts in λ_{max} from the WT). Third, we examined the ability of our models to predict λ_{max} of thirty invertebrate opsins not in *VPOD_1.0* because they are only known from physiological studies (Table S3, Figure S4). Here, we collected data both characterized by single-cell microspectrophotometry (MSP) or electroretinogram methods and with expression localized to cell-type by *in-situ-hybridization* (ISH), to link λ_{max} to a specific opsin (the sequences and metadata can be found in '*msp_erg_raw.txt'* and '*msp_erg_meta.tsv'*, while the resulting predictions can be found under the *'msp_tests'* folder on our GitHub repository). Finally, we directly compared predictive capabilities of models trained on different data subsets by randomly selecting and removing the same 25 wild-type ultraviolet or short-wave sensitive opsins from the training data of the WDS, Vertebrate, WT, and UVS/SWS models before training and querying the model with those same sequences following training (Table S3, Figure S5).

Comparing Machine Learning and Phylogenetic Imputation

We compared performance of ML models to phylogenetic imputation, which estimates phenotypes using phylogenetic information [43,44]. Phylogenetic imputation uses maximum likelihood (we will not abbreviate maximum likelihood as ML to avoid confusion with machine learning), assuming Brownian Motion to predict missing phenotypes using a phylogenetic tree, assuming more closely related species or sequences have more similar phenotypes. For the phylogeny, we constructed opsin gene trees in phyML [50], assuming the 'WAG' substitution model [51] and a proportion of 0.029 invariable sites, Gamma as a rate across sites model, and four substitution rate classes. We randomly removed 50 opsin sequences, and their corresponding λ_{max} values from each of the training datasets used to train our ML models (with the exception of the smaller MWS/LWS and invertebrate datasets, in which we only removed 15), then estimated the removed λ_{max} values using phylogenetic imputation. We used the phylogenetic imputation sub-module of the *phytools* R package [52] for performing imputation. We compared imputed and actual λ_{max} using regression. Imputation seemed sensitive to input alignment, perhaps caused

by very short or zero length branch lengths in the phylogeny, as we could only complete imputation with *phytools* after removing uninformative and heavily gapped regions with Gblocks. To allow direct comparisons of regressions between imputation and ML, we created ML training-data alignments using MAFFT, MUSCLE, and Gblocks in the same way as for imputation and had ML to predict the λ_{max} for the same set of sequences that were used to the phylogenetic imputation method (Table S6).

Testing ability of ML to account for intragenic epistasis

Functional predictions are often misled by epistasis [37], so we tested the ability of our WDS models to predict the effects of epistatic mutations by randomly selecting three double mutants with previously demonstrated epistatic effects from training data in which double mutants, each single mutant, and wild type sequence are all characterized by heterologous expression. The three epistatic double mutants are all derived from bovine rhodopsins: D83N_A292S, F261Y_A269T, and A164S_A269T. We removed the double mutants from the training dataset but retained single mutants to retain base effects of each single mutation and test whether the model treats the mutations as additive or epistatic. We hypothesized that the many instances of multi-mutant sequences with epistatic effects in the training set would allow the model to account for some level of intragenic epistasis. In a best case scenario, the model predicts both the magnitude and direction of non-additivity correctly. We then ran a separate test where we removed the same double mutants plus their corresponding single mutants so we could observe whether the WDS model would still predict epistatic effects from wild type data alone. We subsequently repeated this same process for the WT and Vertebrate models (Table S7).

Identifying known spectral tuning sites

In addition to predicting λ_{max}, we wanted to identify amino acid sites with strong effects on the phenotype, called spectral tuning sites for opsins. To do so, *deepBreaks* produces an 'importance report' of the relative importance of amino acid positions within the sequence relative to the phenotype. This report is generated for each of the top three performing models, with the addition of a column which calculates the 'mean relative importance' value of an individual position. We automated the translation of these feature representations of amino acid positions compared to bovine rhodopsin for the sake of interpretability. We also included the amino acid residue identity at each corresponding position, and whether it is in one of the opsin transmembrane domains (TMD). We used this to provide us with a standardized context for analysis of the most significant positions highlighted by the models which we could use to back-reference against the list of characterized mutants and spectral tuning sites in our database. We analyzed the importance report for each model to see what positions it highlighted as most important, with an extra emphasis placed on the output for the WT models since it was the least likely to be biased by the presence of already-known mutant data (Table S8).

Results

Data Description: A genotype-phenotype database for animal opsins

VPOD_1.0 is a new database, available on GitHub and in DataONE that currently includes all heterologously expressed animal opsins. We refer to a subset of the database with only heterologous data as *VPOD_het_1.0*, although for version 1.0, this is synonymous with the entire database. *VPOD_het_1.0* relies on 68 publications, mainly primary sources, with dates ranging from the 1980's to 2023. The database contains opsin sequences and phenotype data from 166 unique species (counting 35 reconstructed ancestors), including fishes, amphibians, reptiles, mammals, crustaceans, and bivalves. Altogether, *VPOD_het_1.0* contains 864 unique opsin sequences and corresponding λ_{max} values. This includes 318 unique WT opsins and 546 unique experimentally mutated opsins. Of the mutants, 73 are 'chimeric', meaning one or more transmembrane domains of the mutant are copied from a different opsin to replace the original. Phylogenetically, *VPOD_het_1.0* is mainly vertebrate opsins (n = 721), with only 143 unique invertebrate opsins. The vertebrate opsins consist of 113 UVS opsins, 167 SWS opsins, 8 MWS opsins, 83 LWS opsins, 237 Rhodopsin (Rh1), and 113 Rhodopsin-like (Rh2) opsins. Phenotypically, *VPOD_het_1.0* spans a range of λ_{max} values from 350-611 nm. The highest concentration of phenotype values are between 350-375 nm and 475-525 nm (Figure 1), due to the literature bias favoring characterization of UVS/SWS opsins and rhodopsins (Rh1).

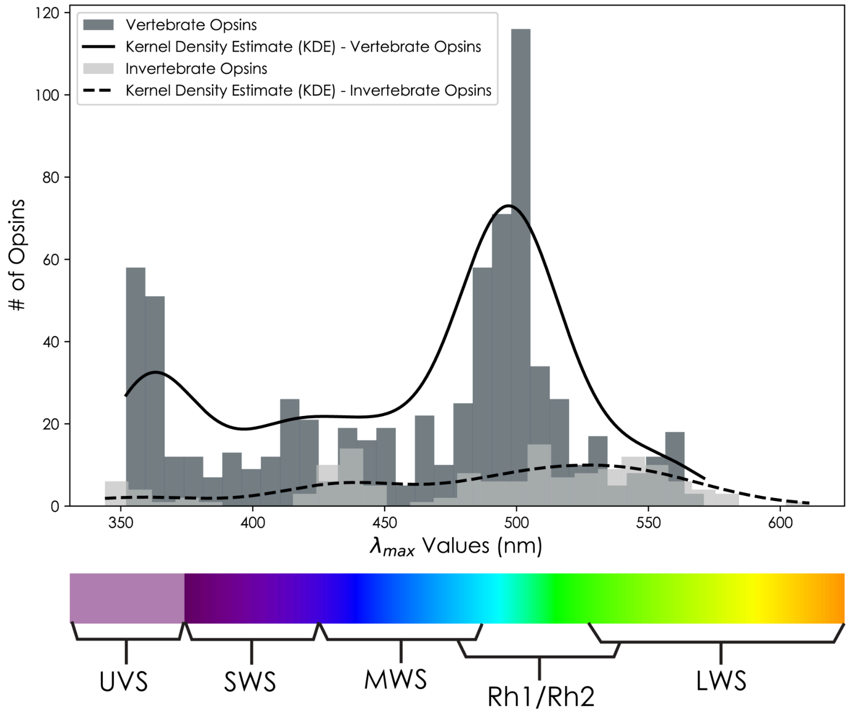

Figure 1. Histogram distributions of Vertebrate and Invertebrate Opsin Light Sensitivity Data - λmax - from *VPOD_het_1.0* with a scaled Kernel Density Estimate (KDE) curves overlaid to better visualize the general shape and characteristics of our λ_{max} distributions. Note an obvious data bias for vertebrate opsins, especially those with λ_{max} values between 350-375nm and 480-510 nm, probably due to focal research on UVS and Rh1 opsins.

The data used for model training strongly impacts accuracy

Several models trained with different subsets of data predicted λ_{max} with high accuracy (Table 1). The top-performing models from these subsets were also consistently produced using the same five algorithms, including the Gradient Boosting Regressor (GBC)[63,64], Bayesian Ridge (BR)[65,66], Light Gradient Boosting Machine (LGBM)[67], Random Forest (RF)[68], and Extreme Gradient Boosted Machine (XGB)[69]. For example, *VPOD_vert_het_1.0* - trained with all vertebrate wild-type, mutant, and chimeric opsins - had the highest 10-fold-cross-validation (CV) R^2 (0.968) and lowest mean absolute error (MAE) (6.56 nm) of any models we compared (Figure 2). Similarly, *VPOD_wds_het_1.0*, trained with the whole dataset, had very high R^2 (0.947) and low MAE (7.47 nm). The two data subsets also shared the same five top performing models (GBC, BR, LGBM, RF, and XGB). In addition, *VPOD_wt_het_1.0* - trained without mutants and only wild type data - had a similarly high R^2 (0.902) and a low MAE (10.3 nm) when predicting unseen wild type data. Overall, this 'wild type-only' model also fared well, even when predicting mutant data not included in the model (Figure 3). At the same time, many instances where mutations caused large shifts in λ_{max} (>10 nm) were not well-predicted by the wildtype-only model, as indicated by large residual values for the predictions of these mutant sequences (Figure 3). Some models, namely those with less than ~200 training sequences, were far less able to accurately predict λ_{max}. For example, *VPOD_mls_het_1.0* – trained only on the 91 MWS/LWS opsins of vertebrates – and *VPOD_inv_het_1.0* – trained only on 144 invertebrate opsins - were among the lowest R^2 (0.677 and 0.814 respectively) of all models we compared (Table 1). Comparatively, when we trained ML models on the previously published Karyasuyama type 1 opsin dataset we call *Karyasuyama_T1_ops* [41], it produced models with similar performance to the invertebrate model, with a moderate R^2 (0.804) and MAE (9.41) (Table 1). The similar levels of performances between T1 and invertebrate models were unexpected, especially considering it has a training dataset five times larger than the invertebrate model. One possible explanation is that the very old age of T1 opsins might have led to a higher complexity of genotype-phenotype associations that are not yet well sampled enough to allow good predictions.

Data availability improves predictive power, with performance thresholds and plateaus depending on the genetic diversity of the training data. Accuracy in predicting λ_{max} for our models trained on more genotypically and phenotypically diverse subsets of data (WDS, Vertebrate, WT) improves as a function of the number of sequences in a dataset, and shows an initial plateau (R^2 = ~0.80-0.90) of diminishing returns around 120-200 sequences, and continues to taper off above 200 sequences (Table S1, Figure S2). For all data subsets, we found the relationship between number of sequences in a dataset and model performance best fits a reciprocal model, which is suitable when the dependent variable plateaus as the independent variable grows larger. We found the coefficients of the reciprocal equations to be different between subsets and to increase in negative magnitude with a decrease in taxonomic/genetic diversity (the Rod model holding the largest negative value of -44). These equations do not

account directly for taxonomic, genetic, or phenotypic diversity, as the number of genes is on the x-axis. Therefore, one should be cautious about applying them to predict model performance based on training data size alone.

Although the Rod, UVS/SWS, and MWS/LWS datasets together comprise the training data for the vertebrate model (our highest performing model, $R^2 = 0.968$), the individual models trained by these data subsets performed worse than predicted by the trend between number of sequences and model performance observed for the WDS, Vertebrate, and WT subsets (S1, S2). For example, the Rod model, with 352 sequences, should have resulted in a model with an R^2 around 0.900-0.960 based on the trendlines for the WDS and Vertebrate datasets (S1, S2) but resulted in an $R^2 = 0.831$.

Table 1. Performance Metrics Across Opsin Subsets and Top Performing Models.

Name	Data Subset Version	# Seqs	Top ML Algorithm	[b]R^2	[a]MAE [nm]	MAPE [%]	[b]MSE	RMSE
Whole Dataset	*VPOD_wds_het_1.0*	864	LGBM	0.947	7.47	1.71	207	13.8
All Wild Types	*VPOD_wt_het_1.0*	318	Bayesian Ridge	0.902	10	2.18	297	16.5
All Mutants	*VPOD_mut_het_1.0*	546	LGBM	0.951	7.89	1.86	194	13.4
Vertebrates	*VPOD_vert_het_1.0*	721	LGBM	0.968	6.56	1.49	111	10.3
WT Vertebrates	*VPOD_wt_vert_het_1.0*	274	GBC	0.961	5.46	1.18	82.1	8.36
Invertebrates	*VPOD_inv_het_1.0*	143	LGBM	0.814	14.7	3.22	614	23.1
Rods	*VPOD_rod_het_1.0*	352	Bayesian Ridge	0.834	3.51	0.71	27.7	5.04
WT Rods	*VPOD_wt_rod_het_1.0*	157	GBC	0.783	3.57	0.72	31.9	5.11
MWS/LWS	*VPOD_mls_het_1.0*	91	XGB	0.677	8.77	1.82	317	15
UVS/SWS	*VPOD_uss_het_1.0*	280	GBC	0.821	8.02	2.06	200	13.6
WT UVS/SWS	*VPOD_wt_uss_het_1.0*	66	Adaboost	0.865	7.79	1.87	152	10.6
T1 Opsins	*Karyasuyama_T1_ops*	884	Random Forest	0.804	9.41	1.76	186	13.5

[a]*Mean absolute error* (MAE) is in relation to the absolute error λ_{max} predictions and interpreted in the same units of 'nm'. [b]R^2 or *mean square error* (MSE) are often interpreted as direct measures of comparing/analyzing model performance and used as training loss terms of the objective function - which measures how well the model fits the training data. One has to often balance between this and the regularization term, which controls the complexity of the model. Thus, a high performance is both simple and predictive; a tradeoff referred to as the '*bias-variance'* tradeoff.

When predicting λ_{max} of thirty unseen wild-type invertebrate opsins, almost every model performed rather poorly, with exception of the WT model (n = 30, $R^2 = 0.887$, MAE = 17.5) (Table S3, Figure S4). The best performing model produced by the sparsely populated *'Invertebrate'* dataset could only predict unseen invertebrate opsins with an R^2 of 0.837 and MAE of 26.3 nm (Table S3. Figure S5). Until the models are trained with more invertebrate

(r-opsin) data, we do not put high confidence in the estimates of λ_{max}. In contrast, when tested on their ability to predict the λ_{max} for WT SWS opsins, the Vertebrate model (R^2 = 0.914, MAE = 7.89) outperformed the predictive power of the WDS (R^2 = 0.833, MAE = 10.2), WT (R^2 = 0.773, MAE = 9.86) and SWS (R^2 = 0.788, MAE = 11.6) models, respectively (S3, S5). In other words, the Vertebrate model is both the overall top performing model and the most accurate model for predicting the λ_{max} of SWS opsins.

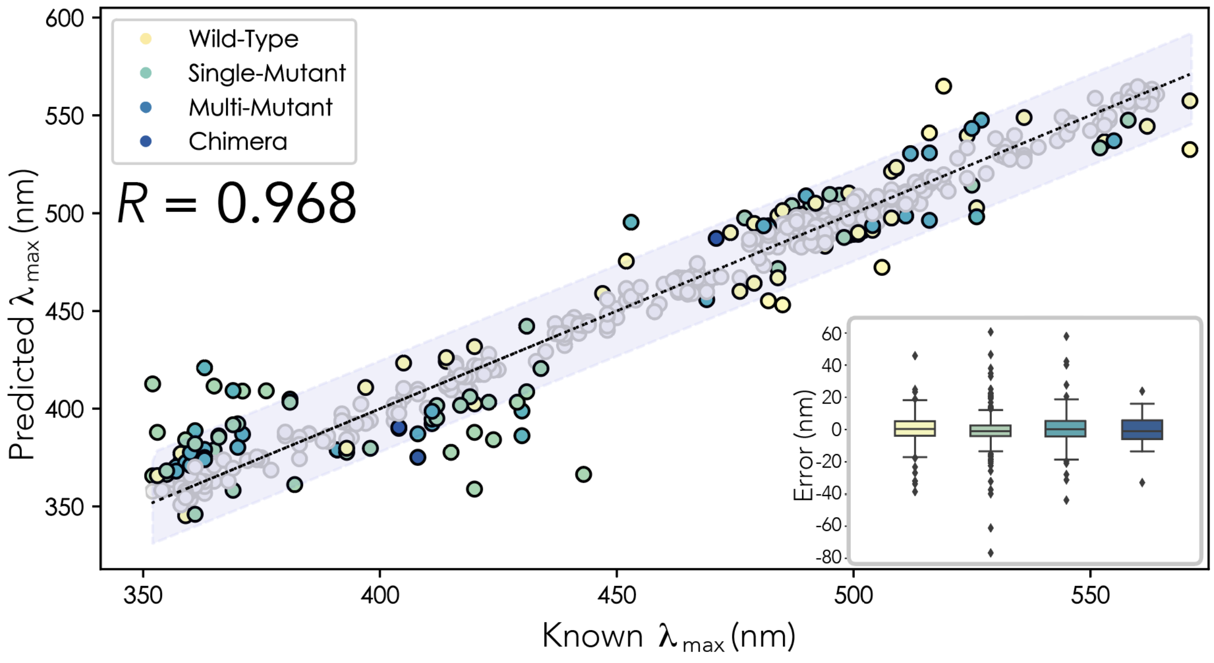

Figure 2. ML model predictions on whole Vertebrate opsin dataset, n = 721, R^2 = 0.968, MAE = 6.68nm, MAPE = 1.52. Sequences were iteratively and randomly selected to be withheld from the training dataset (n=50) to act as unseen test data. This was repeated until all sequences had been sampled once. Mutant predictions in which the absolute difference between the 'known' and 'predicted' λ_{max} are <10nm are represented by gray dots. All predictions in which the absolute difference between the 'known' and 'predicted' λ_{max} are >10nm are represented by colored dots. Yellow dots represent WT predictions, mutants with only a single mutation are green, mutants with greater than one mutation are light-blue, and chimeric opsins are dark-blue. The light-gray bar surrounding the trend-line represents a 95% confidence interval. Inset: Box-plot distribution of prediction error for different opsin data-types from the top performing Vertebrate opsin ML model to better visualize our sources of error. Note, the median for each box-plot hovers around 0nm. Single mutations have the largest spread of error, but this is most likely due to the high abundance of that data-type over all others.

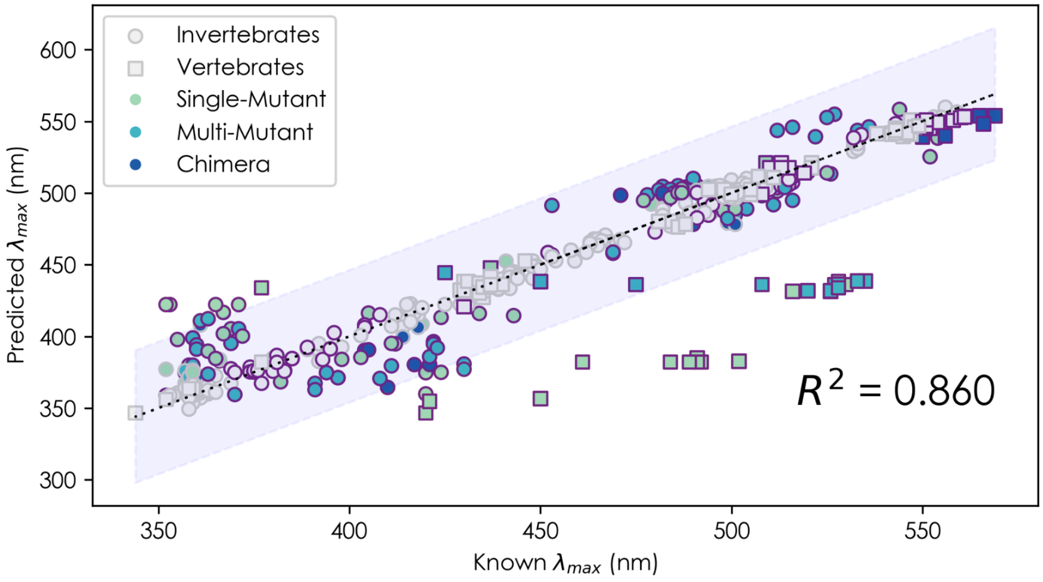

Figure 3. Scatter-plot of Wild-Type Model's λ_{max} predictions for 547 mutant opsins, with an R^2 of 0.860, MAE of 12.36 nm, and MAPE of 2.91%. Mutant predictions in which the absolute difference between the 'known' and 'predicted' λ_{max} are <10nm are represented by gray dots. All predictions in which the absolute difference between the 'known' and 'predicted' λ_{max} are >10nm are represented by colored symbols, further separated by Invertebrate (Squares) and Vertebrate (Circles) opsins. Mutants with only a single mutation are green, mutants with greater than one mutation are light-blue, and chimeric opsins are dark-blue. Mutations which caused a shift of >10nm from the WT are outlined in purple. The light-gray bar surrounding the trend-line represents a 95% confidence interval.

ML predictions of λ_{max} are comparable to phylogenetic imputation

Both ML and phylogenetic imputation were often accurate predictors of λ_{max} (Table S6). When using the same test data, ML models usually outperformed phylogenetic imputation, however slightly (S6), albeit using far less computational time, ML using on the order of minutes to calculate models and imputation using on the order of hours to generate opsin phylogenies. The MWS/LWS dataset was the only instance where phylogenetic imputation (R^2 = 0.784) largely outperformed ML (R^2 = 0.512). We found our implementation protocol for phylogenetic imputation to require removing aligned sites with extensive gaps using Gblocks, probably lessening impacts on imputation of very short branch lengths. We also used the same alignments for training ML to create direct comparison to imputation results. Interestingly, there was a slight but noticeable decrease in ML performance following Gblocks treatment for the Invertebrate, MWS/LWS, and UVS/SWS datasets (Table S6). The R^2 of the MWS/LWS model dropped from 0.677 to 0.645, while the Invertebrate model dropped from 0.814 to 0.797 (Table S6). ML performance remained relatively consistent for the WT, Vertebrate, WDS, SWS/UVS, and Rod

models, with only a slight reduction in R^2 (< 0.01) and slight increase in MAE (+/-1nm) for the WT model. The observed disparity in ML performance following Gblocks processing could be attributed to the reduced amount of features in a dataset by removing aligned sites.

ML often predicts the effects of epistatic mutations

The WDS successfully predicted three out of three instances of epistasis (Table S7) using sequences that were removed from the training data before using the model to predict known epistatic phenotypes. For double mutant D83N_A292S, the model predicted 485.2nm, which was 0.2 nm off the known λ_{max} of 485 nm. If the WDS model believed the sites were additive, the resulting λ_{max} based on adding shifts of single mutants would have been 477.5 nm. Second, for mutant F261Y_A269, the model predicted 520.0nm, for which the known λ_{max} was 520 nm. An additive prediction would have been a λ_{max} 524nm. Third, for mutant A164S_A269T the model predicted a λ_{max} of 515.5 nm, where the known λ_{max} was 514 nm. This is a special case in which the double mutant experiences a form of epistasis where the effect of mutation A269T (λ_{max} = 514) masks the shift otherwise caused by mutation A164S (λ_{max} = 502). Thus, the model seems to have correctly predicted an instance of epistasis in which one mutation masks the effect of another. We believe these results are strong evidence of the model's eventual capabilities to predict and potentially incorporate the intricacies of intragenic epistasis reliably.

We also queried the WT model with these same double mutants to test the importance of mutant sequences in informing the model on epistatic interactions. However, without any mutant data at all, the WT model did not display the same abilities to predict epistasis in any instance. For the double mutant D83N_A292S, the model predicted neither the individual mutations nor the double mutant would have a significant effect on λ_{max}, and all were predicted to be 499.9 nm. For double mutants F261Y_A269 and A164S_A269T, the WT model successfully predicted all individual mutations would cause a red shift, although F261Y and A269 were >3nm off their known λ_{max}, but incorrectly treated the mutational effects as additive for the double mutant (Table S7).

Even without information in the training data on the effect of the single experimental mutations of a pair of experimental mutations with known epistatic effects, we could accurately predict epistasis, as long as that sequence variation was present in the training data from wild type sequences. We removed the single mutants F261Y and A269T from the training data. The WDS model predicted a λ_{max} of F261Y as 508.4 nm: 1.6 nm off from the known λ_{max} of 510 nm. The model predicted a λ_{max} of 510.8 nm for mutant A269T: 3.2 nm off its known λ_{max} of 514 nm. In short, the model predicted the correct direction of phenotypic shift for the single mutants, and while the magnitude of the resulting shifts was not precisely predicted, a 2-4 nm difference between the known and predicted λ_{max} value is still less than the MAE of 7.47nm for the WDS model. As for the double mutant F261Y_A269, the model yet again predicted a λ_{max} of 520 nm, even without the direct genotype-phenotype information provided by the single mutants.

ML predicts tuning sites from Wild-Type sequences alone

The full WT model and its few variants (SWS and Rod WT models) predict several previously characterized 'spectral tuning sites' - functionally demonstrated to change λ_{max} - even with no information on mutants used in the training data (Figure 4, Table S8). For the primary WT model alone we found 15 of the top 25 amino acid sites, ranked by relative importance to the model (all ≥ 0.40), were spectral tuning sites previously characterized by mutagenesis and heterologous expression (Table S8). For example, the especially well-characterized position 308 (p308), known for its role in tuning LWS opsins, and considered to be one of the five key sites in characterizing LWS opsins under the 'Five-Site Rule' [53], had the highest relative importance value of 1.0 when using the full WT model, indicating the amino acid identity at p308 is especially important for predicting λ_{max}. In another example, the full WT model highlighted p181, a phylogenetically conserved counterion in the retinal-opsin Schiff base interaction for all non-vertebrate opsins [54]. Additionally, the transition from E to H at p181 (E181H) is a characteristic of the red-shifted vertebrate LWS opsins [32], easily visualized in Figure 4C. When predicting λ_{max} of bovine rhodopsin with mutation E181H, the WT model predicted a red-shift compared to wild type, as observed with the natural evolution of the LWS opsin lineage. The WT SWS/UVS model similarly highlighted p113, a site functionally characterized as the counterion in the retinal-opsin Schiff base interaction for all vertebrate opsins. Moreover, even the WT Rod model, trained on a mere 157 sequences, identified p292 (S8), another well-characterized and highly conserved spectral tuning site for vertebrate rhodopsins [55–57], as the site with highest relative importance to its predictions of rhodopsin λ_{max}.

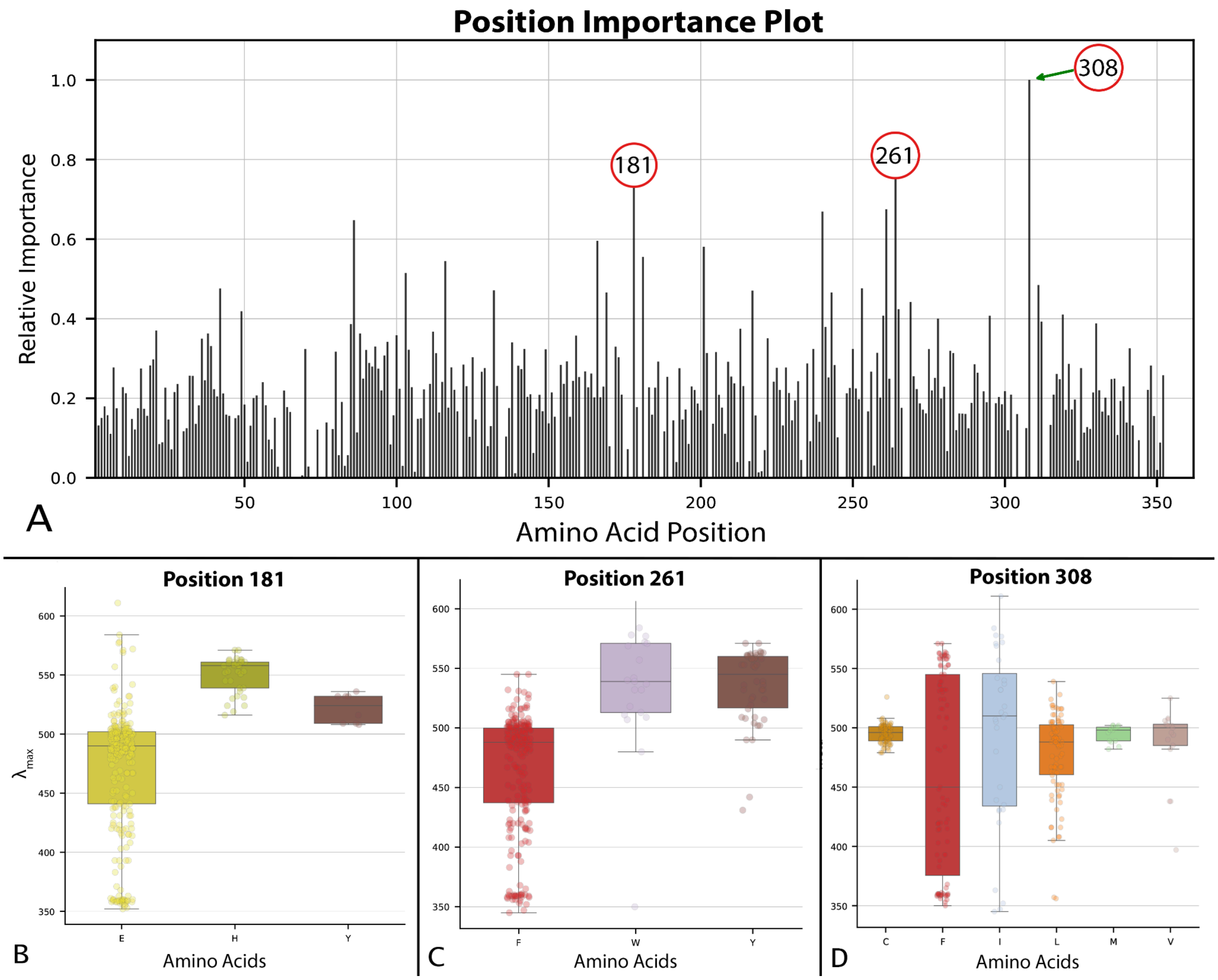

Figure 4. (**A**) Bar graph of relative importance by position generated via 'BayesianRidge' ML Regression Model trained on the WT opsin dataset. We interpret positions with higher relative importance as having a larger effect or weight on λ_{max} prediction. Positions 181, 261 and 308 are highlighted because they are among the highest scoring sites and have all been previously characterized as functionally important to opsin phenotype and function. (**B, C, & D**) These distribution box plots provide a visualization for which amino acid (aa) residues at a particular site are associated with different ranges of lambda max at a site of interest, ordered alphabetically, not by frequency (left to right).

Discussion

To better understand methods to connect genes and their functions, a fundamental goal of biology, we initiate *VPOD,* a database of opsin genes and corresponding spectral sensitivity phenotypes, as a model system for genotype-phenotype interactions. Here, we used *VPOD_1.0* to examine the ability of ML models to predict functions of opsin genes, predict intragenic epistasis, and identify amino acid sites critical for functional changes. In all cases, ML shows promise, especially when given enough training data.

The important relationship between data availability and predictive power

The predictive power of λ_{max} is generally high when using ML for opsins and it improves with a greater amount and variety of data, albeit with diminishing returns. In particular, the number of opsin genes, their genetic diversity, and the relationship between genetic and phenotypic differences are all critical in determining predictive power. Particularly illustrative of these ideas are our analyses with and without experimentally mutated opsins. Even though we might conceive of all wild type data as natural mutants chosen by evolution, experimentally induced mutations are particularly important by often changing just one amino acid that drastically changes phenotype. As such, we found that including mutant data usually improved predictive power and conversely, predicting some phenotypes from laboratory mutagenesis was sometimes difficult without including other mutant data in model training. Nevertheless, the genotype-phenotype landscape may be sampled well-enough using high numbers of only wild type genes, as evidenced by the small difference in performance when adding mutant data to the wild type subset of well-sampled vertebrate opsins (Table 1). In contrast, adding mutant data to the sparsely sampled invertebrate opsins made a big difference. Here, using only wild type data (ignoring all mutants) led to some very inaccurate predictions, especially of large phenotypic shifts caused by experimental mutagenesis of invertebrate opsins (Figure 3), suggesting the genotype-phenotype space is still undersampled for invertebrates. Therefore, targeting invertebrate (rhabdomeric) opsins should be a high priority for new additions to *VPOD*.

A large diversity of training data is also critical for reliably predicting intragenic epistasis – the non-additive effects on a phenotype of interactions between two or more mutations within a gene – which is common [10,37,39,40,58,59] and an obstacle to connecting genotypes and phenotypes [37,60–62]. Our most complete datasets (whole dataset and vertebrate data) identified known cases of intragenic epistasis, but our models trained without experimental mutagenesis data did not. Similarly to the overall predictive power of λ_{max} above, predicting epistasis probably requires sufficient variation at interacting sites, which seems especially enhanced by experimentally mutated genes.

Variation in the availability of genotype-phenotype data for training not only impacts predictive power of phenotype, but also the converse; the ability to predict amino acid sites that change λ_{max}. Several models, including those trained with the whole data set (WDS), Vertebrate, and wild type (WT) data were able to successfully predict previously characterized spectral tuning sites. This is less surprising for models trained with WDS and Vertebrate datasets, due to the prevalence of data, even including mutants in the training data from experiments which specifically targeted sites thought by researchers to be functionally informative. Yet even without any targeted mutational data, three model variants using only wild type data predicted experimentally well-characterized spectral tuning/functional sites, including sites important to the stability of the opsin-chromophore interaction (P181 and P113). This demonstrates the strong potential for ML models to identify amino acid sites that govern phenotype, leading to predictions of candidate spectral tuning sites (CSTS), which can be confirmed with mutagenesis experiments [36,56] if not done so already.

ML algorithm type contributes to the predictive power of ML models.

While probably not as important as the training data used, the ML algorithm itself also impacts predictive power. All five of the best performing ML algorithms (GBC, BR, LGBM, RF, and XGB) are variants of the decision tree model architecture (Table S9), and three out of five, including GBC, LGBM, and XGB, are 'gradient boosted' decision tree based ML algorithms. The gradient boosted algorithms all share the same general principles of gradient boosting [63,70] including the use of ensembles of 'weak learners', usually decision trees, which work sequentially and 'gradient descent' when minimizing a loss function, to improve ML model performance. While LGBM generally performed best for predicting phenotype, it was not as effective in predicting the epistatic effects of mutations, where GBC and XGB showed the highest performance. This suggests that while LGBM excels in general phenotype prediction, the details of GBC and XGB may be better suited for epistasis prediction. The difference likely arises from the unique aspects of each algorithm's model training and settings of hyperparameters. XGB and LGBM differ from GBC by the addition of a regularization term to the objective function and in the process of ensemble tree construction during model training: GBC and XGB use level-based tree fitting while LGBM uses leaf-based tree-fitting. One consequence of leaf-based tree construction is that due to its faster convergence/training time, it can be more prone to overfitting, as it constructs trees on a 'best-first basis' with a fixed number of n-terminal nodes. This creates a model that often performs well but may overgeneralize, missing finer grained collinearities and interdependencies, which would be important for predicting epistasis. As such, our models might be improved by fine-tuning hyperparameters (e.g., learning rate, max-depth, and number of estimators), and the choice of which model to use will depend on the end goals of the analysis.

The assumptions of our method and limitations of ML extrapolation.

Understanding the limitations and assumptions inherent in predictive modeling is vital for accurately interpreting animal color sensitivity from opsin sequences, especially considering the impact of various factors on sensitivity beyond the opsin itself across multiple levels of biological organization. At the cell level, we assume that λ_{max} measured in cell culture (e.g., HEK293, COS cells) is the same as in living photoreceptor cells. We also assume the photopigment uses 11-cis-retinal, as all heterologously expressed opsins in *VPOD* were reconstituted using this chromophore. However, this assumption is violated in some organisms because they use 13-cis-retinal as the *in-vivo* chromophore [71–73], which is associated with a red-shift in λ_{max} [32,71]. At the organ-level, filters such as oil droplets in bird eyes [74–77], pigments in butterfly eyes [78], or a combination of transmissive filter and narrow band reflector in mantis shrimp larval eyes [79], each may selectively influence light reaching photoreceptor cells and therefore animal color sensitivity. Finally, organismal responses to light involve neural processes, so even if an organism possesses the physiological ability to detect certain

wavelengths, it still may not have a use for that ability. Similar considerations for all these assumptions will apply when using ML to infer other functions from other genes. In fact, many genes are more susceptible than opsins (but see [80]] showing the pressure of ocean depth may slightly affect λ_{max} phenotypes) to changes in pH, temperature, and other environmental factors [81], such that databases compiling these gene functions should also record these parameters for use in training ML models.

Perhaps the most important caveat of using ML models to accurately predict phenotype or functional sites is that we assume there is a genotype-phenotype association that we can fit to a function and that our models were trained using ample data to capture these associations. Based on the non-linear fit between size of training data set, and model performance, we estimate that including about 200 sequences (and corresponding λ_{max}) from a taxonomically and phenotypically diverse range still provides improvements to model performance. Above 200 sequences, there is still improvement, but at a diminishing rate consistent with a reciprocal model (Table S1, Figure S2). When using ML for predicting functionally important sites, the addition of experimental mutants to training data that cause large phenotypic changes could heavily bias which sites are selected as 'most important' and potentially mask the importance of other sites. Here again, providing a diverse set of genotype-phenotype data should allow for the discovery of new functional sites, even when including known mutants in the training data with large phenotypic effects. Additionally, not all mutations will have the same effect on different sequences, especially if they are genetically distant; making it important to consider the level of genetic diversity used to train a model when extrapolating to find potentially important functional sites (i.e., if identifying tuning sites for rhodopsins, then using a dataset of only rhodopsins would likely be the best approach, but if data is sparse or if looking for sites that may largely impact spectral tuning across opsin subfamilies, a genetically and phenotypically broad dataset may be better).

Conclusion

Using opsin sequence data with *deepBreaks*, we were able to train regression-based ML models to reliably predict λ_{max}, often accounting for non-additive effects of mutations on function (intragenic-epistasis), and identifying amino acid sites critical for function. We expect future work will improve these already promising results even further through at least two general directions. First, adding more data to *VPOD* will improve results, especially adding invertebrate (rhabdomeric opsins) data, as technical knowledge improves for expressing these genes [35]. In addition, phenotypic data – besides the in-vitro heterologous expression targeted here – is expansive, including λ_{max} measurements from single-cell microspectrophotometry and electroretinograms, but will take considerable effort to link these phenotypes to specific opsin genes. Second, our models can be improved to take advantage of more information. One important addition should be inclusion of physicochemical properties of the amino acids [82], as implemented with success on a small scale of only 26 amino acid positions of microbial opsins to predict red-shifted phenotypes for optogenetics [83]. Additionally, information on protein

structure could be particularly important, such as the distance of an amino acid from the binding pocket of the chromophore [84]. While there are only a few solved crystal structures for opsins [85,86] to provide such data, indirect techniques like homology modeling [87] or neural network-base structural prediction [88] might be usable. Other information about opsins could also be predictive, such as which G-protein the opsin signals to, allowing prediction of which amino acids dictate G-protein specificity. Opsin kinetics [e.g. 89], or even the habitat depth at which the animal lives in the ocean, which not only influences light environment but also alters which amino acids are used in opsins [90], could improve predictive power of the ML models.

Potential Implications

Given the high performance demonstrated in this paper, current models are already robust enough to allow several applications. First, predicting λ_{max} will often be useful, especially for vertebrate opsins. For example, ML could provide an estimate of λ_{max} in a hogfish, whose skin expresses an opsin with unknown absorption and where λ_{max} has implications for a conceptual model of chromatophore expansion [91]. Second, estimates of λ_{max} from opsin sequences formed part of an argument that changes in gene expression, not sequence, adapted Amazon fishes to local light environments [92]. On broader taxonomic scales, predictions of λ_{max} from opsin sequences could expand studies of adaptation, molecular, evolution and constraint in comparison to light environments [93]. Another application could be protein design for optogenetics - the use of genetic light sensors to induce and study expression or response pathways [94–96] - including those associated with embryogenesis [97,98], stress and depression([99–101], or neuronal diseases [102,103]. Finally, our models could be used to simulate molecular evolution under a realistic genotype-phenotype landscape. One shortcoming presently for such simulations is that our models are not trained with non-functional opsins, so even non-functional genes would be predicted to have functional λ_{max} values. A solution could be to add large-scale mutagenesis data to the training set, such as that from deep mutational scanning [104]. As the *VPOD* database expands, there will be many applications for ML, and similar techniques can also be applied to other gene families such as luciferases [16,105,106].

Availability of Supporting Source Code and Requirements

Project name: The Visual Physiology Opsin Database (VPOD)
Project home page: https://github.com/VisualPhysiologyDB/visual-physiology-opsin-db
Operating system(s): Windows, MacOS, and Linux
Programming language: Python
Other requirements: Conda 4.9.2, deepBreaks 1.1.2, GBlocks 0.91b, MAFFT 7.520-1, MUSCLE 3.8.31, mySQL workbench 8.0.36, Python 3.9
License: GNU General Public License (GPL) - Version 3, 29 June 2007

Data Availability

The data set(s) supporting the results and all other code used in this article are available in the '*Visual Physiology Opsin Database*' GitHub repository, [cite unique persistent identifier] and at the '*Visual Physiology Opsin Database'* GigaDB repository, [pending acceptance] . All data and code is covered under a GNU General Public License (Version 3), in accordance with Open Source Initiative (OSI)-policies. DOME-ML (Data, Optimisation, Model, and Evaluation in Machine Learning) annotation, supporting the current study, is available through DOME Wizard.

Abbreviations

Adaboost: Adaptive Boosting
AIC: Akaike Information Criterion
COS1: Monkey kidney cell line
CV: Cross-Validation
DNA: Deoxyribonucleic Acid
ERG: Electroretinogram
GBC: Gradient Boosting Classifier
GPCR: G-Protein Coupled Receptors
HEK293: Human embryonic kidney cell line
ISH: In-situ Hybridization
KDE: Kernel Density Estimate
LGBM: Light Gradient Boosting Machine
LWS: Long-Wave Sensitive
MAE: Mean Absolute Error
MAPE: Mean Absolute Percentage Error
ML: Machine Learning
MSE: Mean Squared Error
MSP: Microspectrophotometry
MWS: Medium Wavelength-Sensitive
NCBI: National Center for Biotechnology Information
nm: Nanometers
RMSE: Root Mean Square Error
RNA: Ribonucleic Acid
SWS: Short-Wave Sensitive
T1: Type-1 {Microbial Opsins}
TMD: Transmembrane Domain
USS: Ultraviolet and Short-wave Sensitive
UVS: Ultraviolet-Sensitive
VPOD: Visual Physiology Opsin Database
WAG: Whelan and Goldman substitution model
WDS: Whole Dataset

WT: Wild-Type
XGB: Extreme Gradient Boosting
λ^{max}: Lambda Max / Wavelength of light with maximal absorbance

Competing Interests

The authors declare they have no competing interests.

Funding

This work was supported by the US National Science Foundation grants DEB-2153773 and IOS-1754770 to THO and DEB-2109688 to AR and KAC. The funders had no role in the study design, data collection and analysis, decision to publish, or preparation of the manuscript.

Authors' Contributions

THO and KAC conceived the study; SAF performed the analysis; SAF provided online documents and software. SAF and THO drafted the original manuscript. All co-authors discussed the results and edited the final manuscript.

Acknowledgments

We acknowledge funding from the National Science Foundation DEB-2153773 and IOS-1754770 to THO and DEB-2109688 to KAC and AR. We acknowledge use of computational facilities purchased with funds from the National Science Foundation (CNS-1725797) and administered by the Center for Scientific Computing (CSC). The CSC is supported by the California NanoSystems Institute and the Materials Research Science and Engineering Center (MRSEC; NSF DMR 2308708) at UC Santa Barbara. We acknowledge R. Varney for providing technical support and expertise on the phylogenetic imputation experiments. Thanks to V. Scriven for literature searches and data entry. Thanks to A. Singh and S. Yi for advice. We acknowledge Oakley Lab for comments on an early draft of the manuscript.

Citations

1. Ovsyannikova IG, Poland GA. Vaccinomics: current findings, challenges and novel approaches for vaccine development. *AAPS J*. Springer; 2011; doi: 10.1208/s12248-011-9281-x.

2. Steinbrück L, McHardy AC. Inference of Genotype–Phenotype Relationships in the Antigenic Evolution of Human Influenza A (H3N2) Viruses. *PLoS Comput Biol*. Public Library of Science; 2012; doi: 10.1371/journal.pcbi.1002492.

3. Roberts JP. Single-Cell Analysis Deepens Antibody Discovery. *Genet Eng Biotechnol News*. Mary Ann Liebert Inc; 2020; doi: 10.1089/gen.40.02.09.

4. Cobb JN, DeClerck G, Greenberg A, Clark R, McCouch S. Next-generation phenotyping: requirements and strategies for enhancing our understanding of genotype–phenotype relationships and its relevance to crop improvement. *Theor Appl Genet*. Springer; 2013; doi:

10.1007/s00122-013-2066-0.

5. Chevin L-M, Collins S, Lefèvre F. Phenotypic plasticity and evolutionary demographic responses to climate change: taking theory out to the field. *Funct Ecol*. Wiley; 2013; doi: 10.1111/j.1365-2435.2012.02043.x.

6. Franks SJ, Weber JJ, Aitken SN. Evolutionary and plastic responses to climate change in terrestrial plant populations. *Evol Appl*. Wiley Online Library; 2014; doi: 10.1111/eva.12112.

7. Gienapp P, Teplitsky C, Alho JS, Mills JA, Merilä J. Climate change and evolution: disentangling environmental and genetic responses. *Mol Ecol*. Wiley Online Library; 2008; doi: 10.1111/j.1365-294X.2007.03413.x.

8. Munday PL, Warner RR, Monro K, Pandolfi JM, Marshall DJ. Predicting evolutionary responses to climate change in the sea. *Ecol Lett*. Wiley Online Library; 2013; doi: 10.1111/ele.12185.

9. Singhal A, Simmons M, Lu Z. Text Mining Genotype-Phenotype Relationships from Biomedical Literature for Database Curation and Precision Medicine. *PLoS Comput Biol*. journals.plos.org; 2016; doi: 10.1371/journal.pcbi.1005017.

10. Kemble H, Nghe P, Tenaillon O. Recent insights into the genotype-phenotype relationship from massively parallel genetic assays. *Evol Appl*. Wiley; 2019; doi: 10.1111/eva.12846.

11. Dikicioglu D, Pir P, Oliver SG. Predicting complex phenotype-genotype interactions to enable yeast engineering: Saccharomyces cerevisiae as a model organism and a cell factory. *Biotechnol J*. Wiley; 2013; doi: 10.1002/biot.201300138.

12. Leung MKK, Delong A, Alipanahi B, Frey BJ. Machine Learning in Genomic Medicine: A Review of Computational Problems and Data Sets. *Proc IEEE*. IEEE; 2016; doi: 10.1109/JPROC.2015.2494198.

13. Guzzetta G, Jurman G, Furlanello C. A machine learning pipeline for quantitative phenotype prediction from genotype data. *BMC Bioinformatics*. 2010; doi: 10.1186/1471-2105-11-S8-S3.

14. Lee B, Zhang S, Poleksic A, Xie L. Heterogeneous Multi-Layered Network Model for Omics Data Integration and Analysis. *Front Genet*. 2019; doi: 10.3389/fgene.2019.01381.

15. Lee Y-C, Christensen JJ, Parnell LD, Smith CE, Shao J, McKeown NM, et al.. Using Machine Learning to Predict Obesity Based on Genome-Wide and Epigenome-Wide Gene-Gene and Gene-Diet Interactions. *Front Genet*. 2021; doi: 10.3389/fgene.2021.783845.

16. Yeh AH-W, Norn C, Kipnis Y, Tischer D, Pellock SJ, Evans D, et al.. De novo design of

luciferases using deep learning. *Nature*. 2023; doi: 10.1038/s41586-023-05696-3.

17. Brandes N, Goldman G, Wang CH, Ye CJ, Ntranos V. Genome-wide prediction of disease variant effects with a deep protein language model. *Nat Genet*. 2023; doi: 10.1038/s41588-023-01465-0.

18. Pinto MF, Oliveira H, Batista S, Cruz L, Pinto M, Correia I, et al.. Prediction of disease progression and outcomes in multiple sclerosis with machine learning. *Sci Rep*. nature.com; 2020; doi: 10.1038/s41598-020-78212-6.

19. Himmelstein DS, Baranzini SE. Heterogeneous Network Edge Prediction: A Data Integration Approach to Prioritize Disease-Associated Genes. *PLoS Comput Biol*. 2015; doi: 10.1371/journal.pcbi.1004259.

20. Sheikh N: Identification and classification of wildlife from camera-trap images using machine learning and computer vision. norma.ncirl.ie; https://norma.ncirl.ie/4283/1/nawazsheikh.pdf (2020). Accessed 2023 Dec 12.

21. Vélez J, McShea W, Shamon H, Castiblanco‐Camacho PJ, Tabak MA, Chalmers C, et al.. An evaluation of platforms for processing camera‐trap data using artificial intelligence. *Methods Ecol Evol*. Wiley; 2023; doi: 10.1111/2041-210x.14044.

22. Kutugata M, Baumgardt J, Goolsby JA, Racelis AE. Automatic Camera-Trap Classification Using Wildlife-Specific Deep Learning in Nilgai Management. *Journal of Fish and Wildlife Management*. Allen Press; 2021; doi: 10.3996/JFWM-20-076.

23. Govardovskii VI, Fyhrquist N, Reuter T, Kuzmin DG, Donner K. In search of the visual pigment template. *Vis Neurosci*. 2000; doi: 10.1017/s0952523800174036.

24. Jacobs GH, Neitz J, Krogh K. Electroretinogram flicker photometry and its applications. *J Opt Soc Am A Opt Image Sci Vis*. opg.optica.org; 1996; doi: 10.1364/josaa.13.000641.

25. Thomas MM, Lamb TD. Light adaptation and dark adaptation of human rod photoreceptors measured from the a-wave of the electroretinogram. *J Physiol*. Wiley Online Library; 1999; doi: 10.1111/j.1469-7793.1999.0479p.x.

26. Rocha FA de F, Gomes BD, Silveira LC de L, Martins SL, Aguiar RG, de Souza JM, et al.. Spectral Sensitivity Measured with Electroretinogram Using a Constant Response Method. *PLoS One*. journals.plos.org; 2016; doi: 10.1371/journal.pone.0147318.

27. Liebman PA. Microspectrophotometry of Photoreceptors. In: Abrahamson EW, Baumann C, Bridges CDB, Crescitelli F, Dartnall HJA, Eakin RM, et al., editors. *Photochemistry of Vision*. Berlin, Heidelberg: Springer Berlin Heidelberg;

28. Yewers MS, McLean CA, Moussalli A, Stuart-Fox D, Bennett ATD, Knott B. Spectral sensitivity of cone photoreceptors and opsin expression in two colour-divergent lineages of the lizard Ctenophorus decresii. *J Exp Biol*. journals.biologists.com; 2015; doi: 10.1242/jeb.131854.

29. Kojima D, Fukada Y. Spectroscopic Analysis of Wavelength Sensitivities of Opsin-Type Photoreceptor Proteins. In: Hirota T, Hatori M, Panda S, editors. *Circadian Clocks*. New York, NY: Springer US;

30. Carlson SD. Microspectrophotometry of visual pigments. *Q Rev Biophys*. cambridge.org; 1972; doi: 10.1017/s0033583500000986.

31. Bowmaker JK. Microspectrophotometry of vertebrate photoreceptors. A brief review. *Vision Res*. Elsevier; 1984; doi: 10.1016/0042-6989(84)90322-5.

32. Hagen JFD, Roberts NS, Johnston RJ Jr. The evolutionary history and spectral tuning of vertebrate visual opsins. *Dev Biol*. 2023; doi: 10.1016/j.ydbio.2022.10.014.

33. Yokoyama S, Radlwimmer FB. The molecular genetics and evolution of red and green color vision in vertebrates. *Genetics*. 2001; doi: 10.1093/genetics/158.4.1697.

34. Bloch NI. The evolution of opsins and color vision: connecting genotype to a complex phenotype. *Acta Biolo Colomb*. Universidad Nacional de Colombia; 2016; doi: 10.15446/abc.v21n3.53907.

35. Liénard MA, Valencia-Montoya WA, Pierce NE. Molecular advances to study the function, evolution and spectral tuning of arthropod visual opsins. *Philos Trans R Soc Lond B Biol Sci*. royalsocietypublishing.org; 2022; doi: 10.1098/rstb.2021.0279.

36. Rajamani R, Lin Y-L, Gao J. The opsin shift and mechanism of spectral tuning in rhodopsin. *J Comput Chem*. Wiley Online Library; 2011; doi: 10.1002/jcc.21663.

37. Smedley GD, McElroy KE, Feller KD, Serb JM. Additive and epistatic effects influence spectral tuning in molluscan retinochrome opsin. *J Exp Biol*. journals.biologists.com; 2022; doi: 10.1242/jeb.242929.

38. Nathans J. Determinants of visual pigment absorbance: identification of the retinylidene Schiff's base counterion in bovine rhodopsin. *Biochemistry*. 1990; doi: 10.1021/bi00493a034.

39. Yokoyama S, Xing J, Liu Y, Faggionato D, Altun A, Starmer WT. Epistatic adaptive evolution of human color vision. *PLoS Genet*. journals.plos.org; 2014; doi: 10.1371/journal.pgen.1004884.

40. Yokoyama S, Altun A, Jia H, Yang H, Koyama T, Faggionato D, et al.. Adaptive

evolutionary paths from UV reception to sensing violet light by epistatic interactions. *Sci Adv*. science.org; 2015; doi: 10.1126/sciadv.1500162.

41. Karasuyama M, Inoue K, Nakamura R, Kandori H, Takeuchi I. Understanding Colour Tuning Rules and Predicting Absorption Wavelengths of Microbial Rhodopsins by Data-Driven Machine-Learning Approach. *Sci Rep*. 2018; doi: 10.1038/s41598-018-33984-w.

42. Adam ZR, Schwieterman EW, Kacar B. Earliest photic zone niches probed by ancestral microbial rhodopsins. *Mol Biol*. academic.oup.com; 2022;

43. Molina-Venegas R, Moreno-Saiz JC, Castro Parga I, Davies TJ, Peres-Neto PR, Rodríguez MÁ. Assessing among-lineage variability in phylogenetic imputation of functional trait datasets. *Ecography* . Wiley; 2018; doi: 10.1111/ecog.03480.

44. Garland T Jr, Ives AR. Using the Past to Predict the Present: Confidence Intervals for Regression Equations in Phylogenetic Comparative Methods. *Am Nat*. 2000; doi: 10.1086/303327.

45. Katoh K, Standley DM. MAFFT multiple sequence alignment software version 7: improvements in performance and usability. *Mol Biol Evol*. academic.oup.com; 2013; doi: 10.1093/molbev/mst010.

46. Edgar RC. MUSCLE: multiple sequence alignment with high accuracy and high throughput. *Nucleic Acids Res*. academic.oup.com; 2004; doi: 10.1093/nar/gkh340.

47. Castresana J. Selection of conserved blocks from multiple alignments for their use in phylogenetic analysis. *Mol Biol Evol*. 2000; doi: 10.1093/oxfordjournals.molbev.a026334.

48. Baghbanzadeh M, Dawson T, Sayoldin B, Oakley T, Crandall K, Rahnavard A. DeepBreaks: A machine learning tool for identifying and prioritizing genotype-phenotype associations. Research Square.

49. Hunter JD. Matplotlib: A 2D Graphics Environment. *Comput Sci Eng*. IEEE; May-June 2007; doi: 10.1109/MCSE.2007.55.

50. Silva JO, Orellana ETV, Torres M. Development of a Parallel Version of PhyML 3.0 Using Shared Memory. *IEEE Latin America Transactions*. IEEE; 2017; doi: 10.1109/TLA.2017.7912593.

51. Le SQ, Gascuel O. An improved general amino acid replacement matrix. *Mol Biol Evol*. academic.oup.com; 2008; doi: 10.1093/molbev/msn067.

52. Revell LJ. phytools: an R package for phylogenetic comparative biology (and other things).

Methods Ecol Evol. Wiley; 2012; doi: 10.1111/j.2041-210x.2011.00169.x.

53. Yokoyama S, Radlwimmer FB. The "five-sites" rule and the evolution of red and green color vision in mammals. *Mol Biol Evol*. 1998; doi: 10.1093/oxfordjournals.molbev.a025956.

54. Shichida Y, Matsuyama T. Evolution of opsins and phototransduction. *Philos Trans R Soc Lond B Biol Sci*. 2009; doi: 10.1098/rstb.2009.0051.

55. Sugawara T, Terai Y, Imai H, Turner GF, Koblmüller S, Sturmbauer C, et al.. Parallelism of amino acid changes at the RH1 affecting spectral sensitivity among deep-water cichlids from Lakes Tanganyika and Malawi. *Proc Natl Acad Sci U S A*. 2005; doi: 10.1073/pnas.0405302102.

56. Takenaka N, Yokoyama S. Mechanisms of spectral tuning in the RH2 pigments of Tokay gecko and American chameleon. *Gene*. 2007; doi: 10.1016/j.gene.2007.04.036.

57. Yokoyama S, Tada T, Zhang H, Britt L. Elucidation of phenotypic adaptations: Molecular analyses of dim-light vision proteins in vertebrates. *Proc Natl Acad Sci U S A*. 2008; doi: 10.1073/pnas.0802426105.

58. Orgogozo V, Morizot B, Martin A. The differential view of genotype–phenotype relationships. *Front Genet*. frontiersin.org; 2015; doi: 10.3389/fgene.2015.00179.

59. Baldwin MW, Ko M-C. Functional evolution of vertebrate sensory receptors. *Horm Behav*. Elsevier; 2020; doi: 10.1016/j.yhbeh.2020.104771.

60. Park Y, Metzger BPH, Thornton JW. Epistatic drift causes gradual decay of predictability in protein evolution. *Science*. science.org; 2022; doi: 10.1126/science.abn6895.

61. Lyons DM, Zou Z, Xu H, Zhang J. Idiosyncratic epistasis creates universals in mutational effects and evolutionary trajectories. *Nat Ecol Evol*. nature.com; 2020; doi: 10.1038/s41559-020-01286-y.

62. Gonzalez Somermeyer L, Fleiss A, Mishin AS, Bozhanova NG, Igolkina AA, Meiler J, et al.. Heterogeneity of the GFP fitness landscape and data-driven protein design. *Elife*. elifesciences.org; 2022; doi: 10.7554/eLife.75842.

63. Friedman JH. Greedy Function Approximation: A Gradient Boosting Machine. *Ann Stat*. Institute of Mathematical Statistics; 29:1189–2322001;

64. Prettenhofer P, Louppe G. Gradient Boosted Regression Trees in Scikit-Learn. *PyData 2014*. orbi.uliege.be;

65. Bedoui A, Lazar NA. Bayesian empirical likelihood for ridge and lasso regressions. *Comput*

Stat Data Anal. Elsevier; 2020; doi: 10.1016/j.csda.2020.106917.

66. Karabatsos G. Fast Marginal Likelihood Estimation of the Ridge Parameter(s) in Ridge Regression and Generalized Ridge Regression for Big Data. arXiv [stat.ME].

67. Fan J, Ma X, Wu L, Zhang F, Yu X, Zeng W. Light Gradient Boosting Machine: An efficient soft computing model for estimating daily reference evapotranspiration with local and external meteorological data. *Agric Water Manage*. Elsevier; 2019; doi: 10.1016/j.agwat.2019.105758.

68. Segal MR. Machine Learning Benchmarks and Random Forest Regression. escholarship.org; 2004;

69. Chen T, He T, Benesty M, Khotilovich V, Tang Y, Cho H, et al.. Xgboost: extreme gradient boosting. *R package version 0 4-2*. cran.ms.unimelb.edu.au; 1:1–42015;

70. Friedman JH. Stochastic gradient boosting. *Comput Stat Data Anal*. 2002; doi: 10.1016/S0167-9473(01)00065-2.

71. Sekharan S, Morokuma K. Why 11-cis-retinal? Why not 7-cis-, 9-cis-, or 13-cis-retinal in the eye? *J Am Chem Soc*. ACS Publications; 2011; doi: 10.1021/ja208789h.

72. Buczyłko J, Saari JC, Crouch RK, Palczewski K. Mechanisms of opsin activation. *J Biol Chem*. ASBMB; 1996; doi: 10.1074/jbc.271.34.20621.

73. Terakita A. The opsins. *Genome Biol*. 2005; doi: 10.1186/gb-2005-6-3-213.

74. Das D, Wilkie SE, Hunt DM, Bowmaker JK. Visual pigments and oil droplets in the retina of a passerine bird, the canary Serinus canaria: microspectrophotometry and opsin sequences. *Vision Res*. 1999; doi: 10.1016/s0042-6989(99)00023-1.

75. Toomey MB, Collins AM, Frederiksen R, Cornwall MC, Timlin JA, Corbo JC. A complex carotenoid palette tunes avian colour vision. *J R Soc Interface*. royalsocietypublishing.org; 2015; doi: 10.1098/rsif.2015.0563.

76. Hart NS, Vorobyev M. Modelling oil droplet absorption spectra and spectral sensitivities of bird cone photoreceptors. *J Comp Physiol A Neuroethol Sens Neural Behav Physiol*. Springer; 2005; doi: 10.1007/s00359-004-0595-3.

77. Toomey MB, Corbo JC. Evolution, Development and Function of Vertebrate Cone Oil Droplets. *Front Neural Circuits*. frontiersin.org; 2017; doi: 10.3389/fncir.2017.00097.

78. Arikawa K, Stavenga D. Random array of colour filters in the eyes of butterflies. *J Exp Biol*. 1997; doi: 10.1242/jeb.200.19.2501.

79. Feller KD, Wilby D, Jacucci G, Vignolini S, Mantell J, Wardill TJ, et al.. Long-Wavelength Reflecting Filters Found in the Larval Retinas of One Mantis Shrimp Family (Nannosquillidae). *Curr Biol*. 2019; doi: 10.1016/j.cub.2019.07.070.

80. Partridge JC, White EM, Douglas RH. The effect of elevated hydrostatic pressure on the spectral absorption of deep-sea fish visual pigments. *J Exp Biol*. 2006; doi: 10.1242/jeb.01984.

81. Ogbunugafor CB, Wylie CS, Diakite I, Weinreich DM, Hartl DL. Adaptive Landscape by Environment Interactions Dictate Evolutionary Dynamics in Models of Drug Resistance. *PLoS Comput Biol*. 2016; doi: 10.1371/journal.pcbi.1004710.

82. Woolley S, Johnson J, Smith MJ, Crandall KA, McClellan DA. TreeSAAP: selection on amino acid properties using phylogenetic trees. *Bioinformatics*. 2003; doi: 10.1093/bioinformatics/btg043.

83. Inoue K, Karasuyama M, Nakamura R, Konno M, Yamada D, Mannen K, et al.. Exploration of natural red-shifted rhodopsins using a machine learning-based Bayesian experimental design. *Commun Biol*. 2021; doi: 10.1038/s42003-021-01878-9.

84. Wang W, Geiger JH, Borhan B. The photochemical determinants of color vision: revealing how opsins tune their chromophore's absorption wavelength. *Bioessays*. 2014; doi: 10.1002/bies.201300094.

85. Palczewski K, Kumasaka T, Hori T, Behnke CA, Motoshima H, Fox BA, et al.. Crystal structure of rhodopsin: A G protein-coupled receptor. *Science*. 2000; doi: 10.1126/science.289.5480.739.

86. Murakami M, Kouyama T. Crystal structure of squid rhodopsin. *Nature*. 2008; doi: 10.1038/nature06925.

87. Briscoe AD. Homology modeling suggests a functional role for parallel amino acid substitutions between bee and butterfly red- and green-sensitive opsins. *Mol Biol Evol*. 2002; doi: 10.1093/oxfordjournals.molbev.a004158.

88. Jumper J, Evans R, Pritzel A, Green T, Figurnov M, Ronneberger O, et al.. Highly accurate protein structure prediction with AlphaFold. *Nature*. 2021; doi: 10.1038/s41586-021-03819-2.

89. Van Nynatten A, Castiglione GM, de A Gutierrez E, Lovejoy NR, Chang BSW. Recreated Ancestral Opsin Associated with Marine to Freshwater Croaker Invasion Reveals Kinetic and Spectral Adaptation. *Mol Biol Evol*. 2021; doi: 10.1093/molbev/msab008.

90. Porter ML, Roberts NW, Partridge JC. Evolution under pressure and the adaptation of visual pigment compressibility in deep-sea environments. *Mol Phylogenet Evol*. Elsevier; 2016; doi:

10.1016/j.ympev.2016.08.007.

91. Schweikert LE, Bagge LE, Naughton LF, Bolin JR, Wheeler BR, Grace MS, et al.. Dynamic light filtering over dermal opsin as a sensory feedback system in fish color change. *Nat Commun*. Nature Publishing Group UK London; 14:46422023;

92. Borghezan E de A, da Silva Pires TH, Zuanon J, Sugiura H, Kohshima S, Kishida T. Unstable environmental conditions constrain the fine-tune between opsin sensitivity and underwater light in an Amazon forest stream fish. *J Evol Biol*. Oxford University Press; 2024; doi: 10.1093/jeb/voae001.

93. Murphy MJ, Westerman EL. Evolutionary history limits species' ability to match colour sensitivity to available habitat light. *Proc Biol Sci*. 2022; doi: 10.1098/rspb.2022.0612.

94. Kwon E, Heo WD. Optogenetic tools for dissecting complex intracellular signaling pathways. *Biochem Biophys Res Commun*. 2020; doi: 10.1016/j.bbrc.2019.12.132.

95. Mukherjee A, Repina NA, Schaffer DV, Kane RS. Optogenetic tools for cell biological applications. J. Thorac. Dis.

96. Tischer D, Weiner OD. Illuminating cell signalling with optogenetic tools. *Nat Rev Mol Cell Biol*. 2014; doi: 10.1038/nrm3837.

97. Kaur P, Saunders TE, Tolwinski NS. Coupling optogenetics and light-sheet microscopy, a method to study Wnt signaling during embryogenesis. *Sci Rep*. nature.com; 2017; doi: 10.1038/s41598-017-16879-0.

98. Fan H, Barnes C, Hwang H, Zhang K, Yang J. Precise modulation of embryonic development through optogenetics. *Genesis*. Wiley Online Library; 2022; doi: 10.1002/dvg.23505.

99. Sparta DR, Jennings JH, Ung RL, Stuber GD. Optogenetic strategies to investigate neural circuitry engaged by stress. *Behav Brain Res*. Elsevier; 2013; doi: 10.1016/j.bbr.2013.05.007.

100. Belzung C, Turiault M, Griebel G. Optogenetics to study the circuits of fear- and depression-like behaviors: a critical analysis. *Pharmacol Biochem Behav*. Elsevier; 2014; doi: 10.1016/j.pbb.2014.04.002.

101. Muir J, Lopez J, Bagot RC. Wiring the depressed brain: optogenetic and chemogenetic circuit interrogation in animal models of depression. *Neuropsychopharmacology*. nature.com; 2019; doi: 10.1038/s41386-018-0291-6.

102. LaLumiere RT. A new technique for controlling the brain: optogenetics and its potential for use in research and the clinic. *Brain Stimul*. 2011; doi: 10.1016/j.brs.2010.09.009.

103. Montagni E, Resta F, Mascaro ALA, Pavone FS. Optogenetics in Brain Research: From a Strategy to Investigate Physiological Function to a Therapeutic Tool. *Photonics*. Multidisciplinary Digital Publishing Institute; 2019; doi: 10.3390/photonics6030092.

104. Penn WD, McKee AG, Kuntz CP, Woods H, Nash V, Gruenhagen TC, et al.. Probing biophysical sequence constraints within the transmembrane domains of rhodopsin by deep mutational scanning. *Sci Adv*. 2020; doi: 10.1126/sciadv.aay7505.

105. Hensley NM, Ellis EA, Leung NY, Coupart J, Mikhailovsky A, Taketa DA, et al.. Selection, drift, and constraint in cypridinid luciferases and the diversification of bioluminescent signals in sea fireflies. *Mol Ecol*. 2021; doi: 10.1111/mec.15673.

106. Schenkmayerova A, Pinto GP, Toul M, Marek M, Hernychova L, Planas-Iglesias J, et al.. Engineering the protein dynamics of an ancestral luciferase. *Nat Commun*. 2021; doi: 10.1038/s41467-021-23450-z.

Supplementary Material

Supplementary Material 1 (S1):

Tracking Model Performance vs. Number of Sequences in Training Data

Data Subset	# of Sequences	R2 - T1	R2 - T2	R2 - T3	R2 - AVG	RD - R2
WDS	864	0.947	0.947	0.947	0.947	0.96
	814	0.941	0.943	0.946	0.943	RD - AIC
	764	0.937	0.945	0.943	0.942	-162.00
	714	0.935	0.946	0.940	0.940	
	664	0.929	0.944	0.938	0.937	
	614	0.928	0.938	0.945	0.937	
	564	0.919	0.936	0.936	0.930	
	514	0.912	0.922	0.944	0.926	
	464	0.916	0.922	0.933	0.924	
	414	0.920	0.918	0.932	0.924	
	364	0.924	0.903	0.923	0.917	
	314	0.921	0.911	0.911	0.914	
	264	0.919	0.888	0.911	0.906	
	214	0.921	0.893	0.900	0.905	
	164	0.912	0.895	0.833	0.880	
	114	0.879	0.895	0.862	0.879	
	64	0.765	0.799	0.713	0.759	

Data Subset	# of Sequences	R2 - T1	R2 - T2	R2 - T3	R2 - AVG	RD - R2
Vertebrate	721	0.968	0.968	0.968	0.968	0.99
	671	0.963	0.966	0.965	0.965	RD - AIC
	621	0.960	0.969	0.965	0.965	-160.00
	571	0.955	0.968	0.961	0.961	
	521	0.949	0.966	0.965	0.960	
	471	0.951	0.964	0.965	0.960	
	421	0.952	0.961	0.966	0.960	
	371	0.951	0.956	0.962	0.956	
	321	0.944	0.961	0.962	0.956	
	271	0.942	0.952	0.951	0.948	
	221	0.938	0.928	0.949	0.939	
	171	0.928	0.907	0.938	0.924	
	121	0.896	0.910	0.911	0.906	
	71	0.925	0.816	0.868	0.870	

Data Subset	# of Sequences	R2 - T1	R2 - T2	R2 - T3	R2 - AVG	RD - R2
Rod	352	0.834	0.834	0.834	0.834	0.93
	337	0.825517	0.808289	0.802864	0.812223	AIC - R2
	322	0.81498	0.835739	0.83895	0.82989	-122.00
	307	0.864023	0.831771	0.850227	0.848674	
	292	0.83162	0.853479	0.804649	0.829916	
	277	0.832486	0.811542	0.827711	0.823913	
	262	0.852768	0.821254	0.839961	0.837994	
	247	0.798275	0.82422	0.818958	0.813817	
	232	0.838121	0.773139	0.789483	0.800248	
	217	0.799491	0.773947	0.768506	0.780648	
	202	0.840374	0.749616	0.759987	0.783326	
	187	0.812757	0.782659	0.800555	0.798657	
	172	0.772936	0.746982	0.777117	0.765678	
	157	0.740628	0.725929	0.726127	0.730895	
	142	0.727746	0.807972	0.668367	0.734695	
	127	0.692876	0.70442	0.765842	0.721046	
	112	0.703357	0.641533	0.74384	0.696243	
	97	0.628481	0.663658	0.763486	0.685208	
	82	0.453915	0.414098	0.603912	0.490642	
	67	0.105291	0.047832	0.556672	0.236599	
	52	-0.09295	0.041141	0.340028	0.096072	

Data Subset	# of Sequences	R2 - T1	R2 - T2	R2 - T3	R2 - AVG	RD_R2
WT	318	0.902	0.902	0.902	0.902	0.84
	303	0.873	0.911	0.899	0.892	RD_AIC
	288	0.891	0.876	0.873	0.884	-89.00
	273	0.890	0.866	0.868	0.878	
	258	0.881	0.859	0.879	0.870	
	243	0.854	0.884	0.825	0.869	
	228	0.851	0.875	0.873	0.863	
	213	0.826	0.775	0.863	0.800	
	198	0.870	0.856	0.856	0.863	
	183	0.866	0.840	0.849	0.853	
	168	0.856	0.830	0.776	0.843	
	153	0.751	0.826	0.832	0.788	
	138	0.744	0.785	0.806	0.765	
	123	0.747	0.855	0.808	0.801	

108	0.712	0.864	0.738	0.788
93	0.749	0.886	0.574	0.817
78	0.660	0.731	0.747	0.695
63	0.731	0.715	0.460	0.723
48	0.343	0.654	0.677	0.499
33	-0.277			-0.277

T = Test , RD = Reciprocal Decay, AIC = Akaike Information Criterion

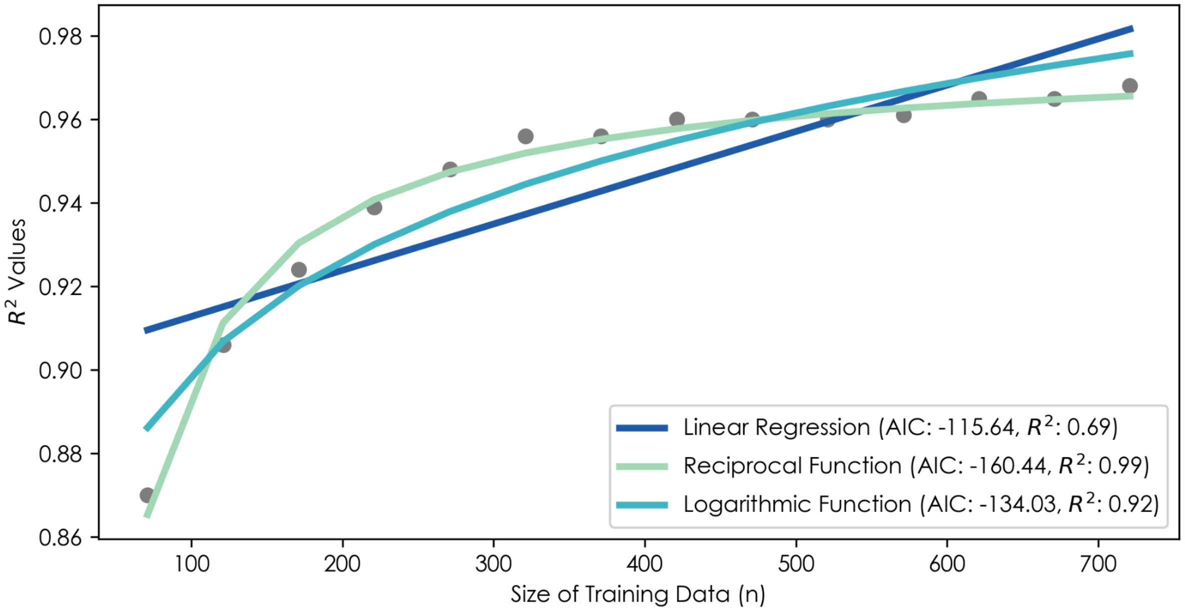

Supplementary Material 2 (S2): Three functions fitted to visualize the relationship between training data size (number of genotypes and corresponding phenotypes) vs. model performance (R2) based on results from the Vertebrate subset of data. The Akaike Information Criterion (AIC) is a measure used for model selection when comparing different statistical models, accounting for both the goodness of fit of the model and the simplicity of the model (the number of parameters used). The goal is to find a balance between a model's ability to explain the data and its complexity, preventing overfitting.

Supplementary Material 3 (S3):
Comparing ML Predictions on Invertebrate and Vertebrate UVS/SWS Opsin MSP Data

Data Split	MSP R2	MAE [nm]	MAPE [%]	SWS Test R2	MAE [nm]	MAPE [%]
Whole Dataset	0.704	30.6	6.8	0.833	10.2	2.45
Vertebrates	0.044	70.8	14.6	0.914	7.89	1.9
Invertebrates	0.837	26.3	6.95	-	-	-
Wild Types Only	0.887	17.5	4.06	0.773	9.86	2.46
UVS/SWS	-	-	-	0.788	11.6	2.92

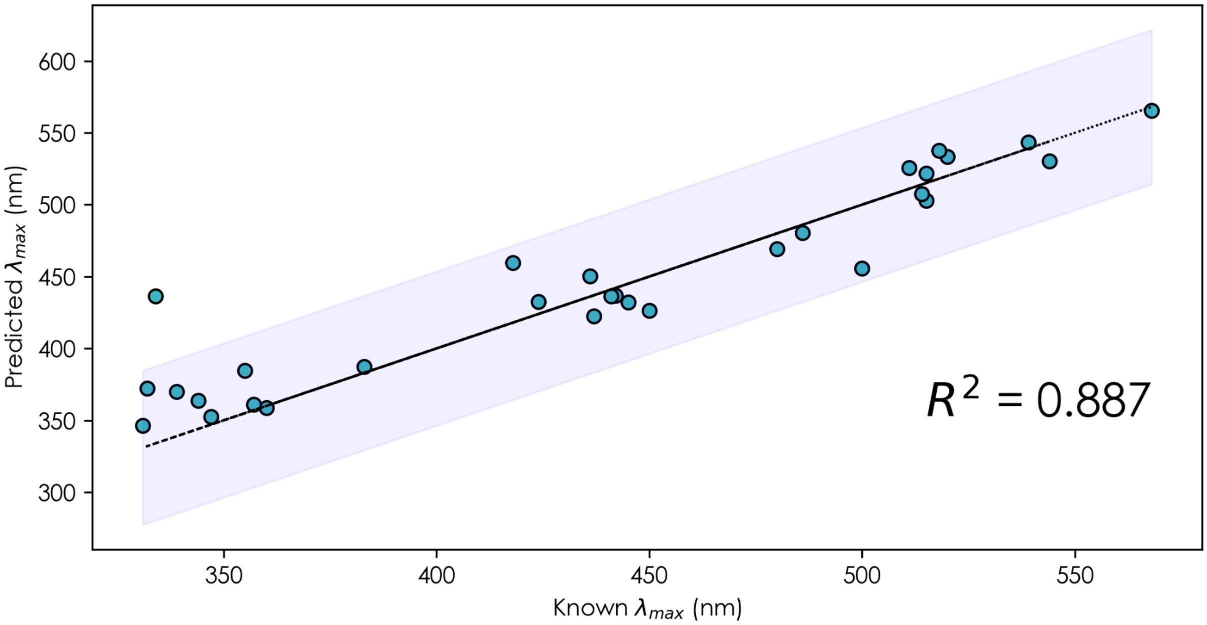

Supplementary 4: Graph of WT model predictions for 30 unseen invertebrate opsins, R2 = 0.887, MAE =17.5nm, MAPE = 4.05. All the 'known' λmax values are from physiological measures, including MSP or ERG measurements (instead of purified heterologously expressed opsins), and are linked to a particular opsin sequence by in-situ hybridization. The light-gray bar surrounding the trend-line represents a 95% confidence interval.

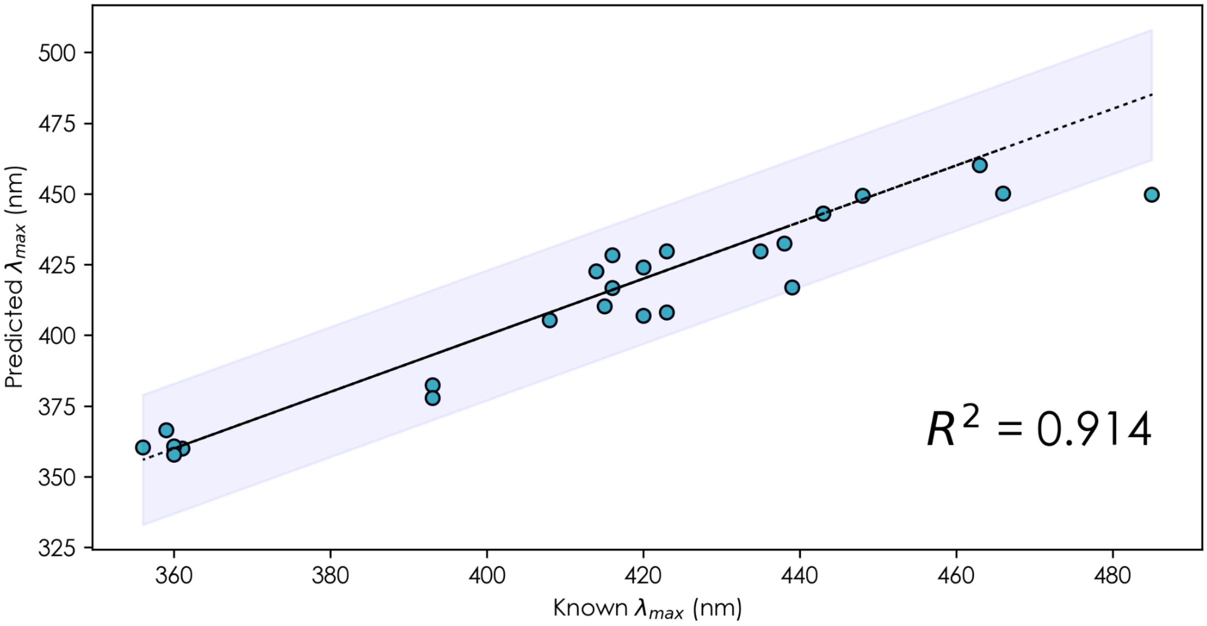

Supplementary 5 (S5): Graph of Vertebrate model predictions for unseen WT-UVS/SWS data, n = 25, R2 = 0.914, MAE =7.89 nm, MAPE = 1.90. All sequences were randomly selected from the UVS/SWS model under the condition that they were WT opsins. The light-gray bar surrounding the trend-line represents a 95% confidence interval.

Supplementary 6 (S6):
Comparing Performances of ML Predictions and Phylogenetic Imputation on a Subsample of Opsin Data

Subset	MAFFT Sample R2	MUSCLE deepBreaks R2	MUSCLE Sample R2	Gblock deepBreaks R2	Gblock Sample R2	Imputation Sample Adj. R2	Imputation . MAE (nm)	Imputation MAPE (%)	Sample Size (n)
Wild-Type	0.868	893	0.918	0.900	0.863	0.836	8.78	1.85	50
Vertebrates	0.968	0.967	0.972	0.967	0.967	0.949	6.76	1.48	50
WDS	0.948	0.946	0.96	0.942	0.964	0.958	7.6	1.65	50
Rods	0.848	0.843	0.824	0.843	0.865	0.713	4.36	0.877	50
Invertebrates	0.706	0.814	0.711	0.797	0.678	0.758	18.3	3.43	15
UVS/SWS	0.87	0.818	0.919	0.820	0.921	0.922	10.9	2.78	25
MWS/LWS	0.499	0.657	0.497	0.645	0.512	0.784	5.91	1.12	15

Supplementary 7 (S7) : Results for epistasis test on the WDS, Vertebrate, WT, and Rod models.

Mutant	Known λmax (nm)	WDS λmax Prediction (nm)	Vertebrate λmax Prediction (nm)	WT λmax Prediction (nm)
NM_001014890 [WT]	500	-	-	-
NM_001014890_D83N_A292S	485	485.2	483.95	499.91
NM_001014890.2_F261Y_A269T	520	520.0	515.39	515.3875
NM_001014890.2_A164S_A269T	514	515.5	513.8	510.2689344

Supplementary 8 (S8):
Functionally characterized spectral tuning sites predicted by the WT models

Data Subset	Position on Bovine	Importance Value (0.0-1.0)	AA Residue on Bovine	TMD	Verified Tuning Site
All WT Opsins	308	1	M	7	Yes
	261	0.675	F	6	Yes
	86	0.648	M	2	Yes
	201	0.581	E	CT/EC	Yes
	181	0.555	E	CT/EC	Yes
	116	0.545	F	3	Yes
	253	0.476	M	6	No
	42	0.476	A	1	No
	217	0.471	I	5	Yes
	169	0.466	A	4	Yes
	243				No
	49	0.419	M	1	Yes
	295	0.407	A	7	Yes
	269	0.442	A	6	Yes
Rod WT Opsins	292	1	A	7	Yes
	122	0.417	E	3	Yes

Supplementary 8 (S8):
Functionally characterized spectral tuning sites predicted by the WT models

Data Subset	Position on Bovine	Importance Value (0.0-1.0)	AA Residue on Bovine	TMD	Verified Tuning Site
All WT Opsins	308	1	M	7	Yes
	261	0.675	F	6	Yes
	86	0.648	M	2	Yes
	201	0.581	E	CT/EC	Yes
	181	0.555	E	CT/EC	Yes
	116	0.545	F	3	Yes
	253	0.476	M	6	No
	42	0.476	A	1	No
	217	0.471	I	5	Yes
	169	0.466	A	4	Yes
	243				No
	49	0.419	M	1	Yes
	295	0.407	A	7	Yes
	269	0.442	A	6	Yes
Rod WT Opsins	292	1	A	7	Yes
	205	0.256	I	5	Yes
SWS WT Opsins	113	0.302	T	3	Yes
	81	0.156	M	2	Yes
	114	0.124	L	3	Yes

Supplementary 9 (S9):
Ranked ML Algorithm Performances

Model Algorithm	Top Model Count	Total Points	Overall Ranking
gbc	3	120	1
BayesianRidge	2	104	2
lgbm	4	100	3/4
rf	1	100	3/4
XGB	1	87	4
ET	0	68	5
Adaboost	1	51	6
DT	0	50	7
LassoLars	0	48	8
Lasso	0	41	9
HubR	0	27	10
LR	0	0	12
