# Supplementary material for: Discovering genotype–phenotype relationships with machine learning and the Visual Physiology Opsin Database (VPOD)
Source: Gigascience. 2024 Oct 26;13:giae073. doi: 10.1093/gigascience/giae073 (PMC11512451; doi:10.1093/gigascience/giae073)
Supplement: giae073_GIGA-D-24-00053_Revision_2 [file giae073_giga-d-24-00053_revision_2.pdf]

GigaScience

Discovering genotype-phenotype relationships with machine learning and the Visual Physiology Opsin Database (VPOD)

--Manuscript Draft--

**Manuscript Number:**	GIGA-D-24-00053R2
**Full Title:**	Discovering genotype-phenotype relationships with machine learning and the Visual Physiology Opsin Database (VPOD)
**Article Type:**	Research
**Funding Information:**	Directorate for Biological Sciences (DEB-2153773) — Prof Todd Oakley<br>Directorate for Biological Sciences (IOS-1754770) — Prof Todd Oakley<br>Directorate for Biological Sciences (DEB-2109688) — Dr. Keith A. Crandall
**Abstract:**	Background: Predicting phenotypes from genetic variation is foundational for fields as diverse as bioengineering and global change biology, highlighting the importance of efficient methods to predict gene functions. Linking genetic changes to phenotypic changes has been a goal of decades of experimental work, especially for some model gene families including light-sensitive opsin proteins. Opsins can be expressed in vitro to measure light absorption parameters, including λmax - the wavelength of maximum absorbance - which strongly affects organismal phenotypes like color vision. Despite extensive research on opsins, the data remain dispersed, uncompiled, and often challenging to access, thereby precluding systematic and comprehensive analyses of the intricate relationships between genotype and phenotype.<br>Results: Here, we report a newly compiled database of all heterologously expressed opsin genes with λmax phenotypes that we call the Visual Physiology Opsin Database (VPOD). VPOD_1.0 contains 864 unique opsin genotypes and corresponding λmax phenotypes collected across all animals from 73 separate publications. We use VPOD data and deepBreaks to show regression-based machine learning (ML) models often reliably predict λmax, account for non-additive effects of mutations on function, and identify functionally critical amino acid sites.<br>Conclusion: The ability to reliably predict functions from gene sequences alone using ML will allow robust exploration of molecular-evolutionary patterns governing phenotype, will inform functional and evolutionary connections to an organism's ecological niche, and may be used more broadly for de-novo protein design. Together, our database, phenotype predictions, and model comparisons lay the groundwork for future research applicable to families of genes with quantifiable and comparable phenotypes.
**Corresponding Author:**	Todd Oakley, PhD<br>UCSB: University of California Santa Barbara<br>Santa Barbara, CA UNITED STATES
**Corresponding Author Secondary Information:**	
**Corresponding Author's Institution:**	UCSB: University of California Santa Barbara
**Corresponding Author's Secondary Institution:**	
**First Author:**	Seth A. Frazer, BS
**First Author Secondary Information:**	
**Order of Authors:**	Seth A. Frazer, BS<br>Mahdi Baghbanzadeh, BS<br>Ali Rahnavard, PhD<br>Keith A. Crandall, PhD

Powered by Editorial Manager® and ProduXion Manager® from Aries Systems Corporation

	Todd Oakley, PhD
**Order of Authors Secondary Information:**	
**Response to Reviewers:**	Please include a point-by-point within the 'Response to Reviewers' box in the submission system. Please ensure you describe additional experiments that were carried out and include a detailed rebuttal of any criticisms or requested revisions that you disagreed with. Please also ensure that your revised manuscript conforms to the journal style, which can be found in the Instructions for Authors on the journal homepage. If the data and code has been modified in the revision process please be sure to update the public versions of this too.<br>--<br><br>We thank the reviewers and editor for their time and comments on our manuscript. Herein we respond to the comments inline, address concerns, and note specific changes to the manuscript. Our responses begin with ****. Reviewer suggestions begin with —-<br><br>Sincerely,<br><br>Seth A. Frazer et al.<br><br>--<br><br>Reviewer reports:<br><br>—Reviewer #2: The authors have addressed the majority of my concerns and improved the manuscript. However, there are still some remaining issues that the authors should address before I would recommend the publication of the manuscript.<br><br>1. Dependence between data points is not a novel problem for data analysis and machine learning in a broad range of subjects. While I appreciate that the authors added a paragraph discussing the issue of phylogenetic relatedness, the setup of the cross-validation and how the data is presented make it difficult to assess to which extend their models over-fit to the data. Referring to their previous reply, lineage-/group-based cross-validation should not be arbitrarily chosen but based on the structure of the data. This is not always a trivial problem and magic solution, I agree. The authors should at least incorporate references to literature discussing the problem and potential solutions for dealing with phylogenetic relatedness at p.11 "While these performance metrics are impressive, it is important to remember that phylogenetic relatedness between sequences..." or in the discussion. For example, Roberts et al. provide a nice overview for cross-validation strategies in<br>various settings including phylogenetic data (they call it "block cross-validation"):<br><br>Roberts et al. (2017). Cross-validation strategies for data with temporal, spatial, hierarchical, or phylogenetic structure. Ecography, 40(8), 913-929.<br><br>**** We thank the reviewer for this comment and we agree that this is an important issue to address within the future directions of our research to unpack the extent to which we can extrapolate/rely on predictions made by these models on new data. As suggested by the reviewer, we now added the suggested reference and the sentence "Roberts et al. [79] provide a discussion of alternative cross-validation strategies such as "block cross-validation" for non-independent data types, including phylogenetically related data which can help mitigate this issue".<br><br><br>—2. Regarding the comparison between models trained on wild type (WT) and WT + mutant data (WDS), I find the comparison rather difficult to follow concerning repeatedly leaving out 25 mutants. For comparing the models, the test set (or test sets) should be the same. This would mean to assess the predictions for the same 25 left out mutants by both the WT and the WDS model (for each 25 left out mutants). If this was done already I would recommend rephrasing the corresponding part in the methods and results to improve the clarity.

**** We thank the reviewer for their insight into the interpretability of this experiment and would like to note that the WT model has no mutants at all in its training so it automatically leaves out the 25 mutants removed in each iteration of training that the WDS model goes through.
To clarify/rephrase the methods further - The WT model is ONLY trained on WT opsin data, thus can simply be tested on all mutants at once AFTER training of the model is complete - it is not used as test data during the actual model training. The WDS in this test is iteratively trained on the entire dataset with only 25 mutants removed at a time and used as test data following the completion of training, so it is not used as test data during the actual training process. This is repeated until all mutants from the WDS have been sampled and tested once.

Here is our modification to that section in context:
"To accomplish this for the WDS models, we iteratively removed 25 mutant opsins at a time from training data, used the same training algorithm (Gradient Boosted Regressor, GBR), and predicted λmax values of withheld opsins following the completion of model training (withheld opsins are not used as test data during the actual model training), until all mutant opsins were sampled once (this notebooks is available on GitHub as 'vpod_wf_iterate_subsample.ipynb'."

We did not modify the corresponding results section as the methods underlying this iterative process are not directly mentioned there.

—In addition, a visualization, for instance, a violin plot of the WT model RMSEs vs. violin plot of the WDS model RMSEs would be useful for the readers.

**** We have added two supplementary figures using cloud plots to visualize the absolute error distributions of the WT vs WDS mutant prediction test (Figure S11) and another for the test comparing the prediction error of the Expected Additive Mutation Values, WT model, and WDS-minusepi model (Figure S12). We've also referenced them in the main text in their corresponding sections.

—3. I would still recommend adding a brief summary of how feature importance scores are computed. So, the reader does not have to look up another manuscript. This does not have to be detailed. As I understand, the feature importance is just the normalized number of feature occurrences or the Gini importance for gradient boosting/random forests or the coefficient for regression models.

**** We have added a slightly more detailed explanation of how importance scores are computed and have provide it below:
"The metrics used to determine these relative importance scores of each position vary based on the structure and output of the algorithms used for model training. For example, xgboost [60] and LightGBM [61,62] use the number of times a feature appears in a tree as a proxy for importance [59], while AdaBoost [63] and random forest [64,65], use Gini importance, which quantifies a feature's contribution to improving prediction accuracy [59,66,67]. For a more detailed explanation on how position importance scores are calculated for different models, refer to the 'Interpretation' heading under the methods section of the deepBreaks publication [59]."

—Minor details:

—Fig. S10: the text at the leaves is not readable. It could be replaced, for instance, with the name of the gene family if that makes sense, or removed.

**** We appreciate the suggestion made by the reviewer but we've decided to not remove text at the leaves and have instead supplied a higher resolution image of the tree in the supplement, namely as a vector-based graphic, for the tree so that readers can zoom in on the tips more clearly, if so desired.

—Fig. 4A: the bars at position 181, 261, and 308, could be indicated, for example, in red or another color, to easier compare A and B

Powered by Editorial Manager® and ProduXion Manager® from Aries Systems Corporation

	**** We have modified figure 4A and 4B as suggested by changing the color of the bars at position 181, 261, and 308 to purple for the sake of easier comparison.
**Additional Information:**	
**Question**	**Response**
Are you submitting this manuscript to a special series or article collection?	No
**Experimental design and statistics**<br><br>Full details of the experimental design and statistical methods used should be given in the Methods section, as detailed in our Minimum Standards Reporting Checklist. Information essential to interpreting the data presented should be made available in the figure legends.<br><br>Have you included all the information requested in your manuscript?	Yes
**Resources**<br><br>A description of all resources used, including antibodies, cell lines, animals and software tools, with enough information to allow them to be uniquely identified, should be included in the Methods section. Authors are strongly encouraged to cite Research Resource Identifiers (RRIDs) for antibodies, model organisms and tools, where possible.<br><br>Have you included the information requested as detailed in our Minimum Standards Reporting Checklist?	Yes
**Availability of data and materials**<br><br>All datasets and code on which the conclusions of the paper rely must be either included in your submission or deposited in publicly available repositories (where available and ethically appropriate), referencing such data using a unique identifier in the references and in the “Availability of Data and Materials”	Yes

Powered by Editorial Manager® and ProduXion Manager® from Aries Systems Corporation

section of your manuscript.<br><br>Have you have met the above requirement as detailed in our Minimum Standards Reporting Checklist?	

Manuscript
Click here to access/download;Manuscript;Revision 2 Frazer et al.docx

Discovering genotype-phenotype relationships with machine learning and the Visual Physiology Opsin Database (VPOD)

Seth A. Frazer[1], Mahdi Baghbanzadeh[2], Ali Rahnavard[2], Keith A. Crandall[2,3], Todd H. Oakley[1,*]

Affiliations:

[1]Ecology, Evolution, and Marine Biology, University of California, Santa Barbara, California 93106

[2]Computational Biology Institute, Department of Biostatistics and Bioinformatics, Milken Institute School of Public Health, The George Washington University, Washington, DC 20052

[3]Department of Invertebrate Zoology, National Museum of Natural History, Smithsonian Institution, Washington, DC 20012

ORCID and Email:

Seth A. Frazer
sethfrazer@ucsb.edu
https://orcid.org/0000-0002-3800-212X

Mahdi Baghbanzadeh
mbagh@gwu.edu
https://orcid.org/0000-0002-1878-2691

Keith A. Crandall
kcrandall@gwu.edu
https://orcid.org/0000-0002-0836-3389

Ali Rahnavard
rahnavard@gwu.edu
https://orcid.org/0000-0002-9710-0248

Todd H. Oakley
oakley@ucsb.edu
https://orcid.org/0000-0002-4478-915X
*Correspondence to oakley@ucsb.edu

Abstract

Background: Predicting phenotypes from genetic variation is foundational for fields as diverse as bioengineering and global change biology, highlighting the importance of efficient methods to predict gene functions. Linking genetic changes to phenotypic changes has been a goal of decades of experimental work, especially for some model gene families including light-sensitive opsin proteins. Opsins can be expressed in vitro to measure light absorption parameters, including λ_{max} - the wavelength of maximum absorbance - which strongly affects organismal phenotypes like color vision. Despite extensive research on opsins, the data remain dispersed, uncompiled, and often challenging to access, thereby precluding systematic and comprehensive analyses of the intricate relationships between genotype and phenotype.

Results: Here, we report a newly compiled database of all heterologously expressed opsin genes with λ_{max} phenotypes that we call the Visual Physiology Opsin Database (*VPOD*). *VPOD_1.0* contains 864 unique opsin genotypes and corresponding λ_{max} phenotypes collected across all animals from 73 separate publications. We use *VPOD* data and *deepBreaks* to show regression-based machine learning (ML) models often reliably predict λ_{max}, account for non-additive effects of mutations on function, and identify functionally critical amino acid sites.

Conclusion: The ability to reliably predict functions from gene sequences alone using ML will allow robust exploration of molecular-evolutionary patterns governing phenotype, will inform functional and evolutionary connections to an organism's ecological niche, and may be used more broadly for *de-novo* protein design. Together, our database, phenotype predictions, and model comparisons lay the groundwork for future research applicable to families of genes with quantifiable and comparable phenotypes.

Key words: Machine learning; Regression, Compiled database; Genotype-phenotype relationships; Predicting phenotypes; Spectral sensitivity; Color-vision; Opsins; Imputation

Key Points

• We introduce the Visual Physiology Opsin Database (VPOD_1.0), which includes 864 unique animal opsin genotypes and corresponding λ_{max} phenotypes from 73 separate publications.

• We demonstrate that regression-based ML models can reliably predict λmax from gene sequence alone, predict non-additive effects of mutations on function, and identify functionally critical amino acid sites.

• We provide an approach that lays the groundwork for future robust exploration of molecular-evolutionary patterns governing phenotype, with potential broader applications to any family of genes with quantifiable and comparable phenotypes.

Introduction

Although critical to progress in drug and vaccine design [1–3], responses to climate change [4–8], and bioengineering [4,9–11], accurately predicting gene function from sequences remains a significant challenge. While there are many ways to elucidate genotype-phenotype relationships experimentally, including deep mutational scanning, and in-vitro heterologous expression with phenotyping, these techniques are often tedious and cost-prohibitive, especially when applied to broad comparative studies of gene families. In addition, accurately predicting the phenotype of a protein using computational methods alone is challenging because of data gaps and the sheer complexity of possible relationships between genes and phenotypes, including epistasis and the non-additive effects of different mutations. Machine learning (ML) is gaining traction for its potential broad biological applications, accessibility, and faster speeds, especially in biological contexts where phenotype data are abundant and quantifiable. Here, classical regression and classification algorithms are sometimes used to train models for phenotype predictions using genotype-phenotype data [12,13], while deep learning models can be used to integrate heterogeneous multi-layered omics and environmental data for establishing higher dimensional genotype-phenotype connections [14,15] or *de-novo* protein design [16]. In broader biological contexts, ML models often inform laboratory experiments to predict directional evolution of diseases and their variants [17–19] or to automate image sorting and animal identification from camera trap data [20–22]. In all cases, ML models are a worthwhile long-term investment for genotype-phenotype studies because models can iteratively improve as empirical data accumulate over time.

Such accumulation of important information is exemplified by decades of laboratory work that has led to significant progress in understanding the genetic basis of phenotypic changes for model gene families such as opsins. Opsins are a family of G-protein Coupled Receptors (GPCR) which bind to a retinal chromophore. The two units together, opsin and chromophore, form visual pigments which absorb photons [23]. Opsins have crucial roles in many organismal functions, including circadian rhythms, phototaxis, and image-forming color vision. A critical opsin phenotype is spectral sensitivity - the range of wavelengths to which a gene or organism is sensitive. The main parameter of opsin spectral sensitivity is λ_{max}, the wavelength of light (in nm) with maximal absorbance [24]. Common methods of characterizing spectral sensitivities and λ_{max} include organ-level electroretinograms (ERG) [25–27], cell-level microspectrophotometry (MSP) [28–32], purification of heterologously expressed opsins followed by spectrophotometry [33], and heterologous action spectroscopy using light response assays for opsins expressed in immortalized cell lines [34]. Different opsins are tuned by changes in amino acid sequences to respond to different wavelengths of light, and many previous studies have expressed experimentally mutated opsins and measured spectral sensitivities to establish genotype-phenotype connections [34–38]. Although other factors sometimes affect spectral responsiveness, including the type of chromophore to which an opsin is covalently bound (11-cis retinal or 11-cis-3,4-didehydro retinal) [39,40], opsins provide a rare case where an intrinsic molecular function extends rather directly to organismal phenotypes, especially those involving

color sensitivity. Despite opsins being a well-studied system with an extensive backlog of published literature, some previous authors expressed doubts that sequence data alone could provide reliable computational predictions of λ_{max} phenotypes [41–44]. At the same time, some λ_{max} predictions showed promise, although on the limited scale of vertebrate cone visual pigments via atomistic molecular simulations [45,46]. Furthermore, only the non-animal, microbial, or Type-1 (T1) opsins have been systematically cataloged and used to examine genotype-phenotype predictive power of ML models [47,48]. While some researchers have made significant efforts to compile peak sensitivity data for terrestrial animal photopigments [49], and taxon-specific light-sensitivity data for groups like frogs [50,51] and ray-finned fishes [52,53], these efforts currently lack direct links to genetic data that are essential for our current study. Consequently, the extensive data on genotype-phenotype associations of animal opsins remains disorganized, decentralized, often in non-computer readable formats within older literature, and under-analyzed computationally.

Here, we report a genotype-phenotype database for animal opsins called the Visual Physiology Opsin Database (*VPOD*). We used standard literature searches to compile all heterologously expressed animal opsin genes with spectral sensitivity measurements. We used this newly compiled and harmonized database to evaluate ML methods for connecting genotypes and phenotypes. We created eleven subsets of the overall database to examine factors that impact the reliability and performance of ML models and briefly compared ML predictions to phylogenetic imputation [54,55]. We also examined whether ML can predict intragenic epistasis, and we predicted amino acid sites particularly important for changing λ_{max}. Using our database of 864 unique opsin sequences and corresponding λ_{max} values, we show ML models trained on opsin data accurately predict the λ_{max} of opsins from genetic data alone [highest R^2 =0.968 with a lowest mean absolute error (MAE) of 6.56 nm], especially when ample and diverse training data are available. ML also predicts some known effects of epistatic mutations on λ_{max}. Finally, ML models identify several sites that cause shifts in λ_{max} (e.g., 'spectral tuning sites') and sites known to be structurally important, even in the absence of mutant data in training. When training data are sufficient, these results support the use of ML as a reliable and efficient predictor of λ_{max} for previously uncharacterized opsins, as a tool for identifying candidate spectral tuning sites and epistatic interactions, and as a more general method for linking gene sequences and phenotypes.

Methods

Compiling a genotype-phenotype database for animal opsins

We collected λ_{max} data for opsins using typical literature review/search methods, with search engine, keywords, and date of access documented in the '*litsearch*' table of the *VPOD* database. We cataloged all usable papers with λ_{max} data in the '*references*' table of *VPOD*, recording DOI and a key to link to the search that found the paper. We documented the details of heterologous expression experiments in the '*heterologous*' table, including species, GenBank accession number for the sequence, mutation(s) (if applicable) using a machine-readable notation, λ_{max}, cell type for expression (e.g., HEK293, COS1, etc.), protein purification method,

type of spectrum (e.g., dark or difference spectrum), and a key to link to the corresponding literature source. Note, we did not record the chromophore used to reconstitute the purified opsin protein because 11-cis retinal is the standard and all instances thus far recorded in the '*heterologous'* table are from experiments using 11-cis retinal (although future iterations of VPOD could record these details if data with alternative chromophores becomes available). We input opsin genetic data in an *'opsins'* table, recording opsin gene family names (e.g., long-wave sensitive=LWS, short-wave sensitive=SWS1, etc.). We also included specific '*gene names'* (where applicable), phylum, class, species information, accession number, DNA sequence, amino acid sequence, and the database from which sequences were retrieved (e.g., NCBI). We recreated all mutant and chimeric (e.g., one or more transmembrane domains of the mutant copied from a different sequence to replace the original) opsin sequences based on literature descriptions using a pair of Python scripts (*mutagenesis.py* and *chimeras.py*) available on our GitHub (https://github.com/VisualPhysiologyDB/visual-physiology-opsin-db). We added all heterologously expressed opsins from the literature to *VPOD*; we call this version of the database *VPOD_1.0*. We refer to heterologous data as *VPOD_het_1.0*, which will allow for future additions to the database to link specific opsin sequences to λ_{max} values established with methods other than heterologous expression, including microspectrophotometry or other methods. During the course of manuscript review, we found and entered 259 new heterologously expressed opsins into VPOD, an update we call *VPOD_1.1* (Figure 1). We decided to keep results from *VPOD_1.0* in the main text because the new data points did not drastically alter any model performances. We also provide this table of performance metrics for *VPOD_1.1* (Table S1). Therefore, all tests and figures should still be assumed to use *VPOD_1.0* data unless stated otherwise.

Training ML models with *deepBreaks*

We performed all data pre-processing, including data extraction, sequence alignments, and formatting, in the Jupyter notebooks '*opsin_model_wf.ipynb*', available on GitHub. We used two multiple sequence alignment methods, MAFFT [56] and MUSCLE [57], and a version of both alignments with a Gblocks [58] refinement (for a total of four alignments), all set to their default parameters to begin to test the sensitivity of model performance to different alignments. We then trained various ML models employing a custom version of *deepBreaks* [59], an ML tool designed for exploring genotype-phenotype associations. *deepBreaks* takes aligned genotype data (DNA, RNA, Amino Acid) and some measure(s) of corresponding continuous or categorical phenotype data as input to train ML models. deepBreaks uses one-hot encoding to convert amino acid sequences into numerical values. One consequence of this encoding is any amino acids at a given position in the alignment, which are not present at that position in any training data, will be treated equivalently as unseen. For example, cases of only A and V at a highly conserved site in the training set that are presented with a sequence with T at that site will be considered as no A and no V. The models cannot distinguish the input whether it's T or other unseen amino acids at that site. The results produced by *deepBreaks* encompass a compilation of 12 regression ML

models [59], showcasing ten metrics of cross-validation performance (ranked by R^2) and a feature importance report derived from the top-performing models which ranks amino acid positions by their relative importance to each model (from 0.0-1.0, with 1.0 being a site with the highest relative importance) for the phenotype in question (λ_{max}). The metrics used to determine these relative importance scores of each position vary based on the structure and output of the algorithms used for model training. For example, xgboost [60] and LightGBM [61,62] use the number of times a feature appears in a tree as a proxy for importance [59], while AdaBoost [63] and random forest [64,65], use Gini importance, which quantifies a feature's contribution to improving prediction accuracy [59,66,67]. For a more detailed explanation on how position importance scores are calculated for different models, refer to the '*Interpretation'* heading under the methods section of the *deepBreaks* publication [59]. In addition to R^2, *deepBreaks* reports the Mean Absolute Error (MAE), Mean Absolute Percent Error (MAPE), Mean Square Error (MSE), Root Mean Square Error (RMSE) for each of the 12 ML models. We evaluated the performance of algorithms based on their relative ranks to look for patterns in which algorithms performed better for different data subsets and approaches. *deepBreaks* also produces a set of distribution box plots (default is 100) to visualize phenotypes (λ_{max}) associated with a particular amino acid identity at a site of interest, ordered alphabetically.

Understanding model performance using different subsets of the database

We created eleven data subsets with varying levels of taxonomic and gene family inclusivity (Table 1) to test which factors most impact the reliability/performance of ML methods. We used naming conventions that include versioning to improve reproducibility and reliability of individual datasets and models. For example, one subset combines ultraviolet and SWS opsins, which we named *VPOD_uss_het_1.0*. Our convention is to name the subset (in this case USS = 'Ultraviolet and Short-wave Sensitive' opsins); name the source of phenotype data (heterologous = het), and record the version number of the dataset (1.0). We also created subsets for medium- and long-wave sensitive opsins (*VPOD_mls_het_1.0)* and all rod (Rh1) and rod-like (Rh2) opsins (*VPOD_rod_het_1.0*). Other subsets use species taxonomy, one for vertebrates (*VPOD_vert_het_1.0)* and another for invertebrates (*VPOD_inv_het_1.0)*. For taxonomic subsets, we considered all sequences from phylum Chordata as 'vertebrates' and the rest as 'invertebrates'. Another subset excludes all mutant opsin sequences, called 'wild-types' (*VPOD_wt_het_1.0)*. A final named subset is the whole data set (*VPOD_wds_het_1.0)*(Figure 2).

Using various subsets of data, we performed a number of experiments to better understand the performance of ML models in predicting λ_{max}. First, to better understand how training data relate to model performance, R^2 and training data size, we gradually increased the size of training datasets by starting from zero and incrementally adding between 15-50 randomly selected sequences at a time for the WDS, Vertebrate, WT, and Rod subsets separately; repeating the process three times per subset (Table S2). We then analyzed the fit between the size of training data sets (x-axis) and model performance (y-axis), comparing six non-linear models with AIC to find the model that best explains the observed variation (Figure S3). Second, to

understand if ML could predict known phenotypic changes due to experimental mutations, we queried the top performing WT model (which lacks data from artificially mutated sequences) using all experimentally mutated opsins to predict their known phenotypes. We plotted these results using *matplotlib [68]* to visualize characteristics of poorly predicted outliers (e.g., taxonomic bias or sensitivity to mutations which caused large shifts in λ_{max} from the WT) (Figure 3). To test further whether including these mutant data significantly improves predictions of λ_{max}, we used the *VPOD_het_1.1* dataset (Table S1) and a *Wilcoxon Signed-Rank Test* [69,70] to compare distributions of squared error for predictions by the WDS model (contains mutant data) and WT model (no mutant data) on all mutant data (n = 761) and separately comparing only mutants causing the largest phenotypic changes in λ_{max} (>10 nm from the wild-type; n = 346). To accomplish this for the WDS models, we iteratively removed 25 mutant opsins at a time from training data, used the same training algorithm (Gradient Boosted Regressor, GBR), and predicted λ_{max} values of withheld opsins following the completion of model training (withheld opsins are not used as test data during the actual model training), until all mutant opsins were sampled once (this notebooks is available on GitHub as *'vpod_wf_iterate_subsample.ipynb'*. Third, we examined the ability of our models to predict λ_{max} of thirty invertebrate opsins not in *VPOD_1.0* because they are only known from physiological studies (Table S4, Figure S5). Here, we collected data both characterized by single-cell microspectrophotometry (MSP) or electroretinogram methods and with expression localized to cell-type by *in-situ-hybridization* (ISH), to link λ_{max} to a specific opsin (the sequences and metadata can be found in '*msp_erg_raw.txt'* and '*msp_erg_meta.tsv'*, while the resulting predictions can be found under the *'msp_tests'* folder on our GitHub repository). Finally, we directly compared predictive capabilities of models trained on different data subsets by randomly selecting and removing the same 25 wild-type ultraviolet or short-wave sensitive opsins from the training data of the WDS, Vertebrate, WT, and UVS/SWS models before training and querying the model with those same sequences following training (Table S4, Figure S6).

Comparing Machine Learning and Phylogenetic Imputation

We compared performance of ML models to phylogenetic imputation, which estimates phenotypes using phylogenetic information [54,55]. Phylogenetic imputation uses maximum likelihood (we will not abbreviate maximum likelihood as ML to avoid confusion with machine learning), usually assuming Brownian Motion to predict missing phenotypes using a phylogenetic tree, such that more closely related species or sequences have more similar phenotypes. For the phylogeny, we constructed opsin gene trees in phyML [71], assuming the 'WAG' substitution model [72] and a proportion of 0.029 invariable sites, with Gamma as a rate across sites model, and four substitution rate classes. We randomly removed 50 opsin sequences, and their corresponding λ_{max} values from each of the ML training datasets (with the exception of the smaller MWS/LWS and invertebrate datasets, where we only removed 15), then estimated the removed λ_{max} values using phylogenetic imputation. We used the phylogenetic imputation sub-module of the *phytools* R package [73] for imputation. We compared imputed and actual

λ_{max} using regression. Imputation seemed sensitive to input alignment, perhaps caused by very short or zero length branch lengths in the phylogeny, as we could only complete imputation with *phytools* after removing uninformative and heavily gapped regions with Gblocks. To allow direct comparisons of regressions between imputation and ML, we recreated ML training-data alignments using MAFFT, MUSCLE, and Gblocks in the same way as for imputation and predicted λ_{max} for the same sets of sequences as imputation (Table S7).

Testing ability of ML to account for intragenic epistasis

Functional predictions are often misled by epistasis [41], so we tested the ability of our WDS models to predict the effects of epistatic mutations by haphazardly selecting three double mutants with previously demonstrated epistatic effects from training data in which double mutants, each single mutant, and wild type sequence are all characterized by heterologous expression. The three epistatic double mutants are all derived from bovine rhodopsins: D83N_A292S, F261Y_A269T, and A164S_A269T. We removed the double mutants from the training dataset but retained single mutants to test whether the model treats the mutations as additive or epistatic. We hypothesized that the many instances of multi-mutant sequences with epistatic effects in the training set would allow the model to account for both the magnitude and direction of intragenic epistasis. We then ran a separate test where we removed the same double mutants plus their corresponding single mutants to observe whether the WDS model still predicts epistatic effects from wild type data alone. We subsequently repeated this same process for the WT and Vertebrate models (Table S8).

We ran an additional experiment to test the general ability to to predict epistatic interactions between mutations for all available data. Here, we identified all multi-mutants that have phenotype data for each individual component mutation. Next we selected those multi-mutants with non-additive (epistatic) interactions between mutations (which we define as >1 nm difference between the actual multi-mutant phenotype and the sum of changes in phenotype due to the individual mutations). These 111 ‘epistatic mutants’ were then all removed from WDS (*VPOD_wds_het_1.1*) to create a new training data set called ‘WDS-minusepi’ that lacks evidence of intragenic epistasis. For this test we hypothesized that if the ML approach can account for epistasis, the RMSE of predictions of the 111 epistatic mutants would be significantly lower for the model trained with WDS-minusepi than the model trained with no mutants at all (WT). We tested for statistically significant differences in the distributions of square error for predictions made by WDS-minusepi versus WT; WDS-minusepi versus the Epistasis-free Additive Mutation Values (EAMV, which represents the expected λ_{max} for mutants if the effects of their singular mutational components were treated as additive). We also predict a statistically significant difference between predictions made by WT and EAMV only if WT contains enough natural variation (not based on mutants) to observe patterns of intragenic epistasis. These statistical tests assumed a Bonferroni correction for multiple tests.

Identifying known spectral tuning sites

In addition to predicting λ_{max}, we wanted to identify amino acid sites with strong effects on the phenotype, called spectral tuning sites for opsins. To do so, *deepBreaks* produces an 'importance report' of the relative importance of amino acid positions within the sequence relative to the phenotype. This report is generated for each of the top three performing models, with the addition of a column which calculates the 'mean relative importance' value of each individual position. We automated the translation of these feature representations of aligned amino acid positions compared to bovine rhodopsin for the sake of interpretability. We also included the amino acid residue identity at each corresponding position, and whether it is in one of the opsin transmembrane domains (TMD). We used this to provide us with a standardized context for analysis of the most significant positions highlighted by the models, which we could use to compare to published mutants and known spectral tuning sites. We analyzed the importance report for each model to see what positions it highlighted as most important, with an extra emphasis placed on the output for the WT models since it was the least likely to be biased by the presence of already-known mutant data (Table S9), as previous researchers often chose suspected tuning sites for mutagenesis experiments.

Results

Data Description: A genotype-phenotype database for animal opsins

VPOD is a new database, available on GitHub and in *GigaDB* [74] that currently includes all heterologously expressed animal opsins. We refer to a subset of the database with only heterologous data as *VPOD_het_1.0*, although for version 1.0, this is synonymous with the entire database. *VPOD_het_1.0* relies on 73 publications, mainly primary sources, with dates ranging from the 1980's to 2023. The database contains opsin sequences and phenotype data from 166 unique species (counting 35 reconstructed ancestors), including fishes, amphibians, reptiles, mammals, crustaceans, and bivalves. Altogether, *VPOD_het_1.0* contains 864 unique opsin sequences and corresponding λ_{max} values. This includes 318 unique WT opsins and 546 unique experimentally mutated opsins (447 from vertebrates and 99 from invertebrates) from 82 species (73 vertebrate and 9 invertebrate species). Of the mutants, 73 are 'chimeric', meaning one or more transmembrane domains of the mutant are copied from a different opsin to replace the original. Phylogenetically, *VPOD_het_1.0* is mainly vertebrate opsins (n = 721), with only 143 unique invertebrate opsins (Figure S10). The vertebrate opsins consist of 113 UVS opsins, 167 SWS opsins, 8 MWS opsins, 83 LWS opsins, 237 Rhodopsin (Rh1), and 113 Rhodopsin-like (Rh2) opsins (Figure S10). Phenotypically, *VPOD_het_1.0* spans a range of λ_{max} values from 350-611 nm. The highest concentration of phenotype values are between 350-375 nm and 475-525 nm (Figure 1), due to the literature bias favoring characterization of UVS/SWS opsins and rhodopsins (Rh1).

The data used for model training strongly impacts accuracy

Several models trained with different subsets of data predicted λ_{max} with high accuracy (Table 1). The top-performing models from these subsets consistently used the same five algorithms, including the Gradient Boosting Regressor (GBR) [67,75], Bayesian Ridge (BR) [76,77], Light Gradient Boosting Machine (LGBM) [78], Random Forest (RF) [65], and Extreme Gradient Boosted Machine (XGB) [60]. For example, *VPOD_vert_het_1.0* - trained with all vertebrate wild-type, mutant, and chimeric opsins - had the highest 10-fold-cross-validation (CV) R^2 (0.968) and lowest mean absolute error (MAE) (6.56 nm) of any models we compared (Figure 2). Similarly, *VPOD_wds_het_1.0*, trained with the whole dataset, had very high R^2 (0.947) and low MAE (7.47 nm). The two data subsets also shared the same five top performing models (GBR, BR, LGBM, RF, and XGB). In addition, *VPOD_wt_het_1.0* - trained without mutants and only wild type data - had a similarly high R^2 (0.902) and a low MAE (10.3 nm) when predicting unseen wild type data. Overall, this 'wild type-only' model also fared well, even when predicting mutant data not included in the model (Figure 3). While these performance metrics are impressive, it is important to remember that phylogenetic relatedness between sequences of a dataset could inflate values, like R^2 , when using random sampling for cross-validation because opsins that are more similar to those in the training data will be easier to predict, and phylogenetically clustered sequences will also be more likely to be resampled. Roberts et al. [79] provide a discussion of alternative cross-validation strategies such as "block cross-validation" for non-independent data types, including phylogenetically related data which can help mitigate this issue. Despite overall high R^2, we noticed multiple instances where mutations that cause large shifts in λ_{max} (>10 nm) were not well-predicted by the wildtype-only model, as indicated by large residual values for the predictions of these mutant sequences (Figure 3). We found including mutant data significantly improves predictions of λ_{max} when comparing predictions of models trained with (WDS) and without (WT) mutant data and rejecting the null hypotheses of no underlying differences between the distribution of squared error for predictions of all mutants (p-value = 9.96e-22, WDS Root Mean Square Error (RMSE) = 12.6nm, WT RMSE = 17.6nm)(Figure S11) and when predicting phenotypes of mutants with large shifts in λ_{max} (p-value = 2.29e-25, WDS Root Mean Square Error (RMSE) = 17.0nm, WT RMSE = 24.2nm)(Figure S11).

In addition to including mutant data, data availability more generally improves predictive power, with performance thresholds and plateaus depending on the genetic diversity of the training data. Overall accuracy in predicting λ_{max} for our models trained on more genotypically and phenotypically complete subsets of data (WDS, Vertebrate, WT) improves as a function of the number of sequences in a dataset, and shows an initial plateau (R^2 = ~0.80-0.90) of diminishing returns around 120-200 sequences that continues to taper off above 200 sequences (Table S2, Figure S3). Consistent with a rough performance threshold, we found models from data subsets with less than ~200 training sequences to far less accurately predict λ_{max}. For example, *VPOD_mls_het_1.0* – trained only on the 91 MWS/LWS opsins of vertebrates – and *VPOD_inv_het_1.0* – trained only on 144 invertebrate opsins - showed among the lowest R^2

(0.677 and 0.814 respectively; Table 1). For all data subsets, we found the relationship between number of sequences in a dataset and model performance best fits a reciprocal model, which is suitable when the dependent variable plateaus as the independent variable grows larger. We found the coefficients of the reciprocal equations to be different between data subsets and to increase in negative magnitude with a decrease in taxonomic/genetic diversity (the Rod model holding the largest negative value of -44). These equations do not account directly for taxonomic, genetic, or phenotypic diversity, as the raw number of genes is the value of the x-axis. Therefore, one should be cautious about applying them to predict model performance based on training data size alone.

The complicated relationship between size of training dataset and predictive power is further illustrated by models from some larger data subsets that resulted in rather poor predictions. One large dataset (884 sequences), the previously published Karyasuyama type 1 opsin dataset (*Karyasuyama_T1_ops [47]*) showed only moderate R^2 (0.804) and MAE (9.41), similar to models from the much smaller invertebrate data (Table 1). One explanation for lower predictive power could be that the very old age of T1 opsins led to a higher complexity and diversity of genotype-phenotype associations, which are not yet completely sampled enough to allow good predictions. In addition, models based on Rod, UVS/SWS, and MWS/LWS subsets tend to show lower R^2 than might be at first expected (Table S2, Figure S3), especially since these three datasets together comprise the training data for the vertebrate model (our highest performing model, $R^2 = 0.968$). For example, the Rod model, with 352 sequences, should have resulted in a model with an R^2 around 0.900-0.960 based on the trendlines for the WDS and Vertebrate datasets (Table S2, Figure S3) but resulted in an $R^2 = 0.831$. A possible explanation for this lower R^2 value for Rod models is the small degree of variability in $\lambda_{max.}$ When variation is low, even very small differences from model predictions could lead to larger differeces in R^2. Therefore, when a data subset such as Rod opsins contains limited variability in the response variable (λ_{max}), additional metrics that are less sensitive to variance will be important, such as MAE or RMSE, which report the absolute magnitude of errors rather than the proportion of explained variance. To illustrate further, most models tested on their ability to predict the λ_{max} for a set of 25 subsampled WT-SWS opsins from VPOD, performed relatively poorly based on R^2 alone (Table S4); with the Vertebrate model ($R^2 = 0.914$, MAE = 7.89) demonstrating a relatively greater predictive power than all other models (Table S4, Figure S6). However, between the Vertebrate and lowest performing model (SWS model; $R^2 = 0.778$, MAE = 11.6nm), there is only a 3.71 nm increase in MAE; a much less dramatic perceived shift in performance than might be interpreted from R^2 alone.

Table 1. Performance Metrics Across Opsin Subsets and Top Performing Models.

Name	Data Subset Version	# Seqs	Top ML Algorithm	[b]R^2	[a]MAE [nm]	[a]MAPE [%]	[b]MSE	[b]RMSE
Whole Dataset	*VPOD_wds_het_1.0*	864	LGBM	0.947	7.47	1.71	207	13.8
All Wild Types	*VPOD_wt_het_1.0*	318	Bayesian Ridge	0.902	10	2.18	297	16.5
All Mutants	*VPOD_mut_het_1.0*	546	LGBM	0.951	7.89	1.86	194	13.4
Vertebrates	*VPOD_vert_het_1.0*	721	LGBM	0.968	6.56	1.49	111	10.3
WT Vertebrates	*VPOD_wt_vert_het_1.0*	274	GBR	0.961	5.46	1.18	82.1	8.36
Invertebrates	*VPOD_inv_het_1.0*	143	LGBM	0.814	14.7	3.22	614	23.1
Rods	*VPOD_rod_het_1.0*	352	Bayesian Ridge	0.834	3.51	0.71	27.7	5.04
WT Rods	*VPOD_wt_rod_het_1.0*	157	GBR	0.783	3.57	0.72	31.9	5.11
MWS/LWS	*VPOD_mls_het_1.0*	91	XGB	0.677	8.77	1.82	317	15
UVS/SWS	*VPOD_uss_het_1.0*	280	GBR	0.821	8.02	2.06	200	13.6
WT UVS/SWS	*VPOD_wt_uss_het_1.0*	66	Adaboost	0.865	7.79	1.87	152	10.6
T1 Opsins	*Karyasuyama_T1_ops*	884	Random Forest	0.804	9.41	1.76	186	13.5

[a]*Mean absolute error* (MAE) and *mean absolute percent error* (MAPE) are in relation to the absolute error λ_{max} predictions and interpreted in the same units of 'nm'. [b]R^2, *mean square error* (MSE) or *root mean square error* (RMSE) are often interpreted as direct measures of comparing/analyzing model performance and used as training loss terms of the objective function - which measures how well the model fits the training data. One has to often balance between this and the regularization term, which controls the complexity of the model. Thus, a high performance is both simple and predictive; a tradeoff referred to as the '*bias-variance'* tradeoff.

When predicting λ_{max} of thirty unseen wild-type invertebrate opsins from a separately curated MSP dataset, almost every model performed rather poorly, with exception of the WT model (n = 30, R^2 = 0.887, MAE = 17.5) (Table S4, Figure S5). The best performing model produced by the sparsely populated *'Invertebrate'* dataset could only predict unseen invertebrate opsins with an R^2 of 0.837 and MAE of 26.3 nm (Table S4, Figure S6). Until the models are trained with more invertebrate (r-opsin) data, we would not put high confidence in the estimates of λ_{max}. Furthermore, these separately curated invertebrate opsins are independent of the phylogenetic relatedness of the data used in model training, and therefore provide a less inflated estimate of the ability to predict λ_{max} compared to random resampling of training data. Because of the sparsity of invertebrate data in the training set, this result further highlights that opsins more distantly related to those in the database will be more difficult to predict.

ML predictions of λ_{max} are comparable to phylogenetic imputation

Both ML and phylogenetic imputation were often accurate predictors of λ_{max} (Table S7). When using the same test data, ML models usually outperformed phylogenetic imputation, however slightly (Table S7), albeit using far less computational time: ML used on the order of minutes to calculate models and imputation used on the order of hours to generate opsin phylogenies. The MWS/LWS dataset was the only instance where phylogenetic imputation (R^2 = 0.784) largely outperformed ML (R^2 = 0.512). We found our implementation protocol for phylogenetic imputation required removing aligned sites with extensive gaps (for which we used Gblocks); we speculate this lessened the impacts of very short branch lengths on model fitting during imputation. To allow direct comparisons between approaches, we also used the same trimmed alignments for training ML models. Interestingly, there was a slight but noticeable decrease in ML performance following Gblocks trimming for the Invertebrate, MWS/LWS, and UVS/SWS datasets (Table S7). The R^2 of the MWS/LWS model dropped from 0.677 to 0.645, while the Invertebrate model dropped from 0.814 to 0.797 (Table S7). ML performance remained relatively consistent after tripping for the WT, Vertebrate, WDS, SWS/UVS, and Rod models, with only a slight reduction in R^2 (< 0.01) and slight increase in MAE (+/-1nm) for the WT model. We speculate the observed differences in ML performance following Gblocks processing is due to the reduced number of features in the datasets from removing aligned sites.

ML often predicts the effects of epistatic mutations

The WDS successfully predicted three out of three individual instances of epistasis (Table S8) using sequences that were removed from the training data before using the model to predict known epistatic phenotypes. For double mutant D83N_A292S, the model predicted 485.2 nm, which was 0.2 nm off the known λ_{max} of 485 nm. If the WDS model believed the sites were additive, the resulting λ_{max} based on adding shifts of single mutants would have been much lower, at 477.5 nm. Second, for mutant F261Y_A269, the model predicted 520.0 nm, for which the known λ_{max} was 520 nm. An additive prediction would have been higher, 524 nm. Third, for mutant A164S_A269T the model predicted a λ_{max} of 515.5 nm, where the known λ_{max} was 514 nm. This is a special case in which the double mutant experiences a form of epistasis where the effect of mutation A269T (λ_{max} = 514) masks the shift otherwise caused by mutation A164S (λ_{max} = 502 nm). Thus, the model correctly predicted an instance of epistasis in which one mutation masks the effect of another.

We also queried the WT model with these same three double mutants to test the importance of mutant sequences in informing the model on epistatic interactions. However, without any mutant data at all, the WT model did not display the same abilities to predict epistasis in any instance. For the double mutant D83N_A292S, the model predicted neither the individual mutations nor the double mutant would have a significant effect on λ_{max}, and all were predicted to be 499.9 nm. For double mutants F261Y_A269 and A164S_A269T, the WT model successfully predicted all individual mutations would cause a red shift (although F261Y and

A269 were >3 nm off their known λ_{max}), but incorrectly treated the mutational effects as additive for the double mutant (Table S8).

Our broader experiment to test the predictability of epistatic effects using the WDS-minusepi model (which excluded from training all 111 opsins with known non-additive mutational effects, which we call epistatic opsins) correctly predicted epistasis for 105/111 of the epistatic opsins with higher R^2 (0.969) and much lower RMSE (12.4 nm) than predictions by the WT model ($R^2 = 0.894$, RMSE = 22.3 nm), which contains no experimentally mutated opsins, and the EAMV ($R^2 = 0.878$, RMSE = 29.8nm), which ignores epistatic effects, respectively (Figure S12). Our test of the null hypotheses of no underlying differences between the distribution of squared error for predictions of the 111 epistatic mutants were rejected after Bonferroni correction by the WDS-minusepi model versus WT model (p-value = 1.24e-06); WDS-minusepi model versus EAMV (p-value = 2.56e-09), but not rejected for the WT model versus EAMV (p-value = 0.086) (Figure S12). Together, the large differences in RMSE and the results of the statistical tests strongly support the idea that the inclusion of even single mutants significantly reduces the error of ML models when predicting epistatic interactions between mutations and that this error is also less than the error we would observe if our models simply treated mutations as additive. Nevertheless, the insignificant difference between WT predictions and EAMV indicate there is not enough information about epistatic interactions in wild type (non-mutant) data alone to accurately predict intragenic epistasis.

ML predicts tuning sites from Wild-Type sequences alone

The full WT model and its few variants (SWS and Rod WT models) predict several previously characterized 'spectral tuning sites' - functionally demonstrated to change λ_{max} - even with no information on mutants used in the training data (Figure 4, Table S9). For the primary WT model alone, we found 15 of the top 25 amino acid sites, ranked by relative importance to the model (all ≥ 0.40), were spectral tuning sites previously characterized by mutagenesis and heterologous expression (Table S9). For example, the especially well-characterized position 308 (p308), known for its role in tuning LWS opsins, and considered to be one of the five key sites in characterizing LWS opsins under the 'Five-Site Rule' [80], had the highest relative importance value of 1.0 when using the full WT model, indicating the amino acid identity at p308 is especially important for predicting λ_{max}. In another example, the full WT model highlighted p181, a phylogenetically conserved counterion in the retinal-opsin Schiff base interaction for all non-vertebrate opsins [81,82]. Additionally, the transition from E to H at p181 (E181H) is a characteristic of the red-shifted vertebrate LWS opsins [35,82], easily visualized in Figure 4C. When predicting λ_{max} of bovine rhodopsin with mutation E181H, the WT model predicted a red-shift compared to wild type, as observed with the natural evolution of the LWS opsin lineage. The WT SWS/UVS model similarly highlighted p113, a site functionally characterized as the counterion in the retinal-opsin Schiff base

interaction for all vertebrate opsins [35,82] and as a known spectral tuning site in SWS/UVS opsins [83]. Moreover, even the WT Rod model, trained on a mere 157 sequences, identified p292 (Table S9), another well-characterized and conserved spectral tuning site for vertebrate rhodopsins [84–86], as the site with highest relative importance to its predictions of rhodopsin λ_{max}. These spectral tuning sites are not simply conserved sites, as there is little to no correlation between amino acid sites important to model predictions (importance scores) and their relative *Shannon Entropy* [87,88] scores ($R^2 = 0.001$). This is somewhat expected as *deepBreaks* drops all conserved ('zero-entropy') sites during preprocessing, because a site with no variation provides no important information to the effects of variation on the resulting phenotype. In addition, we predict any correlation between site conservation and model importance would be for sites that are moderately conserved and in close proximity to opsin-chromophore binding site (position 296) or binding pocket [41,42,89].

Discussion

To better understand methods to connect genes and their functions, we initiate *VPOD,* a database of opsin genes and corresponding spectral sensitivity phenotypes. Here, we used *VPOD_1.0* to examine the ability of ML models to predict functions of opsin genes, predict intragenic epistasis, and identify amino acid sites critical for functional changes. In all cases, ML shows promise, especially when given enough training data.

The important relationship between data availability and predictive power

The predictive power of λ_{max} is often high when using ML for opsins, and it improves with a greater amount and variety of data, albeit with diminishing returns. In particular, the number of opsin genes, their genetic diversity, and the relationship between genetic and phenotypic differences are all critical in determining predictive power. Particularly illustrative of these ideas are our analyses with and without experimentally mutated opsins. Even though we might conceive of all wild type data as natural mutants chosen by evolution, experimentally induced mutations are particularly important by often changing just one amino acid that drastically changes phenotype. As such, we found that including mutant data usually improved predictive power and conversely, predicting some phenotypes from laboratory mutagenesis was sometimes difficult without including other mutant data in model training (Figure S11). However, relying on published mutant data alone is not optimal because it is derived from a non-random subset of species because people continue to work in established systems. Nevertheless, the genotype-phenotype landscape may be sampled well enough using high numbers of only wild type genes, as evidenced by the small difference in performance when adding mutant data to the wild type subset of well-sampled vertebrate opsins (Table 1). In contrast, adding mutant data to the sparsely sampled invertebrate opsins made a big difference. For invertebrate opsins, using only wild type data (ignoring all mutants) led to some very inaccurate predictions, especially of large phenotypic shifts caused by experimental mutagenesis (Figure 3), indicating the genotype-phenotype space is still undersampled for invertebrates. This is expected since ML

learns from patterns in the underlying dataset, making predictions of distantly related opsins from those in *VPOD* more unreliable. We acknowledge this as a significant drawback for the ML approach, especially in systems or taxonomic groups lacking sufficient or reliable data. Thus, given this currently limited dataset we do not put high confidence in the λ_{max} estimates of either wild-type or mutant invertebrate (rhabdomeric) opsins. Therefore, targeting invertebrate opsins should be a high priority for new additions to *VPOD*.

A large diversity of training data is also critical for reliably predicting intragenic epistasis – the non-additive effects on a phenotype of interactions between two or more mutations within a gene – which is common [10,41,43,44,91,92] and an obstacle to connecting genotypes and phenotypes [41,93–95]. Our most complete datasets (whole dataset and vertebrate dataset) identified known cases of intragenic epistasis, but our models trained without experimental mutagenesis data did not. Moreover, ML demonstrates some capacity to predict the epistatic interactions between mutations, even when only provided with the single mutation components - as is evidenced by our WDS-minusepi dataset test (Figure S12). Similarly to the overall predictive power of λ_{max} above, predicting epistasis probably requires sufficient variation at interacting sites, which seems especially enhanced by experimentally mutated genes.

Variation in the availability of genotype-phenotype data for training not only impacts predictive power of phenotype, but also the converse; the ability to predict amino acid sites that change λ_{max}. Several models, including those trained with the whole data set (WDS), Vertebrate, and wild type (WT) data were able to successfully predict previously characterized spectral tuning sites. This is less surprising for models trained with WDS and Vertebrate datasets, due to the prevalence of data, even including mutants in the training data from experiments which specifically targeted sites thought by researchers to be functionally informative. Yet even without any targeted mutational data, three model variants using only wild type data predicted experimentally well-characterized spectral tuning/functional sites, including sites important to the stability of the opsin-chromophore interaction (P181 and P113). This demonstrates the strong potential for ML models to identify amino acid sites that govern phenotype, leading to predictions of candidate spectral tuning sites, which can be confirmed with mutagenesis experiments [38,85] if not done so already.

ML algorithm type contributes to the predictive power of ML models.

While probably not as important as the training data used, the ML algorithm itself also impacts predictive power. All five of the best performing ML algorithms (GBR, BR, LGBM, RF, and XGB) are variants of the decision tree model architecture (Table S13), and three out of five, including GBR, LGBM, and XGB, are 'gradient boosted' decision tree based ML algorithms. The gradient boosted algorithms all share the same general principles of gradient boosting [75,96] including the use of ensembles of 'weak learners', usually decision trees, which work sequentially and 'gradient descent' when minimizing a loss function, to improve ML model performance. While LGBM generally performed best for predicting phenotype, it was not as effective in predicting the epistatic effects of mutations, where GBR and XGB showed the

highest performance. This suggests that while LGBM excels in general phenotype prediction, the details of GBR and XGB may be better suited for epistasis prediction. The difference likely arises from the unique aspects of each algorithm's model training and settings of hyperparameters. XGB and LGBM differ from GBR by the addition of a regularization term to the objective function and in the process of ensemble tree construction during model training: GBR and XGB use level-based tree fitting while LGBM uses leaf-based tree-fitting. One consequence of leaf-based tree construction is that due to its faster convergence/training time, it can create complex trees that are more prone to overfitting, thereby 'learning' patterns which may not exist as it constructs trees on a 'best-first basis' with a fixed number of n-terminal nodes [62,78]. This creates a model that often performs well on training data but may overgeneralize, missing finer grained collinearities and interdependencies, which would be important for predicting epistasis. As such, our models might be improved by fine-tuning hyperparameters (e.g., learning rate, max-depth, and number of estimators), and the choice of which model to use will depend on the end goals of the analysis.

The assumptions of our method and limitations of ML extrapolation.

Understanding the limitations and assumptions inherent in predictive modeling is vital for accurately interpreting animal color sensitivity from opsin sequences, especially considering the impact of various factors on sensitivity beyond the opsin itself across multiple levels of biological organization. At the cell level, we assume that λ_{max} measured in cell culture (e.g., HEK293, COS cells) is the same as in living photoreceptor cells. We also assume the photopigment uses 11-cis-retinal, as all heterologously expressed opsins in *VPOD* were reconstituted using this chromophore. However, this assumption is violated in some organisms because they use 13-cis-retinal as the *in-vivo* chromophore [23,97,98], which is associated with a red-shift in λ_{max} [35,97]. At the organ-level, filters such as oil droplets in bird eyes [99–102], pigments in butterfly eyes [103], or a combination of transmissive filter and narrow band reflector in mantis shrimp larval eyes [104], each may selectively influence light reaching photoreceptor cells and therefore animal color sensitivity. Finally, organismal responses to light involve neural processes, so even if an organism possesses the physiological ability to detect certain wavelengths, it still may not have a use for that ability. Similar considerations for all these assumptions will apply when using ML to infer other functions from other genes. In fact, many genes are more susceptible than opsins (but see [105] showing the pressure of ocean depth may slightly affect λ_{max} phenotypes) to changes in pH, temperature, and other environmental factors [106], such that databases compiling these gene functions should also record these parameters for use in training ML models.

Perhaps the most important caveat of using ML models to accurately predict phenotype or functional sites is that we assume there is a genotype-phenotype association that we can fit to a function and that our models were trained using ample data to capture these associations. Based on the non-linear fit between size of training data set, and model performance, we estimate that including about 200 sequences (and corresponding λ_{max}) from a taxonomically and

phenotypically diverse range still provides improvements to model performance. Above 200 sequences, there is still improvement, but at a diminishing rate consistent with a reciprocal model (Table S2, Figure S3). That said, we encourage caution when extrapolating these results to predict model performance on training data size alone as the equations we used do not account directly for taxonomic, genetic, or phenotypic diversity. When using ML for predicting functionally important sites, the addition of experimental mutants to training data that cause large phenotypic changes could heavily bias which sites are selected as 'most important' and potentially mask the importance of other sites. Here again, providing a diverse set of genotype-phenotype data should allow for the discovery of new functional sites, even when including known mutants in the training data with large phenotypic effects. Additionally, providing a large number of mutations from a limited breadth of taxa can bias model predictions as not all mutations will have the same effect on different sequences, especially if they are genetically distant. This makes it all-the-more important to consider the level of genetic diversity used to train a model when extrapolating to find potentially important functional sites (i.e., if identifying tuning sites for rhodopsins, then using a dataset of only rhodopsins would likely be the best approach, but if data is sparse or if looking for sites that may largely impact spectral tuning across opsin subfamilies, a genetically and phenotypically broad dataset may be better).

Conclusion

Using opsin sequence data with *deepBreaks*, we were able to train regression-based ML models to reliably predict λ_{max}, often accounting for non-additive effects of mutations on function (intragenic-epistasis), and identifying amino acid sites critical for function. We expect future work will improve these already promising results even further through at least two general directions. First, adding more data to *VPOD* will improve results, especially adding invertebrate (rhabdomeric opsins) data, as technical knowledge improves for expressing these genes [34]. In addition, phenotypic data – besides the in-vitro heterologous expression targeted here – is expansive, including λ_{max} measurements from microspectrophotometry and electroretinograms, but will take considerable effort to link these phenotypes to specific opsin genes. Second, our models can be improved to take advantage of more information. One important addition should be inclusion of physicochemical properties of the amino acids [107], as implemented with success on a small scale of only 26 amino acid positions of microbial opsins to predict red-shifted phenotypes for optogenetics [108]. Additionally, information on protein structure could be particularly important, such as the distance of an amino acid from the binding pocket of the chromophore [40]. While there are only a few solved crystal structures for opsins [109,110] to provide such data, indirect techniques like homology modeling [111] or neural network-based structural prediction [112] might be usable. Other information about opsins could also be predictive, such as which G-protein the opsin signals to, allowing prediction of which amino acids dictate G-protein specificity. Opsin kinetics [e.g. 113], or even the habitat depth at which the animal lives in the ocean, which not only influences light environment but also alters which amino acids are used in opsins [114], could improve predictive power of the ML models. Finally, we once again caution against treating predictions of λ_{max} uncritically,

because the quantity and quality of genotype-phenotype data used to train a model -- including the taxonomic, genetic, and phenotypic diversity -- is integral to the reliability of a model's predictions. Thus, ML models like those used here can be considered tools to make predictions based on summaries of existing knowledge, thereby complementing traditional experimental methods.

Potential Implications

Given the high performance demonstrated in this paper, current models are already robust enough to allow several applications. First, predicting λ_{max} will often be useful, especially for vertebrate opsins. For example, ML could provide an estimate of λ_{max} in a hogfish, whose skin expresses an opsin with unknown absorption and where λ_{max} has implications for a conceptual model of chromatophore expansion [115]. Second, estimates of λ_{max} from opsin sequences formed part of an argument that changes in gene expression, not sequence, adapted Amazon fishes to local light environments [116]. On broader taxonomic scales, predictions of λ_{max} from opsin sequences could expand studies of adaptation, molecular, evolution and constraint in comparison to light environments [117]. Another application could be protein design for optogenetics - the use of genetic light sensors to induce and study expression or response pathways [118–120] - including those associated with embryogenesis [121,122], stress and depression [123–125], or neuronal diseases [126,127]. Finally, our models could be used to simulate molecular evolution under a realistic genotype-phenotype landscape. One shortcoming presently for such simulations is that our models are not trained with non-functional opsins, so even non-functional genes would be predicted to have functional λ_{max} values. A solution could be to add large-scale mutagenesis data to the training set, such as that from deep mutational scanning [128], although the authors indicated the method is only in a proof-of-concept stage, such that the results are too noisy to be useful for model training. As the *VPOD* database expands, there will be many applications for ML, and similar techniques can also be applied to other gene families such as luciferases [16,129,130].

Availability of Supporting Source Code and Requirements

Project name: The Visual Physiology Opsin Database (VPOD)
Project home page: https://github.com/VisualPhysiologyDB/visual-physiology-opsin-db
DOI: VPOD_1.0 - 10.5281/zenodo.10667840 // VPOD_1.1 - 10.5281/zenodo.12213246
Docker image of the latest version of the deepBreaks:
https://hub.docker.com/repository/docker/omicseye/deepbreaks-dc/general.
Operating system(s): Windows, MacOS, and Linux
Programming language: Python, R
Other requirements: Conda 4.9.2, deepBreaks 1.1.2, GBlocks 0.91b, MAFFT 7.520-1, MUSCLE 3.8.31, mySQL workbench 8.0.36, Python 3.9, RStudio 2023.06.2+562
License: GNU General Public License (GPL) - Version 3, 29 June 2007

Figure Legends

Figure 1. Histogram distributions of Vertebrate and Invertebrate Opsins and Absorbance Data - λmax - from *VPOD_het_1.1* with a scaled Kernel Density Estimate (KDE) curves overlaid to better visualize the general shape and characteristics of our λ_{max} distributions. Note an obvious data bias for vertebrate opsins, especially those with λ_{max} values between 350-375nm and 480-510 nm, probably due to focal research on UVS and Rh1 opsins.

Figure 2. ML model predictions on whole Vertebrate opsin dataset, n = 721, R^2 = 0.968, MAE = 6.68nm, MAPE = 1.52. Sequences were iteratively and randomly selected to be withheld from the training dataset (n=50) to act as unseen test data. This was repeated until all sequences had been sampled once. Predictions in which the absolute difference between the 'known' and 'predicted' λ_{max} are <10nm are represented by gray dots. All predictions in which the absolute difference between the 'known' and 'predicted' λ_{max} are >10nm are represented by colored dots. Yellow dots represent WT predictions, mutants with only a single mutation are green, mutants with greater than one mutation are light-blue, and chimeric opsins are dark-blue. The light-gray bar surrounding the trend-line represents a 95% confidence interval. Inset: Box-plot distribution of prediction error for different opsin data-types from the top performing Vertebrate opsin ML model to better visualize our sources of error. Note, the median for each box-plot hovers around 0nm. Single mutations have the largest spread of error, but this is most likely due to the high abundance of that data-type over all others.

Figure 3. Scatter-plot of Wild-Type Model's λ_{max} predictions for 546 mutant opsins, with an R^2 of 0.860, MAE of 12.36 nm, and MAPE of 2.91%. Mutant predictions in which the absolute difference between the 'known' and 'predicted' λ_{max} are <10nm are represented by gray dots. All predictions in which the absolute difference between the 'known' and 'predicted' λ_{max} are >10nm are represented by colored symbols, further separated by Invertebrate (Squares) and Vertebrate (Circles) opsins. Mutants with only a single mutation are green, mutants with greater than one mutation are light-blue, and chimeric opsins are dark-blue. Mutations which caused a shift of >10nm from the WT are outlined in purple. The light-gray bar surrounding the trend-line represents a 95% confidence interval.

Figure 4. (**A/B**) Blue bars indicate the seven transmembrane domain regions of the bovine rhodopsin and are labeled accordingly. Purple bars indicate the top three most important positions to predictions of λ_{max} by the 'BayesianRidge' ML Regression Model trained on the WT opsin dataset. (**A)** Bar graph of relative entropy scores by position calculated via Shannon Entropy [70,87,88] using the multi-sequence alignment for the WT data subset. (**B**) Bar graph of relative importance by position generated via 'BayesianRidge' ML Regression Model trained on

the WT opsin dataset. We interpret positions with higher relative importance as having a larger effect or weight on λ_{max} prediction. Positions 181 [35,82], 261 [86,90] and 308 [80] are highlighted in purple because they are among the highest scoring sites and have all been previously characterized as functionally important to opsin phenotype and function. Based on an R^2 of 0.001, there is no linear relationship between relative entropy by position and the relative importance of scores by position. (**C, D, & E**) These distribution box plots provide a visualization for which amino acid (aa) residues at a particular site are associated with different ranges of lambda max at a site of interest, ordered alphabetically, not by frequency (left to right). For a more detailed explanation on how position importance scores are calculated for different models, refer to the 'Interpretation' heading under the methods section of the deepBreaks publication [59]

Data Availability

The data set(s) supporting the results and all other code used in this article are available in the '*Visual Physiology Opsin Database*' GitHub repository (10.5281/zenodo.12213246), and at the '*Visual Physiology Opsin Database'* GigaDB repository, [pending acceptance] . All data and code is covered under a GNU General Public License (Version 3), in accordance with Open Source Initiative (OSI)-policies. DOME-ML (Data, Optimisation, Model, and Evaluation in Machine Learning) annotation, supporting the current study, is available through DOME Wizard. The Docker image provided above includes a summary of required package libraries and instructions on how to use it. Along with our existing online materials with tools used, *deepBreaks*, we also have a Jupyter notebook, instructions for Conda installation, and Code Ocean capsule (https://codeocean.com/capsule/9484494/tree/v1) for the deepBreaks. These resources should help practitioners using the main ML program we used, deepBreaks, described elsewhere, use the VPOD database for Opsin applications.

Abbreviations

Adaboost: Adaptive Boosting
AIC: Akaike Information Criterion
COS1: Monkey kidney cell line
CV: Cross-Validation
DNA: Deoxyribonucleic Acid
EAMV: Epistasis-free Additive Mutation Values
ERG: Electroretinogram

GBR: Gradient Boosting Regressor
GPCR: G-Protein Coupled Receptors
HEK293: Human embryonic kidney cell line
ISH: In-situ Hybridization
KDE: Kernel Density Estimate
LGBM: Light Gradient Boosting Machine
LWS: Long-Wave Sensitive
MAE: Mean Absolute Error
MAPE: Mean Absolute Percentage Error
ML: Machine Learning
MSE: Mean Squared Error
MSP: Microspectrophotometry
MWS: Medium Wavelength-Sensitive
NCBI: National Center for Biotechnology Information
nm: Nanometers
RMSE: Root Mean Square Error
RNA: Ribonucleic Acid
SWS: Short-Wave Sensitive
T1: Type-1 {Microbial Opsins}
TMD: Transmembrane Domain
USS: Ultraviolet and Short-wave Sensitive
UVS: Ultraviolet-Sensitive
VPOD: Visual Physiology Opsin Database
WAG: Whelan and Goldman substitution model
WDS: Whole Dataset
WT: Wild-Type
XGB: Extreme Gradient Boosting
λ^{max}: Lambda Max / Wavelength of light with maximal absorbance

Competing Interests

The authors declare they have no competing interests.

Funding

This work was supported by the US National Science Foundation grants DEB-2153773 and IOS-1754770 to THO and DEB-2109688 to AR and KAC. The funders had no role in the study design, data collection and analysis, decision to publish, or preparation of the manuscript.

Authors’ Contributions

THO and KAC conceived the study; SAF performed the analysis; SAF provided online documents and software. SAF and THO drafted the original manuscript. All co-authors discussed the results and edited the final manuscript.

Acknowledgments

We acknowledge funding from the National Science Foundation DEB-2153773 and IOS-1754770 to THO and DEB-2109688 to KAC and AR. We acknowledge use of computational facilities purchased with funds from the National Science Foundation (CNS-1725797) and administered by the Center for Scientific Computing (CSC). The CSC is supported by the California NanoSystems Institute and the Materials Research Science and Engineering Center (MRSEC; NSF DMR 2308708) at UC Santa Barbara. We acknowledge R. Varney for providing technical support and expertise on the phylogenetic imputation experiments. Thanks to V. Scriven for literature searches and data entry. Thanks to A. Singh and S. Yi for advice. We acknowledge Oakley Lab for comments on an early draft of the manuscript. We thank the reviewers for helpful suggestions to improve the presentation of our work.

Citations

1. Ovsyannikova IG, Poland GA. Vaccinomics: current findings, challenges and novel approaches for vaccine development. *AAPS J*. Springer; 2011; doi: 10.1208/s12248-011-9281-x.

2. Steinbrück L, McHardy AC. Inference of Genotype–Phenotype Relationships in the Antigenic Evolution of Human Influenza A (H3N2) Viruses. *PLoS Comput Biol*. Public Library of Science; 2012; doi: 10.1371/journal.pcbi.1002492.

3. Roberts JP. Single-Cell Analysis Deepens Antibody Discovery. *Genet Eng Biotechnol News*. Mary Ann Liebert Inc; 2020; doi: 10.1089/gen.40.02.09.

4. Cobb JN, DeClerck G, Greenberg A, Clark R, McCouch S. Next-generation phenotyping: requirements and strategies for enhancing our understanding of genotype–phenotype relationships and its relevance to crop improvement. *Theor Appl Genet*. Springer; 2013; doi: 10.1007/s00122-013-2066-0.

5. Chevin L-M, Collins S, Lefèvre F. Phenotypic plasticity and evolutionary demographic responses to climate change: taking theory out to the field. *Funct Ecol*. Wiley; 2013; doi: 10.1111/j.1365-2435.2012.02043.x.

6. Franks SJ, Weber JJ, Aitken SN. Evolutionary and plastic responses to climate change in terrestrial plant populations. *Evol Appl*. Wiley Online Library; 2014; doi: 10.1111/eva.12112.

7. Gienapp P, Teplitsky C, Alho JS, Mills JA, Merilä J. Climate change and evolution: disentangling environmental and genetic responses. *Mol Ecol*. Wiley Online Library; 2008; doi: 10.1111/j.1365-294X.2007.03413.x.

8. Munday PL, Warner RR, Monro K, Pandolfi JM, Marshall DJ. Predicting evolutionary responses to climate change in the sea. *Ecol Lett*. Wiley Online Library; 2013; doi: 10.1111/ele.12185.

9. Singhal A, Simmons M, Lu Z. Text Mining Genotype-Phenotype Relationships from Biomedical Literature for Database Curation and Precision Medicine. *PLoS Comput Biol*. journals.plos.org; 2016; doi: 10.1371/journal.pcbi.1005017.

10. Kemble H, Nghe P, Tenaillon O. Recent insights into the genotype-phenotype relationship from massively parallel genetic assays. *Evol Appl*. Wiley; 2019; doi: 10.1111/eva.12846.

11. Dikicioglu D, Pir P, Oliver SG. Predicting complex phenotype-genotype interactions to enable yeast engineering: Saccharomyces cerevisiae as a model organism and a cell factory. *Biotechnol J*. Wiley; 2013; doi: 10.1002/biot.201300138.

12. Leung MKK, Delong A, Alipanahi B, Frey BJ. Machine Learning in Genomic Medicine: A Review of Computational Problems and Data Sets. *Proc IEEE*. IEEE; 2016; doi: 10.1109/JPROC.2015.2494198.

13. Guzzetta G, Jurman G, Furlanello C. A machine learning pipeline for quantitative phenotype prediction from genotype data. *BMC Bioinformatics*. 2010; doi: 10.1186/1471-2105-11-S8-S3.

14. Lee B, Zhang S, Poleksic A, Xie L. Heterogeneous Multi-Layered Network Model for Omics Data Integration and Analysis. *Front Genet*. 2019; doi: 10.3389/fgene.2019.01381.

15. Lee Y-C, Christensen JJ, Parnell LD, Smith CE, Shao J, McKeown NM, et al.. Using Machine Learning to Predict Obesity Based on Genome-Wide and Epigenome-Wide Gene-Gene and Gene-Diet Interactions. *Front Genet*. 2021; doi: 10.3389/fgene.2021.783845.

16. Yeh AH-W, Norn C, Kipnis Y, Tischer D, Pellock SJ, Evans D, et al.. De novo design of luciferases using deep learning. *Nature*. 2023; doi: 10.1038/s41586-023-05696-3.

17. Brandes N, Goldman G, Wang CH, Ye CJ, Ntranos V. Genome-wide prediction of disease variant effects with a deep protein language model. *Nat Genet*. 2023; doi: 10.1038/s41588-023-01465-0.

18. Pinto MF, Oliveira H, Batista S, Cruz L, Pinto M, Correia I, et al.. Prediction of disease progression and outcomes in multiple sclerosis with machine learning. *Sci Rep*. nature.com; 2020; doi: 10.1038/s41598-020-78212-6.

19. Himmelstein DS, Baranzini SE. Heterogeneous Network Edge Prediction: A Data Integration Approach to Prioritize Disease-Associated Genes. *PLoS Comput Biol*. 2015; doi: 10.1371/journal.pcbi.1004259.

20. Sheikh N: Identification and classification of wildlife from camera-trap images using machine learning and computer vision. norma.ncirl.ie; https://norma.ncirl.ie/4283/1/nawazsheikh.pdf (2020). Accessed 2023 Dec 12.

21. Vélez J, McShea W, Shamon H, Castiblanco-Camacho PJ, Tabak MA, Chalmers C, et al.. An evaluation of platforms for processing camera-trap data using artificial intelligence. *Methods Ecol Evol*. Wiley; 2023; doi: 10.1111/2041-210x.14044.

22. Kutugata M, Baumgardt J, Goolsby JA, Racelis AE. Automatic Camera-Trap Classification Using Wildlife-Specific Deep Learning in Nilgai Management. *Journal of Fish and Wildlife Management*. Allen Press; 2021; doi: 10.3996/JFWM-20-076.

23. Terakita A. The opsins. *Genome Biol*. 2005; doi: 10.1186/gb-2005-6-3-213.

24. Govardovskii VI, Fyhrquist N, Reuter T, Kuzmin DG, Donner K. In search of the visual pigment template. *Vis Neurosci*. 2000; doi: 10.1017/s0952523800174036.

25. Jacobs GH, Neitz J, Krogh K. Electroretinogram flicker photometry and its applications. *J Opt Soc Am A Opt Image Sci Vis*. opg.optica.org; 1996; doi: 10.1364/josaa.13.000641.

26. Thomas MM, Lamb TD. Light adaptation and dark adaptation of human rod photoreceptors measured from the a-wave of the electroretinogram. *J Physiol*. Wiley Online Library; 1999; doi: 10.1111/j.1469-7793.1999.0479p.x.

27. Rocha FA de F, Gomes BD, Silveira LC de L, Martins SL, Aguiar RG, de Souza JM, et al.. Spectral Sensitivity Measured with Electroretinogram Using a Constant Response Method. *PLoS One*. journals.plos.org; 2016; doi: 10.1371/journal.pone.0147318.

28. Liebman PA. Microspectrophotometry of Photoreceptors. In: Abrahamson EW, Baumann C, Bridges CDB, Crescitelli F, Dartnall HJA, Eakin RM, et al., editors. *Photochemistry of Vision*. Berlin, Heidelberg: Springer Berlin Heidelberg;

29. Yewers MS, McLean CA, Moussalli A, Stuart-Fox D, Bennett ATD, Knott B. Spectral sensitivity of cone photoreceptors and opsin expression in two colour-divergent lineages of the lizard Ctenophorus decresii. *J Exp Biol*. journals.biologists.com; 2015; doi: 10.1242/jeb.131854.

30. Kojima D, Fukada Y. Spectroscopic Analysis of Wavelength Sensitivities of Opsin-Type Photoreceptor Proteins. In: Hirota T, Hatori M, Panda S, editors. *Circadian Clocks*. New York, NY: Springer US;

31. Carlson SD. Microspectrophotometry of visual pigments. *Q Rev Biophys*. cambridge.org; 1972; doi: 10.1017/s0033583500000986.

32. Bowmaker JK. Microspectrophotometry of vertebrate photoreceptors. A brief review. *Vision Res*. Elsevier; 1984; doi: 10.1016/0042-6989(84)90322-5.

33. Merbs SL, Nathans J. Absorption spectra of human cone pigments. *Nature*. 1992; doi: 10.1038/356433a0.

34. Liénard MA, Valencia-Montoya WA, Pierce NE. Molecular advances to study the function, evolution and spectral tuning of arthropod visual opsins. *Philos Trans R Soc Lond B Biol Sci*. royalsocietypublishing.org; 2022; doi: 10.1098/rstb.2021.0279.

35. Hagen JFD, Roberts NS, Johnston RJ Jr. The evolutionary history and spectral tuning of vertebrate visual opsins. *Dev Biol*. 2023; doi: 10.1016/j.ydbio.2022.10.014.

36. Yokoyama S, Radlwimmer FB. The molecular genetics and evolution of red and green color vision in vertebrates. *Genetics*. 2001; doi: 10.1093/genetics/158.4.1697.

37. Bloch NI. The evolution of opsins and color vision: connecting genotype to a complex

phenotype. *Acta Biolo Colomb*. Universidad Nacional de Colombia; 2016; doi: 10.15446/abc.v21n3.53907.

38. Rajamani R, Lin Y-L, Gao J. The opsin shift and mechanism of spectral tuning in rhodopsin. *J Comput Chem*. Wiley Online Library; 2011; doi: 10.1002/jcc.21663.

39. Hárosi FI. An analysis of two spectral properties of vertebrate visual pigments. *Vision Res*. 1994; doi: 10.1016/0042-6989(94)90134-1.

40. Wang W, Geiger JH, Borhan B. The photochemical determinants of color vision: revealing how opsins tune their chromophore's absorption wavelength. *Bioessays*. 2014; doi: 10.1002/bies.201300094.

41. Smedley GD, McElroy KE, Feller KD, Serb JM. Additive and epistatic effects influence spectral tuning in molluscan retinochrome opsin. *J Exp Biol*. journals.biologists.com; 2022; doi: 10.1242/jeb.242929.

42. Nathans J. Determinants of visual pigment absorbance: identification of the retinylidene Schiff's base counterion in bovine rhodopsin. *Biochemistry*. 1990; doi: 10.1021/bi00493a034.

43. Yokoyama S, Xing J, Liu Y, Faggionato D, Altun A, Starmer WT. Epistatic adaptive evolution of human color vision. *PLoS Genet*. journals.plos.org; 2014; doi: 10.1371/journal.pgen.1004884.

44. Yokoyama S, Altun A, Jia H, Yang H, Koyama T, Faggionato D, et al.. Adaptive evolutionary paths from UV reception to sensing violet light by epistatic interactions. *Sci Adv*. science.org; 2015; doi: 10.1126/sciadv.1500162.

45. Patel D, Barnes JE, Davies WIL, Stenkamp DL, Patel JS. Short-wavelength-sensitive 2 (Sws2) visual photopigment models combined with atomistic molecular simulations to predict spectral peaks of absorbance. *PLoS Comput Biol*. 2020; doi: 10.1371/journal.pcbi.1008212.

46. Patel JS, Brown CJ, Ytreberg FM, Stenkamp DL. Predicting peak spectral sensitivities of vertebrate cone visual pigments using atomistic molecular simulations. *PLoS Comput Biol*. 2018; doi: 10.1371/journal.pcbi.1005974.

47. Karasuyama M, Inoue K, Nakamura R, Kandori H, Takeuchi I. Understanding Colour Tuning Rules and Predicting Absorption Wavelengths of Microbial Rhodopsins by Data-Driven Machine-Learning Approach. *Sci Rep*. 2018; doi: 10.1038/s41598-018-33984-w.

48. Adam ZR, Schwieterman EW, Kacar B. Earliest photic zone niches probed by ancestral microbial rhodopsins. *Mol Biol*. academic.oup.com; 2022;

49. Longcore T. A compendium of photopigment peak sensitivities and visual spectral response curves of terrestrial wildlife to guide design of outdoor nighttime lighting. *Basic Appl Ecol*. 2023; doi: 10.1016/j.baae.2023.09.002.

50. Schott RK, Fujita MK, Streicher JW, Gower DJ, Thomas KN, Loew ER, et al.. Diversity and

Evolution of Frog Visual Opsins: Spectral Tuning and Adaptation to Distinct Light Environments. *Mol Biol Evol*. 2024; doi: 10.1093/molbev/msae049.

51. Schott RK, Perez L, Kwiatkowski MA, Imhoff V, Gumm JM. Evolutionary analyses of visual opsin genes in frogs and toads: Diversity, duplication, and positive selection. *Ecol Evol*. 2022; doi: 10.1002/ece3.8595.

52. Schweikert LE, Fitak RR, Caves EM, Sutton TT, Johnsen S. Spectral sensitivity in ray-finned fishes: diversity, ecology and shared descent. *J Exp Biol*. 2018; doi: 10.1242/jeb.189761.

53. Schweikert LE, Caves EM, Solie SE, Sutton TT, Johnsen S. Variation in rod spectral sensitivity of fishes is best predicted by habitat and depth. *J Fish Biol*. 2019; doi: 10.1111/jfb.13859.

54. Molina-Venegas R, Moreno-Saiz JC, Castro Parga I, Davies TJ, Peres-Neto PR, Rodríguez MÁ. Assessing among-lineage variability in phylogenetic imputation of functional trait datasets. *Ecography* . Wiley; 2018; doi: 10.1111/ecog.03480.

55. Garland T Jr, Ives AR. Using the Past to Predict the Present: Confidence Intervals for Regression Equations in Phylogenetic Comparative Methods. *Am Nat*. 2000; doi: 10.1086/303327.

56. Katoh K, Standley DM. MAFFT multiple sequence alignment software version 7: improvements in performance and usability. *Mol Biol Evol*. academic.oup.com; 2013; doi: 10.1093/molbev/mst010.

57. Edgar RC. MUSCLE: multiple sequence alignment with high accuracy and high throughput. *Nucleic Acids Res*. academic.oup.com; 2004; doi: 10.1093/nar/gkh340.

58. Castresana J. Selection of conserved blocks from multiple alignments for their use in phylogenetic analysis. *Mol Biol Evol*. 2000; doi: 10.1093/oxfordjournals.molbev.a026334.

59. Baghbanzadeh M, Dawson T, Sayoldin B, Oakley T, Crandall K, Rahnavard A. DeepBreaks: A machine learning tool for identifying and prioritizing genotype-phenotype associations. Research Square.

60. Chen T, He T, Benesty M, Khotilovich V, Tang Y, Cho H, et al.. Xgboost: extreme gradient boosting. *R package version 0 4-2*. cran.ms.unimelb.edu.au; 1:1–42015;

61. Sibindi R, Mwangi RW, Waititu AG. A boosting ensemble learning based hybrid light gradient boosting machine and extreme gradient boosting model for predicting house prices. *Eng Rep*. Wiley; 2023; doi: 10.1002/eng2.12599.

62. Ke G, Meng Q, Finley T, Wang T, Chen W, Ma W, et al.. LightGBM: A highly efficient Gradient Boosting Decision Tree. *Adv Neural Inf Process Syst*. :3146–542017;

63. Schapire RE. Explaining AdaBoost. In: Schölkopf B, Luo Z, Vovk V, editors. *Empirical Inference: Festschrift in Honor of Vladimir N Vapnik*. Berlin, Heidelberg: Springer Berlin

Heidelberg;

64. Rigatti SJ. Random Forest. *J Insur Med*. meridian.allenpress.com; 2017; doi: 10.17849/insm-47-01-31-39.1.

65. Segal MR. Machine Learning Benchmarks and Random Forest Regression. escholarship.org; 2004;

66. Pedregosa F, Varoquaux G, Gramfort A, Michel V, Thirion B, Grisel O, et al.. Scikit-learn: Machine Learning in Python. *J Mach Learn Res*. JMLR.org; 12:2825–302011;

67. Prettenhofer P, Louppe G. Gradient Boosted Regression Trees in Scikit-Learn. *PyData 2014*. orbi.uliege.be;

68. Hunter JD. Matplotlib: A 2D Graphics Environment. *Comput Sci Eng*. IEEE; May-June 2007; doi: 10.1109/MCSE.2007.55.

69. Damian Riina M, Stambaugh C, Stambaugh N, Huber KE. Chapter 28 - Continuous variable analyses: t-test, Mann–Whitney, Wilcoxin rank. In: Eltorai AEM, Bakal JA, Kim DW, Wazer DE, editors. *Translational Radiation Oncology*. Academic Press;

70. Gommers R, Virtanen P, Burovski E, Weckesser W, Oliphant TE, Haberland M, et al.. scipy/scipy: SciPy 1.9.0. Zenodo. Zenodo;

71. Silva JO, Orellana ETV, Torres M. Development of a Parallel Version of PhyML 3.0 Using Shared Memory. *IEEE Latin America Transactions*. IEEE; 2017; doi: 10.1109/TLA.2017.7912593.

72. Le SQ, Gascuel O. An improved general amino acid replacement matrix. *Mol Biol Evol*. academic.oup.com; 2008; doi: 10.1093/molbev/msn067.

73. Revell LJ. phytools: an R package for phylogenetic comparative biology (and other things). *Methods Ecol Evol*. Wiley; 2012; doi: 10.1111/j.2041-210x.2011.00169.x.

74. Sneddon TP, Li P, Edmunds SC. GigaDB: announcing the GigaScience database. *Gigascience*. 2012; doi: 10.1186/2047-217X-1-11.

75. Friedman JH. Greedy Function Approximation: A Gradient Boosting Machine. *Ann Stat*. Institute of Mathematical Statistics; 29:1189–2322001;

76. Bedoui A, Lazar NA. Bayesian empirical likelihood for ridge and lasso regressions. *Comput Stat Data Anal*. Elsevier; 2020; doi: 10.1016/j.csda.2020.106917.

77. Karabatsos G. Fast Marginal Likelihood Estimation of the Ridge Parameter(s) in Ridge Regression and Generalized Ridge Regression for Big Data. arXiv [stat.ME].

78. Fan J, Ma X, Wu L, Zhang F, Yu X, Zeng W. Light Gradient Boosting Machine: An efficient soft computing model for estimating daily reference evapotranspiration with local and external

meteorological data. *Agric Water Manage*. Elsevier; 2019; doi: 10.1016/j.agwat.2019.105758.

79. Roberts DR, Bahn V, Ciuti S, Boyce MS, Elith J, Guillera-Arroita G, et al.. Cross-validation strategies for data with temporal, spatial, hierarchical, or phylogenetic structure. *Ecography* . Wiley; 2017; doi: 10.1111/ecog.02881.

80. Yokoyama S, Radlwimmer FB. The “five-sites” rule and the evolution of red and green color vision in mammals. *Mol Biol Evol*. 1998; doi: 10.1093/oxfordjournals.molbev.a025956.

81. Shichida Y, Matsuyama T. Evolution of opsins and phototransduction. *Philos Trans R Soc Lond B Biol Sci*. 2009; doi: 10.1098/rstb.2009.0051.

82. Terakita A, Koyanagi M, Tsukamoto H, Yamashita T, Miyata T, Shichida Y. Counterion displacement in the molecular evolution of the rhodopsin family. *Nat Struct Mol Biol*. 2004; doi: 10.1038/nsmb731.

83. Shi Y, Radlwimmer FB, Yokoyama S. Molecular genetics and the evolution of ultraviolet vision in vertebrates. *Proc Natl Acad Sci U S A*. 2001; doi: 10.1073/pnas.201257398.

84. Sugawara T, Terai Y, Imai H, Turner GF, Koblmüller S, Sturmbauer C, et al.. Parallelism of amino acid changes at the RH1 affecting spectral sensitivity among deep-water cichlids from Lakes Tanganyika and Malawi. *Proc Natl Acad Sci U S A*. 2005; doi: 10.1073/pnas.0405302102.

85. Takenaka N, Yokoyama S. Mechanisms of spectral tuning in the RH2 pigments of Tokay gecko and American chameleon. *Gene*. 2007; doi: 10.1016/j.gene.2007.04.036.

86. Yokoyama S, Tada T, Zhang H, Britt L. Elucidation of phenotypic adaptations: Molecular analyses of dim-light vision proteins in vertebrates. *Proc Natl Acad Sci U S A*. 2008; doi: 10.1073/pnas.0802426105.

87. Shannon C. A mathematical theory of communication (1948). direct.mit.edu; 2021; doi: 10.7551/MITPRESS/12274.003.0014.

88. Ramazzotti M, Degl’Innocenti D, Manao G, Ramponi G. Entropy calculator: getting the best from your multiple protein alignments. *Ital J Biochem*. researchgate.net; 53:16–222004;

89. Lin SW, Sakmar TP. Colour tuning mechanisms of visual pigments. *Novartis Found Symp*. 1999; doi: 10.1002/9780470515693.ch8.

90. Chan T, Lee M, Sakmar TP. Introduction of hydroxyl-bearing amino acids causes bathochromic spectral shifts in rhodopsin. Amino acid substitutions responsible for red-green color pigment spectral tuning. *J Biol Chem*. 267:9478–801992;

91. Orgogozo V, Morizot B, Martin A. The differential view of genotype–phenotype relationships. *Front Genet*. frontiersin.org; 2015; doi: 10.3389/fgene.2015.00179.

92. Baldwin MW, Ko M-C. Functional evolution of vertebrate sensory receptors. *Horm Behav*. Elsevier; 2020; doi: 10.1016/j.yhbeh.2020.104771.

93. Park Y, Metzger BPH, Thornton JW. Epistatic drift causes gradual decay of predictability in protein evolution. *Science*. science.org; 2022; doi: 10.1126/science.abn6895.

94. Lyons DM, Zou Z, Xu H, Zhang J. Idiosyncratic epistasis creates universals in mutational effects and evolutionary trajectories. *Nat Ecol Evol*. nature.com; 2020; doi: 10.1038/s41559-020-01286-y.

95. Gonzalez Somermeyer L, Fleiss A, Mishin AS, Bozhanova NG, Igolkina AA, Meiler J, et al.. Heterogeneity of the GFP fitness landscape and data-driven protein design. *Elife*. elifesciences.org; 2022; doi: 10.7554/eLife.75842.

96. Friedman JH. Stochastic gradient boosting. *Comput Stat Data Anal*. 2002; doi: 10.1016/S0167-9473(01)00065-2.

97. Sekharan S, Morokuma K. Why 11-cis-retinal? Why not 7-cis-, 9-cis-, or 13-cis-retinal in the eye? *J Am Chem Soc*. ACS Publications; 2011; doi: 10.1021/ja208789h.

98. Buczyłko J, Saari JC, Crouch RK, Palczewski K. Mechanisms of opsin activation. *J Biol Chem*. ASBMB; 1996; doi: 10.1074/jbc.271.34.20621.

99. Das D, Wilkie SE, Hunt DM, Bowmaker JK. Visual pigments and oil droplets in the retina of a passerine bird, the canary Serinus canaria: microspectrophotometry and opsin sequences. *Vision Res*. 1999; doi: 10.1016/s0042-6989(99)00023-1.

100. Toomey MB, Collins AM, Frederiksen R, Cornwall MC, Timlin JA, Corbo JC. A complex carotenoid palette tunes avian colour vision. *J R Soc Interface*. royalsocietypublishing.org; 2015; doi: 10.1098/rsif.2015.0563.

101. Hart NS, Vorobyev M. Modelling oil droplet absorption spectra and spectral sensitivities of bird cone photoreceptors. *J Comp Physiol A Neuroethol Sens Neural Behav Physiol*. Springer; 2005; doi: 10.1007/s00359-004-0595-3.

102. Toomey MB, Corbo JC. Evolution, Development and Function of Vertebrate Cone Oil Droplets. *Front Neural Circuits*. frontiersin.org; 2017; doi: 10.3389/fncir.2017.00097.

103. Arikawa K, Stavenga D. Random array of colour filters in the eyes of butterflies. *J Exp Biol*. 1997; doi: 10.1242/jeb.200.19.2501.

104. Feller KD, Wilby D, Jacucci G, Vignolini S, Mantell J, Wardill TJ, et al.. Long-Wavelength Reflecting Filters Found in the Larval Retinas of One Mantis Shrimp Family (Nannosquillidae). *Curr Biol*. 2019; doi: 10.1016/j.cub.2019.07.070.

105. Partridge JC, White EM, Douglas RH. The effect of elevated hydrostatic pressure on the spectral absorption of deep-sea fish visual pigments. *J Exp Biol*. 2006; doi: 10.1242/jeb.01984.

106. Ogbunugafor CB, Wylie CS, Diakite I, Weinreich DM, Hartl DL. Adaptive Landscape by Environment Interactions Dictate Evolutionary Dynamics in Models of Drug Resistance. *PLoS Comput Biol*. 2016; doi: 10.1371/journal.pcbi.1004710.

107. Woolley S, Johnson J, Smith MJ, Crandall KA, McClellan DA. TreeSAAP: selection on amino acid properties using phylogenetic trees. *Bioinformatics*. 2003; doi: 10.1093/bioinformatics/btg043.

108. Inoue K, Karasuyama M, Nakamura R, Konno M, Yamada D, Mannen K, et al.. Exploration of natural red-shifted rhodopsins using a machine learning-based Bayesian experimental design. *Commun Biol*. 2021; doi: 10.1038/s42003-021-01878-9.

109. Palczewski K, Kumasaka T, Hori T, Behnke CA, Motoshima H, Fox BA, et al.. Crystal structure of rhodopsin: A G protein-coupled receptor. *Science*. 2000; doi: 10.1126/science.289.5480.739.

110. Murakami M, Kouyama T. Crystal structure of squid rhodopsin. *Nature*. 2008; doi: 10.1038/nature06925.

111. Briscoe AD. Homology modeling suggests a functional role for parallel amino acid substitutions between bee and butterfly red- and green-sensitive opsins. *Mol Biol Evol*. 2002; doi: 10.1093/oxfordjournals.molbev.a004158.

112. Jumper J, Evans R, Pritzel A, Green T, Figurnov M, Ronneberger O, et al.. Highly accurate protein structure prediction with AlphaFold. *Nature*. 2021; doi: 10.1038/s41586-021-03819-2.

113. Van Nynatten A, Castiglione GM, de A Gutierrez E, Lovejoy NR, Chang BSW. Recreated Ancestral Opsin Associated with Marine to Freshwater Croaker Invasion Reveals Kinetic and Spectral Adaptation. *Mol Biol Evol*. 2021; doi: 10.1093/molbev/msab008.

114. Porter ML, Roberts NW, Partridge JC. Evolution under pressure and the adaptation of visual pigment compressibility in deep-sea environments. *Mol Phylogenet Evol*. Elsevier; 2016; doi: 10.1016/j.ympev.2016.08.007.

115. Schweikert LE, Bagge LE, Naughton LF, Bolin JR, Wheeler BR, Grace MS, et al.. Dynamic light filtering over dermal opsin as a sensory feedback system in fish color change. *Nat Commun*. Nature Publishing Group UK London; 14:46422023;

116. Borghezan E de A, da Silva Pires TH, Zuanon J, Sugiura H, Kohshima S, Kishida T. Unstable environmental conditions constrain the fine-tune between opsin sensitivity and underwater light in an Amazon forest stream fish. *J Evol Biol*. Oxford University Press; 2024; doi: 10.1093/jeb/voae001.

117. Murphy MJ, Westerman EL. Evolutionary history limits species' ability to match colour sensitivity to available habitat light. *Proc Biol Sci*. 2022; doi: 10.1098/rspb.2022.0612.

118. Kwon E, Heo WD. Optogenetic tools for dissecting complex intracellular signaling pathways. *Biochem Biophys Res Commun*. 2020; doi: 10.1016/j.bbrc.2019.12.132.

119. Mukherjee A, Repina NA, Schaffer DV, Kane RS. Optogenetic tools for cell biological applications. J. Thorac. Dis.

120. Tischer D, Weiner OD. Illuminating cell signalling with optogenetic tools. *Nat Rev Mol Cell Biol*. 2014; doi: 10.1038/nrm3837.

121. Kaur P, Saunders TE, Tolwinski NS. Coupling optogenetics and light-sheet microscopy, a method to study Wnt signaling during embryogenesis. *Sci Rep*. nature.com; 2017; doi: 10.1038/s41598-017-16879-0.

122. Fan H, Barnes C, Hwang H, Zhang K, Yang J. Precise modulation of embryonic development through optogenetics. *Genesis*. Wiley Online Library; 2022; doi: 10.1002/dvg.23505.

123. Sparta DR, Jennings JH, Ung RL, Stuber GD. Optogenetic strategies to investigate neural circuitry engaged by stress. *Behav Brain Res*. Elsevier; 2013; doi: 10.1016/j.bbr.2013.05.007.

124. Belzung C, Turiault M, Griebel G. Optogenetics to study the circuits of fear- and depression-like behaviors: a critical analysis. *Pharmacol Biochem Behav*. Elsevier; 2014; doi: 10.1016/j.pbb.2014.04.002.

125. Muir J, Lopez J, Bagot RC. Wiring the depressed brain: optogenetic and chemogenetic circuit interrogation in animal models of depression. *Neuropsychopharmacology*. nature.com; 2019; doi: 10.1038/s41386-018-0291-6.

126. LaLumiere RT. A new technique for controlling the brain: optogenetics and its potential for use in research and the clinic. *Brain Stimul*. 2011; doi: 10.1016/j.brs.2010.09.009.

127. Montagni E, Resta F, Mascaro ALA, Pavone FS. Optogenetics in Brain Research: From a Strategy to Investigate Physiological Function to a Therapeutic Tool. *Photonics*. Multidisciplinary Digital Publishing Institute; 2019; doi: 10.3390/photonics6030092.

128. Penn WD, McKee AG, Kuntz CP, Woods H, Nash V, Gruenhagen TC, et al.. Probing biophysical sequence constraints within the transmembrane domains of rhodopsin by deep mutational scanning. *Sci Adv*. 2020; doi: 10.1126/sciadv.aay7505.

129. Hensley NM, Ellis EA, Leung NY, Coupart J, Mikhailovsky A, Taketa DA, et al.. Selection, drift, and constraint in cypridinid luciferases and the diversification of bioluminescent signals in sea fireflies. *Mol Ecol*. 2021; doi: 10.1111/mec.15673.

130. Schenkmayerova A, Pinto GP, Toul M, Marek M, Hernychova L, Planas-Iglesias J, et al.. Engineering the protein dynamics of an ancestral luciferase. *Nat Commun*. 2021; doi: 10.1038/s41467-021-23450-z.

Manuscript with track changes

Click here to access/download;Manuscript;Revision2 Track Changes - Frazer et al.docx

Discovering genotype-phenotype relationships with machine learning and the Visual Physiology Opsin Database (VPOD)

Seth A. Frazer[1], Mahdi Baghbanzadeh[2], Ali Rahnavard[2], Keith A. Crandall[2,3], Todd H. Oakley[1,*]

Affiliations:

[1]Ecology, Evolution, and Marine Biology, University of California, Santa Barbara, California 93106

[2]Computational Biology Institute, Department of Biostatistics and Bioinformatics, Milken Institute School of Public Health, The George Washington University, Washington, DC 20052

[3]Department of Invertebrate Zoology, National Museum of Natural History, Smithsonian Institution, Washington, DC 20012

ORCID and Email:

Seth A. Frazer
sethfrazer@ucsb.edu
https://orcid.org/0000-0002-3800-212X

Mahdi Baghbanzadeh
mbagh@gwu.edu
https://orcid.org/0000-0002-1878-2691

Keith A. Crandall
kcrandall@gwu.edu
https://orcid.org/0000-0002-0836-3389

Ali Rahnavard
rahnavard@gwu.edu
https://orcid.org/0000-0002-9710-0248

Todd H. Oakley
oakley@ucsb.edu
https://orcid.org/0000-0002-4478-915X
*Correspondence to oakley@ucsb.edu

Abstract

Background: Predicting phenotypes from genetic variation is foundational for fields as diverse as bioengineering and global change biology, highlighting the importance of efficient methods to predict gene functions. Linking genetic changes to phenotypic changes has been a goal of decades of experimental work, especially for some model gene families including light-sensitive opsin proteins. Opsins can be expressed in vitro to measure light absorption parameters, including λ_{max} - the wavelength of maximum absorbance - which strongly affects organismal phenotypes like color vision. Despite extensive research on opsins, the data remain dispersed, uncompiled, and often challenging to access, thereby precluding systematic and comprehensive analyses of the intricate relationships between genotype and phenotype.

Results: Here, we report a newly compiled database of all heterologously expressed opsin genes with λ_{max} phenotypes that we call the Visual Physiology Opsin Database (*VPOD*). *VPOD_1.0* contains 864 unique opsin genotypes and corresponding λ_{max} phenotypes collected across all animals from 73 separate publications. We use *VPOD* data and *deepBreaks* to show regression-based machine learning (ML) models often reliably predict λ_{max}, account for non-additive effects of mutations on function, and identify functionally critical amino acid sites.

Conclusion: The ability to reliably predict functions from gene sequences alone using ML will allow robust exploration of molecular-evolutionary patterns governing phenotype, will inform functional and evolutionary connections to an organism's ecological niche, and may be used more broadly for *de-novo* protein design. Together, our database, phenotype predictions, and model comparisons lay the groundwork for future research applicable to families of genes with quantifiable and comparable phenotypes.

Key words: Machine learning; Regression, Compiled database; Genotype-phenotype relationships; Predicting phenotypes; Spectral sensitivity; Color-vision; Opsins; Imputation

Key Points

• We introduce the Visual Physiology Opsin Database (VPOD_1.0), which includes 864 unique animal opsin genotypes and corresponding λ_{max} phenotypes from 73 separate publications.

• We demonstrate that regression-based ML models can reliably predict λmax from gene sequence alone, predict non-additive effects of mutations on function, and identify functionally critical amino acid sites.

• We provide an approach that lays the groundwork for future robust exploration of molecular-evolutionary patterns governing phenotype, with potential broader applications to any family of genes with quantifiable and comparable phenotypes.

Formatted Table

Introduction

Although critical to progress in drug and vaccine design [1–3], responses to climate change [4–8], and bioengineering [4,9–11], accurately predicting gene function from sequences remains a significant challenge. While there are many ways to elucidate genotype-phenotype relationships experimentally, including deep mutational scanning, and in-vitro heterologous expression with phenotyping, these techniques are often tedious and cost-prohibitive, especially when applied to broad comparative studies of gene families. In addition, accurately predicting the phenotype of a protein using computational methods alone is challenging because of data gaps and the sheer complexity of possible relationships between genes and phenotypes, including epistasis and the non-additive effects of different mutations. Machine learning (ML) is gaining traction for its potential broad biological applications, accessibility, and faster speeds, especially in biological contexts where phenotype data are abundant and quantifiable. Here, classical regression and classification algorithms are sometimes used to train models for phenotype predictions using genotype-phenotype data [12,13], while deep learning models can be used to integrate heterogeneous multi-layered omics and environmental data for establishing higher dimensional genotype-phenotype connections [14,15] or *de-novo* protein design [16]. In broader biological contexts, ML models often inform laboratory experiments to predict directional evolution of diseases and their variants [17–19] or to automate image sorting and animal identification from camera trap data [20–22]. In all cases, ML models are a worthwhile long-term investment for genotype-phenotype studies because models can iteratively improve as empirical data accumulate over time.

Such accumulation of important information is exemplified by decades of laboratory work that has led to significant progress in understanding the genetic basis of phenotypic changes for model gene families such as opsins. Opsins are a family of G-protein Coupled Receptors (GPCR) which bind to a retinal chromophore. The two units together, opsin and chromophore, form visual pigments which absorb photons [23]. Opsins have crucial roles in many organismal functions, including circadian rhythms, phototaxis, and image-forming color vision. A critical opsin phenotype is spectral sensitivity - the range of wavelengths to which a gene or organism is sensitive. The main parameter of opsin spectral sensitivity is λ_{max}, the wavelength of light (in nm) with maximal absorbance [24]. Common methods of characterizing spectral sensitivities and λ_{max} include organ-level electroretinograms (ERG) [25–27], cell-level microspectrophotometry (MSP) [28–32], purification of heterologously expressed opsins followed by spectrophotometry [33], and heterologous action spectroscopy using light response assays for opsins expressed in immortalized cell lines [34]. Different opsins are tuned by changes in amino acid sequences to respond to different wavelengths of light, and many previous studies have expressed experimentally mutated opsins and measured spectral sensitivities to establish genotype-phenotype connections [34–38]. Although other factors sometimes affect spectral responsiveness, including the type of chromophore to which an opsin is covalently bound (11-cis retinal or 11-cis-3,4-didehydro retinal) [39,40], opsins provide a rare case where an intrinsic molecular function extends rather directly to organismal phenotypes, especially those involving

color sensitivity. Despite opsins being a well-studied system with an extensive backlog of published literature, some previous authors expressed doubts that sequence data alone could provide reliable computational predictions of λ_{max} phenotypes [41–44]. At the same time, some λ_{max} predictions showed promise, although on the limited scale of vertebrate cone visual pigments via atomistic molecular simulations [45,46]. Furthermore, only the non-animal, microbial, or Type-1 (T1) opsins have been systematically cataloged and used to examine genotype-phenotype predictive power of ML models [47,48]. While some researchers have made significant efforts to compile peak sensitivity data for terrestrial animal photopigments [49], and taxon-specific light-sensitivity data for groups like frogs [50,51] and ray-finned fishes [52,53], these efforts currently lack direct links to genetic data that are essential for our current study. Consequently, the extensive data on genotype-phenotype associations of animal opsins remains disorganized, decentralized, often in non-computer readable formats within older literature, and under-analyzed computationally.

Here, we report a genotype-phenotype database for animal opsins called the Visual Physiology Opsin Database (*VPOD*). We used standard literature searches to compile all heterologously expressed animal opsin genes with spectral sensitivity measurements. We used this newly compiled and harmonized database to evaluate ML methods for connecting genotypes and phenotypes. We created eleven subsets of the overall database to examine factors that impact the reliability and performance of ML models and briefly compared ML predictions to phylogenetic imputation [54,55]. We also examined whether ML can predict intragenic epistasis, and we predicted amino acid sites particularly important for changing λ_{max}. Using our database of 864 unique opsin sequences and corresponding λ_{max} values, we show ML models trained on opsin data accurately predict the λ_{max} of opsins from genetic data alone [highest R^2 =0.968 with a lowest mean absolute error (MAE) of 6.56 nm], especially when ample and diverse training data are available. ML also predicts some known effects of epistatic mutations on λ_{max}. Finally, ML models identify several sites that cause shifts in λ_{max} (e.g., 'spectral tuning sites') and sites known to be structurally important, even in the absence of mutant data in training. When training data are sufficient, these results support the use of ML as a reliable and efficient predictor of λ_{max} for previously uncharacterized opsins, as a tool for identifying candidate spectral tuning sites and epistatic interactions, and as a more general method for linking gene sequences and phenotypes.

Methods

Compiling a genotype-phenotype database for animal opsins

We collected λ_{max} data for opsins using typical literature review/search methods, with search engine, keywords, and date of access documented in the '*litsearch'* table of the *VPOD* database. We cataloged all usable papers with λ_{max} data in the '*references'* table of *VPOD*, recording DOI and a key to link to the search that found the paper. We documented the details of heterologous expression experiments in the '*heterologous'* table, including species, GenBank accession number for the sequence, mutation(s) (if applicable) using a machine-readable notation, λ_{max}, cell type for expression (e.g., HEK293, COS1, etc.), protein purification method,

type of spectrum (e.g., dark or difference spectrum), and a key to link to the corresponding literature source. Note, we did not record the chromophore used to reconstitute the purified opsin protein because 11-cis retinal is the standard and all instances thus far recorded in the '*heterologous'* table are from experiments using 11-cis retinal (although future iterations of VPOD could record these details if data with alternative chromophores becomes available). We input opsin genetic data in an *'opsins'* table, recording opsin gene family names (e.g., long-wave sensitive=LWS, short-wave sensitive=SWS1, etc.). We also included specific '*gene names'* (where applicable), phylum, class, species information, accession number, DNA sequence, amino acid sequence, and the database from which sequences were retrieved (e.g., NCBI). We recreated all mutant and chimeric (e.g., one or more transmembrane domains of the mutant copied from a different sequence to replace the original) opsin sequences based on literature descriptions using a pair of Python scripts (*mutagenesis.py* and *chimeras.py*) available on our GitHub (https://github.com/VisualPhysiologyDB/visual-physiology-opsin-db). We added all heterologously expressed opsins from the literature to *VPOD*; we call this version of the database *VPOD_1.0*. We refer to heterologous data as *VPOD_het_1.0*, which will allow for future additions to the database to link specific opsin sequences to λ_{max} values established with methods other than heterologous expression, including microspectrophotometry or other methods. During the course of manuscript review, we found and entered 259 new heterologously expressed opsins into VPOD, an update we call *VPOD_1.1* (Figure 1). We decided to keep results from *VPOD_1.0* in the main text because the new data points did not drastically alter any model performances. We also provide this table of performance metrics for *VPOD_1.1* (Table S1). Therefore, all tests and figures should still be assumed to use *VPOD_1.0* data unless stated otherwise.

Training ML models with *deepBreaks*

We performed all data pre-processing, including data extraction, sequence alignments, and formatting, in the Jupyter notebooks '*opsin_model_wf.ipynb*', available on GitHub. We used two multiple sequence alignment methods, MAFFT [56] and MUSCLE [57], and a version of both alignments with a Gblocks [58] refinement (for a total of four alignments), all set to their default parameters to begin to test the sensitivity of model performance to different alignments. We then trained various ML models employing a custom version of *deepBreaks* [59], an ML tool designed for exploring genotype-phenotype associations. *deepBreaks* takes aligned genotype data (DNA, RNA, Amino Acid) and some measure(s) of corresponding continuous or categorical phenotype data as input to train ML models. deepBreaks uses one-hot encoding to convert amino acid sequences into numerical values. One consequence of this encoding is any amino acids at a given position in the alignment, which are not present at that position in any training data, will be treated equivalently as unseen. For example, cases of only A and V at a highly conserved site in the training set that are presented with a sequence with T at that site will be considered as no A and no V. The models cannot distinguish the input whether it's T or other unseen amino acids at that site. The results produced by *deepBreaks* encompass a compilation of 12 regression ML

models [59], showcasing ten metrics of cross-validation performance (ranked by R^2) and a feature importance report derived from the top-performing modelmodels which ranks amino acid positions by their relative importance to theeach model (from 0.0-1.0, with 1.0 being a site with the highest relative importance) for the phenotype in question (λ_{max}). The metrics used to determine these relative importance scores of each position vary based on the structure and output of the algorithms used for model training. For example, xgboost [60] and LightGBM [61,62] use the number of times a feature appears in a tree as a proxy for importance [59], while AdaBoost [63] and random forest [64,65], use Gini importance, which quantifies a feature's contribution to improving prediction accuracy [59,66,67]. For a more detailed explanation on how position importance scores are calculated for different models, refer to the '*Interpretation'* heading under the methods section of the *deepBreaks* publication [59]. In addition to R^2, *deepBreaks* reports the Mean Absolute Error (MAE), Mean Absolute Percent Error (MAPE), Mean Square Error (MSE), Root Mean Square Error (RMSE) for each of the 12 ML models. We evaluated the performance of algorithms based on their relative ranks to look for patterns in which algorithms performed better for different data subsets and approaches. *deepBreaks* also produces a set of distribution box plots (default is 100) to visualize phenotypes (λ_{max}) associated with a particular amino acid identity at a site of interest, ordered alphabetically.

Formatted: Font: Roboto, 10.5 pt, Font color: Custom Color(RGB(68,71,70))

Understanding model performance using different subsets of the database

We created eleven data subsets with varying levels of taxonomic and gene family inclusivity (Table 1) to test which factors most impact the reliability/performance of ML methods. We used naming conventions that include versioning to improve reproducibility and reliability of individual datasets and models. For example, one subset combines ultraviolet and SWS opsins, which we named *VPOD_uss_het_1.0*. Our convention is to name the subset (in this case USS = 'Ultraviolet and Short-wave Sensitive' opsins); name the source of phenotype data (heterologous = het), and record the version number of the dataset (1.0). We also created subsets for medium- and long-wave sensitive opsins (*VPOD_mls_het_1.0)* and all rod (Rh1) and rod-like (Rh2) opsins (*VPOD_rod_het_1.0*). Other subsets use species taxonomy, one for vertebrates (*VPOD_vert_het_1.0)* and another for invertebrates (*VPOD_inv_het_1.0)*. For taxonomic subsets, we considered all sequences from phylum Chordata as 'vertebrates' and the rest as 'invertebrates'. Another subset excludes all mutant opsin sequences, called 'wild-types' (*VPOD_wt_het_1.0)*. A final named subset is the whole data set (*VPOD_wds_het_1.0)*(Figure 2).

Using various subsets of data, we performed a number of experiments to better understand the performance of ML models in predicting λ_{max}. First, to better understand how training data relate to model performance, R^2 and training data size, we gradually increased the size of training datasets by starting from zero and incrementally adding between 15-50 randomly selected sequences at a time for the WDS, Vertebrate, WT, and Rod subsets separately; repeating the process three times per subset (Table S2). We then analyzed the fit between the size of training data sets (x-axis) and model performance (y-axis), comparing six non-linear models with AIC to find the model that best explains the observed variation (Figure S3). Second, to

understand if ML could predict known phenotypic changes due to experimental mutations, we queried the top performing WT model (which lacks data from artificially mutated sequences) using all experimentally mutated opsins to predict their known phenotypes. We plotted these results using *matplotlib* [~~60~~68] to visualize characteristics of poorly predicted outliers (e.g., taxonomic bias or sensitivity to mutations which caused large shifts in λ_{max} from the WT) (Figure 3). To test further whether including these mutant data significantly improves predictions of λ_{max}, we used the *VPOD_het_1.1* dataset (Table S1) and a *Wilcoxon Signed-Rank Test* ~~(WSRT)~~ [~~61,62~~69,70] to compare distributions of squared error for predictions by the WDS model (contains mutant data) and WT model (no mutant data) on all mutant data (n = 761) and separately comparing only mutants causing the largest phenotypic changes in λ_{max} (>10 nm from the wild-type; n = 346). To accomplish this for the WDS models, we iteratively removed 25 mutant opsins at a time from training data, used the same training algorithm (Gradient Boosted Regressor, GBR), and predicted λ_{max} values of withheld opsins ~~for each~~following the completion of model training ~~cycle~~(withheld opsins are not used as test data during the actual model training), until all mutant opsins were sampled once (this notebooks is available on GitHub as *'vpod_wf_iterate_subsample.ipynb'*. Third, we examined the ability of our models to predict λ_{max} of thirty invertebrate opsins not in *VPOD_1.0* because they are only known from physiological studies (Table S4, Figure S5). Here, we collected data both characterized by single-cell microspectrophotometry (MSP) or electroretinogram methods and with expression localized to cell-type by *in-situ-hybridization* (ISH), to link λ_{max} to a specific opsin (the sequences and metadata can be found in '*msp_erg_raw.txt'* and '*msp_erg_meta.tsv'*, while the resulting predictions can be found under the *'msp_tests'* folder on our GitHub repository). Finally, we directly compared predictive capabilities of models trained on different data subsets by randomly selecting and removing the same 25 wild-type ultraviolet or short-wave sensitive opsins from the training data of the WDS, Vertebrate, WT, and UVS/SWS models before training and querying the model with those same sequences following training (Table S4, Figure S6).

Formatted: Font: Not Italic

Formatted: Font: Not Italic

Formatted: Font: Not Italic

Comparing Machine Learning and Phylogenetic Imputation

We compared performance of ML models to phylogenetic imputation, which estimates phenotypes using phylogenetic information [54,55]. Phylogenetic imputation uses maximum likelihood (we will not abbreviate maximum likelihood as ML to avoid confusion with machine learning), usually assuming Brownian Motion to predict missing phenotypes using a phylogenetic tree, such that more closely related species or sequences have more similar phenotypes. For the phylogeny, we constructed opsin gene trees in phyML [~~63~~71], assuming the 'WAG' substitution model [~~64~~72] and a proportion of 0.029 invariable sites, with Gamma as a rate across sites model, and four substitution rate classes. We randomly removed 50 opsin sequences, and their corresponding λ_{max} values from each of the ML training datasets (with the exception of the smaller MWS/LWS and invertebrate datasets, where we only removed 15), then estimated the removed λ_{max} values using phylogenetic imputation. We used the phylogenetic imputation sub-module of the *phytools* R package [~~65~~73] for imputation. We compared imputed

and actual λ_{max} using regression. Imputation seemed sensitive to input alignment, perhaps caused by very short or zero length branch lengths in the phylogeny, as we could only complete imputation with *phytools* after removing uninformative and heavily gapped regions with Gblocks. To allow direct comparisons of regressions between imputation and ML, we recreated ML training-data alignments using MAFFT, MUSCLE, and Gblocks in the same way as for imputation and predicted λ_{max} for the same sets of sequences as imputation (Table S7).

Testing ability of ML to account for intragenic epistasis

Functional predictions are often misled by epistasis [41], so we tested the ability of our WDS models to predict the effects of epistatic mutations by haphazardly selecting three double mutants with previously demonstrated epistatic effects from training data in which double mutants, each single mutant, and wild type sequence are all characterized by heterologous expression. The three epistatic double mutants are all derived from bovine rhodopsins: D83N_A292S, F261Y_A269T, and A164S_A269T. We removed the double mutants from the training dataset but retained single mutants to test whether the model treats the mutations as additive or epistatic. We hypothesized that the many instances of multi-mutant sequences with epistatic effects in the training set would allow the model to account for both the magnitude and direction of intragenic epistasis. We then ran a separate test where we removed the same double mutants plus their corresponding single mutants to observe whether the WDS model still predicts epistatic effects from wild type data alone. We subsequently repeated this same process for the WT and Vertebrate models (Table S8).

We ran an additional experiment to test the general ability to to predict epistatic interactions between mutations for all available data. Here, we identified all multi-mutants that have phenotype data for each individual component mutation. Next we selected those multi-mutants with non-additive (epistatic) interactions between mutations (which we define as >1 nm difference between the actual multi-mutant phenotype and the sum of changes in phenotype due to the individual mutations). These 111 ‘epistatic mutants’ were then all removed from WDS (*VPOD_wds_het_1.1*) to create a new training data set called ‘WDS-minusepi’ that lacks evidence of intragenic epistasis. For this test we hypothesized that if the ML approach can account for epistasis, the RMSE of predictions of the 111 epistatic mutants would be significantly lower for the model trained with WDS-minusepi than the model trained with no mutants at all (WT). We tested for statistically significant differences in the distributions of square error for predictions made by WDS-minusepi versus WT; WDS-minusepi versus the ~~Expected~~Epistasis-free Additive Mutation Values (EAMV, which represents the expected λ_{max} for mutants if the effects of their singular mutational components were treated as additive). We also predict a statistically significant difference between predictions made by WT and EAMV only if WT contains enough natural variation (not based on mutants) to observe patterns of intragenic epistasis. These statistical tests assumed a Bonferroni correction for multiple tests.

Identifying known spectral tuning sites

In addition to predicting λ_{max}, we wanted to identify amino acid sites with strong effects on the phenotype, called spectral tuning sites for opsins. To do so, *deepBreaks* produces an 'importance report' of the relative importance of amino acid positions within the sequence relative to the phenotype. This report is generated for each of the top three performing models, with the addition of a column which calculates the 'mean relative importance' value of each individual position. We automated the translation of these feature representations of aligned amino acid positions compared to bovine rhodopsin for the sake of interpretability. We also included the amino acid residue identity at each corresponding position, and whether it is in one of the opsin transmembrane domains (TMD). We used this to provide us with a standardized context for analysis of the most significant positions highlighted by the models, which we could use to compare to published mutants and known spectral tuning sites. We analyzed the importance report for each model to see what positions it highlighted as most important, with an extra emphasis placed on the output for the WT models since it was the least likely to be biased by the presence of already-known mutant data (Table S9), as previous researchers often chose suspected tuning sites for mutagenesis experiments.

Results

Data Description: A genotype-phenotype database for animal opsins

VPOD is a new database, available on GitHub and in *GigaDB* [~~66~~74] that currently includes all heterologously expressed animal opsins. We refer to a subset of the database with only heterologous data as *VPOD_het_1.0*, although for version 1.0, this is synonymous with the entire database. *VPOD_het_1.0* relies on 73 publications, mainly primary sources, with dates ranging from the 1980's to 2023. The database contains opsin sequences and phenotype data from 166 unique species (counting 35 reconstructed ancestors), including fishes, amphibians, reptiles, mammals, crustaceans, and bivalves. Altogether, *VPOD_het_1.0* contains 864 unique opsin sequences and corresponding λ_{max} values. This includes 318 unique WT opsins and 546 unique experimentally mutated opsins (447 from vertebrates and 99 from invertebrates) from 82 species (73 vertebrate and 9 invertebrate species). Of the mutants, 73 are 'chimeric', meaning one or more transmembrane domains of the mutant are copied from a different opsin to replace the original. Phylogenetically, *VPOD_het_1.0* is mainly vertebrate opsins (n = 721), with only 143 unique invertebrate opsins (Figure S10). The vertebrate opsins consist of 113 UVS opsins, 167 SWS opsins, 8 MWS opsins, 83 LWS opsins, 237 Rhodopsin (Rh1), and 113 Rhodopsin-like (Rh2) opsins (Figure S10). Phenotypically, *VPOD_het_1.0* spans a range of λ_{max} values from 350-611 nm. The highest concentration of phenotype values are between 350-375 nm and 475-525 nm (Figure 1), due to the literature bias favoring characterization of UVS/SWS opsins and rhodopsins (Rh1).

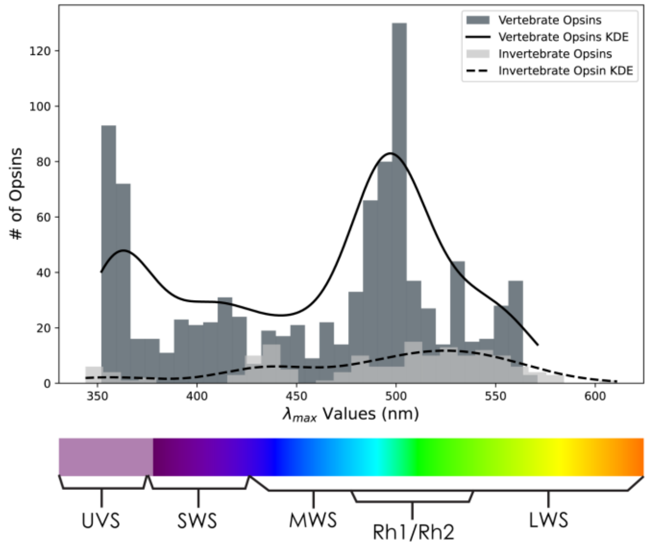

Figure 1. Histogram distributions of Vertebrate and Invertebrate Opsins and Absorbance Data - λmax - from *VPOD_het_1.1* with a scaled Kernel Density Estimate (KDE) curves overlaid to better visualize the general shape and characteristics of our λ_{max} distributions. Note an obvious data bias for vertebrate opsins, especially those with λ_{max} values between 350-375nm and 480-510 nm, probably due to focal research on UVS and Rh1 opsins.

The data used for model training strongly impacts accuracy

Several models trained with different subsets of data predicted λ_{max} with high accuracy (Table 1). The top-performing models from these subsets consistently used the same five algorithms, including the Gradient Boosting Regressor (GBR) [67,6875], Bayesian Ridge (BR) [69,7076,77], Light Gradient Boosting Machine (LGBM) [7178], Random Forest (RF) [7265], and Extreme Gradient Boosted Machine (XGB) [7360]. For example, *VPOD_vert_het_1.0* - trained with all vertebrate wild-type, mutant, and chimeric opsins - had the highest 10-fold-cross-validation (CV) R^2 (0.968) and lowest mean absolute error (MAE) (6.56 nm) of any models we compared (Figure 2). Similarly, *VPOD_wds_het_1.0*, trained with the whole dataset, had very high R^2 (0.947) and low MAE (7.47 nm). The two data subsets also shared the same five top performing models (GBR, BR, LGBM, RF, and XGB). In addition, *VPOD_wt_het_1.0* - trained without mutants and only wild type data - had a similarly high R^2 (0.902) and a low MAE (10.3 nm) when predicting unseen wild type data. Overall, this 'wild type-only' model also fared well, even when predicting mutant data not included in the model (Figure 3). While these performance metrics are impressive, it is important to remember that phylogenetic relatedness between sequences of a dataset could inflate values, like R^2 , when using random sampling for cross-validation because opsins that are more similar to those in the training data will be easier to predict, and phylogenetically clustered sequences will also be more likely to be resampled. At the same timeRoberts et al. [79] provide a discussion of alternative cross-validation strategies such as "block cross-validation" for non-independent data types, including phylogenetically related data which can help mitigate this issue. Despite overall high R^2, we noticed multiple instances where mutations that cause large shifts in λ_{max} (>10 nm) were not well-predicted by the

wildtype-only model, as indicated by large residual values for the predictions of these mutant sequences (Figure 3). We found including mutant data significantly improves predictions of λ_{max} when comparing predictions of models trained with (WDS) and without (WT) mutant data and rejecting the null hypotheses of no underlying differences between the distribution of squared error for predictions of all mutants (p-value = 9.96e-22, WDS Root Mean Square Error (RMSE) = 12.6nm, WT RMSE = 17.6nm)(Figure S11) and when predicting phenotypes of mutants with large shifts in λ_{max} (p-value = 2.29e-25, WDS Root Mean Square Error (RMSE) = 17.0nm, WT RMSE = 24.2nm)(Figure S11).

In addition to including mutant data, data availability more generally improves predictive power, with performance thresholds and plateaus depending on the genetic diversity of the training data. Overall accuracy in predicting λ_{max} for our models trained on more genotypically and phenotypically complete subsets of data (WDS, Vertebrate, WT) improves as a function of the number of sequences in a dataset, and shows an initial plateau (R^2 = ~0.80-0.90) of diminishing returns around 120-200 sequences that continues to taper off above 200 sequences (Table S2, Figure S3). Consistent with a rough performance threshold, we found models from data subsets with less than ~200 training sequences to far less accurately predict λ_{max}. For example, *VPOD_mls_het_1.0* – trained only on the 91 MWS/LWS opsins of vertebrates – and *VPOD_inv_het_1.0* – trained only on 144 invertebrate opsins - showed among the lowest R^2 (0.677 and 0.814 respectively; Table 1). For all data subsets, we found the relationship between number of sequences in a dataset and model performance best fits a reciprocal model, which is suitable when the dependent variable plateaus as the independent variable grows larger. We found the coefficients of the reciprocal equations to be different between data subsets and to increase in negative magnitude with a decrease in taxonomic/genetic diversity (the Rod model holding the largest negative value of -44). These equations do not account directly for taxonomic, genetic, or phenotypic diversity, as the raw number of genes is the value of the x-axis. Therefore, one should be cautious about applying them to predict model performance based on training data size alone.

The complicated relationship between size of training dataset and predictive power is further illustrated by models from some larger data subsets that resulted in rather poor predictions. One large dataset (884 sequences), the previously published Karyasuyama type 1 opsin dataset (*Karyasuyama_T1_ops [47]*) showed only moderate R^2 (0.804) and MAE (9.41), similar to models from the much smaller invertebrate data (Table 1). One explanation for lower predictive power could be that the very old age of T1 opsins led to a higher complexity and diversity of genotype-phenotype associations, which are not yet completely sampled enough to allow good predictions. In addition, models based on Rod, UVS/SWS, and MWS/LWS subsets tend to show lower R^2 than might be at first expected (Table S2, Figure S3), especially since these three datasets together comprise the training data for the vertebrate model (our highest performing model, R^2 = 0.968). For example, the Rod model, with 352 sequences, should have resulted in a model with an R^2 around 0.900-0.960 based on the trendlines for the WDS and Vertebrate datasets (Table S2, Figure S3) but resulted in an R^2 = 0.831. A possible explanation

for this lower R^2 value for Rod models is the small degree of variability in $\lambda_{max.}$ When variation is low, even very small differences from model predictions could lead to larger differeces in R^2. Therefore, when a data subset such as Rod opsins contains limited variability in the response variable (λ_{max}), additional metrics that are less sensitive to variance will be important, such as MAE or RMSE, which report the absolute magnitude of errors rather than the proportion of explained variance. To illustrate further, most models tested on their ability to predict the λ_{max} for a set of 25 subsampled WT-SWS opsins from VPOD, performed relatively poorly based on R^2 alone (Table S4); with the Vertebrate model (R^2 = 0.914, MAE = 7.89) demonstrating a relatively greater predictive power than all other models (Table S4, Figure S6). However, between the Vertebrate and lowest performing model (SWS model; R^2 = 0.778, MAE = 11.6nm), there is only a 3.71 nm increase in MAE; a much less dramatic perceived shift in performance than might be interpreted from R^2 alone.

Table 1. Performance Metrics Across Opsin Subsets and Top Performing Models.

Formatted Table

Name	Data Subset Version	# Seqs	Top ML Algorithm	[b]R^2	[a]MAE [nm]	[a]MAPE [%]	[b]MSE	[b]RMSE
Whole Dataset	*VPOD_wds_het_1.0*	864	LGBM	0.947	7.47	1.71	207	13.8
All Wild Types	*VPOD_wt_het_1.0*	318	Bayesian Ridge	0.902	10	2.18	297	16.5
All Mutants	*VPOD_mut_het_1.0*	546	LGBM	0.951	7.89	1.86	194	13.4
Vertebrates	*VPOD_vert_het_1.0*	721	LGBM	0.968	6.56	1.49	111	10.3
WT Vertebrates	*VPOD_wt_vert_het_1.0*	274	GBR	0.961	5.46	1.18	82.1	8.36
Invertebrates	*VPOD_inv_het_1.0*	143	LGBM	0.814	14.7	3.22	614	23.1
Rods	*VPOD_rod_het_1.0*	352	Bayesian Ridge	0.834	3.51	0.71	27.7	5.04
WT Rods	*VPOD_wt_rod_het_1.0*	157	GBR	0.783	3.57	0.72	31.9	5.11
MWS/LWS	*VPOD_mls_het_1.0*	91	XGB	0.677	8.77	1.82	317	15
UVS/SWS	*VPOD_uss_het_1.0*	280	GBR	0.821	8.02	2.06	200	13.6
WT UVS/SWS	*VPOD_wt_uss_het_1.0*	66	Adaboost	0.865	7.79	1.87	152	10.6
T1 Opsins	*Karyasuyama_T1_ops*	884	Random Forest	0.804	9.41	1.76	186	13.5

[a]*Mean absolute error* (MAE) and *mean absolute percent error* (MAPE) are in relation to the absolute error λ_{max} predictions and interpreted in the same units of 'nm'. [b]R^2, *mean square error* (MSE) or *root mean square error* (RMSE) are often interpreted as direct measures of comparing/analyzing model performance and used as training loss terms of the objective function - which measures how well the model fits the training data. One has to often balance between this and the regularization term, which controls the complexity of the model. Thus, a high performance is both simple and predictive; a tradeoff referred to as the '*bias-variance'* tradeoff.

Formatted Table

When predicting λ_{max} of thirty unseen wild-type invertebrate opsins from a separately curated MSP dataset, almost every model performed rather poorly, with exception of the WT model (n = 30, R^2 = 0.887, MAE = 17.5) (Table S4, Figure S5). The best performing model produced by the sparsely populated *'Invertebrate'* dataset could only predict unseen invertebrate opsins with an R^2 of 0.837 and MAE of 26.3 nm (Table S4, Figure S6). Until the models are trained with more invertebrate (r-opsin) data, we would not put high confidence in the estimates of λ_{max}. Furthermore, these separately curated invertebrate opsins are independent of the phylogenetic relatedness of the data used in model training, and therefore provide a less inflated estimate of the ability to predict λ_{max} compared to random resampling of training data. Because of the sparsity of invertebrate data in the training set, this result further highlights that opsins more distantly related to those in the database will be more difficult to predict.

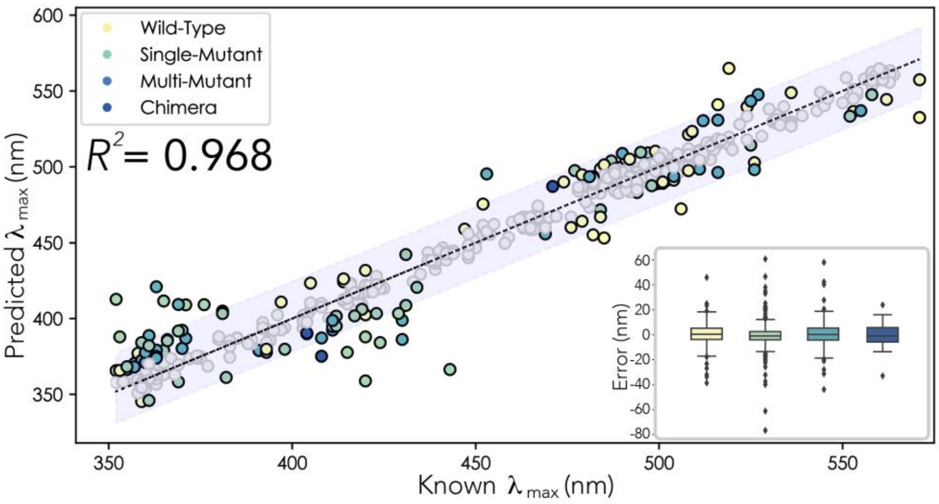

Figure 2. ML model predictions on whole Vertebrate opsin dataset, n = 721, R^2 = 0.968, MAE = 6.68nm, MAPE = 1.52. Sequences were iteratively and randomly selected to be withheld from the training dataset (n=50) to act as unseen test data. This was repeated until all sequences had been sampled once. Predictions in which the absolute difference between the 'known' and 'predicted' λ_{max} are <10nm are represented by gray dots. All predictions in which the absolute difference between the 'known' and 'predicted' λ_{max} are >10nm are represented by colored dots. Yellow dots represent WT predictions, mutants with only a single mutation are green, mutants with greater than one mutation are light-blue, and chimeric opsins are dark-blue. The light-gray bar surrounding the trend-line represents a 95% confidence interval. Inset: Box-plot distribution of prediction error for different opsin data-types from the top performing Vertebrate opsin ML model to better visualize our sources of error. Note, the median for each box-plot hovers around 0nm. Single mutations have the largest spread of error, but this is most likely due to the high abundance of that data-type over all others.

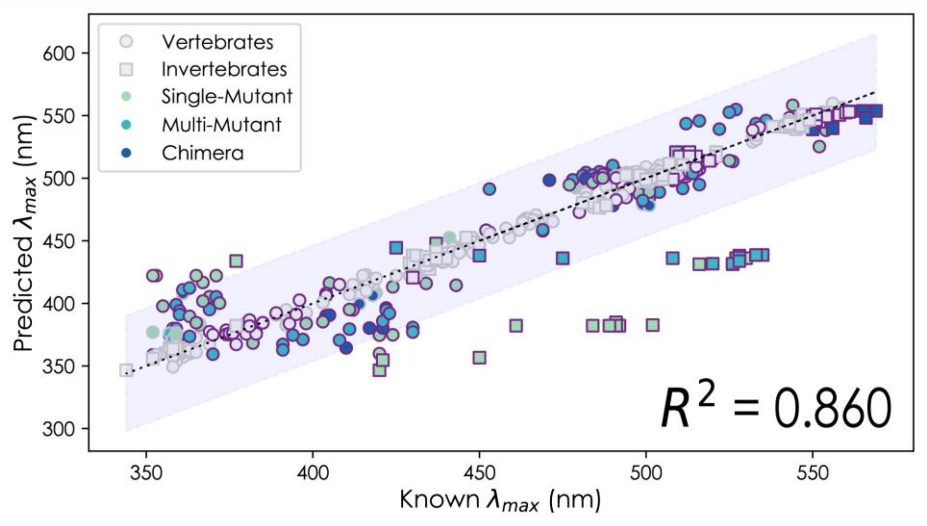

Figure 3. Scatter-plot of Wild-Type Model's λ_{max} predictions for 546 mutant opsins, with an R^2 of 0.860, MAE of 12.36 nm, and MAPE of 2.91%. Mutant predictions in which the absolute difference between the 'known' and 'predicted' λ_{max} are <10nm are represented by gray dots. All predictions in which the absolute difference between the 'known' and 'predicted' λ_{max} are >10nm are represented by colored symbols, further separated by Invertebrate (Squares) and Vertebrate (Circles) opsins. Mutants with only a single mutation are green, mutants with greater than one mutation are light-blue, and chimeric opsins are dark-blue. Mutations which caused a shift of >10nm from the WT are outlined in purple. The light-gray bar surrounding the trend-line represents a 95% confidence interval.

ML predictions of λ_{max} are comparable to phylogenetic imputation

Both ML and phylogenetic imputation were often accurate predictors of λ_{max} (Table S7). When using the same test data, ML models usually outperformed phylogenetic imputation, however slightly (Table S7), albeit using far less computational time: ML used on the order of minutes to calculate models and imputation used on the order of hours to generate opsin phylogenies. The MWS/LWS dataset was the only instance where phylogenetic imputation (R^2 = 0.784) largely outperformed ML (R^2 = 0.512). We found our implementation protocol for phylogenetic imputation required removing aligned sites with extensive gaps (for which we used Gblocks); we speculate this lessened the impacts of very short branch lengths on model fitting during imputation. To allow direct comparisons between approaches, we also used the same trimmed alignments for training ML models. Interestingly, there was a slight but noticeable decrease in ML performance following Gblocks trimming for the Invertebrate, MWS/LWS, and UVS/SWS datasets (Table S7). The R^2 of the MWS/LWS model dropped from 0.677 to 0.645, while the Invertebrate model dropped from 0.814 to 0.797 (Table S7). ML performance

remained relatively consistent after tripping for the WT, Vertebrate, WDS, SWS/UVS, and Rod models, with only a slight reduction in R^2 (< 0.01) and slight increase in MAE (+/-1nm) for the WT model. We speculate the observed differences in ML performance following Gblocks processing is due to the reduced number of features in the datasets from removing aligned sites.

ML often predicts the effects of epistatic mutations

The WDS successfully predicted three out of three individual instances of epistasis (Table S8) using sequences that were removed from the training data before using the model to predict known epistatic phenotypes. For double mutant D83N_A292S, the model predicted 485.2 nm, which was 0.2 nm off the known λ_{max} of 485 nm. If the WDS model believed the sites were additive, the resulting λ_{max} based on adding shifts of single mutants would have been much lower, at 477.5 nm. Second, for mutant F261Y_A269, the model predicted 520.0 nm, for which the known λ_{max} was 520 nm. An additive prediction would have been higher, 524 nm. Third, for mutant A164S_A269T the model predicted a λ_{max} of 515.5 nm, where the known λ_{max} was 514 nm. This is a special case in which the double mutant experiences a form of epistasis where the effect of mutation A269T (λ_{max} = 514) masks the shift otherwise caused by mutation A164S (λ_{max} = 502 nm). Thus, the model correctly predicted an instance of epistasis in which one mutation masks the effect of another.

We also queried the WT model with these same three double mutants to test the importance of mutant sequences in informing the model on epistatic interactions. However, without any mutant data at all, the WT model did not display the same abilities to predict epistasis in any instance. For the double mutant D83N_A292S, the model predicted neither the individual mutations nor the double mutant would have a significant effect on λ_{max}, and all were predicted to be 499.9 nm. For double mutants F261Y_A269 and A164S_A269T, the WT model successfully predicted all individual mutations would cause a red shift (although F261Y and A269 were >3 nm off their known λ_{max}), but incorrectly treated the mutational effects as additive for the double mutant (Table S8).

~~The~~Our broader experiment to test the predictability of epistatic effects using the WDS-minusepi model (which excluded ~~all 111 epistatic mutants during~~from training all 111 opsins with known non-additive mutational effects, which we call epistatic opsins) correctly predicted epistasis for 105/111 of the epistatic ~~mutants~~opsins with higher R^2 (0.969) and much lower RMSE (12.4 nm) than predictions by the WT model (R^2 = 0.894, RMSE = 22.3 nm~~)~~), which contains no experimentally mutated opsins, and the EAMV (R^2 = 0.878, RMSE = 29.8nm~~)~~), which ignores epistatic effects, respectively~~.~~ (Figure S12). Our test of the null hypotheses of no underlying differences between the distribution of squared error for predictions of the 111 epistatic mutants were rejected after Bonferroni correction by the WDS-minusepi model versus WT model (p-value = 1.24e-06); WDS-minusepi model versus EAMV (p-value = 2.56e-09), but not rejected for the WT model versus EAMV (p-value = 0.086) (Figure S12). Together, the large differences in RMSE and the results of the ~~WSRTs~~statistical tests strongly support the idea that the inclusion of even single mutants ~~greatly~~significantly reduces the error of ML models when

predicting epistatic interactions between mutations and that this error is significantlyalso less than the error we would observe if our models simply treated mutations as additive. Nevertheless, the insignificant difference between WT predictions and EAMV indicate there is not enough information about epistatic interactions in wild type (non-mutant) data alone to accurately predict intragenic epistasis.

ML predicts tuning sites from Wild-Type sequences alone

The full WT model and its few variants (SWS and Rod WT models) predict several previously characterized 'spectral tuning sites' - functionally demonstrated to change λ_{max} - even with no information on mutants used in the training data (Figure 4, Table S9). For the primary WT model alone, we found 15 of the top 25 amino acid sites, ranked by relative importance to the model (all ≥ 0.40), were spectral tuning sites previously characterized by mutagenesis and heterologous expression (Table S9). For example, the especially well-characterized position 308 (p308), known for its role in tuning LWS opsins, and considered to be one of the five key sites in characterizing LWS opsins under the 'Five-Site Rule' [7480], had the highest relative importance value of 1.0 when using the full WT model, indicating the amino acid identity at p308 is especially important for predicting λ_{max}. In another example, the full WT model highlighted p181, a phylogenetically conserved counterion in the retinal-opsin Schiff base interaction for all non-vertebrate opsins [75,7681,82]. Additionally, the transition from E to H at p181 (E181H) is a characteristic of the red-shifted vertebrate LWS opsins [35,7682], easily visualized in Figure 4C. When predicting λ_{max} of bovine rhodopsin with mutation E181H, the WT model predicted a red-shift compared to wild type, as observed with the natural evolution of the LWS opsin lineage. The WT SWS/UVS model similarly highlighted p113, a site functionally characterized as the counterion in the retinal-opsin Schiff base interaction for all vertebrate opsins [35,7682] and as a known spectral tuning site in SWS/UVS opsins [7783]. Moreover, even the WT Rod model, trained on a mere 157 sequences, identified p292 (Table S9), another well-characterized and conserved spectral tuning site for vertebrate rhodopsins [78–8084–86], as the site with highest relative importance to its predictions of rhodopsin λ_{max}. These spectral tuning sites are not simply conserved sites, as there is little to no correlation between amino acid sites important to model predictions (importance scores) and their relative *Shannon Entropy* [81,8287,88] scores (R^2 = 0.001). This is somewhat expected as *deepBreaks* drops all conserved ('zero-entropy') sites during preprocessing, because a site with no variation provides no important information to the effects of variation on the resulting phenotype. In addition, we predict any correlation between site conservation and model importance would be for sites that are moderately conserved and in close proximity to opsin-chromophore binding site (position 296) or binding pocket [41,42,8389].

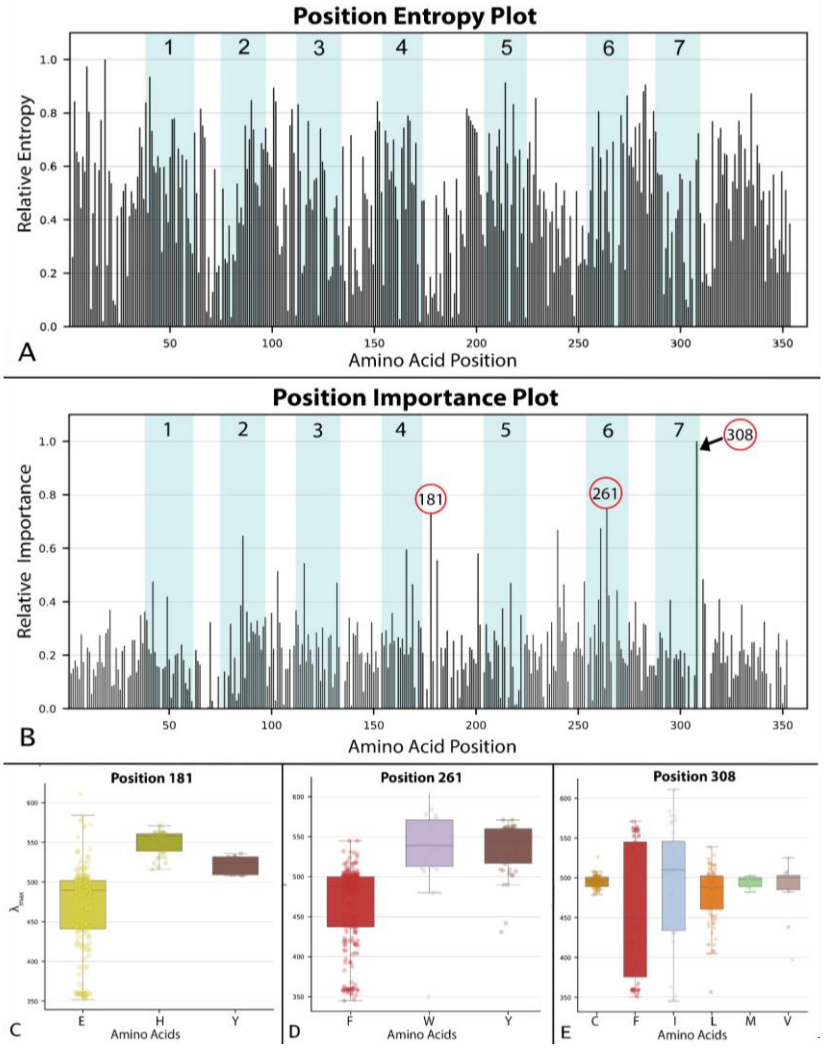

Figure 4. (**A/B**) Blue bars indicate the seven transmembrane domain regions of the bovine rhodopsin and are labeled accordingly. (**A)** Bar graph of relative entropy scores by position calculated via Shannon Entropy [62,81,82] using the multi-sequence alignment for the WT data subset. (**B**) Bar graph of relative importance by position generated via 'BayesianRidge' ML Regression Model trained on the WT opsin dataset. We interpret positions with higher relative importance as having a larger effect or weight on λ_{max} prediction. Positions 181 [35,76], 261 [80,84] and 308 [74] are highlighted because they are among the highest scoring sites and have all been previously characterized as functionally important to opsin phenotype and function.

Based on an R^2 of 0.001, there is no linear relationship between relative entropy by position and the relative importance of scores by position. (**C, D, & E**) These distribution box plots provide a visualization for which amino acid (aa) residues at a particular site are associated with different ranges of lambda max at a site of interest, ordered alphabetically, not by frequency (left to right). For a more detailed explanation on how position importance scores are calculated for different models, refer to the 'Interpretation' heading under the methods section of the deepBreaks publication [59]

Formatted: Font: Not Bold, Not Italic, Highlight

Formatted: Indent: First line: 0.5"

Discussion

To better understand methods to connect genes and their functions, we initiate *VPOD*, a database of opsin genes and corresponding spectral sensitivity phenotypes. Here, we used *VPOD_1.0* to examine the ability of ML models to predict functions of opsin genes, predict intragenic epistasis, and identify amino acid sites critical for functional changes. In all cases, ML shows promise, especially when given enough training data.

The important relationship between data availability and predictive power

The predictive power of λ_{max} is often high when using ML for opsins, and it improves with a greater amount and variety of data, albeit with diminishing returns. In particular, the number of opsin genes, their genetic diversity, and the relationship between genetic and phenotypic differences are all critical in determining predictive power. Particularly illustrative of these ideas are our analyses with and without experimentally mutated opsins. Even though we might conceive of all wild type data as natural mutants chosen by evolution, experimentally induced mutations are particularly important by often changing just one amino acid that drastically changes phenotype. As such, we found that including mutant data usually improved predictive power and conversely, predicting some phenotypes from laboratory mutagenesis was sometimes difficult without including other mutant data in model training. (Figure S11). However, relying on published mutant data alone is not optimal because it is derived from a non-random subset of species because people continue to work in established systems. Nevertheless, the genotype-phenotype landscape may be sampled well enough using high numbers of only wild type genes, as evidenced by the small difference in performance when adding mutant data to the wild type subset of well-sampled vertebrate opsins (Table 1). In contrast, adding mutant data to the sparsely sampled invertebrate opsins made a big difference. For invertebrate opsins, using only wild type data (ignoring all mutants) led to some very inaccurate predictions, especially of large phenotypic shifts caused by experimental mutagenesis (Figure 3), indicating the genotype-phenotype space is still undersampled for invertebrates. This is expected since ML learns from patterns in the underlying dataset, making predictions of distantly related opsins from those in *VPOD* more unreliable. We acknowledge this as a significant drawback for the ML approach, especially in systems or taxonomic groups lacking sufficient or reliable data. Thus, given this currently limited dataset we do not put high confidence in the λ_{max} estimates of either wild-type or mutant invertebrate (rhabdomeric) opsins. Therefore, targeting invertebrate opsins should be a high priority for new additions to *VPOD*.

A large diversity of training data is also critical for reliably predicting intragenic epistasis – the non-additive effects on a phenotype of interactions between two or more mutations within a gene – which is common [10,41,43,44,~~85,86~~91,92] and an obstacle to connecting genotypes and phenotypes [41,~~87–89~~93–95]. Our most complete datasets (whole dataset and vertebrate dataset) identified known cases of intragenic epistasis, but our models trained without experimental mutagenesis data did not. Moreover, ML demonstrates some capacity to predict the epistatic interactions between mutations, even when only provided with the single mutation components - as is evidenced by our WDS-minusepi dataset test~~.~~ (Figure S12). Similarly to the overall predictive power of λ_{max} above, predicting epistasis probably requires sufficient variation at interacting sites, which seems especially enhanced by experimentally mutated genes.

Variation in the availability of genotype-phenotype data for training not only impacts predictive power of phenotype, but also the converse; the ability to predict amino acid sites that change λ_{max}. Several models, including those trained with the whole data set (WDS), Vertebrate, and wild type (WT) data were able to successfully predict previously characterized spectral tuning sites. This is less surprising for models trained with WDS and Vertebrate datasets, due to the prevalence of data, even including mutants in the training data from experiments which specifically targeted sites thought by researchers to be functionally informative. Yet even without any targeted mutational data, three model variants using only wild type data predicted experimentally well-characterized spectral tuning/functional sites, including sites important to the stability of the opsin-chromophore interaction (P181 and P113). This demonstrates the strong potential for ML models to identify amino acid sites that govern phenotype, leading to predictions of candidate spectral tuning sites, which can be confirmed with mutagenesis experiments [38,~~79~~85] if not done so already.

ML algorithm type contributes to the predictive power of ML models.

While probably not as important as the training data used, the ML algorithm itself also impacts predictive power. All five of the best performing ML algorithms (GBR, BR, LGBM, RF, and XGB) are variants of the decision tree model architecture (Table ~~S21~~S13), and three out of five, including GBR, LGBM, and XGB, are 'gradient boosted' decision tree based ML algorithms. The gradient boosted algorithms all share the same general principles of gradient boosting [~~67,90~~75,96] including the use of ensembles of 'weak learners', usually decision trees, which work sequentially and 'gradient descent' when minimizing a loss function, to improve ML model performance. While LGBM generally performed best for predicting phenotype, it was not as effective in predicting the epistatic effects of mutations, where GBR and XGB showed the highest performance. This suggests that while LGBM excels in general phenotype prediction, the details of GBR and XGB may be better suited for epistasis prediction. The difference likely arises from the unique aspects of each algorithm's model training and settings of hyperparameters. XGB and LGBM differ from GBR by the addition of a regularization term to the objective function and in the process of ensemble tree construction during model training: GBR and XGB use level-based tree fitting while LGBM uses leaf-based tree-fitting. One

consequence of leaf-based tree construction is that due to its faster convergence/training time, it can create complex trees that are more prone to overfitting, thereby 'learning' patterns which may not exist as it constructs trees on a 'best-first basis' with a fixed number of n-terminal nodes [~~71,91~~62,78]. This creates a model that often performs well on training data but may overgeneralize, missing finer grained collinearities and interdependencies, which would be important for predicting epistasis. As such, our models might be improved by fine-tuning hyperparameters (e.g., learning rate, max-depth, and number of estimators), and the choice of which model to use will depend on the end goals of the analysis.

The assumptions of our method and limitations of ML extrapolation.

Understanding the limitations and assumptions inherent in predictive modeling is vital for accurately interpreting animal color sensitivity from opsin sequences, especially considering the impact of various factors on sensitivity beyond the opsin itself across multiple levels of biological organization. At the cell level, we assume that λ_{max} measured in cell culture (e.g., HEK293, COS cells) is the same as in living photoreceptor cells. We also assume the photopigment uses 11-cis-retinal, as all heterologously expressed opsins in *VPOD* were reconstituted using this chromophore. However, this assumption is violated in some organisms because they use 13-cis-retinal as the *in-vivo* chromophore [23,~~92,93~~97,98], which is associated with a red-shift in λ_{max} [35,~~92~~97]. At the organ-level, filters such as oil droplets in bird eyes [~~94–97~~99–102], pigments in butterfly eyes [~~98~~103], or a combination of transmissive filter and narrow band reflector in mantis shrimp larval eyes [~~99~~104], each may selectively influence light reaching photoreceptor cells and therefore animal color sensitivity. Finally, organismal responses to light involve neural processes, so even if an organism possesses the physiological ability to detect certain wavelengths, it still may not have a use for that ability. Similar considerations for all these assumptions will apply when using ML to infer other functions from other genes. In fact, many genes are more susceptible than opsins (but see [~~100~~105] showing the pressure of ocean depth may slightly affect λ_{max} phenotypes) to changes in pH, temperature, and other environmental factors [~~101~~106], such that databases compiling these gene functions should also record these parameters for use in training ML models.

Perhaps the most important caveat of using ML models to accurately predict phenotype or functional sites is that we assume there is a genotype-phenotype association that we can fit to a function and that our models were trained using ample data to capture these associations. Based on the non-linear fit between size of training data set, and model performance, we estimate that including about 200 sequences (and corresponding λ_{max}) from a taxonomically and phenotypically diverse range still provides improvements to model performance. Above 200 sequences, there is still improvement, but at a diminishing rate consistent with a reciprocal model (Table S2, Figure S3). That said, we encourage caution when extrapolating these results to predict model performance on training data size alone as the equations we used do not account directly for taxonomic, genetic, or phenotypic diversity. When using ML for predicting functionally important sites, the addition of experimental mutants to training data that cause large

phenotypic changes could heavily bias which sites are selected as 'most important' and potentially mask the importance of other sites. Here again, providing a diverse set of genotype-phenotype data should allow for the discovery of new functional sites, even when including known mutants in the training data with large phenotypic effects. Additionally, providing a large number of mutations from a limited breadth of taxa can bias model predictions as not all mutations will have the same effect on different sequences, especially if they are genetically distant. This makes it all-the-more important to consider the level of genetic diversity used to train a model when extrapolating to find potentially important functional sites (i.e., if identifying tuning sites for rhodopsins, then using a dataset of only rhodopsins would likely be the best approach, but if data is sparse or if looking for sites that may largely impact spectral tuning across opsin subfamilies, a genetically and phenotypically broad dataset may be better).

Formatted: Font: 11 pt

Conclusion

Using opsin sequence data with *deepBreaks*, we were able to train regression-based ML models to reliably predict λ_{max}, often accounting for non-additive effects of mutations on function (intragenic-epistasis), and identifying amino acid sites critical for function. We expect future work will improve these already promising results even further through at least two general directions. First, adding more data to *VPOD* will improve results, especially adding invertebrate (rhabdomeric opsins) data, as technical knowledge improves for expressing these genes [34]. In addition, phenotypic data – besides the in-vitro heterologous expression targeted here – is expansive, including λ_{max} measurements from microspectrophotometry and electroretinograms, but will take considerable effort to link these phenotypes to specific opsin genes. Second, our models can be improved to take advantage of more information. One important addition should be inclusion of physicochemical properties of the amino acids [~~102~~107], as implemented with success on a small scale of only 26 amino acid positions of microbial opsins to predict red-shifted phenotypes for optogenetics [~~103~~108]. Additionally, information on protein structure could be particularly important, such as the distance of an amino acid from the binding pocket of the chromophore [40]. While there are only a few solved crystal structures for opsins [~~104,105~~109,110] to provide such data, indirect techniques like homology modeling [~~106~~111] or neural network-based structural prediction [~~107~~112] might be usable. Other information about opsins could also be predictive, such as which G-protein the opsin signals to, allowing prediction of which amino acids dictate G-protein specificity. Opsin kinetics [e.g. ~~108~~113], or even the habitat depth at which the animal lives in the ocean, which not only influences light environment but also alters which amino acids are used in opsins [~~109~~114], could improve predictive power of the ML models. Finally, we once again caution against treating predictions of λ_{max} uncritically, because the quantity and quality of genotype-phenotype data used to train a model -- including the taxonomic, genetic, and phenotypic diversity -- is integral to the reliability of a model's predictions. Thus, ML models like those used here can be considered tools to make predictions based on summaries of existing knowledge, thereby complementing traditional experimental methods.

Potential Implications

Given the high performance demonstrated in this paper, current models are already robust enough to allow several applications. First, predicting λ_{max} will often be useful, especially for vertebrate opsins. For example, ML could provide an estimate of λ_{max} in a hogfish, whose skin expresses an opsin with unknown absorption and where λ_{max} has implications for a conceptual model of chromatophore expansion [~~110~~115]. Second, estimates of λ_{max} from opsin sequences formed part of an argument that changes in gene expression, not sequence, adapted Amazon fishes to local light environments [~~111~~116]. On broader taxonomic scales, predictions of λ_{max} from opsin sequences could expand studies of adaptation, molecular, evolution and constraint in comparison to light environments [~~112~~117]. Another application could be protein design for optogenetics - the use of genetic light sensors to induce and study expression or response pathways [~~113–115~~118–120] - including those associated with embryogenesis [~~116,117~~121,122], stress and depression [~~118–120~~123–125], or neuronal diseases [~~121,122~~126,127]. Finally, our models could be used to simulate molecular evolution under a realistic genotype-phenotype landscape. One shortcoming presently for such simulations is that our models are not trained with non-functional opsins, so even non-functional genes would be predicted to have functional λ_{max} values. A solution could be to add large-scale mutagenesis data to the training set, such as that from deep mutational scanning [~~123~~128], although the authors indicated the method is only in a proof-of-concept stage, such that the results are too noisy to be useful for model training. As the *VPOD* database expands, there will be many applications for ML, and similar techniques can also be applied to other gene families such as luciferases [16,~~124,125~~129,130].

Availability of Supporting Source Code and Requirements

Project name: The Visual Physiology Opsin Database (VPOD)
Project home page: https://github.com/VisualPhysiologyDB/visual-physiology-opsin-db
DOI: VPOD_1.0 - 10.5281/zenodo.10667840 // VPOD_1.1 - 10.5281/zenodo.12213246
Docker image of the latest version of the deepBreaks:
https://hub.docker.com/repository/docker/omicseye/deepbreaks-dc/general.
Operating system(s): Windows, MacOS, and Linux
Programming language: Python, R
Other requirements: Conda 4.9.2, deepBreaks 1.1.2, GBlocks 0.91b, MAFFT 7.520-1, MUSCLE 3.8.31, mySQL workbench 8.0.36, Python 3.9, RStudio 2023.06.2+562
License: GNU General Public License (GPL) - Version 3, 29 June 2007

Formatted: Outline numbered + Level: 1 + Numbering Style: Bullet + Aligned at: 0.25" + Indent at: 0.5"

Figure Legends

Figure 1. Histogram distributions of Vertebrate and Invertebrate Opsins and Absorbance Data - λmax - from *VPOD_het_1.1* with a scaled Kernel Density Estimate (KDE) curves overlaid to better visualize the general shape and characteristics of our λ_{max} distributions. Note an obvious data bias for vertebrate opsins, especially those with λ_{max} values between 350-375nm and 480-510 nm, probably due to focal research on UVS and Rh1 opsins.

Figure 2. ML model predictions on whole Vertebrate opsin dataset, n = 721, R^2 = 0.968, MAE = 6.68nm, MAPE = 1.52. Sequences were iteratively and randomly selected to be withheld from the training dataset (n=50) to act as unseen test data. This was repeated until all sequences had been sampled once. Predictions in which the absolute difference between the 'known' and 'predicted' λ_{max} are <10nm are represented by gray dots. All predictions in which the absolute difference between the 'known' and 'predicted' λ_{max} are >10nm are represented by colored dots. Yellow dots represent WT predictions, mutants with only a single mutation are green, mutants with greater than one mutation are light-blue, and chimeric opsins are dark-blue. The light-gray bar surrounding the trend-line represents a 95% confidence interval. Inset: Box-plot distribution of prediction error for different opsin data-types from the top performing Vertebrate opsin ML model to better visualize our sources of error. Note, the median for each box-plot hovers around 0nm. Single mutations have the largest spread of error, but this is most likely due to the high abundance of that data-type over all others.

Figure 3. Scatter-plot of Wild-Type Model's λ_{max} predictions for 546 mutant opsins, with an R^2 of 0.860, MAE of 12.36 nm, and MAPE of 2.91%. Mutant predictions in which the absolute difference between the 'known' and 'predicted' λ_{max} are <10nm are represented by gray dots. All predictions in which the absolute difference between the 'known' and 'predicted' λ_{max} are >10nm are represented by colored symbols, further separated by Invertebrate (Squares) and Vertebrate (Circles) opsins. Mutants with only a single mutation are green, mutants with greater than one mutation are light-blue, and chimeric opsins are dark-blue. Mutations which caused a shift of >10nm from the WT are outlined in purple. The light-gray bar surrounding the trend-line represents a 95% confidence interval.

Figure 4. (**A/B**) Blue bars indicate the seven transmembrane domain regions of the bovine rhodopsin and are labeled accordingly. Purple bars indicate the top three most important positions to predictions of λ_{max} by the 'BayesianRidge' ML Regression Model trained on the WT opsin dataset. (**A**) Bar graph of relative entropy scores by position calculated via Shannon Entropy [70,87,88] using the multi-sequence alignment for the WT data subset. (**B**) Bar graph of relative importance by position generated via 'BayesianRidge' ML Regression Model trained on the WT opsin dataset. We interpret positions with higher relative importance as having a larger effect or weight on λ_{max} prediction. Positions 181 [35,82], 261 [86,90] and 308 [80] are

highlighted in purple because they are among the highest scoring sites and have all been previously characterized as functionally important to opsin phenotype and function. Based on an R^2 of 0.001, there is no linear relationship between relative entropy by position and the relative importance of scores by position. (**C, D, & E**) These distribution box plots provide a visualization for which amino acid (aa) residues at a particular site are associated with different ranges of lambda max at a site of interest, ordered alphabetically, not by frequency (left to right). For a more detailed explanation on how position importance scores are calculated for different models, refer to the 'Interpretation' heading under the methods section of the deepBreaks publication [59]

Data Availability

The data set(s) supporting the results and all other code used in this article are available in the '*Visual Physiology Opsin Database*' GitHub repository (10.5281/zenodo.12213246), and at the '*Visual Physiology Opsin Database'* GigaDB repository, [pending acceptance] . All data and code is covered under a GNU General Public License (Version 3), in accordance with Open Source Initiative (OSI)-policies. DOME-ML (Data, Optimisation, Model, and Evaluation in Machine Learning) annotation, supporting the current study, is available through DOME Wizard. The Docker image provided above includes a summary of required package libraries and instructions on how to use it. Along with our existing online materials with tools used, *deepBreaks*, we also have a Jupyter notebook, instructions for Conda installation, and Code Ocean capsule (https://codeocean.com/capsule/9484494/tree/v1) for the deepBreaks. These resources should help practitioners using the main ML program we used, deepBreaks, described elsewhere, use the VPOD database for Opsin applications.

Abbreviations

Adaboost: Adaptive Boosting
AIC: Akaike Information Criterion
COS1: Monkey kidney cell line
CV: Cross-Validation
DNA: Deoxyribonucleic Acid
EAMV: ~~Expected~~Epistasis-free Additive ~~Mutational Value~~Mutation Values
ERG: Electroretinogram
GBR: Gradient Boosting Regressor
GPCR: G-Protein Coupled Receptors

Formatted: Indent: Left: -0.25", Outline numbered + Level: 1 + Numbering Style: Bullet + Aligned at: 0.25" + Indent at: 0.5"

HEK293: Human embryonic kidney cell line
ISH: In-situ Hybridization
KDE: Kernel Density Estimate
LGBM: Light Gradient Boosting Machine
LWS: Long-Wave Sensitive
MAE: Mean Absolute Error
MAPE: Mean Absolute Percentage Error
ML: Machine Learning
MSE: Mean Squared Error
MSP: Microspectrophotometry
MWS: Medium Wavelength-Sensitive
NCBI: National Center for Biotechnology Information
nm: Nanometers
RMSE: Root Mean Square Error
RNA: Ribonucleic Acid
SWS: Short-Wave Sensitive
T1: Type-1 {Microbial Opsins}
TMD: Transmembrane Domain
USS: Ultraviolet and Short-wave Sensitive
UVS: Ultraviolet-Sensitive
VPOD: Visual Physiology Opsin Database
WAG: Whelan and Goldman substitution model
WDS: Whole Dataset
~~WSRT: Wilcoxon Signed Rank Test~~
WT: Wild-Type
XGB: Extreme Gradient Boosting
λ^{max}: Lambda Max / Wavelength of light with maximal absorbance

Formatted: Indent: Left: -0.25", Outline numbered + Level: 1 + Numbering Style: Bullet + Aligned at: 0.25" + Indent at: 0.5"

Competing Interests

The authors declare they have no competing interests.

Funding

This work was supported by the US National Science Foundation grants DEB-2153773 and IOS-1754770 to THO and DEB-2109688 to AR and KAC. The funders had no role in the study design, data collection and analysis, decision to publish, or preparation of the manuscript.

Authors' Contributions

THO and KAC conceived the study; SAF performed the analysis; SAF provided online documents and software. SAF and THO drafted the original manuscript. All co-authors discussed the results and edited the final manuscript.

Acknowledgments

We acknowledge funding from the National Science Foundation DEB-2153773 and IOS-1754770 to THO and DEB-2109688 to KAC and AR. We acknowledge use of computational facilities purchased with funds from the National Science Foundation (CNS-1725797) and administered by the Center for Scientific Computing (CSC). The CSC is supported by the California NanoSystems Institute and the Materials Research Science and Engineering Center (MRSEC; NSF DMR 2308708) at UC Santa Barbara. We acknowledge R. Varney for providing technical support and expertise on the phylogenetic imputation experiments. Thanks to V. Scriven for literature searches and data entry. Thanks to A. Singh and S. Yi for advice. We acknowledge Oakley Lab for comments on an early draft of the manuscript. We thank the reviewers for helpful suggestions to improve the presentation of our work.

Citations

1. Ovsyannikova IG, Poland GA. Vaccinomics: current findings, challenges and novel approaches for vaccine development. *AAPS J*. Springer; 2011; doi: 10.1208/s12248-011-9281-x.

2. Steinbrück L, McHardy AC. Inference of Genotype–Phenotype Relationships in the Antigenic Evolution of Human Influenza A (H3N2) Viruses. *PLoS Comput Biol*. Public Library of Science; 2012; doi: 10.1371/journal.pcbi.1002492.

3. Roberts JP. Single-Cell Analysis Deepens Antibody Discovery. *Genet Eng Biotechnol News*. Mary Ann Liebert Inc; 2020; doi: 10.1089/gen.40.02.09.

4. Cobb JN, DeClerck G, Greenberg A, Clark R, McCouch S. Next-generation phenotyping: requirements and strategies for enhancing our understanding of genotype–phenotype relationships and its relevance to crop improvement. *Theor Appl Genet*. Springer; 2013; doi: 10.1007/s00122-013-2066-0.

5. Chevin L-M, Collins S, Lefèvre F. Phenotypic plasticity and evolutionary demographic responses to climate change: taking theory out to the field. *Funct Ecol*. Wiley; 2013; doi: 10.1111/j.1365-2435.2012.02043.x.

6. Franks SJ, Weber JJ, Aitken SN. Evolutionary and plastic responses to climate change in terrestrial plant populations. *Evol Appl*. Wiley Online Library; 2014; doi: 10.1111/eva.12112.

7. Gienapp P, Teplitsky C, Alho JS, Mills JA, Merilä J. Climate change and evolution: disentangling environmental and genetic responses. *Mol Ecol*. Wiley Online Library; 2008; doi: 10.1111/j.1365-294X.2007.03413.x.

8. Munday PL, Warner RR, Monro K, Pandolfi JM, Marshall DJ. Predicting evolutionary responses to climate change in the sea. *Ecol Lett*. Wiley Online Library; 2013; doi: 10.1111/ele.12185.

9. Singhal A, Simmons M, Lu Z. Text Mining Genotype-Phenotype Relationships from Biomedical Literature for Database Curation and Precision Medicine. *PLoS Comput Biol*. journals.plos.org; 2016; doi: 10.1371/journal.pcbi.1005017.

10. Kemble H, Nghe P, Tenaillon O. Recent insights into the genotype-phenotype relationship from massively parallel genetic assays. *Evol Appl*. Wiley; 2019; doi: 10.1111/eva.12846.

11. Dikicioglu D, Pir P, Oliver SG. Predicting complex phenotype-genotype interactions to enable yeast engineering: Saccharomyces cerevisiae as a model organism and a cell factory. *Biotechnol J*. Wiley; 2013; doi: 10.1002/biot.201300138.

12. Leung MKK, Delong A, Alipanahi B, Frey BJ. Machine Learning in Genomic Medicine: A Review of Computational Problems and Data Sets. *Proc IEEE*. IEEE; 2016; doi: 10.1109/JPROC.2015.2494198.

13. Guzzetta G, Jurman G, Furlanello C. A machine learning pipeline for quantitative phenotype prediction from genotype data. *BMC Bioinformatics*. 2010; doi: 10.1186/1471-2105-11-S8-S3.

14. Lee B, Zhang S, Poleksic A, Xie L. Heterogeneous Multi-Layered Network Model for Omics Data Integration and Analysis. *Front Genet*. 2019; doi: 10.3389/fgene.2019.01381.

15. Lee Y-C, Christensen JJ, Parnell LD, Smith CE, Shao J, McKeown NM, et al.. Using Machine Learning to Predict Obesity Based on Genome-Wide and Epigenome-Wide Gene-Gene and Gene-Diet Interactions. *Front Genet*. 2021; doi: 10.3389/fgene.2021.783845.

16. Yeh AH-W, Norn C, Kipnis Y, Tischer D, Pellock SJ, Evans D, et al.. De novo design of luciferases using deep learning. *Nature*. 2023; doi: 10.1038/s41586-023-05696-3.

17. Brandes N, Goldman G, Wang CH, Ye CJ, Ntranos V. Genome-wide prediction of disease variant effects with a deep protein language model. *Nat Genet*. 2023; doi: 10.1038/s41588-023-01465-0.

18. Pinto MF, Oliveira H, Batista S, Cruz L, Pinto M, Correia I, et al.. Prediction of disease progression and outcomes in multiple sclerosis with machine learning. *Sci Rep*. nature.com; 2020; doi: 10.1038/s41598-020-78212-6.

19. Himmelstein DS, Baranzini SE. Heterogeneous Network Edge Prediction: A Data Integration Approach to Prioritize Disease-Associated Genes. *PLoS Comput Biol*. 2015; doi: 10.1371/journal.pcbi.1004259.

20. Sheikh N: Identification and classification of wildlife from camera-trap images using machine learning and computer vision. norma.ncirl.ie; https://norma.ncirl.ie/4283/1/nawazsheikh.pdf (2020). Accessed 2023 Dec 12.

21. Vélez J, McShea W, Shamon H, Castiblanco-Camacho PJ, Tabak MA, Chalmers C, et al.. An evaluation of platforms for processing camera-trap data using artificial intelligence. *Methods Ecol Evol*. Wiley; 2023; doi: 10.1111/2041-210x.14044.

22. Kutugata M, Baumgardt J, Goolsby JA, Racelis AE. Automatic Camera-Trap Classification Using Wildlife-Specific Deep Learning in Nilgai Management. *Journal of Fish and Wildlife Management*. Allen Press; 2021; doi: 10.3996/JFWM-20-076.

23. Terakita A. The opsins. *Genome Biol*. 2005; doi: 10.1186/gb-2005-6-3-213.

24. Govardovskii VI, Fyhrquist N, Reuter T, Kuzmin DG, Donner K. In search of the visual pigment template. *Vis Neurosci*. 2000; doi: 10.1017/s0952523800174036.

25. Jacobs GH, Neitz J, Krogh K. Electroretinogram flicker photometry and its applications. *J Opt Soc Am A Opt Image Sci Vis*. opg.optica.org; 1996; doi: 10.1364/josaa.13.000641.

26. Thomas MM, Lamb TD. Light adaptation and dark adaptation of human rod photoreceptors measured from the a-wave of the electroretinogram. *J Physiol*. Wiley Online Library; 1999; doi: 10.1111/j.1469-7793.1999.0479p.x.

27. Rocha FA de F, Gomes BD, Silveira LC de L, Martins SL, Aguiar RG, de Souza JM, et al.. Spectral Sensitivity Measured with Electroretinogram Using a Constant Response Method. *PLoS One*. journals.plos.org; 2016; doi: 10.1371/journal.pone.0147318.

28. Liebman PA. Microspectrophotometry of Photoreceptors. In: Abrahamson EW, Baumann C, Bridges CDB, Crescitelli F, Dartnall HJA, Eakin RM, et al., editors. *Photochemistry of Vision*. Berlin, Heidelberg: Springer Berlin Heidelberg;

29. Yewers MS, McLean CA, Moussalli A, Stuart-Fox D, Bennett ATD, Knott B. Spectral sensitivity of cone photoreceptors and opsin expression in two colour-divergent lineages of the lizard Ctenophorus decresii. *J Exp Biol*. journals.biologists.com; 2015; doi: 10.1242/jeb.131854.

30. Kojima D, Fukada Y. Spectroscopic Analysis of Wavelength Sensitivities of Opsin-Type Photoreceptor Proteins. In: Hirota T, Hatori M, Panda S, editors. *Circadian Clocks*. New York, NY: Springer US;

31. Carlson SD. Microspectrophotometry of visual pigments. *Q Rev Biophys*. cambridge.org; 1972; doi: 10.1017/s0033583500000986.

32. Bowmaker JK. Microspectrophotometry of vertebrate photoreceptors. A brief review. *Vision Res*. Elsevier; 1984; doi: 10.1016/0042-6989(84)90322-5.

33. Merbs SL, Nathans J. Absorption spectra of human cone pigments. *Nature*. 1992; doi: 10.1038/356433a0.

34. Liénard MA, Valencia-Montoya WA, Pierce NE. Molecular advances to study the function, evolution and spectral tuning of arthropod visual opsins. *Philos Trans R Soc Lond B Biol Sci*. royalsocietypublishing.org; 2022; doi: 10.1098/rstb.2021.0279.

35. Hagen JFD, Roberts NS, Johnston RJ Jr. The evolutionary history and spectral tuning of vertebrate visual opsins. *Dev Biol*. 2023; doi: 10.1016/j.ydbio.2022.10.014.

36. Yokoyama S, Radlwimmer FB. The molecular genetics and evolution of red and green color vision in vertebrates. *Genetics*. 2001; doi: 10.1093/genetics/158.4.1697.

37. Bloch NI. The evolution of opsins and color vision: connecting genotype to a complex

phenotype. *Acta Biolo Colomb*. Universidad Nacional de Colombia; 2016; doi: 10.15446/abc.v21n3.53907.

38. Rajamani R, Lin Y-L, Gao J. The opsin shift and mechanism of spectral tuning in rhodopsin. *J Comput Chem*. Wiley Online Library; 2011; doi: 10.1002/jcc.21663.

39. Hárosi FI. An analysis of two spectral properties of vertebrate visual pigments. *Vision Res*. 1994; doi: 10.1016/0042-6989(94)90134-1.

40. Wang W, Geiger JH, Borhan B. The photochemical determinants of color vision: revealing how opsins tune their chromophore's absorption wavelength. *Bioessays*. 2014; doi: 10.1002/bies.201300094.

41. Smedley GD, McElroy KE, Feller KD, Serb JM. Additive and epistatic effects influence spectral tuning in molluscan retinochrome opsin. *J Exp Biol*. journals.biologists.com; 2022; doi: 10.1242/jeb.242929.

42. Nathans J. Determinants of visual pigment absorbance: identification of the retinylidene Schiff's base counterion in bovine rhodopsin. *Biochemistry*. 1990; doi: 10.1021/bi00493a034.

43. Yokoyama S, Xing J, Liu Y, Faggionato D, Altun A, Starmer WT. Epistatic adaptive evolution of human color vision. *PLoS Genet*. journals.plos.org; 2014; doi: 10.1371/journal.pgen.1004884.

44. Yokoyama S, Altun A, Jia H, Yang H, Koyama T, Faggionato D, et al.. Adaptive evolutionary paths from UV reception to sensing violet light by epistatic interactions. *Sci Adv*. science.org; 2015; doi: 10.1126/sciadv.1500162.

45. Patel D, Barnes JE, Davies WIL, Stenkamp DL, Patel JS. Short-wavelength-sensitive 2 (Sws2) visual photopigment models combined with atomistic molecular simulations to predict spectral peaks of absorbance. *PLoS Comput Biol*. 2020; doi: 10.1371/journal.pcbi.1008212.

46. Patel JS, Brown CJ, Ytreberg FM, Stenkamp DL. Predicting peak spectral sensitivities of vertebrate cone visual pigments using atomistic molecular simulations. *PLoS Comput Biol*. 2018; doi: 10.1371/journal.pcbi.1005974.

47. Karasuyama M, Inoue K, Nakamura R, Kandori H, Takeuchi I. Understanding Colour Tuning Rules and Predicting Absorption Wavelengths of Microbial Rhodopsins by Data-Driven Machine-Learning Approach. *Sci Rep*. 2018; doi: 10.1038/s41598-018-33984-w.

48. Adam ZR, Schwieterman EW, Kacar B. Earliest photic zone niches probed by ancestral microbial rhodopsins. *Mol Biol*. academic.oup.com; 2022;

49. Longcore T. A compendium of photopigment peak sensitivities and visual spectral response curves of terrestrial wildlife to guide design of outdoor nighttime lighting. *Basic Appl Ecol*. 2023; doi: 10.1016/j.baae.2023.09.002.

50. Schott RK, Fujita MK, Streicher JW, Gower DJ, Thomas KN, Loew ER, et al.. Diversity and

Evolution of Frog Visual Opsins: Spectral Tuning and Adaptation to Distinct Light Environments. *Mol Biol Evol*. 2024; doi: 10.1093/molbev/msae049.

51. Schott RK, Perez L, Kwiatkowski MA, Imhoff V, Gumm JM. Evolutionary analyses of visual opsin genes in frogs and toads: Diversity, duplication, and positive selection. *Ecol Evol*. 2022; doi: 10.1002/ece3.8595.

52. Schweikert LE, Fitak RR, Caves EM, Sutton TT, Johnsen S. Spectral sensitivity in ray-finned fishes: diversity, ecology and shared descent. *J Exp Biol*. 2018; doi: 10.1242/jeb.189761.

53. Schweikert LE, Caves EM, Solie SE, Sutton TT, Johnsen S. Variation in rod spectral sensitivity of fishes is best predicted by habitat and depth. *J Fish Biol*. 2019; doi: 10.1111/jfb.13859.

54. Molina-Venegas R, Moreno-Saiz JC, Castro Parga I, Davies TJ, Peres-Neto PR, Rodríguez MÁ. Assessing among-lineage variability in phylogenetic imputation of functional trait datasets. *Ecography* . Wiley; 2018; doi: 10.1111/ecog.03480.

55. Garland T Jr, Ives AR. Using the Past to Predict the Present: Confidence Intervals for Regression Equations in Phylogenetic Comparative Methods. *Am Nat*. 2000; doi: 10.1086/303327.

56. Katoh K, Standley DM. MAFFT multiple sequence alignment software version 7: improvements in performance and usability. *Mol Biol Evol*. academic.oup.com; 2013; doi: 10.1093/molbev/mst010.

57. Edgar RC. MUSCLE: multiple sequence alignment with high accuracy and high throughput. *Nucleic Acids Res*. academic.oup.com; 2004; doi: 10.1093/nar/gkh340.

58. Castresana J. Selection of conserved blocks from multiple alignments for their use in phylogenetic analysis. *Mol Biol Evol*. 2000; doi: 10.1093/oxfordjournals.molbev.a026334.

59. Baghbanzadeh M, Dawson T, Sayoldin B, Oakley T, Crandall K, Rahnavard A. DeepBreaks: A machine learning tool for identifying and prioritizing genotype-phenotype associations. Research Square.

~~60. Hunter JD. Matplotlib: A 2D Graphics Environment.~~ 60. Chen T, He T, Benesty M, Khotilovich V, Tang Y, Cho H, et al.. Xgboost: extreme gradient boosting. *R package version 0 4-2*. cran.ms.unimelb.edu.au; 1:1–42015;

61. Sibindi R, Mwangi RW, Waititu AG. A boosting ensemble learning based hybrid light gradient boosting machine and extreme gradient boosting model for predicting house prices. *Eng Rep*. Wiley; 2023; doi: 10.1002/eng2.12599.

62. Ke G, Meng Q, Finley T, Wang T, Chen W, Ma W, et al.. LightGBM: A highly efficient Gradient Boosting Decision Tree. *Adv Neural Inf Process Syst*. :3146–542017;

63. Schapire RE. Explaining AdaBoost. In: Schölkopf B, Luo Z, Vovk V, editors. *Empirical*

Inference: Festschrift in Honor of Vladimir N Vapnik. Berlin, Heidelberg: Springer Berlin Heidelberg;

64. Rigatti SJ. Random Forest. *J Insur Med*. meridian.allenpress.com; 2017; doi: 10.17849/insm-47-01-31-39.1.

65. Segal MR. Machine Learning Benchmarks and Random Forest Regression. escholarship.org; 2004;

66. Pedregosa F, Varoquaux G, Gramfort A, Michel V, Thirion B, Grisel O, et al.. Scikit-learn: Machine Learning in Python. *J Mach Learn Res*. JMLR.org; 12:2825–302011;

67. Prettenhofer P, Louppe G. Gradient Boosted Regression Trees in Scikit-Learn. *PyData 2014*. orbi.uliege.be;

68. Hunter JD. Matplotlib: A 2D Graphics Environment. *Comput Sci Eng*. IEEE; May-June 2007; doi: 10.1109/MCSE.2007.55.

~~61~~69. Damian Riina M, Stambaugh C, Stambaugh N, Huber KE. Chapter 28 - Continuous variable analyses: t-test, Mann–Whitney, Wilcoxin rank. In: Eltorai AEM, Bakal JA, Kim DW, Wazer DE, editors. *Translational Radiation Oncology*. Academic Press;

~~62~~70. Gommers R, Virtanen P, Burovski E, Weckesser W, Oliphant TE, Haberland M, et al.. scipy/scipy: SciPy 1.9.0. Zenodo. Zenodo;

~~63~~71. Silva JO, Orellana ETV, Torres M. Development of a Parallel Version of PhyML 3.0 Using Shared Memory. *IEEE Latin America Transactions*. IEEE; 2017; doi: 10.1109/TLA.2017.7912593.

~~64~~72. Le SQ, Gascuel O. An improved general amino acid replacement matrix. *Mol Biol Evol*. academic.oup.com; 2008; doi: 10.1093/molbev/msn067.

~~65~~73. Revell LJ. phytools: an R package for phylogenetic comparative biology (and other things). *Methods Ecol Evol*. Wiley; 2012; doi: 10.1111/j.2041-210x.2011.00169.x.

~~66~~74. Sneddon TP, Li P, Edmunds SC. GigaDB: announcing the GigaScience database. *Gigascience*. 2012; doi: 10.1186/2047-217X-1-11.

~~67~~75. Friedman JH. Greedy Function Approximation: A Gradient Boosting Machine. *Ann Stat*. Institute of Mathematical Statistics; 29:1189–2322001;

~~68. Prettenhofer P, Louppe G. Gradient Boosted Regression Trees in Scikit-Learn.~~ 76. Bedoui A, Lazar NA. Bayesian empirical likelihood for ridge and lasso regressions. ~~*PyData 2014*. orbi.uliege.be;~~

~~69. Bedoui A, Lazar NA. Bayesian empirical likelihood for ridge and lasso regressions.~~ *Comput Stat Data Anal*. Elsevier; 2020; doi: 10.1016/j.csda.2020.106917.

7077. Karabatsos G. Fast Marginal Likelihood Estimation of the Ridge Parameter(s) in Ridge Regression and Generalized Ridge Regression for Big Data. arXiv [stat.ME].

7178. Fan J, Ma X, Wu L, Zhang F, Yu X, Zeng W. Light Gradient Boosting Machine: An efficient soft computing model for estimating daily reference evapotranspiration with local and external meteorological data. *Agric Water Manage*. Elsevier; 2019; doi: 10.1016/j.agwat.2019.105758.

72. Segal MR. Machine Learning Benchmarks and Random Forest Regression. escholarship.org; 2004;

73. Chen T, He T, Benesty M, Khotilovich V, Tang Y, Cho H, et al.. Xgboost: extreme gradient boosting. 79. Roberts DR, Bahn V, Ciuti S, Boyce MS, Elith J, Guillera-Arroita G, et al.. Cross-validation strategies for data with temporal, spatial, hierarchical, or phylogenetic structure. *Ecography* . Wiley; 2017; doi: 10.1111/ecog.02881.

80. Yokoyama S, Radlwimmer FB. The “five-sites” rule and the evolution of red and green color vision in mammals. *R package version 0.4-2*. cran.ms.unimelb.edu.au; 1:1–42015;

74. Yokoyama S, Radlwimmer FB. The “five-sites” rule and the evolution of red and green color vision in mammals. *Mol Biol Evol*. 1998; doi: 10.1093/oxfordjournals.molbev.a025956.

7581. Shichida Y, Matsuyama T. Evolution of opsins and phototransduction. *Philos Trans R Soc Lond B Biol Sci*. 2009; doi: 10.1098/rstb.2009.0051.

7682. Terakita A, Koyanagi M, Tsukamoto H, Yamashita T, Miyata T, Shichida Y. Counterion displacement in the molecular evolution of the rhodopsin family. *Nat Struct Mol Biol*. 2004; doi: 10.1038/nsmb731.

7783. Shi Y, Radlwimmer FB, Yokoyama S. Molecular genetics and the evolution of ultraviolet vision in vertebrates. *Proc Natl Acad Sci U S A*. 2001; doi: 10.1073/pnas.201257398.

7884. Sugawara T, Terai Y, Imai H, Turner GF, Koblmüller S, Sturmbauer C, et al.. Parallelism of amino acid changes at the RH1 affecting spectral sensitivity among deep-water cichlids from Lakes Tanganyika and Malawi. *Proc Natl Acad Sci U S A*. 2005; doi: 10.1073/pnas.0405302102.

7985. Takenaka N, Yokoyama S. Mechanisms of spectral tuning in the RH2 pigments of Tokay gecko and American chameleon. *Gene*. 2007; doi: 10.1016/j.gene.2007.04.036.

8086. Yokoyama S, Tada T, Zhang H, Britt L. Elucidation of phenotypic adaptations: Molecular analyses of dim-light vision proteins in vertebrates. *Proc Natl Acad Sci U S A*. 2008; doi: 10.1073/pnas.0802426105.

8187. Shannon C. A mathematical theory of communication (1948). direct.mit.edu; 2021; doi: 10.7551/MITPRESS/12274.003.0014.

8288. Ramazzotti M, Degl’Innocenti D, Manao G, Ramponi G. Entropy calculator: getting the best from your multiple protein alignments. *Ital J Biochem*. researchgate.net; 53:16–222004;

~~83~~89. Lin SW, Sakmar TP. Colour tuning mechanisms of visual pigments. *Novartis Found Symp*. 1999; doi: 10.1002/9780470515693.ch8.

~~84~~90. Chan T, Lee M, Sakmar TP. Introduction of hydroxyl-bearing amino acids causes bathochromic spectral shifts in rhodopsin. Amino acid substitutions responsible for red-green color pigment spectral tuning. *J Biol Chem*. 267:9478–801992;

~~85~~91. Orgogozo V, Morizot B, Martin A. The differential view of genotype–phenotype relationships. *Front Genet*. frontiersin.org; 2015; doi: 10.3389/fgene.2015.00179.

~~86~~92. Baldwin MW, Ko M-C. Functional evolution of vertebrate sensory receptors. *Horm Behav*. Elsevier; 2020; doi: 10.1016/j.yhbeh.2020.104771.

~~87~~93. Park Y, Metzger BPH, Thornton JW. Epistatic drift causes gradual decay of predictability in protein evolution. *Science*. science.org; 2022; doi: 10.1126/science.abn6895.

~~88~~94. Lyons DM, Zou Z, Xu H, Zhang J. Idiosyncratic epistasis creates universals in mutational effects and evolutionary trajectories. *Nat Ecol Evol*. nature.com; 2020; doi: 10.1038/s41559-020-01286-y.

~~89~~95. Gonzalez Somermeyer L, Fleiss A, Mishin AS, Bozhanova NG, Igolkina AA, Meiler J, et al.. Heterogeneity of the GFP fitness landscape and data-driven protein design. *Elife*. elifesciences.org; 2022; doi: 10.7554/eLife.75842.

~~90~~96. Friedman JH. Stochastic gradient boosting. *Comput Stat Data Anal*. 2002; doi: 10.1016/S0167-9473(01)00065-2.

~~91. Ke G, Meng Q, Finley T, Wang T, Chen W, Ma W, et al.. LightGBM: A highly efficient Gradient Boosting Decision Tree.~~ 97. Sekharan S, Morokuma K. Why 11-cis-retinal? Why not 7-cis-, 9-cis-, or 13-cis-retinal in the eye? ~~*Adv Neural Inf Process Syst*. :3146–542017;~~

~~92. Sekharan S, Morokuma K. Why 11-cis-retinal? Why not 7-cis-, 9-cis-, or 13-cis-retinal in the eye?~~ *J Am Chem Soc*. ACS Publications; 2011; doi: 10.1021/ja208789h.

~~93~~98. Buczyłko J, Saari JC, Crouch RK, Palczewski K. Mechanisms of opsin activation. *J Biol Chem*. ASBMB; 1996; doi: 10.1074/jbc.271.34.20621.

~~94~~99. Das D, Wilkie SE, Hunt DM, Bowmaker JK. Visual pigments and oil droplets in the retina of a passerine bird, the canary Serinus canaria: microspectrophotometry and opsin sequences. *Vision Res*. 1999; doi: 10.1016/s0042-6989(99)00023-1.

~~95~~100. Toomey MB, Collins AM, Frederiksen R, Cornwall MC, Timlin JA, Corbo JC. A complex carotenoid palette tunes avian colour vision. *J R Soc Interface*. royalsocietypublishing.org; 2015; doi: 10.1098/rsif.2015.0563.

~~96~~101. Hart NS, Vorobyev M. Modelling oil droplet absorption spectra and spectral sensitivities of bird cone photoreceptors. *J Comp Physiol A Neuroethol Sens Neural Behav Physiol*. Springer; 2005; doi: 10.1007/s00359-004-0595-3.

~~97~~102. Toomey MB, Corbo JC. Evolution, Development and Function of Vertebrate Cone Oil Droplets. *Front Neural Circuits*. frontiersin.org; 2017; doi: 10.3389/fncir.2017.00097.

~~98~~103. Arikawa K, Stavenga D. Random array of colour filters in the eyes of butterflies. *J Exp Biol*. 1997; doi: 10.1242/jeb.200.19.2501.

~~99~~104. Feller KD, Wilby D, Jacucci G, Vignolini S, Mantell J, Wardill TJ, et al.. Long-Wavelength Reflecting Filters Found in the Larval Retinas of One Mantis Shrimp Family (Nannosquillidae). *Curr Biol*. 2019; doi: 10.1016/j.cub.2019.07.070.

~~100~~105. Partridge JC, White EM, Douglas RH. The effect of elevated hydrostatic pressure on the spectral absorption of deep-sea fish visual pigments. *J Exp Biol*. 2006; doi: 10.1242/jeb.01984.

~~101~~106. Ogbunugafor CB, Wylie CS, Diakite I, Weinreich DM, Hartl DL. Adaptive Landscape by Environment Interactions Dictate Evolutionary Dynamics in Models of Drug Resistance. *PLoS Comput Biol*. 2016; doi: 10.1371/journal.pcbi.1004710.

~~102~~107. Woolley S, Johnson J, Smith MJ, Crandall KA, McClellan DA. TreeSAAP: selection on amino acid properties using phylogenetic trees. *Bioinformatics*. 2003; doi: 10.1093/bioinformatics/btg043.

~~103~~108. Inoue K, Karasuyama M, Nakamura R, Konno M, Yamada D, Mannen K, et al.. Exploration of natural red-shifted rhodopsins using a machine learning-based Bayesian experimental design. *Commun Biol*. 2021; doi: 10.1038/s42003-021-01878-9.

~~104~~109. Palczewski K, Kumasaka T, Hori T, Behnke CA, Motoshima H, Fox BA, et al.. Crystal structure of rhodopsin: A G protein-coupled receptor. *Science*. 2000; doi: 10.1126/science.289.5480.739.

~~105~~110. Murakami M, Kouyama T. Crystal structure of squid rhodopsin. *Nature*. 2008; doi: 10.1038/nature06925.

~~106~~111. Briscoe AD. Homology modeling suggests a functional role for parallel amino acid substitutions between bee and butterfly red- and green-sensitive opsins. *Mol Biol Evol*. 2002; doi: 10.1093/oxfordjournals.molbev.a004158.

~~107~~112. Jumper J, Evans R, Pritzel A, Green T, Figurnov M, Ronneberger O, et al.. Highly accurate protein structure prediction with AlphaFold. *Nature*. 2021; doi: 10.1038/s41586-021-03819-2.

~~108~~113. Van Nynatten A, Castiglione GM, de A Gutierrez E, Lovejoy NR, Chang BSW. Recreated Ancestral Opsin Associated with Marine to Freshwater Croaker Invasion Reveals Kinetic and Spectral Adaptation. *Mol Biol Evol*. 2021; doi: 10.1093/molbev/msab008.

~~109~~114. Porter ML, Roberts NW, Partridge JC. Evolution under pressure and the adaptation of visual pigment compressibility in deep-sea environments. *Mol Phylogenet Evol*. Elsevier; 2016; doi: 10.1016/j.ympev.2016.08.007.

~~110~~115. Schweikert LE, Bagge LE, Naughton LF, Bolin JR, Wheeler BR, Grace MS, et al.. Dynamic light filtering over dermal opsin as a sensory feedback system in fish color change. *Nat Commun*. Nature Publishing Group UK London; 14:46422023;

~~111~~116. Borghezan E de A, da Silva Pires TH, Zuanon J, Sugiura H, Kohshima S, Kishida T. Unstable environmental conditions constrain the fine-tune between opsin sensitivity and underwater light in an Amazon forest stream fish. *J Evol Biol*. Oxford University Press; 2024; doi: 10.1093/jeb/voae001.

~~112~~117. Murphy MJ, Westerman EL. Evolutionary history limits species' ability to match colour sensitivity to available habitat light. *Proc Biol Sci*. 2022; doi: 10.1098/rspb.2022.0612.

~~113~~118. Kwon E, Heo WD. Optogenetic tools for dissecting complex intracellular signaling pathways. *Biochem Biophys Res Commun*. 2020; doi: 10.1016/j.bbrc.2019.12.132.

~~114~~119. Mukherjee A, Repina NA, Schaffer DV, Kane RS. Optogenetic tools for cell biological applications. J. Thorac. Dis.

~~115~~120. Tischer D, Weiner OD. Illuminating cell signalling with optogenetic tools. *Nat Rev Mol Cell Biol*. 2014; doi: 10.1038/nrm3837.

~~116~~121. Kaur P, Saunders TE, Tolwinski NS. Coupling optogenetics and light-sheet microscopy, a method to study Wnt signaling during embryogenesis. *Sci Rep*. nature.com; 2017; doi: 10.1038/s41598-017-16879-0.

~~117~~122. Fan H, Barnes C, Hwang H, Zhang K, Yang J. Precise modulation of embryonic development through optogenetics. *Genesis*. Wiley Online Library; 2022; doi: 10.1002/dvg.23505.

~~118~~123. Sparta DR, Jennings JH, Ung RL, Stuber GD. Optogenetic strategies to investigate neural circuitry engaged by stress. *Behav Brain Res*. Elsevier; 2013; doi: 10.1016/j.bbr.2013.05.007.

~~119~~124. Belzung C, Turiault M, Griebel G. Optogenetics to study the circuits of fear- and depression-like behaviors: a critical analysis. *Pharmacol Biochem Behav*. Elsevier; 2014; doi: 10.1016/j.pbb.2014.04.002.

~~120~~125. Muir J, Lopez J, Bagot RC. Wiring the depressed brain: optogenetic and chemogenetic circuit interrogation in animal models of depression. *Neuropsychopharmacology*. nature.com; 2019; doi: 10.1038/s41386-018-0291-6.

~~121~~126. LaLumiere RT. A new technique for controlling the brain: optogenetics and its potential for use in research and the clinic. *Brain Stimul*. 2011; doi: 10.1016/j.brs.2010.09.009.

~~122~~127. Montagni E, Resta F, Mascaro ALA, Pavone FS. Optogenetics in Brain Research: From a Strategy to Investigate Physiological Function to a Therapeutic Tool. *Photonics*. Multidisciplinary Digital Publishing Institute; 2019; doi: 10.3390/photonics6030092.

123128. Penn WD, McKee AG, Kuntz CP, Woods H, Nash V, Gruenhagen TC, et al.. Probing biophysical sequence constraints within the transmembrane domains of rhodopsin by deep mutational scanning. *Sci Adv*. 2020; doi: 10.1126/sciadv.aay7505.

124129. Hensley NM, Ellis EA, Leung NY, Coupart J, Mikhailovsky A, Taketa DA, et al.. Selection, drift, and constraint in cypridinid luciferases and the diversification of bioluminescent signals in sea fireflies. *Mol Ecol*. 2021; doi: 10.1111/mec.15673.

125130. Schenkmayerova A, Pinto GP, Toul M, Marek M, Hernychova L, Planas-Iglesias J, et al.. Engineering the protein dynamics of an ancestral luciferase. *Nat Commun*. 2021; doi: 10.1038/s41467-021-23450-z.

Figure 1

Click here to access/download;Figure;Figure 1 - opsin_histogram2.png

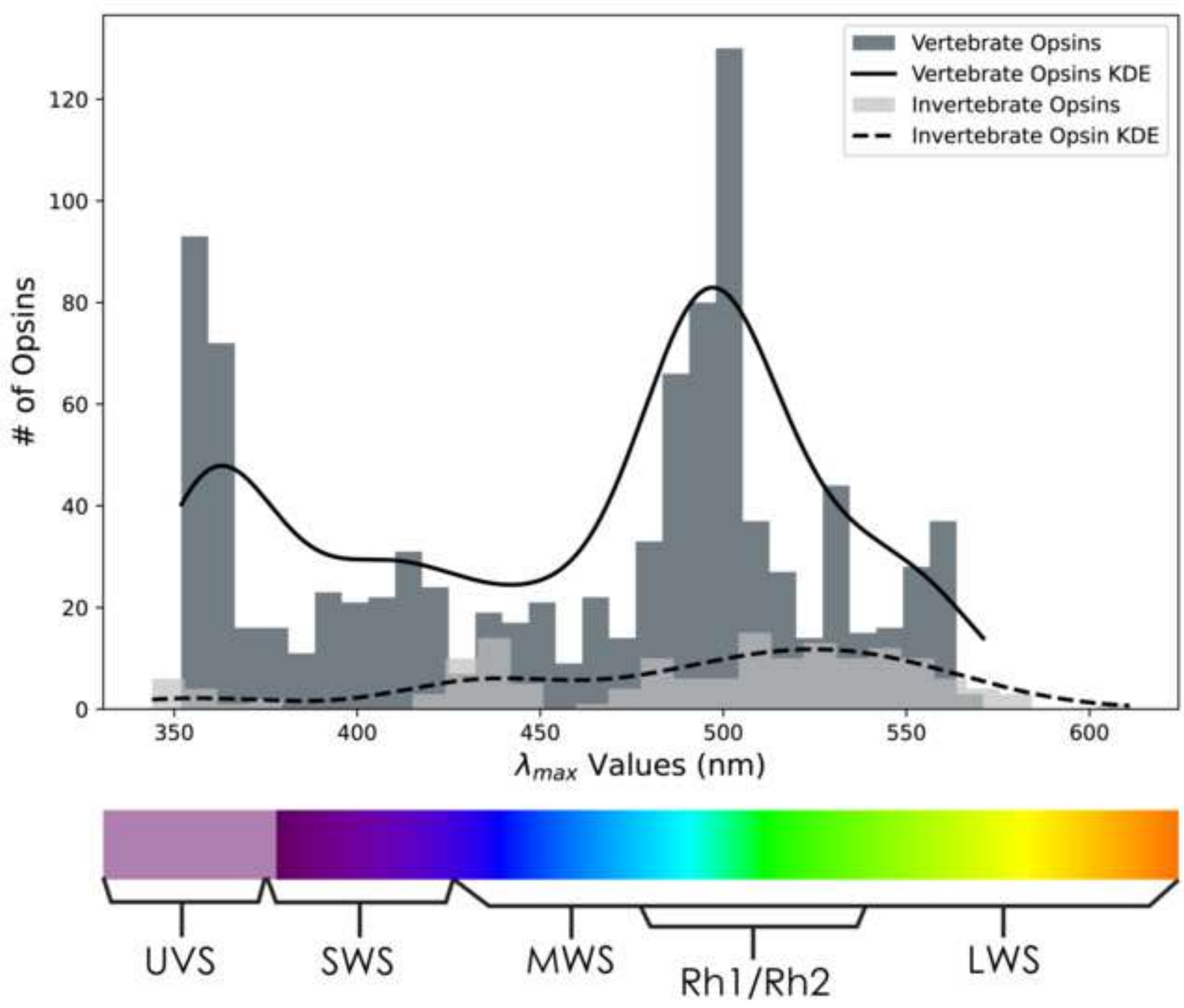

Figure 2

Click here to access/download;Figure;Frazer24GIGAscience_Figure2.eps

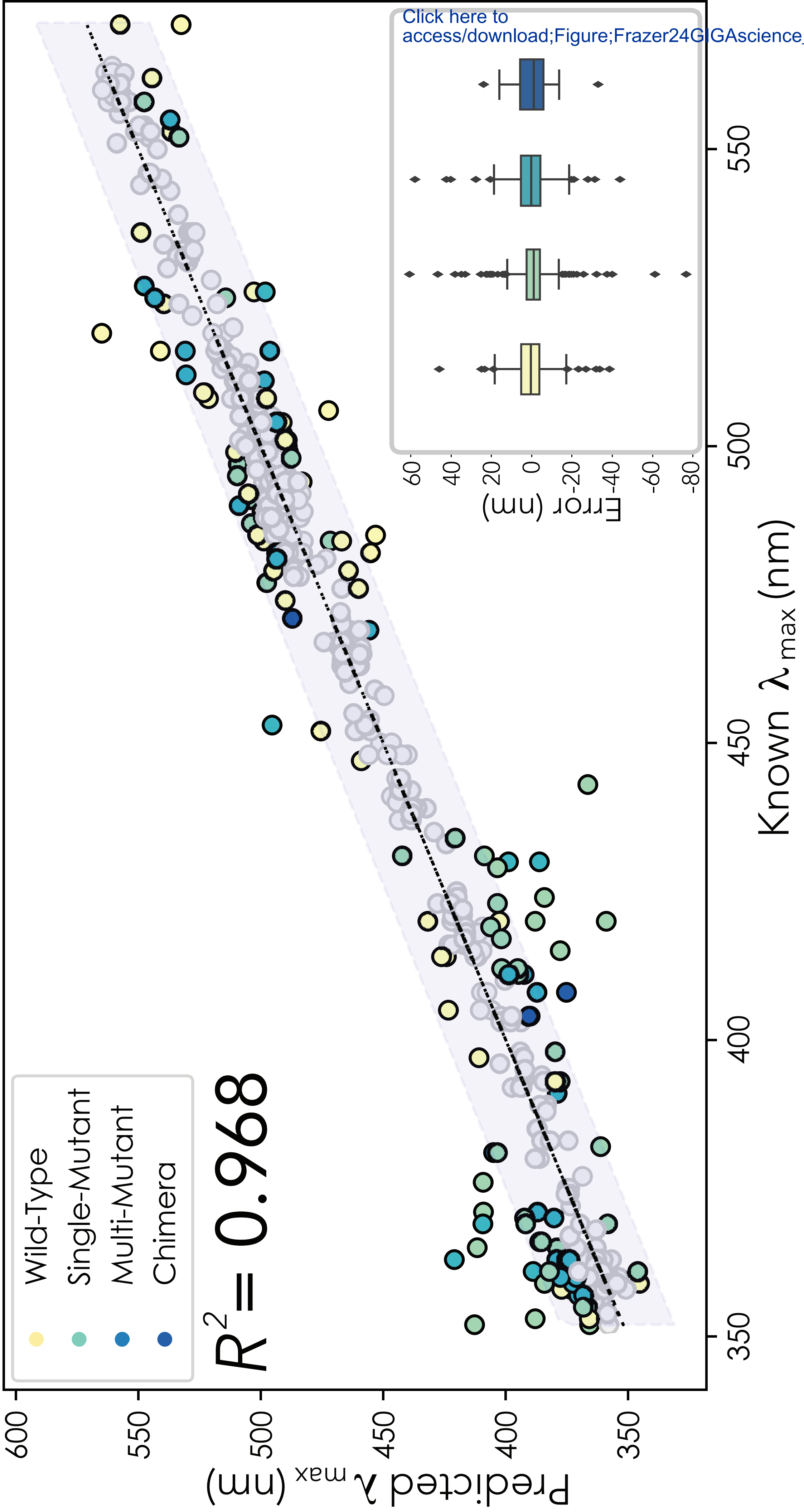

Figure 3

Click here to access/download;Figure;Figure 3 - final_wt_mut_test_95ci.png

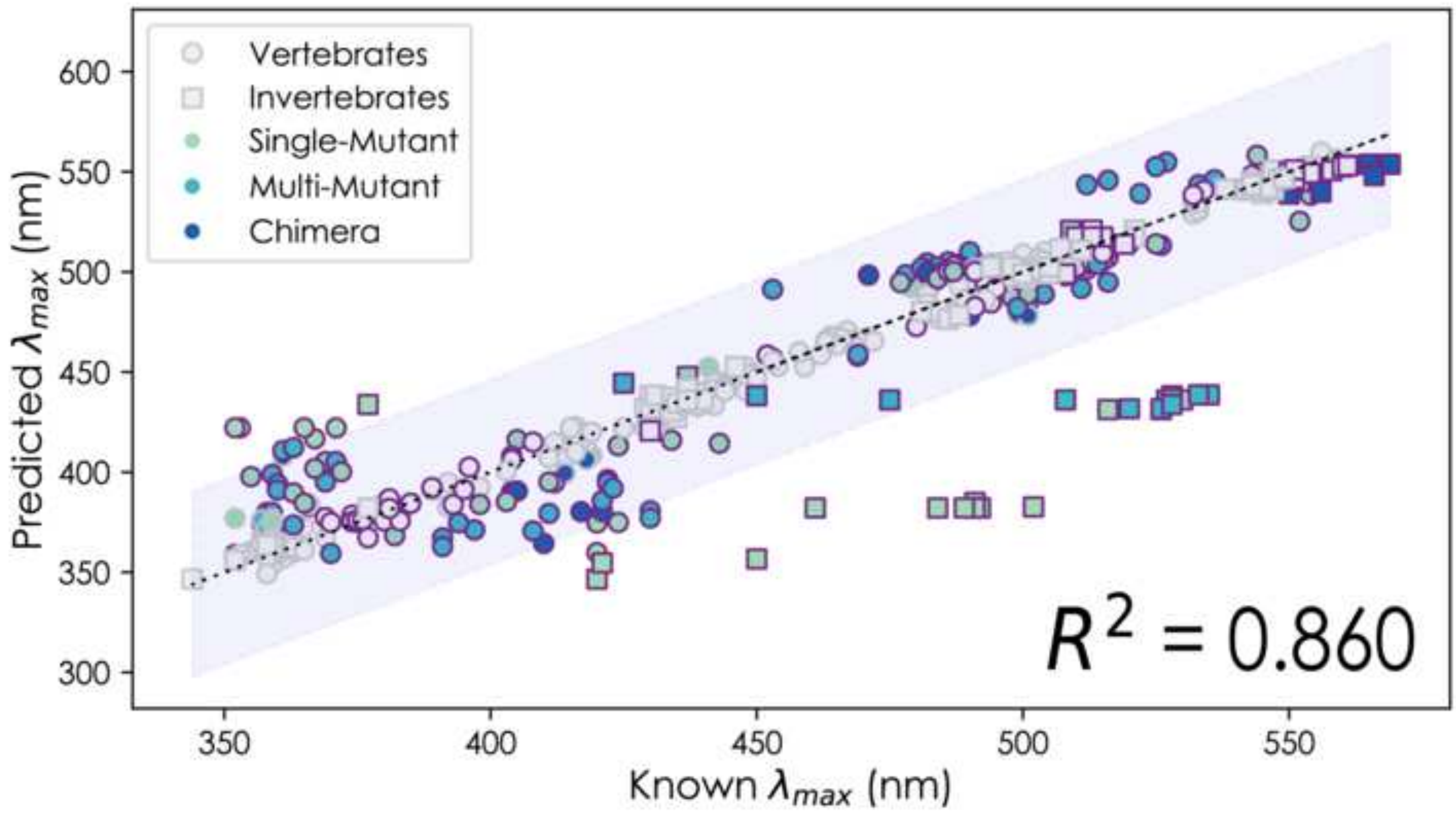

Figure 4

Click here to access/download;Figure;Figure 4 - wt_sig_pos_fig_7_13_24.png

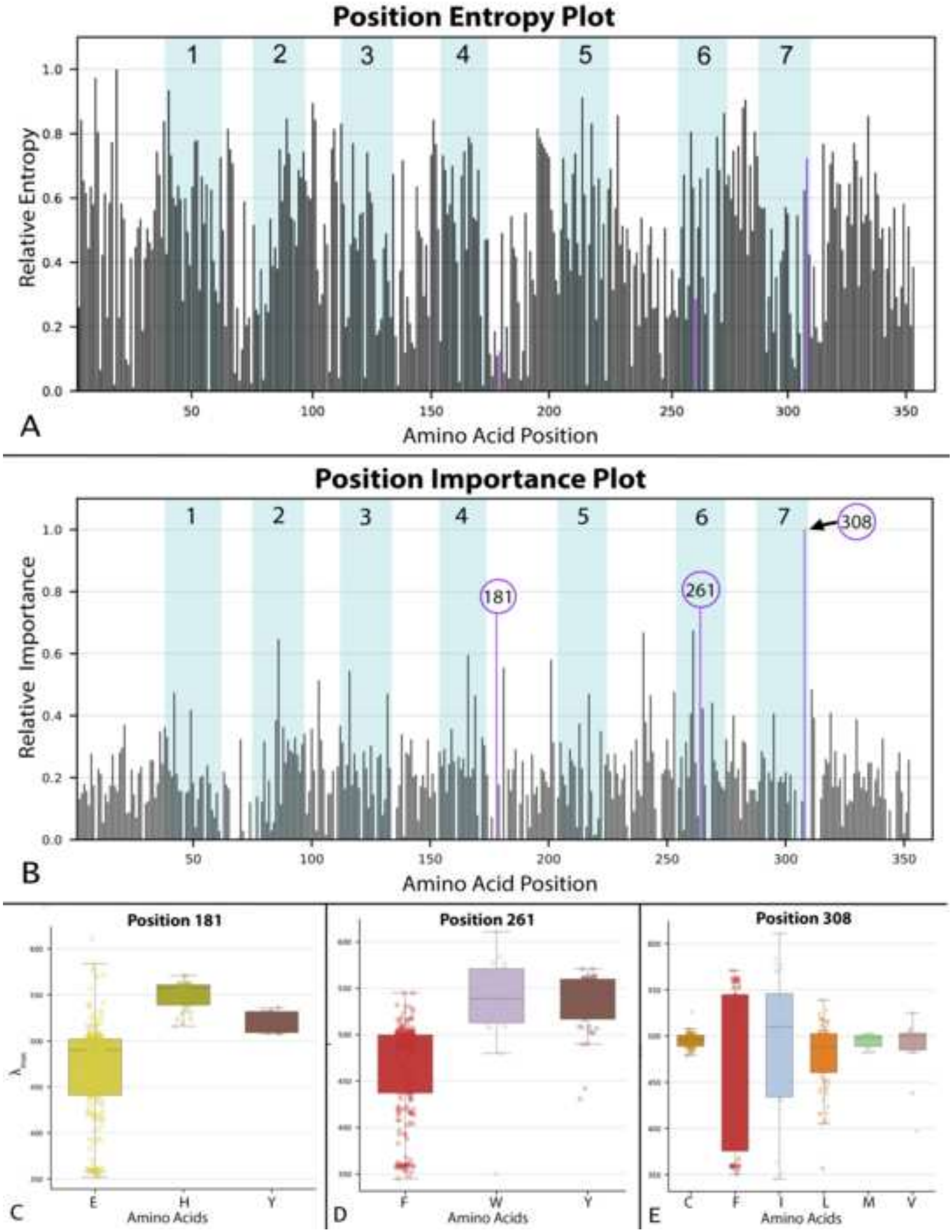

Click here to access/download
Supplementary Material
Supplementary Material
_GIGAScience24_REVISION2.pdf
