# Supplementary material for: Discovering genotype–phenotype relationships with machine learning and the Visual Physiology Opsin Database (VPOD)
Source: Gigascience. 2024 Oct 26;13:giae073. doi: 10.1093/gigascience/giae073 (PMC11512451; doi:10.1093/gigascience/giae073)
Supplement: giae073_Supplemental_File [file giae073_supplemental_file.pdf]

Supplementary Material

Supplementary Material 1 (S1):
Performance Metrics Across Opsin Subsets and Top Performing Models for *VPOD_1.1*.

Name	Data Subset Version	# Seqs	Top ML Algorithm	[b]R^2	[a]MAE [nm]	MAPE [%]	[b]MSE	RMSE
Whole Dataset	*VPOD_wds_het_1.1*	1123	LGBM	0.962	6.57	1.51	157	12.3
All Wild Types	*VPOD_wt_het_1.1*	362	XGB	0.926	8.97	1.90	241	14.5
All Mutants	*VPOD_mut_het_1.1*	761	LGBM	0.967	6.89	1.62	151	11.9
Vertebrates	*VPOD_vert_het_1.1*	968	LGBM	0.978	6.09	1.39	94.0	9.27
Invertebrates	*VPOD_inv_het_1.1*	155	GBR	0.875	14.6	3.18	625	21.8
Rods	*VPOD_rod_het_1.1*	396	GBR	0.834	3.66	0.75	31.1	5.28
MWS/LWS	*VPOD_mls_het_1.1*	162	BR	0.772	3.57	0.67	54.3	6.40
UVS/SWS	*VPOD_uss_het_1.1*	391	RF	0.790	8.84	2.27	215	14.2
T1 Opsins	*Karyasuyama_T1_ops*	884	RF	0.804	9.41	1.76	186	13.5

Supplementary Material 2 (S2):

Tracking Model Performance vs. Number of Sequences in Training Data.

Data Subset	# of Sequences	**R**²- T1	**R**²- T2	**R**²- T3	**R**²- AVG	RD - **R**²
WDS	864	0.947	0.947	0.947	0.947	0.96
	814	0.941	0.943	0.946	0.943	RD - AIC
	764	0.937	0.945	0.943	0.942	-162.00
	714	0.935	0.946	0.940	0.940	
	664	0.929	0.944	0.938	0.937	
	614	0.928	0.938	0.945	0.937	
	564	0.919	0.936	0.936	0.930	
	514	0.912	0.922	0.944	0.926	
	464	0.916	0.922	0.933	0.924	
	414	0.920	0.918	0.932	0.924	
	364	0.924	0.903	0.923	0.917	
	314	0.921	0.911	0.911	0.914	
	264	0.919	0.888	0.911	0.906	
	214	0.921	0.893	0.900	0.905	
	164	0.912	0.895	0.833	0.880	
	114	0.879	0.895	0.862	0.879	
	64	0.765	0.799	0.713	0.759	
Data Subset	# of Sequences	**R**²- T1	**R**²- T2	**R**²- T3	**R**²- AVG	RD - **R**²
Vertebrate	721	0.968	0.968	0.968	0.968	0.99
	671	0.963	0.966	0.965	0.965	RD - AIC
	621	0.960	0.969	0.965	0.965	-160.00
	571	0.955	0.968	0.961	0.961	
	521	0.949	0.966	0.965	0.960	
	471	0.951	0.964	0.965	0.960	
	421	0.952	0.961	0.966	0.960	
	371	0.951	0.956	0.962	0.956	
	321	0.944	0.961	0.962	0.956	
	271	0.942	0.952	0.951	0.948	
	221	0.938	0.928	0.949	0.939	
	171	0.928	0.907	0.938	0.924	
	121	0.896	0.910	0.911	0.906	
	71	0.925	0.816	0.868	0.870	

Data Subset	# of Sequences	R^2- T1	R^2- T2	R^2- T3	R^2- AVG	RD - R^2
Rod	352	0.834	0.834	0.834	0.834	0.93
	337	0.825517	0.808289	0.802864	0.812223	AIC - R2
	322	0.81498	0.835739	0.83895	0.82989	-122.00
	307	0.864023	0.831771	0.850227	0.848674	
	292	0.83162	0.853479	0.804649	0.829916	
	277	0.832486	0.811542	0.827711	0.823913	
	262	0.852768	0.821254	0.839961	0.837994	
	247	0.798275	0.82422	0.818958	0.813817	
	232	0.838121	0.773139	0.789483	0.800248	
	217	0.799491	0.773947	0.768506	0.780648	
	202	0.840374	0.749616	0.759987	0.783326	
	187	0.812757	0.782659	0.800555	0.798657	
	172	0.772936	0.746982	0.777117	0.765678	
	157	0.740628	0.725929	0.726127	0.730895	
	142	0.727746	0.807972	0.668367	0.734695	
	127	0.692876	0.70442	0.765842	0.721046	
	112	0.703357	0.641533	0.74384	0.696243	
	97	0.628481	0.663658	0.763486	0.685208	
	82	0.453915	0.414098	0.603912	0.490642	
	67	0.105291	0.047832	0.556672	0.236599	
	52	-0.09295	0.041141	0.340028	0.096072	

Data Subset	# of Sequences	R^2- T1	R^2- T2	R^2- T3	R^2- AVG	RD_R2
WT	318	0.902	0.902	0.902	0.902	0.84
	303	0.873	0.911	0.899	0.892	RD_AIC
	288	0.891	0.876	0.873	0.884	-89.00
	273	0.890	0.866	0.868	0.878	
	258	0.881	0.859	0.879	0.870	
	243	0.854	0.884	0.825	0.869	
	228	0.851	0.875	0.873	0.863	
	213	0.826	0.775	0.863	0.800	
	198	0.870	0.856	0.856	0.863	
	183	0.866	0.840	0.849	0.853	
	168	0.856	0.830	0.776	0.843	
	153	0.751	0.826	0.832	0.788	
	138	0.744	0.785	0.806	0.765	
	123	0.747	0.855	0.808	0.801	
	108	0.712	0.864	0.738	0.788	

93	0.749	0.886	0.574	0.817
78	0.660	0.731	0.747	0.695
63	0.731	0.715	0.460	0.723
48	0.343	0.654	0.677	0.499
33	-0.277			-0.277

T = Test , RD = Reciprocal Decay, AIC = Akaike Information Criterion

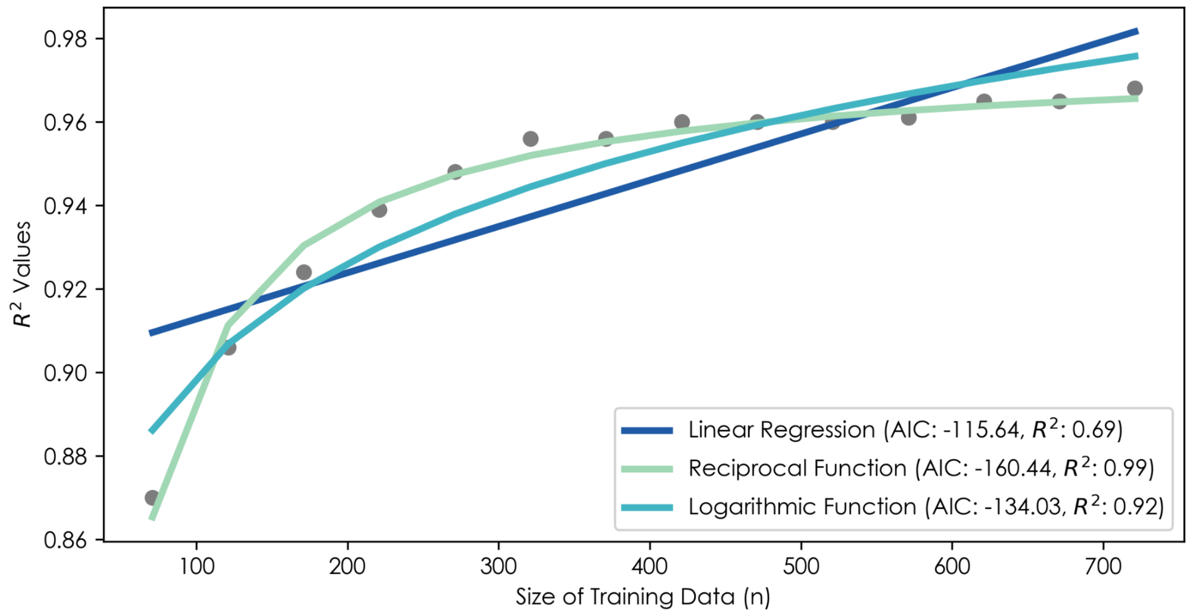

Supplementary Material 3 (S3):
Three functions fitted to visualize the relationship between training data size (number of genotypes and corresponding phenotypes) vs. model performance (**R^2**) based on results from the Vertebrate subset of data. The Akaike Information Criterion (AIC) is a measure used for model selection when comparing different statistical models, accounting for both the goodness of fit of the model and the simplicity of the model (the number of parameters used). The goal is to find a balance between a model's ability to explain the data and its complexity, preventing overfitting.

Supplementary Material 4 (S4):
Comparing ML Predictions on Invertebrate and Vertebrate UVS/SWS Opsin MSP Data.

Data Split	MSP **R^2**	MAE [nm]	MAPE [%]	SWS Test **R^2**	MAE [nm]	MAPE [%]
Whole Dataset	0.704	30.6	6.8	0.833	10.2	2.45
Vertebrates	0.044	70.8	14.6	0.914	7.89	1.9
Invertebrates	0.837	26.3	6.95	-	-	-
Wild Types Only	0.887	17.5	4.06	0.773	9.86	2.46
UVS/SWS	-	-	-	0.788	11.6	2.92

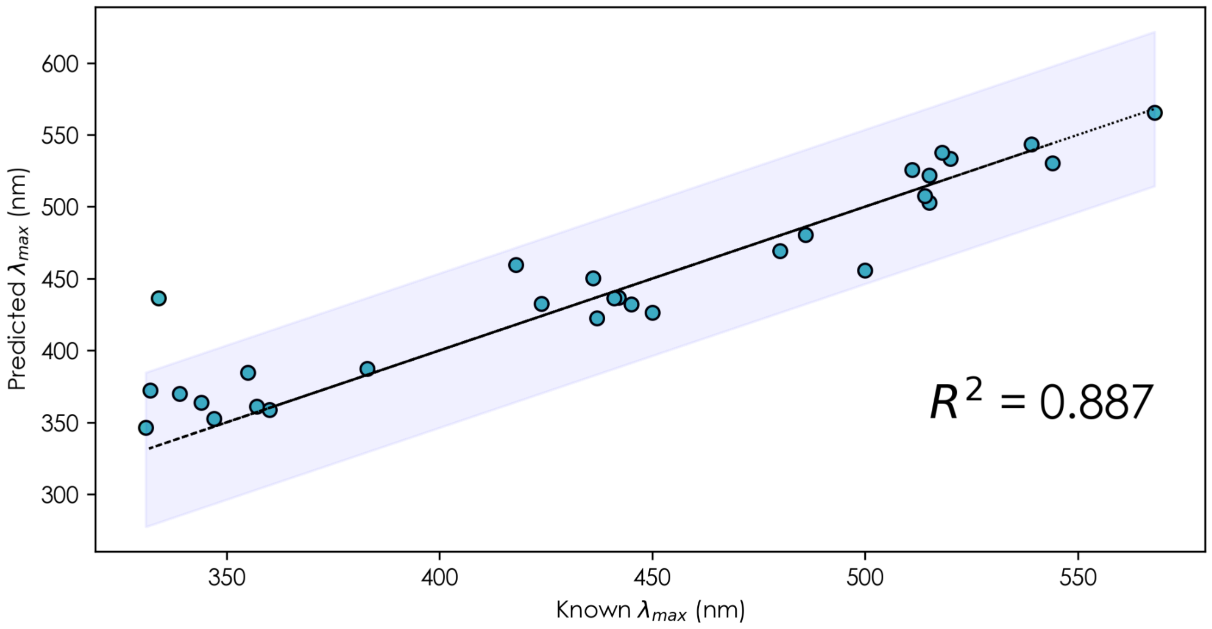

Supplementary Material 5 (S5):
Graph of WT model predictions for 30 unseen invertebrate opsins, **R^2**= 0.887, MAE =17.5nm, MAPE = 4.05. All the ‘known’ λmax values are from physiological measures, including MSP or ERG measurements (instead of purified heterologously expressed opsins), and are linked to a particular opsin sequence by in-situ hybridization. The light-gray bar surrounding the trend-line represents a 95% confidence interval.

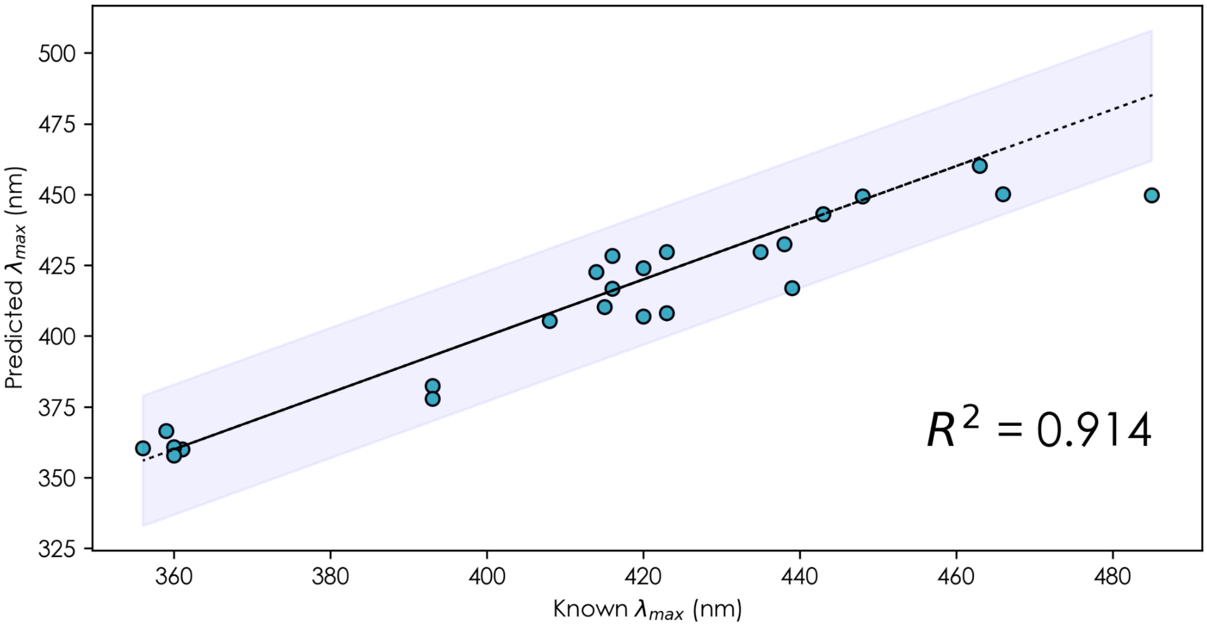

Supplementary Material 6 (S6):
Graph of Vertebrate model predictions for unseen WT-UVS/SWS data, n = 25, **R^2**= 0.914, MAE =7.89 nm, MAPE = 1.90. All sequences were randomly selected from the UVS/SWS model under the condition that they were WT opsins. The light-gray bar surrounding the trend-line represents a 95% confidence interval.

Supplementary Material 7 (S7):
Comparing Performances of ML Predictions and Phylogenetic Imputation on a Subsample of Opsin Data.

Subset	MAFFT Sample **R^2**	MUSCLE deepBreaks **R^2**	MUSCLE Sample **R^2**	Gblock deepBreaks **R^2**	Gblock Sample **R^2**	Imputation Sample Adj. **R^2**	Imputation. MAE (nm)	Imputation MAPE (%)	Sample Size (n)
Wild-Type	0.868	0.893	0.918	0.900	0.863	0.836	8.78	1.85	50
Vertebrates	0.968	0.967	0.972	0.967	0.967	0.949	6.76	1.48	50
WDS	0.948	0.946	0.96	0.942	0.964	0.958	7.6	1.65	50
Rods	0.848	0.843	0.824	0.843	0.865	0.713	4.36	0.877	50
Invertebrates	0.706	0.814	0.711	0.797	0.678	0.758	18.3	3.43	15
UVS/SWS	0.87	0.818	0.919	0.820	0.921	0.922	10.9	2.78	25
MWS/LWS	0.499	0.657	0.497	0.645	0.512	0.784	5.91	1.12	15

Supplementary Material 8 (S8) :
Results for epistasis test on the WDS, Vertebrate, WT, and Rod models.

Mutant	Known λmax (nm)	WDS λmax Prediction (nm)	Vertebrate λmax Prediction (nm)	WT λmax Prediction (nm)
NM_001014890 [WT]	500	-	-	-
NM_001014890_D83N_A292S	485	485.2	483.95	499.91
NM_001014890.2_F261Y_A269T	520	520.0	515.39	515.3875
NM_001014890.2_A164S_A269T	514	515.5	513.8	510.2689344

Supplementary Material 9 (S9):
Functionally characterized spectral tuning sites predicted by the WT models.

Data Subset	Position on Bovine	Importance Value (0.0-1.0)	AA Residue on Bovine	TMD	Verified Tuning Site
All WT Opsins	308	1	M	7	Yes
	261	0.675	F	6	Yes
	86	0.648	M	2	Yes
	201	0.581	E	CT/EC	Yes
	181	0.555	E	CT/EC	Yes
	116	0.545	F	3	Yes
	253	0.476	M	6	No
	42	0.476	A	1	No
	217	0.471	I	5	Yes
	169	0.466	A	4	Yes
	243				No
	49	0.419	M	1	Yes
	295	0.407	A	7	Yes
	269	0.442	A	6	Yes
Rod WT Opsins	292	1	A	7	Yes
	122	0.417	E	3	Yes
	205	0.256	I	5	Yes
SWS WT Opsins	113	0.302	T	3	Yes
	81	0.156	M	2	Yes
	114	0.124	L	3	Yes

Supplementary Material 10 (S10):
Phylogenetic gene-tree of all wild-type opsins (n=362), including ancestral constructs (branch lengths = 0), constructed from VPOD_wt_het_1.1 dataset.
In these tree we've annotated the major opsin groups (c-opsins, r-opsins and some tetraopsins), then further annotated the c-opsin families (LWS, SWS1, SWS2, Rh1, and Rh2). We've also assigned taxonomic annotations by class, which are color-coded and provided by the key.

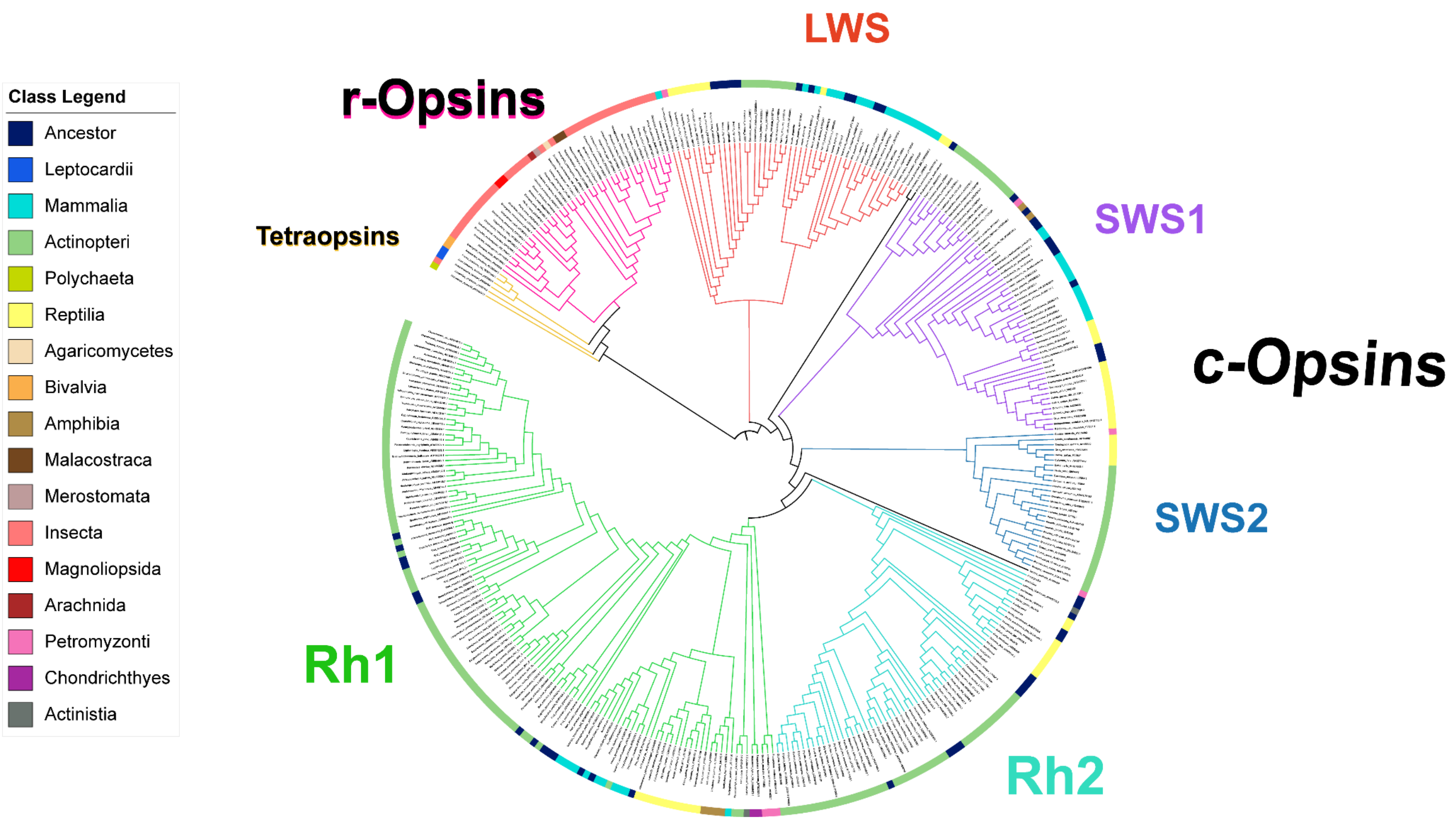

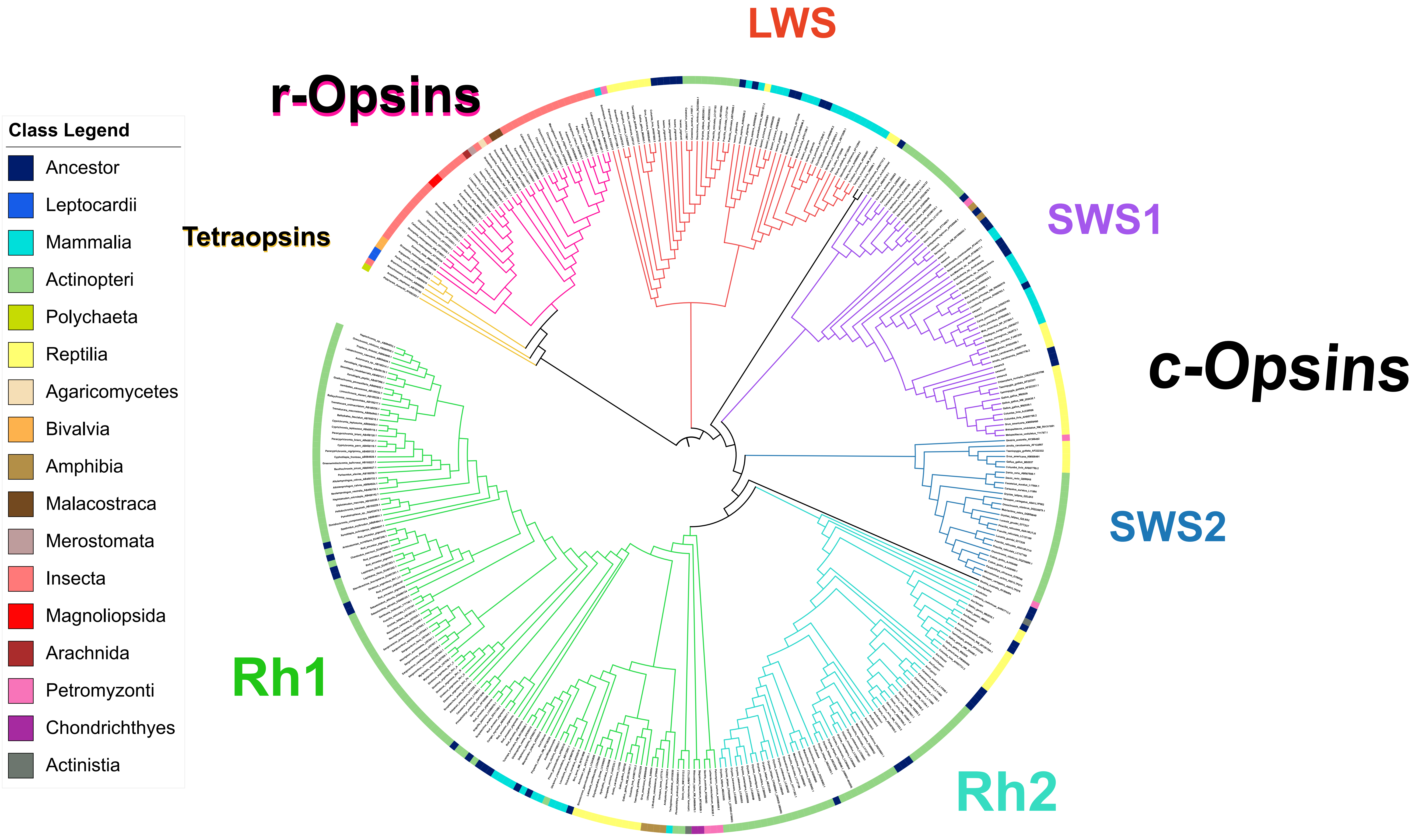

LWS
r-Opsins
Tetraopsins
SWS1
c-Opsins
SWS2
Rh1
Rh2
Class Legend
Ancestor
Leptocardii
Mammalia
Actinopteri
Polychaeta
Reptilia
Agaricomycetes
Bivalvia
Amphibia
Malacostraca
Merostomata
Insecta
Magnoliopsida
Arachnida
Petromyzonti
Chondrichthyes
Actinistia

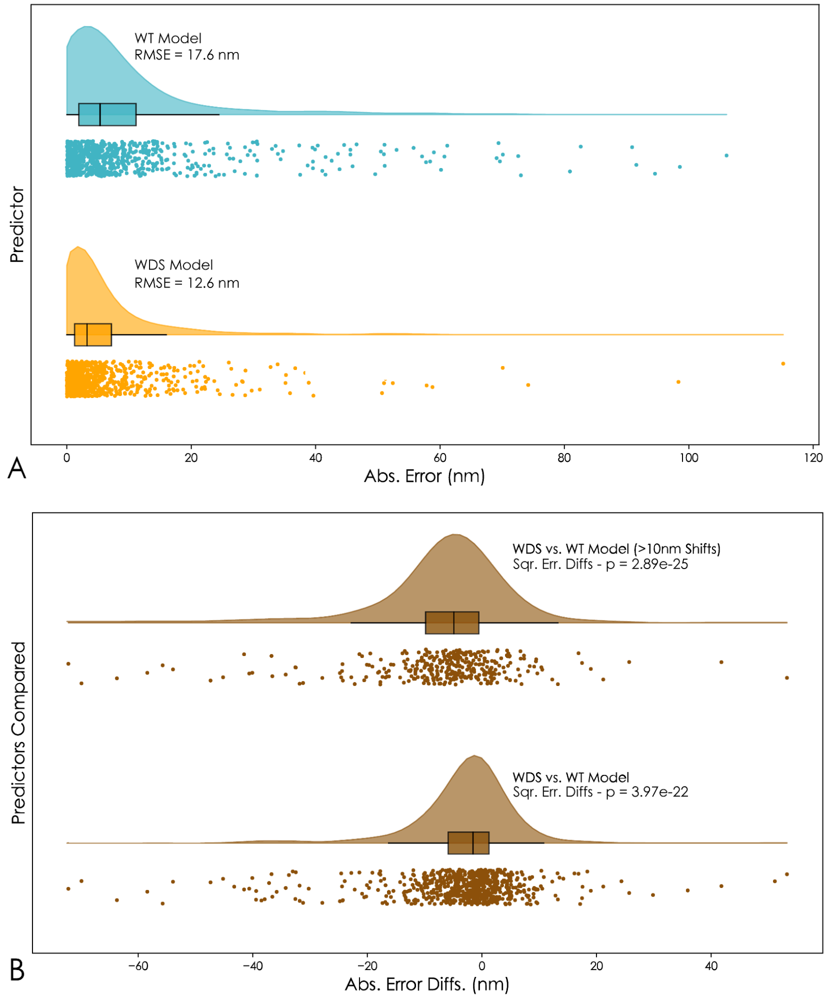

Supplementary Material S11 (S11):

Including data from experimentally mutated opsin sequences reduces errors in predicting λ_{max}

(**A**) Distributions of errors from predicting λ_{max} of experimentally mutated opsin sequences. Blue are prediction errors when using the WT model, which lacks experimentally mutated sequences (Root Mean Square Error (RMSE) = 17.6 nm). Orange are prediction errors when using the WDS model, which includes experimentally mutated sequences (RMSE = 12.6nm). (**B**) Data from experimental mutants significantly improves predictions of λ_{max} when using a model trained with experimental mutants (WDS) compared to a model without (WT) experimental mutant data; rejecting the null hypotheses of no difference between prediction errors based on different models. At top is the distribution of differences between predictions with and without experimental mutants in the training data for large effect mutations (> 10 nm). At bottom is the same for all mutations. We plot differences of absolute error instead of squared error in B for easier visualization, although p-values were calculated using distributions of squared errors. Additionally, plotting raw differences allows seeing most values are below zero, meaning predictions with WDS (which has experimental mutants) have less error than those without experimental data from mutants (WT).

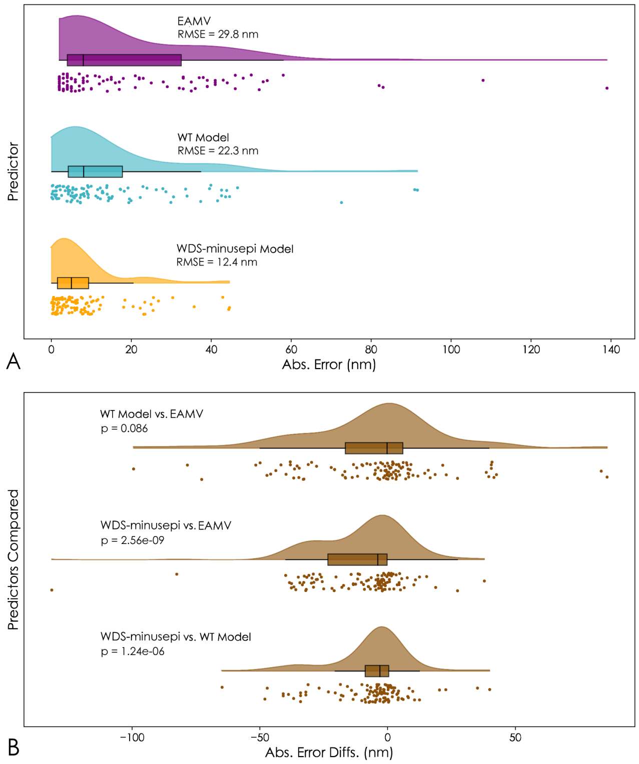

Supplementary Material 12 (S12): Including data from experimentally mutated opsin sequences reduces errors in predicting epistatic effects (**A**) We analyzed opsins with multiple mutations whose known effect on λ_{max} phenotype were non-additive (epistasis). In Purple, we plot the difference (absolute error in nm) between known λ_{max} phenotypes with epistasis, compared to λ_{max} phenotypes ignoring epistasis by assuming individual mutations are not additive, which we call Epistasis-free Additive Mutation Values (EAMV). Here Root Mean Square Error (RMSE) = 29.8 nm. In Blue, we plot errors when predicting epistatic phenotypes using a model trained without opsins containing experimentally generated mutations (WT), which lead to RMSE = 12.4 nm. In Orange, we plot errors when predicting epistatic phenotypes using a model trained with opsins containing experimentally generated mutations but excluding those whose mutational effects are non-additive (WDS-minusepi), which lead to RMSE = 12.4. (**B**) Our tests of the null hypotheses of no underlying differences between the distribution of squared error for predictions of λ_{max} for he 111 'epistatic opsins' were rejected with Wilcoxon Sign-Rank Tests after Bonferroni correction by the WDS-minusepi model versus WT model (p = 1.24e-06); WDS-minusepi model versus EAV (p= 2.56e-09), but not rejected for the WT model versus EAV (p = 0.086). The large differences in RMSE and the results of the statistical comparisons strongly support the idea that the inclusion of even single mutants greatly reduces the error of ML models when predicting epistatic interactions between mutations and that this error is significantly less than the error we would observe if our models simply treated mutations as additive. Conversely, the insignificant difference between WT predictions and EAMV indicate there is not enough information about epistatic interactions in wild type (which excludes artificially mutated opsins) data alone to accurately predict intragenic epistasis. As with S11, We plot differences of absolute error instead of squared error in B for easier visualization, but use squared error for statistical comparison.

Supplementary Material 13 (S13):
Ranked ML Algorithm Performances.

Model Algorithm	Top Model Count	Total Points	Overall Ranking
gbc	3	120	1
BayesianRidge	2	104	2
lgbm	4	100	3/4
rf	1	100	3/4
XGB	1	87	4
ET	0	68	5
Adaboost	1	51	6
DT	0	50	7
LassoLars	0	48	8
Lasso	0	41	9
HubR	0	27	10
LR	0	0	12
