# Supplementary material for: Discovering genotype–phenotype relationships with machine learning and the Visual Physiology Opsin Database (VPOD)
Source: Gigascience. 2024 Oct 26;13:giae073. doi: 10.1093/gigascience/giae073 (PMC11512451; doi:10.1093/gigascience/giae073)
Supplement: giae073_Response_to_Reviewer_Comments_Original_Submission [file giae073_response_to_reviewer_comments_original_submission.pdf]

Author's Response To Reviewer Comments

We thank the reviewers and editor for their time and comments on our manuscript. Herein we respond to the comments inline, address concerns, and note specific changes to the manuscript. Our responses are in blue.

Sincerely,

Seth A. Frazer et al.

Editor’s Comments:

Based on these reports, and my own assessment as Editor, I am pleased to inform you that it is potentially acceptable for publication in GigaScience, once you have carried out some essential revisions suggested by our reviewers.
Reviewer #2 in particular has some concerns regarding the analysis, description and presentation of the data which need to be addressed and clarified.

-

Reviewer reports
Reviewer #1:

Frazer and colleagues set out to assess the ability of machine learning (ML) approaches to predict spectral sensitivity (lmax) of animal opsins from their amino acid sequence. To this end they first develop a database of phenotyped opsins (opsin sequences with known lmax), which they term Vpod1. They then explore how various factors of the ML process impact its ability to predict lmax. These include the nature of the input training dataset (size, phylogenetic and gene family diversity, inclusion of data from mutagenesis experiments) and the ML method. For comparison they include a phylogenetic imputation approach that predicts lmax based upon overall sequence similarity. They test the validity of their approach according to their ML pipelines' ability to predict: lmax for the training dataset; the outcome of mutagenesis; lmax for a test dataset extracted from the training dataset; known epistatic interactions; and established spectral tuning sites. In all cases, they report various degrees of success and conclude that the ML approach can be used to predict lmax (almost as well as phylogenetic imputation but with reduced computational cost) provided that the training dataset is sufficiently rich (it performs poorly for invertebrate opsins for which data are limited) and, ideally, benefits from mutagenesis datasets.
I am no expert in machine learning and will leave others to comment on that aspect of methodology but in general this study represents an interesting addition to the literature. The idea of predicting lmax from amino acid sequence is not new e.g. as the authors acknowledge the '5 sites rule' for cone pigments is long established. Applying ML holds the promise of a more efficient process for achieving similar predictability for other branches of the animal opsin family. In that regard, the inherent limitation in the ML approach is highlighted - it is particularly valuable in branches of the family for which information is sparse (invertebrate opsins), but performs poorly in those branches without more starting information about structure:function relationships (which itself replaces the need for ML to some extent). Nonetheless, it certainly has the potential to be a valuable tool and this paper represents a sound exploration of its characteristics and one important feature of the paper is that it confirms that ML can allow fairly good predictions based solely on data from wildtype opsin sequences.

I have relatively few suggestions for improvement. The most important is that the authors appear to have omitted one technology for the process of defining lmax (introduction, method and discussion). We and others have used heterologous action spectroscopy to describe lmax for a growing number of animal opsins. In this technique spectral sensitivity is defined using live cell assays of light response for opsins

expressed in immortalized cell lines. Those data could be included in the Vpod1 dataset. It would also be appropriate to mention the approach as a tool for populating the training dataset as it has the advantage of being applicable to opsins that don't reliably form pigments in vitro (e.g. many invertebrate opsins) and does not rely on access to the animal itself but only to its genome sequence.

***We thank the reviewer for their suggestion to highlight heterologous action spectroscopy and we have added this in the introduction paragraph along with ERG, MSP, and heterologous expression; followed by purification and absorbance measures. To be clear, we did already include data from heterologous action spectroscopy in VPOD_1.0 but we did not make the proper effort to clarify/distinguish this technique from what we referred to in the first version of the manuscript with the blanket terminology ‘heterologous expression data’. We now clarified action spectroscopy as a separate technique.

The authors also may wish to relate Vpod1 to another recently published database of animal spectral sensitivities albeit collected for a different purpose (https://doi.org/10.1016/j.baae.2023.09.002).

***We thank the reviewer for their suggestion and have now cited this compendium in the introduction. We were aware of these and other compendia of λmax data, but those are not connected to any genetic data (opsin sequences). We have now added mention of these previously published databases.

Some minor points. The authors note with surprise that ML performed poorly for the rod opsin dataset. Could this be because their metric (R2) is sensitive to the degree of variability in lmax in the training dataset, which is constrained in rod opsins?

***We acknowledge that the degree of variability in λmax is constrained for rod type opsins, and that because variance in the response variable is low, even small changes in the model's predictions could lead to larger changes in R^2. In situations with limited variability in the response variable (in our case λmax), it might also be helpful to consider alternative metrics we provided that are less sensitive to variance, such as mean absolute error (MAE) and root mean squared error (RMSE). These metrics focus on the absolute magnitude of the errors rather than the proportion of explained variance, making them less susceptible to the issues of constrained/limited variance. In line with the reviewer’s suggestion, and our response here about MAE and RMSE, we’ve added very similar text in the main text within the results section.

I found the pastel colors in Fig2 and 3 hard to discern, more separation on the color pallet would be appreciated.

***We thank the reviewer for their suggestion, however, we decided to keep the original colors because the colors were selected for color-blind friendliness.

Reviewer #2:

The authors compiled a collection of opsin/rhodopsin proteins and their associated light absorption properties from literature; using the measured wavelength of maximum absorbance as a proxy. In addition, they include multiple sequence alignments (MSA) of the proteins including subsets of vertebrate and invertebrate sequences. The data is provided as tab-seperated, comma-seperated, and FASTA files. This is a valuable resource for studying opsins and vision related phenotypes. The authors then use gradient-boosting, random forests, and bayesian ridge regression to predict the wavelength of maximum absorbance from the protein sequence MSAs. Furthermore, they investigate whether their models can be used to identify amino acid changes that impact the wavelength of maximum absorbance and epistasis. This is based on a small set of opsin mutants that have been reported in literature. The manuscript is well structured and written. I have some concerns regarding the

analysis, description and presentation of the data.

1. A traditional cross-validation by random sampling can be inadequate for phylogenetically related sequences. If closely related species are part of the data training and test sets may contain nearly identical sequences. Excluding entire lineages instead of random sequences during training would circumvent this issue.

***We agree with the reviewer that phylogenetic relatedness will inflate R2 values when using random sampling in cross-validation. However, we disagree that excluding entire lineages would circumvent this issue. This is because of the always-hierarchical nature of phylogenetics: even if excluding one lineage, the remaining sequences will still have a phylogenetic structure. Furthermore, there is no way to define at what depth such a 'lineage' should be defined. Instead of excluding entire lineages of an arbitrarily chosen depth that would still have phylogenetic signal, we have further emphasized in the writing that phylogenetic non-independence will inflate R2 values. In addition, we believe some of the tests and discussions of the original version were consistent with this truth. Namely, opsin sequences that are distantly related from those in the data used for training generally will be more difficult to predict. This is the reason phylogenetic non-independence inflates R2 because sampling sequences closely related to others in the database will be very easily predicted, and will lead to higher R2 than sequences chosen completely at random from all animal opsins. This is inherent in our discussion of the lower predictability of invertebrate opsins: Because we have fewer invertebrate (rhabdomeric) opsins, we have lower R2 when targeting those sequences. In addition, our test using MSP data is independent of the phylogenetic relatedness of the data used in model training, and, therefore, provides a less inflated estimate of the ability to predict λmax. In summary, we clarified in the text that non-independence can inflate R2 and we believe the implications of this are clear -- that opsins distantly related from those in the database will be more difficult to predict.

2. Based on Fig. 3, Fig. 2, and p. 6, the models do not generalize well given that they only predict mutants well which exhibit a similar wavelength of maximum absorbance as the wild type. Based on the plots (Fig. 2 and Fig. 3) it does not look to me like the model trained on mutants+WT performs substantially better than the WT model for mutants with large wavelength shifts. This would be in contrast to p.16 "Particularly illustrative of these ideas are our analyses with and without experimentally mutated opsins". The authors should either show statistics regarding the improved performance for mutants with large shifts or change the corresponding parts.

***We acknowledge that there is not a particularly easy visual distinction between the predictive power of our Whole Dataset Model and Wild-type model, when using mutagenesis data (especially those mutants which cause large shifts in λmax). As suggested by the reviewer, we have now added a statistical analysis using the Wilcoxon Signed-Rank Test (WSRT) on ALL mutagenesis data, which indicates that there is a statistically significant difference between the two model's error distributions, thereby supporting the alternative hypothesis that the distribution underlying the difference between the squared error of the WDS and WT models (WDS squared error - WT squared error) is stochastically less than a distribution symmetric about zero (Wilcoxon Test Statistic = 87309.0, p-value = 9.96e-22, WDS Root Mean Square Error (RMSE) = 12.6nm, WT RMSE = 17.6nm). Furthermore, statistical analysis using WSRT on only mutant data where mutations cause large shifts in λmax (greater than 10nm) from the wild-type indicates that there is also a statistically significant difference between the two model's squared error distributions and once again supports the alternative hypothesis that the distribution underlying the difference between the error of the WDS and WT models is stochastically less than a distribution symmetric about zero (Wilcoxon Test Statistic = 10802.2, p-value = 2.29e-25, WDS Root Mean Square Error (RMSE) = 17.0nm, WT RMSE = 24.2nm). We have included a Bonferroni correction for these multiple tests which remain statistically significant. The statistical support provided by these tests and the lower RMSE values for the WDS model for predicting mutations with large shifts from the WT opsins supports the improved predictive power of our WDS model over the WT model.

***In line with this response, we have also added something similar in the main text discussion section.

3. The data set description should be more detailed in parts. It should be shown how the opsins/rhodopsins

classes (UVS, SWS, MWS, Rhodopsins, LWS) are distributed across the vertebrate and invertebrate phylogeny, for example by a phylogenetic tree and their number per species. Are the mutated opsins/rhodopsins derived from a small set of species or do they reflect most of the vertebrate phylogeny?

***We thank the reviewer for their suggestion, and we have now constructed a phylogenetic tree from the wild-type opsin data, including ancestral constructs (branch lengths = 0), as shown in Figure S10. In this tree we've annotated the major opsin groups (c-opsins, r-opsins and some tetraopsins) and then further annotated the c-opsin families (LWS, SWS1, SWS2, Rh1, and Rh2) and assigned taxonomic annotations by class instead of by species because we believe it is more informative. Additionally, in the manuscript, we have already noted that the mutant opsin data are derived from a smaller subset of species inherently because people continue to work in established systems. Nevertheless, we have clarified that further in the data description and discussion.

4. How importance scores are estimated for the different models should be explained.

***We believe that because the calculations of feature importance scores occur as part of the deepBreaks pipeline, directly explaining how importance scores are calculated is beyond the scope of this paper as it is clearly detailed under the 'Interpretation' heading of the methods section in the deepBreaks publication. Nevertheless, in line with your suggestion, we've added the following sentence to the methods section of our paper, directly referring to the deepBreaks publication for those who want a more detailed explanation of the feature importance score processes.:
"For a more detailed explanation on how position importance scores are calculated for different models, refer to the 'Interpretation' heading under the methods section of the deepBreaks publication [59]"

5. The "ML often predicts the effects of epistatic mutations" section needs some clarifications. Why were only three sequences investigated? Do none of the other double mutants show epistasis when compared with the corresponding single mutations? In this paragraph, it is not always clear whether wavelengths and additive wavelengths are obtained from predictions or actual measurements.

***We thank the reviewer for pointing out the lack of support for this claim and we decided to run additional experiments to explore more generally the capabilities of our ML models to predict epistatic interactions between mutations. For this, we selected all multi-mutants where we possess the data for each individual component mutation, then further filtered by mutants with non-additive (epistatic) interactions between mutations (which we define as >1nm difference than the additive effects of the individual mutations). The final selection of 111 'epistatic mutants' was then removed from the WDS training data all-at-once, and used as test data following training ($R2 = 0.969$, RMSE = 12.4nm) in a dataset we term WDS-epi. 105/111 of the mutants defined as epistatic were predicted as epistatic (non-additive) by the WDS-epi model (>=1nm difference than the expected additive λmax values). We then compared those predictions to predictions made by the WT model which contains no mutant data ($R2 = 0.894$, RMSE = 22.3nm) and to the λmax values we'd expect for each mutant if the mutations all behaved in an additive fashion ($R2 = 0.878$, RMSE = 29.8nm). We used another WRST to test for significant differences in the distributions of squared error between the WDS-epi model and WT model; WDS-epi model and the expected additive mutation λmax values (EAV); and WT model and EAV. The results of these WSRTs indicate a statistically significant difference between the distributions of squared error for the WDS-epi model to WT model (p-value = 1.24e-06) and WDS-epi model to EAV (p-value = 2.56e-09) but no statistically significant difference between the distributions of squared error for the WT model to EAV (p-value = 0.086). Moreover, the RMSE of the WDS-epi model (12.4nm) is half that of the WT model (22.3nm) and the EAV (29.8nm), respectively. Together, these results show that the inclusion of even single mutants greatly reduces the error of ML models when predicting epistatic interactions between mutations and that this error is significantly less than the error we'd get if our models simply treated mutations as additive.

6. The description in git repository (https://github.com/VisualPhysiologyDB/visual-physiology-opsin-db) is very sparse. The content of the different files and how they relate to each other should at least be briefly explained in a README. It would also be helpful to add gene names, and the source of the sequence to the meta files.

***We have updated the GitHub repository as requested with more detailed information but have not added gene names to the metadata files - those can be found in the raw database file 'opsins.csv' under the 'vpod_data/VPOD_1.0/raw_database_files' folder.

Minor comments

1. For Fig. 4A, since MSAs are already computed it would be interesting to indicate the conservation of amino acids per position. Are important amino acids correlated with sequence conservation?
***We thank the reviewer for their suggestion and we have added an entropy plot by aligned amino acid positions from the wild-type opsin data, which is now Fig. 4A - and we have moved the relative importance plot to Fig. 4B. Based on the low R2 of 0.001, there is seemingly little to no correlation between amino acid sites that are important to model predictions and their relative entropy scores. This is somewhat expected since we do drop all 'zero-entropy' sites during preprocessing, since a site with no variation stands to provide no information to the effects of variation on the resulting phenotype. However, we'd predict that if there were any correlation between site conservation and model importance, it would be for sites which are moderately conserved and in close proximity to the essential chromophore binding site (site 296).

2. In Tab. 1, R2 is used to compare different models which are based on different subsets, and also pot. differently sized MSAs. An adjusted R2 might be more suitable to account for different numbers of features.

***While we appreciate the reviewer's suggestion, it's important to note that a key step in the preprocessing completed by deepBreaks is the 'gap_drop' step, in which the program drops all positions/columns with gaps in greater than some threshold proportion (typically 0.6). Thus the number of features remains very similar (within 5-10 position difference) across the datasets. As a result, we do not believe the size of the sequence alignment is a factor in differences between R2 values.

3. It would be helpful to add a Docker image to the github repository to make it easier to use

***We appreciate the reviewer's comment about adding a Docker image; we have added it and the link to the Docker image of the latest version of the deepBreaks program:
https://hub.docker.com/repository/docker/omicseye/deepbreaks-dc/general.
This Docker image includes a summary of required package libraries and instructions on how to use it. Along with our existing online materials with tools used, deepBreaks, we also have a Jupyter notebook, instructions for Conda installation, and Code Ocean capsule (https://codeocean.com/capsule/9484494/tree/v1) for deepBreaks. These resources should help practitioners using the main ML program we used, deepBreaks, described elsewhere, use the VPOD database for Opsin applications, or for other genotype:phenotype data.

Reviewer #3:

In their manuscript, Frazer et al. developed a machine-learning approach to predict the spectral sensitivity of a visual pigment based on the gene/amino acid sequence of the opsin protein. First, they created a visual opsin database based on heterologously expressed genes from the literature. They then used deepBreaks, an ML tool developed to explore genotype-phenotype associations, to run several different models and test how well ML could predict spectral sensitivity. Their main findings are that the larger the dataset for training and the more diverse (both in opsin sequences themselves and phylogenetic breadth they were derived from) the dataset, the better the predictions will become. However, there is a plateau for the number of training sequences that should be used as a minimum (~ 200), with a slight gain afterwards. As such, the suggested ML approach works well for larger datasets but needs refining for smaller datasets. There are also several drawbacks
to the approach that need to be carefully considered when interpreting the results, including the fact that ML cannot accurately predict the effect on phenotype if confronted with a new mutation or a new combination of mutations not used during training.

I found the study to be well-written and easy to follow. The results support the conclusions, and as far as I can tell, the ML and associated analysis were performed accurately. All the code and the database are

readily accessible, too. It is great to see that we are at a point now where computational power has reached a level that can be used to predict gene-phenotype relationships accurately. The use of ML to study the function of (visual) opsins, i.e., spectral sensitivity, especially if additional parameters can be included, will undoubtedly be of great help to the field and welcomed by the community. As such, I have no major concerns and only a few minor comments I recommend addressing before publication.

Minor comments

Introduction

- Please add a sentence to explain that a visual pigment consists of an opsin protein bound to a chromophore/retinal and that the two units together lead to the 'spectral sensitivity' phenotype. You cover it in the discussion, but it would be helpful for the reader to have this information upfront.

***We thank the reviewer for their suggestion and have added the following sentence to our introduction:
“Opsins are a family of G-protein Coupled Receptors (GPCR) which bind to a retinal chromophore. The two units together, opsin and chromophore, form visual pigments which absorb photons [23].”
Here is the sentence in full context:
“Opsins are a family of G-protein Coupled Receptors (GPCR) which bind to a retinal chromophore. The two units together, opsin and chromophore, form visual pigments which absorb photons [23]. Opsins have crucial roles in many organismal functions, including circadian rhythms, phototaxis, and image-forming color vision. A critical opsin phenotype is spectral sensitivity - the range of wavelengths to which a gene or organism is sensitive.”

- Please provide a reference for the following statement: '[…], and purification of heterologously expressed opsins followed by spectrophotometry [REF]'.

***We added the reference as requested.

- You say, 'Despite opsins being a well-studied system with an extensive backlog of published literature, previous authors expressed doubts that sequence data alone can provide reliable computational predictions of λmax phenotypes [37-40]'.

I agree that the spectral sensitivity predictions from sequences have been criticised in the past as they were sometimes oversimplified (including some of our work). However, spectral sensitivity predictions based on computational modelling, albeit not using ML, have previously been attempted successfully several times, e.g., by Jagdish Suresh Patel and colleagues, and should be mentioned here.

***We have added the reference to the work of Jagdish Suresh Patel as suggested in the introduction and provide the updated text below.
“Despite opsins being a well-studied system with an extensive backlog of published literature, previous authors expressed doubts that sequence data alone can provide reliable computational predictions of λmax phenotypes [41–44]. At the same time, λmax predictions showed promise on the limited scale of vertebrate cone visual pigments via atomistic molecular simulations [45,46]. “

- You say that: 'The extensive data on animal opsin genotype-phenotype associations remains disorganized, decentralized, often in non-computer readable formats in older literature, and under-analyzed computationally'.

Again, I agree that the opsin data can profit from a centralized data bank like the one you created. However, there have been several previous attempts at summarizing opsin data in recent years (although not specific for heterologously expressed opsins), for vertebrates at least. For example, work by Schweikert and colleagues on fish visual opsins and recent work on frog opsins by Schott et al. These studies should be mentioned and cited appropriately here, as tremendous work went into collating the datasets in the first place.

**As mentioned above in response to a different suggestion, we have added the citations, noting though

that these are not opsin databases, but rather databases of lambda-max values without specific connection to genetic sequences.

Results

- The use of MWS opsin is somewhat confusing. I presume this refers to vertebrate lws genes that are mid-wavelength shifted? Why have these as a separate group? Ancestrally, there are five sub-families of visual opsin genes in vertebrates: sws1 & sws2 (SWS), rh1, rh2 and lws (MWS & LWS). The MWS range in Figure 1 should be part of a larger lws derived grouping.

***We thank the reviewer for their suggestion and have modified Figure accordingly.

- This part reads like a discussion. It also needs a reference for the age of T1 opsins: 'The similar levels of performances between T1 and invertebrate models were unexpected, especially considering it has a training dataset five times larger than the invertebrate model. One possible explanation is that the very old age of T1 opsins [REF] might have led to a higher complexity of genotype-phenotype associations that are not yet well sampled enough to allow good predictions.'

***We believe that short discussion points, which are directly related to a specific result, are well-placed in the results section. Because the T1 opsins are not a strong focus of the rest of the manuscript, we believe it is difficult to incorporate these points into the discussion.

- These two sentences could also be weaved into the discussion rather than the results section: 'These equations do not account directly for taxonomic, genetic, or phenotypic diversity, as the number of genes is on the x-axis. Therefore, one should be cautious about applying them to predict model performance based on training data size alone.'

***We believe that short discussion points, which are directly related to a result, are well-placed in the results section. Therefore, we kept some mention of these points in the Results. In addition, we added a sentence to the discussion section as requested.:
“Despite the insight provided by these equations, we still encourage caution when applying them to predict model performance on training data size alone as they do not account directly for taxonomic, genetic, or phenotypic diversity. ”
Here is the sentence in context:
“Based on the non-linear fit between size of training data set, and model performance, we estimate that including about 200 sequences (and corresponding λmax) from a taxonomically and phenotypically diverse range still provides improvements to model performance. Above 200 sequences, there is still improvement, but at a diminishing rate consistent with a reciprocal model (Table S1, Figure S2). Despite the insight provided by these equations, we still encourage caution when applying them to predict model performance on training data size alone as they do not account directly for taxonomic, genetic, or phenotypic diversity. ”

- Table 1: What do MAPE and RMSE stand for, and what do those numbers mean? Maybe also include a short explanation of the acronyms and their meaning in the main body of the text.

**We have now spelled out each acronym in the methods section, explaining the results provided by deepBreaks.

- This should also be mentioned in the discussion: 'Until the models are trained with more invertebrate (r-opsin) data, we do not put high confidence in the estimates of λmax.'

***In line with our previous discussion we have added the following sentence to our discussion: ‘Given this currently limited dataset we do not put high confidence in the λmax estimates of both wild-type and mutant invertebrate (rhabdomeric) opsins.’
Below is the new entry in context:
‘Here, using only wild type data (ignoring all mutants) led to some very inaccurate predictions, especially of

large phenotypic shifts caused by experimental mutagenesis of invertebrate opsins (Figure 3), suggesting the genotype-phenotype space is still undersampled for invertebrates. Given this currently limited dataset, we do not put high confidence in the λmax estimates of both wild-type and mutant invertebrate (rhabdomeric) opsins. Therefore, targeting invertebrate opsins should be a high priority for new additions to VPOD.'

- Figure 2 legend: Third line, why 'Mutant predictions …'? Aren't the predictions for all sequences?

***Thank you, we fixed this typo.

- Figure 3 legend: It says 547 mutant sequences here and 546 sequences in Table 1.

***Thank you, we fixed this typo.

- Provide a reference for the following sentence: 'The WT SWS/UVS model similarly highlighted p113, a site functionally characterized as the counterion in the retinal-opsin Schiff base interaction for all vertebrate opsins.

***We have added the citations as requested.

- Figure 4 legend: Please provide references for the following sentence: 'Positions 181, 261 and 308 are highlighted because they are among the highest scoring sites and have all been previously characterized as functionally important to opsin phenotype and function.'

***We have added the citations as requested.

Discussion

- Please simplify and do not overstate the first sentence. I suggest: 'To better understand methods to connect genes and their functions, we initiated VPOD, a database of opsin genes and corresponding spectral sensitivity phenotypes.'

**Thank you, we have modified the first sentence exactly as suggested.

- Section: The important relationship between data availability and predictive power.

You mention that ML could not accurately predict spectral sensitivity if mutant genes were excluded, especially if smaller datasets are used. This was to be expected since ML is not per-se 'smart' but learns from patterns in the underlying dataset. However, it is a significant drawback of the approach, and I encourage you to state this more clearly. My main concern is that future users will take the ML predictions as absolute truth instead of verifying or experimentally verifying the predictions.

***We thank the reviewer for their suggestions and have added the following sentence to the discussion section as suggested under the heading 'The important relationship between data availability and predictive power'

"This was to be expected since ML is not per-se 'smart' but learns from patterns in the underlying dataset; a significant drawback for the ML approach, especially in systems or taxonomic groups lacking sufficient or reliable data."

Here is the sentence in context:
"In contrast, adding mutant data to the sparsely sampled invertebrate opsins made a big difference. Here, using only wild type data (ignoring all mutants) led to some very inaccurate predictions, especially of large phenotypic shifts caused by experimental mutagenesis of invertebrate opsins (Figure 3), suggesting the genotype-phenotype space is still undersampled for invertebrates. This was to be expected since ML is not per-se 'smart' but learns from patterns in the underlying dataset; a significant drawback for the ML approach, especially in systems or taxonomic groups lacking sufficient or reliable data. Thus, given this

currently limited dataset, we do not put high confidence in the λmax estimates of both wild-type and mutant invertebrate (rhabdomeric) opsins. Therefore, targeting invertebrate opsins should be a high priority for new additions to VPOD. ”

Additionally, we've added the following sentences to the conclusion to further encourage the use of caution when extrapolating the predictions from ML models.

“Finally, we once again caution against treating predictions of λmax as an absolute truth, as the quantity, and even more so, the quality of the genotype-phenotype data used to train a model, including the taxonomic, genetic, and phenotypic diversity, is integral to the reliability of a model's predictions. Thus, ML models such as the ones presented in this publication should be treated as summaries of all the available knowledge for a specific subject; making predictions based on a set of established priors and acting as a supplemental tool in addition to traditional methods of experimental verification.”

- Provide a reference for the following sentence: 'One consequence of leaf-based tree construction is that due to its faster convergence/training time, it can be more prone to overfitting, as it constructs trees on a 'best-first basis' with a fixed number of n-terminal nodes.'

***We have added the citations as requested.

- You should include some information regarding the assumptions in the Introduction and the Methods section. For example, information about what chromophore interaction was modelled should be in the methods, and the basic information about how visual pigments are formed and what different chromophore types are being used by which species should be in the Introduction: 'We also assume the photopigment uses 11-cis-retinal, as all heterologously expressed opsins in VPOD were reconstituted using this chromophore. However, this assumption is violated in some organisms because they use 13-cis-retinal as the in-vivo chromophore [71-73], which is associated with a red-shift in λmax [32,71].'

***We thank the reviewer for their suggestion and have added the following sentences to our introduction:
“Opsins are a family of G-protein Coupled Receptors (GPCR) which bind to a retinal chromophore. The two units together, opsin and chromophore, form visual pigments which absorb photons [23].”

“Although other factors sometimes affect spectral responsiveness, including the type of chromophore to which an opsin is covalently bound (11-cis retinal or 11-cis-3,4-didehydro retinal) [39,40], opsins provide a rare case where an intrinsic molecular function extends rather directly to organismal phenotypes, especially those involving color sensitivity. ”

***Additionally, we added this sentence to the methods section under the header ‘Compiling a genotype-phenotype database for animal opsins’:
“Note, we do not record the chromophore used to reconstitute the purified opsin protein because 11-cis retinal is the standard, and all instances recorded in the ‘heterologous’ table are from experiments which utilized 11-cis retinal (although future iterations of VPOD could record these details if this a sufficient amount of heterologous data which utilizes alternative chromophores becomes available).”

Conclusion

- I recommend being more cautious about the predictive power for epistatic effects since you tested it only on three samples and the predictions were severely restricted by the training dataset containing the single mutant samples.
***We thank the reviewer for their suggestion and note we have addressed this issue above. Please see above for Reviewer 2's comment on epistasis and our experimental response.

--
Please also take a moment to check our website at https://www.editorialmanager.com/giga/l.asp?i=156956&l=8V0W4WDS for any additional comments that were saved as attachments. Please note that as GigaScience has a policy of open peer review, you will be able to see the names of the reviewers.
