# Supplementary material for: Discovering genotype–phenotype relationships with machine learning and the Visual Physiology Opsin Database (VPOD)
Source: Gigascience. 2024 Oct 26;13:giae073. doi: 10.1093/gigascience/giae073 (PMC11512451; doi:10.1093/gigascience/giae073)
Supplement: giae073_Response_to_Reviewer_Comments_Revision_1 [file giae073_response_to_reviewer_comments_revision_1.pdf]

Author's Response To Reviewer Comments

Please include a point-by-point within the 'Response to Reviewers' box in the submission system. Please ensure you describe additional experiments that were carried out and include a detailed rebuttal of any criticisms or requested revisions that you disagreed with. Please also ensure that your revised manuscript conforms to the journal style, which can be found in the Instructions for Authors on the journal homepage. If the data and code has been modified in the revision process please be sure to update the public versions of this too.

We thank the reviewers and editor for their time and comments on our manuscript. Herein we respond to the comments inline, address concerns, and note specific changes to the manuscript. Our responses begin with ****. Reviewer suggestions begin with —-

Sincerely,

Seth A. Frazer et al.

Reviewer reports:

—Reviewer #2: The authors have addressed the majority of my concerns and improved the manuscript. However, there are still some remaining issues that the authors should address before I would recommend the publication of the manuscript.

1. Dependence between data points is not a novel problem for data analysis and machine learning in a broad range of subjects. While I appreciate that the authors added a paragraph discussing the issue of phylogenetic relatedness, the setup of the cross-validation and how the data is presented make it difficult to assess to which extend their models over-fit to the data. Referring to their previous reply, lineage-/group-based cross-validation should not be arbitrarily chosen but based on the structure of the data. This is not always a trivial problem and magic solution, I agree. The authors should at least incorporate references to literature discussing the problem and potential solutions for dealing with phylogenetic relatedness at p.11 "While these performance metrics are impressive, it is important to remember that phylogenetic relatedness between sequences..." or in the discussion. For example, Roberts et al. provide a nice overview for cross-validation strategies in
various settings including phylogenetic data (they call it "block cross-validation"):

Roberts et al. (2017). Cross-validation strategies for data with temporal, spatial, hierarchical, or phylogenetic structure. Ecography, 40(8), 913-929.

**** We thank the reviewer for this comment and we agree that this is an important issue to address within the future directions of our research to unpack the extent to which we can extrapolate/rely on predictions made by these models on new data. As suggested by the reviewer, we now added the suggested reference and the sentence "Roberts et al. [79] provide a discussion of alternative cross-validation strategies such as "block cross-validation" for non-independent data types, including phylogenetically related data which can help mitigate this issue".

—2. Regarding the comparison between models trained on wild type (WT) and WT + mutant data (WDS), I find the comparison rather difficult to follow concerning repeatedly leaving out 25 mutants. For comparing the models, the test set (or test sets) should be the same. This would mean to assess the predictions for the same 25 left out mutants by both the WT and the WDS model (for each 25 left out mutants). If this

was done already I would recommend rephrasing the corresponding part in the methods and results to improve the clarity.

**** We thank the reviewer for their insight into the interpretability of this experiment and would like to note that the WT model has no mutants at all in its training so it automatically leaves out the 25 mutants removed in each iteration of training that the WDS model goes through.
To clarify/rephrase the methods further - The WT model is ONLY trained on WT opsin data, thus can simply be tested on all mutants at once AFTER training of the model is complete - it is not used as test data during the actual model training. The WDS in this test is iteratively trained on the entire dataset with only 25 mutants removed at a time and used as test data following the completion of training, so it is not used as test data during the actual training process. This is repeated until all mutants from the WDS have been sampled and tested once.

Here is our modification to that section in context:
"To accomplish this for the WDS models, we iteratively removed 25 mutant opsins at a time from training data, used the same training algorithm (Gradient Boosted Regressor, GBR), and predicted λmax values of withheld opsins following the completion of model training (withheld opsins are not used as test data during the actual model training), until all mutant opsins were sampled once (this notebooks is available on GitHub as 'vpod_wf_iterate_subsample.ipynb'."

We did not modify the corresponding results section as the methods underlying this iterative process are not directly mentioned there.

—In addition, a visualization, for instance, a violin plot of the WT model RMSEs vs. violin plot of the WDS model RMSEs would be useful for the readers.

**** We have added two supplementary figures using cloud plots to visualize the absolute error distributions of the WT vs WDS mutant prediction test (Figure S11) and another for the test comparing the prediction error of the Expected Additive Mutation Values, WT model, and WDS-minusepi model (Figure S12). We've also referenced them in the main text in their corresponding sections.

—3. I would still recommend adding a brief summary of how feature importance scores are computed. So, the reader does not have to look up another manuscript. This does not have to be detailed. As I understand, the feature importance is just the normalized number of feature occurrences or the Gini importance for gradient boosting/random forests or the coefficient for regression models.

**** We have added a slightly more detailed explanation of how importance scores are computed and have provide it below:
"The metrics used to determine these relative importance scores of each position vary based on the structure and output of the algorithms used for model training. For example, xgboost [60] and LightGBM [61,62] use the number of times a feature appears in a tree as a proxy for importance [59], while AdaBoost [63] and random forest [64,65], use Gini importance, which quantifies a feature's contribution to improving prediction accuracy [59,66,67]. For a more detailed explanation on how position importance scores are calculated for different models, refer to the 'Interpretation' heading under the methods section of the deepBreaks publication [59]."

—Minor details:

—Fig. S10: the text at the leaves is not readable. It could be replaced, for instance, with the name of the gene family if that makes sense, or removed.

**** We appreciate the suggestion made by the reviewer but we've decided to not remove text at the leaves and have instead supplied a higher resolution image of the tree in the supplement, namely as a vector-based graphic, for the tree so that readers can zoom in on the tips more clearly, if so desired.

—Fig. 4A: the bars at position 181, 261, and 308, could be indicated, for example, in red or another color,

to easier compare A and B

**** We have modified figure 4A and 4B as suggested by changing the color of the bars at position 181, 261, and 308 to purple for the sake of easier comparison.
