# Supplementary material for: Discovering genotype–phenotype relationships with machine learning and the Visual Physiology Opsin Database (VPOD)
Source: Gigascience. 2024 Oct 26;13:giae073. doi: 10.1093/gigascience/giae073 (PMC11512451; doi:10.1093/gigascience/giae073)
Supplement: giae073_Response_to_Reviewer_Comments_Revision_2 [file giae073_response_to_reviewer_comments_revision_2.pdf]

Author's Response To Reviewer Comments

We made all the changes to document indicated by the editor, namely adding citations to our database and docker image resources.
