# Supplementary material for: Discovering genotype–phenotype relationships with machine learning and the Visual Physiology Opsin Database (VPOD)
Source: Gigascience. 2024 Oct 26;13:giae073. doi: 10.1093/gigascience/giae073 (PMC11512451; doi:10.1093/gigascience/giae073)
Supplement: giae073_Reviewer_1_Report_Original_Submission — Robert Lucas -- 3/28/2024 Reviewed [file giae073_reviewer_1_report_original_submission.pdf]

Reviewer Report

Title: Discovering genotype-phenotype relationships with machine learning and the Visual Physiology Opsin Database (VPOD)

Version: Original Submission **Date:** 3/28/2024

Reviewer name: Robert Lucas

Reviewer Comments to Author:

Frazer and colleagues set out to assess the ability of machine learning (ML) approaches to predict spectral sensitivity (lmax) of animal opsins from their amino acid sequence. To this end they first develop a database of phenotyped opsins (opsin sequences with known lmax), which they term Vpod1. They then explore how various factors of the ML process impact its ability to predict lmax. These include the nature of the input training dataset (size, phylogenetic and gene family diversity, inclusion of data from mutagenesis experiments) and the ML method. For comparison they include a phylogenetic imputation approach that predicts lmax based upon overall sequence similarity. They test the validity of their approach according to their ML pipelines' ability to predict: lmax for the training dataset; the outcome of mutagenesis; lmax for a test dataset extracted from the training dataset; known epistatic interactions; and established spectral tuning sites. In all cases, they report various degrees of success and conclude that the ML approach can be used to predict lmax (almost as well as phylogenetic imputation but with reduced computational cost) provided that the training dataset is sufficiently rich (it performs poorly for invertebrate opsins for which data are limited) and, ideally, benefits from mutagenesis datasets.

I am no expert in machine learning and will leave others to comment on that aspect of methodology but in general this study represents an interesting addition to the literature. The idea of predicting lmax from amino acid sequence is not new e.g. as the authors acknowledge the '5 sites rule' for cone pigments is long established. Applying ML holds the promise of a more efficient process for achieving similar predictability for other branches of the animal opsin family. In that regard, the inherent limitation in the ML approach is highlighted - it is particularly valuable in branches of the family for which information is sparse (invertebrate opsins), but performs poorly in those branches without more starting information about structure:function relationships (which itself replaces the need for ML to some extent). Nonetheless, it certainly has the potential to be a valuable tool and this paper represents a sound exploration of its characteristics and one important feature of the paper is that it confirms that ML can allow fairly good predictions based solely on data from wildtype opsin sequences.

I have relatively few suggestions for improvement. The most important is that the authors appear to have omitted one technology for the process of defining lmax (introduction, method and discussion). We and others have used heterologous action spectroscopy to describe lmax for a growing number of animal opsins. In this technique spectral sensitivity is defined using live cell assays of light response for opsins expressed in immortalised cell lines. Those data could be included in the Vpod1 dataset. It would also be appropriate to mention the approach as a tool for populating the training dataset as it has the advantage of being applicable to opsins that don't reliably form pigments in vitro (e.g. many

invertebrate opsins) and does not rely on access to the animal itself but only to its genome sequence. The authors also may wish to relate Vpod1 to another recently published database of animal spectral sensitivities albeit collected for a different purpose (https://doi.org/10.1016/j.baae.2023.09.002). Some minor points. The authors note with surprise that ML performed poorly for the rod opsin dataset. Could this be because their metric (R2) is sensitive to the degree of variability in lmax in the training dataset, which is constrained in rod opsins? I found the pastel colours in Fig2 and 3 hard to discern, more separation on the colour pallet would be appreciated.

Methods

Are the methods appropriate to the aims of the study, are they well described, and are necessary controls included? Choose an item.

Conclusions

Are the conclusions adequately supported by the data shown? Choose an item.

Reporting Standards

Does the manuscript adhere to the journal's guidelines on minimum standards of reporting? Choose an item.

Choose an item.

Statistics

Are you able to assess all statistics in the manuscript, including the appropriateness of statistical tests used? Choose an item.

Quality of Written English

Please indicate the quality of language in the manuscript: Choose an item.

Declaration of Competing Interests

Please complete a declaration of competing interests, considering the following questions:

- Have you in the past five years received reimbursements, fees, funding, or salary from an organisation that may in any way gain or lose financially from the publication of this manuscript, either now or in the future?
- Do you hold any stocks or shares in an organisation that may in any way gain or lose financially from the publication of this manuscript, either now or in the future?
- Do you hold or are you currently applying for any patents relating to the content of the manuscript?

- Have you received reimbursements, fees, funding, or salary from an organization that holds or has applied for patents relating to the content of the manuscript?
- Do you have any other financial competing interests?
- Do you have any non-financial competing interests in relation to this paper?

If you can answer no to all of the above, write 'I declare that I have no competing interests' below. If your reply is yes to any, please give details below.

None

I agree to the open peer review policy of the journal. I understand that my name will be included on my report to the authors and, if the manuscript is accepted for publication, my named report including any attachments I upload will be posted on the website along with the authors' responses. I agree for my report to be made available under an Open Access Creative Commons CC-BY license (http://creativecommons.org/licenses/by/4.0/). I understand that any comments which I do not wish to be included in my named report can be included as confidential comments to the editors, which will not be published.

Choose an item.

To further support our reviewers, we have joined with Publons, where you can gain additional credit to further highlight your hard work (see: https://publons.com/journal/530/gigascience). On publication of this paper, your review will be automatically added to Publons, you can then choose whether or not to claim your Publons credit. I understand this statement.

Yes Choose an item.
