# Supplementary material for: Discovering genotype–phenotype relationships with machine learning and the Visual Physiology Opsin Database (VPOD)
Source: Gigascience. 2024 Oct 26;13:giae073. doi: 10.1093/gigascience/giae073 (PMC11512451; doi:10.1093/gigascience/giae073)
Supplement: giae073_Reviewer_2_Report_Original_Submission — Nikolai Hecker -- 4/4/2024 Reviewed [file giae073_reviewer_2_report_original_submission.pdf]

Reviewer Report

Title: Discovering genotype-phenotype relationships with machine learning and the Visual Physiology Opsin Database (VPOD)

Version: Original Submission **Date:** 4/4/2024

Reviewer name: Nikolai Hecker

Reviewer Comments to Author:

The authors compiled a collection of opsin/rhodopsin proteins and their associated light absorption properties from literature; using the measured wavelength of maximum absorbance as a proxy. In addition, they include multiple sequence alignments (MSA) of the proteins including subsets of vertebrate and invertebrate sequences. The data is provided as tab-seperated, comma-seperated, and FASTA files. This is a valuable resource for studying opsins and vision related phenotypes. The authors then use gradient-boosting, random forests, and bayesian ridge regression to predict the wavelength of maximum absorbance from the protein sequence MSAs. Furthermore, they investigate whether their models can be used to identify amino acid changes that impact the wavelength of maximum absorbance and epistasis. This is based on a small set of opsin mutants that have been reported in literature. The manuscript is well structured and written. I have some concerns regarding the analysis, description and presentation of the data.

1. A traditional cross-validation by random sampling can be inadequate for phylogenetically related sequences. If closely related species are part of the data training and test sets may contain nearly identical sequences. Excluding entire lineages instead of random sequences during training would circumvent this issue.
2. Based on Fig. 3, Fig. 2, and p. 6, the models do not generalize well given that they only predict mutants well which exhibit a similar wavelength of maximum absorbance as the wild type. Based on the plots (Fig. 2 and Fig. 3) it does not look to me like the model trained on mutants+WT performs substantially better than the WT model for mutants with large wavelength shifts. This would be in contrast to p.16 "Particularly illustrative of these ideas are our analyses with and without experimentally mutated opsins". The authors should either show statistics regarding the improved performance for mutants with large shifts or change the corresponding parts.
3. The data set description should be more detailed in parts. It should be shown how the opsins/rhodopsins classes (UVS, SWS, MWS, Rhodopsins, LWS) are distributed across the vertebrate and invertebrate phylogeny, for example by a phylogenetic tree and their number per species. Are the mutated opsins/rhodopsins derived from a small set of species or do they reflect most of the vertebrate phylogeny?
4. How importance scores are estimated for the different models should be explained.
5. The "ML often predicts the effects of epistatic mutations" section needs some clarifications. Why were only three sequences investigated? Do none of the other double mutants show epistasis when compared with the corresponding single mutations? In this paragraph, it is not always clear whether wavelengths and additive wavelengths are obtained from predictions or actual measurements.

6. The description in git repository (https://github.com/VisualPhysiologyDB/visual-physiology-opsin-db) is very sparse. The content of the different files and how they relate to each other should at least be briefly explained in a README. It would also be helpful to add gene names, and the source of the sequence to the meta files.

Minor comments

1. For Fig. 4A, since MSAs are already computed it would be interesting to indicate the conservation amino acids per position. Are important amino acids correlated with sequence conservation?
2. In Tab. 1, R2 is used to compare different models which are based on different subsets, and also pot. differently sized MSAs. An adjusted R2 might be more suitable to account for different numbers of features.
3. It would be helpful to add a Docker image to the github repository to make it easier to use

Methods

Are the methods appropriate to the aims of the study, are they well described, and are necessary controls included? Choose an item.

Conclusions

Are the conclusions adequately supported by the data shown? Choose an item.

Reporting Standards

Does the manuscript adhere to the journal's guidelines on [minimum standards of reporting?]() Choose an item.

Choose an item.

Statistics

Are you able to assess all statistics in the manuscript, including the appropriateness of statistical tests used? Choose an item.

Quality of Written English

Please indicate the quality of language in the manuscript: Choose an item.

Declaration of Competing Interests

Please complete a declaration of competing interests, considering the following questions:

- Have you in the past five years received reimbursements, fees, funding, or salary from an organisation that may in any way gain or lose financially from the publication of this manuscript, either now or in the future?

- Do you hold any stocks or shares in an organisation that may in any way gain or lose financially from the publication of this manuscript, either now or in the future?
- Do you hold or are you currently applying for any patents relating to the content of the manuscript?
- Have you received reimbursements, fees, funding, or salary from an organization that holds or has applied for patents relating to the content of the manuscript?
- Do you have any other financial competing interests?
- Do you have any non-financial competing interests in relation to this paper?

If you can answer no to all of the above, write 'I declare that I have no competing interests' below. If your reply is yes to any, please give details below.

I declare that I have no competing interests

I agree to the open peer review policy of the journal. I understand that my name will be included on my report to the authors and, if the manuscript is accepted for publication, my named report including any attachments I upload will be posted on the website along with the authors' responses. I agree for my report to be made available under an Open Access Creative Commons CC-BY license (http://creativecommons.org/licenses/by/4.0/). I understand that any comments which I do not wish to be included in my named report can be included as confidential comments to the editors, which will not be published.

Choose an item.

To further support our reviewers, we have joined with Publons, where you can gain additional credit to further highlight your hard work (see: https://publons.com/journal/530/gigascience). On publication of this paper, your review will be automatically added to Publons, you can then choose whether or not to claim your Publons credit. I understand this statement.

Yes Choose an item.
