# Supplementary material for: Discovering genotype–phenotype relationships with machine learning and the Visual Physiology Opsin Database (VPOD)
Source: Gigascience. 2024 Oct 26;13:giae073. doi: 10.1093/gigascience/giae073 (PMC11512451; doi:10.1093/gigascience/giae073)
Supplement: giae073_Reviewer_2_Report_Revision_1 — Nikolai Hecker -- 7/9/2024 Reviewed [file giae073_reviewer_2_report_revision_1.pdf]

Reviewer Report

Title: Discovering genotype-phenotype relationships with machine learning and the Visual Physiology Opsin Database (VPOD)

Version: Revision 1 **Date:** 7/9/2024

Reviewer name: Nikolai Hecker

Reviewer Comments to Author:

The authors have addressed the majority of my concerns and improved the manuscript. However, there are still some remaining issues that the authors should address before I would recommend the publication of the manuscript.

1. Dependence between data points is not a novel problem for data analysis and machine learning in a broad range of subjects. While I appreciate that the authors added a paragraph discussing the issue of phylogenetic relatedness, the setup of the cross-validation and how the data is presented make it difficult to assess to which extend their models over-fit to the data. Referring to their previous reply, lineage-/group-based cross-validation should not be arbitrarily chosen but based on the structure of the data. This is not always a trivial problem and magic solution, I agree. The authors should at least incorporate references to literature discussing the problem and potential solutions for dealing with phylogenetic relatedness at p.11 "While these performance metrics are impressive, it is important to remember that phylogenetic relatedness between sequences..." or in the discussion. For example, Roberts et al. provide a nice overview for cross-validation strategies in various settings including phylogenetic data (they call it "block cross-validation"):
Roberts et al. (2017). Crossâ€validation strategies for data with temporal, spatial, hierarchical, or phylogenetic structure. Ecography, 40(8), 913-929.
2. Regarding the comparison between models trained on wild type (WT) and WT + mutant data (WDS), I find the comparison rather difficult to follow concerning repeatably leaving out 25 mutants. For comparing the models, the test set (or test sets) should be the same. This would mean to assess the predictions for the same 25 left out mutants by both the WT and the WDS model (for each 25 left out mutants). If this was done already I would recommend rephrasing the corresponding part in the methods and results to improve the clarity. In addition, a visualization, for instance, a violin plot of the WT model RMSEs vs. violin plot of the WDS model RMSEs would be useful for the readers.
3. I would still recommed adding a brief summary of how feature importance scores are computed. So, the reader does not have to look up another manuscript. This does not have to be detailed. As I understand, the feature importance is just the normalized number of feature occurrences or the Gini importance for gradient boosting/random forests or the coefficient for regression models.

Minor details:
Fig. S10: the text at the leaves is not readable. It could be replaced, for instance, with the name of the gene family if that make sense, or removed.
Fig. 4A: the bars at position 181, 261, and 308, could be indicated, for example, in red or another color,

to easier compare A and B

Methods

Are the methods appropriate to the aims of the study, are they well described, and are necessary controls included? Choose an item.

Conclusions

Are the conclusions adequately supported by the data shown? Choose an item.

Reporting Standards

Does the manuscript adhere to the journal's guidelines on [minimum standards of reporting?]() Choose an item.

Choose an item.

Statistics

Are you able to assess all statistics in the manuscript, including the appropriateness of statistical tests used? Choose an item.

Quality of Written English

Please indicate the quality of language in the manuscript: Choose an item.

Declaration of Competing Interests

Please complete a declaration of competing interests, considering the following questions:

- Have you in the past five years received reimbursements, fees, funding, or salary from an organisation that may in any way gain or lose financially from the publication of this manuscript, either now or in the future?
- Do you hold any stocks or shares in an organisation that may in any way gain or lose financially from the publication of this manuscript, either now or in the future?
- Do you hold or are you currently applying for any patents relating to the content of the manuscript?
- Have you received reimbursements, fees, funding, or salary from an organization that holds or has applied for patents relating to the content of the manuscript?
- Do you have any other financial competing interests?
- Do you have any non-financial competing interests in relation to this paper?

If you can answer no to all of the above, write 'I declare that I have no competing interests' below. If your reply is yes to any, please give details below.

I declare that I have no competing interests

I agree to the open peer review policy of the journal. I understand that my name will be included on my report to the authors and, if the manuscript is accepted for publication, my named report including any attachments I upload will be posted on the website along with the authors' responses. I agree for my report to be made available under an Open Access Creative Commons CC-BY license (http://creativecommons.org/licenses/by/4.0/). I understand that any comments which I do not wish to be included in my named report can be included as confidential comments to the editors, which will not be published.

Choose an item.

To further support our reviewers, we have joined with Publons, where you can gain additional credit to further highlight your hard work (see: https://publons.com/journal/530/gigascience). On publication of this paper, your review will be automatically added to Publons, you can then choose whether or not to claim your Publons credit. I understand this statement.

Yes Choose an item.
