# Supplementary material for: Discovering genotype–phenotype relationships with machine learning and the Visual Physiology Opsin Database (VPOD)
Source: Gigascience. 2024 Oct 26;13:giae073. doi: 10.1093/gigascience/giae073 (PMC11512451; doi:10.1093/gigascience/giae073)
Supplement: giae073_Reviewer_3_Report_Original_Submission — Fabio Cortesi -- 4/14/2024 Reviewed [file giae073_reviewer_3_report_original_submission.pdf]

Reviewer Report

Title: Discovering genotype-phenotype relationships with machine learning and the Visual Physiology Opsin Database (VPOD)

Version: Original Submission **Date:** 4/14/2024

Reviewer name: Fabio Cortesi

Reviewer Comments to Author:

In their manuscript, Frazer et al. developed a machine-learning approach to predict the spectral sensitivity of a visual pigment based on the gene/amino acid sequence of the opsin protein. First, they created a visual opsin database based on heterologously expressed genes from the literature. They then used deepBreaks, an ML tool developed to explore genotype-phenotype associations, to run several different models and test how well ML could predict spectral sensitivity. Their main findings are that the larger the dataset for training and the more diverse (both in opsin sequences themselves and phylogenetic breadth they were derived from) the dataset, the better the predictions will become. However, there is a plateau for the number of training sequences that should be used as a minimum (~ 200), with a slight gain afterwards. As such, the suggested ML approach works well for larger datasets but needs refining for smaller datasets. There are also several drawbacks to the approach that need to be carefully considered when interpreting the results, including the fact that ML cannot accurately predict the effect on phenotype if confronted with a new mutation or a new combination of mutations not used during training.
I found the study to be well-written and easy to follow. The results support the conclusions, and as far as I can tell, the ML and associated analysis were performed accurately. All the code and the database are readily accessible, too. It is great to see that we are at a point now where computational power has reached a level that can be used to predict gene-phenotype relationships accurately. The use of ML to study the function of (visual) opsins, i.e., spectral sensitivity, especially if additional parameters can be included, will undoubtedly be of great help to the field and welcomed by the community. As such, I have no major concerns and only a few minor comments I recommend addressing before publication.
Minor comments
Introduction
- Please add a sentence to explain that a visual pigment consists of an opsin protein bound to a chromophore/retinal and that the two units together lead to the 'spectral sensitivity' phenotype. You cover it in the discussion, but it would be helpful for the reader to have this information upfront.
- Please provide a reference for the following statement: '[…], and purification of heterologously expressed opsins followed by spectrophotometry [REF]'.
- You say, 'Despite opsins being a well-studied system with an extensive backlog of published literature, previous authors expressed doubts that sequence data alone can provide reliable computational predictions of Î»max phenotypes [37-40]'.
I agree that the spectral sensitivity predictions from sequences have been criticised in the past as they were sometimes oversimplified (including some of our work). However, spectral sensitivity predictions

based on computational modelling, albeit not using ML, have previously been attempted successfully several times, e.g., by Jagdish Suresh Patel and colleagues, and should be mentioned here.
- You say that: 'The extensive data on animal opsin genotype-phenotype associations remains disorganized, decentralized, often in non-computer readable formats in older literature, and under-analyzed computationally'.
Again, I agree that the opsin data can profit from a centralised databank like the one you created. However, there have been several previous attempts at summarizing opsin data in recent years (although not specific for heterologously expressed opsins), for vertebrates at least. For example, work by Schweikert and colleagues on fish visual opsins and recent work on frog opsins by Schott et al. These studies should be mentioned and cited appropriately here, as tremendous work went into collating the datasets in the first place.
Results
- The use of MWS opsin is somewhat confusing. I presume this refers to vertebrate lws genes that are mid-wavelength shifted? Why have these as a separate group? Ancestrally, there are five sub-families of visual opsin genes in vertebrates: sws1 & sws2 (SWS), rh1, rh2 and lws (MWS & LWS). The MWS range in Figure 1 should be part of a larger lws derived grouping.
- This part reads like a discussion. It also needs a reference for the age of T1 opsins: 'The similar levels of performances between T1 and invertebrate models were unexpected, especially considering it has a training dataset five times larger than the invertebrate model. One possible explanation is that the very old age of T1 opsins [REF] might have led to a higher complexity of genotype-phenotype associations that are not yet well sampled enough to allow good predictions.'
- These two sentences could also be weaved into the discussion rather than the results section: 'These equations do not account directly for taxonomic, genetic, or phenotypic diversity, as the number of genes is on the x-axis. Therefore, one should be cautious about applying them to predict model performance based on training data size alone.'
- Table 1: What do MAPE and RMSE stand for, and what do those numbers mean? Maybe also include a short explanation of the acronyms and their meaning in the main body of the text.
- This should also be mentioned in the discussion: 'Until the models are trained with more invertebrate (r-opsin) data, we do not put high confidence in the estimates of Î»max.'
- Figure 2 legend: Third line, why 'Mutant predictions …'? Aren't the predictions for all sequences?
- Figure 3 legend: It says 547 mutant sequences here and 546 sequences in Table 1.
- Provide a reference for the following sentence: 'The WT SWS/UVS model similarly highlighted p113, a site functionally characterized as the counterion in the retinal-opsin Schiff base interaction for all vertebrate opsins.
- Figure 4 legend: Please provide references for the following sentence: 'Positions 181, 261 and 308 are highlighted because they are among the highest scoring sites and have all been previously characterized as functionally important to opsin phenotype and function.'
Discussion
- Please simplify and do not overstate the first sentence. I suggest: 'To better understand methods to connect genes and their functions, we initiated VPOD, a database of opsin genes and corresponding spectral sensitivity phenotypes.'
- Section: The important relationship between data availability and predictive power.

You mention that ML could not accurately predict spectral sensitivity if mutant genes were excluded, especially if smaller datasets are used. This was to be expected since ML is not per-se 'smart' but learns from patterns in the underlying dataset. However, it is a significant drawback of the approach, and I encourage you to state this more clearly. My main concern is that future users will take the ML predictions as absolute truth instead of verifying or experimentally verifying the predictions.
- Provide a reference for the following sentence: 'One consequence of leaf-based tree construction is that due to its faster convergence/training time, it can be more prone to overfitting, as it constructs trees on a 'best-first basis' with a fixed number of n-terminal nodes.'
- You should include some information regarding the assumptions in the Introduction and the Methods section. For example, information about what chromophore interaction was modelled should be in the methods, and the basic information about how visual pigments are formed and what different chromophore types are being used by which species should be in the Introduction: 'We also assume the photopigment uses 11-cis-retinal, as all heterologously expressed opsins in VPOD were reconstituted using this chromophore. However, this assumption is violated in some organisms because they use 13-cis-retinal as the in-vivo chromophore [71-73], which is associated with a red-shift in Î»max [32,71].'
Conclusion
- I recommend being more cautious about the predictive power for epistatic effects since you tested it only on three samples and the predictions were severely restricted by the training dataset containing the single mutant samples.

Methods

Are the methods appropriate to the aims of the study, are they well described, and are necessary controls included? Choose an item.

Conclusions

Are the conclusions adequately supported by the data shown? Choose an item.

Reporting Standards

Does the manuscript adhere to the journal's guidelines on [minimum standards of reporting?]() Choose an item.

Choose an item.

Statistics

Are you able to assess all statistics in the manuscript, including the appropriateness of statistical tests used? Choose an item.

Quality of Written English

Please indicate the quality of language in the manuscript: Choose an item.

Declaration of Competing Interests

Please complete a declaration of competing interests, considering the following questions:

- Have you in the past five years received reimbursements, fees, funding, or salary from an organisation that may in any way gain or lose financially from the publication of this manuscript, either now or in the future?
- Do you hold any stocks or shares in an organisation that may in any way gain or lose financially from the publication of this manuscript, either now or in the future?
- Do you hold or are you currently applying for any patents relating to the content of the manuscript?
- Have you received reimbursements, fees, funding, or salary from an organization that holds or has applied for patents relating to the content of the manuscript?
- Do you have any other financial competing interests?
- Do you have any non-financial competing interests in relation to this paper?

If you can answer no to all of the above, write 'I declare that I have no competing interests' below. If your reply is yes to any, please give details below.

I declare that I have no competing interests.

I agree to the open peer review policy of the journal. I understand that my name will be included on my report to the authors and, if the manuscript is accepted for publication, my named report including any attachments I upload will be posted on the website along with the authors' responses. I agree for my report to be made available under an Open Access Creative Commons CC-BY license (http://creativecommons.org/licenses/by/4.0/). I understand that any comments which I do not wish to be included in my named report can be included as confidential comments to the editors, which will not be published.

Choose an item.

To further support our reviewers, we have joined with Publons, where you can gain additional credit to further highlight your hard work (see: https://publons.com/journal/530/gigascience). On publication of this paper, your review will be automatically added to Publons, you can then choose whether or not to claim your Publons credit. I understand this statement.

Yes Choose an item.
